# C–H arylation and alkenylation of imidazoles by nickel catalysis: solvent-accelerated imidazole C–H activation†
†Electronic supplementary information (ESI) available. See DOI: 10.1039/c5sc02942b


**DOI:** 10.1039/c5sc02942b

**Published:** 2015-09-08

**Authors:** Kei Muto, Taito Hatakeyama, Junichiro Yamaguchi, Kenichiro Itami

**Affiliations:** a Institute of Transformative Bio-Molecules (WPI-ITbM) and Graduate School of Science , Nagoya University , Chikusa , Nagoya 464-8602 , Japan . Email: itami@chem.nagoya-u.ac.jp ; Email: junichiro@chem.nagoya-u.ac.jp; b Central Research Laboratory Technology and Development Division , Kanto Chemicals Co. Inc. , Saitama 340-0003 , Japan; c JST , ERATO , Itami Molecular Nanocarbon Project , Nagoya University , Chikusa , Nagoya 464-8602 , Japan

## Abstract

The first nickel-catalyzed C–H arylations and alkenylations of imidazoles with phenol and enol derivatives are described. Under the influence of Ni(OTf)_2_/dcype/K_3_PO_4_ (dcype: 1,2-bis(dicyclohexylphosphino)ethane) in *t*-amyl alcohol, imidazoles can undergo C–H arylation with phenol derivatives. The C–H arylation of imidazoles with chloroarenes as well as that of thiazoles and oxazoles with phenol derivatives can also be achieved with this catalytic system. By changing the ligand to dcypt (3,4-bis(dicyclohexylphosphino)thiophene), enol derivatives could also be employed as coupling partners achieving the C–H alkenylation of imidazoles as well as thiazoles and oxazoles. Thus, a range of C2-arylated and alkenylated azoles can be synthesized using this newly developed nickel-based catalytic system. The key to the success of the C–H coupling of imidazoles is the use of a tertiary alcohol as solvent. This also allows the use of an air-stable nickel(ii) salt as the catalyst precursor.

## Abstract 2

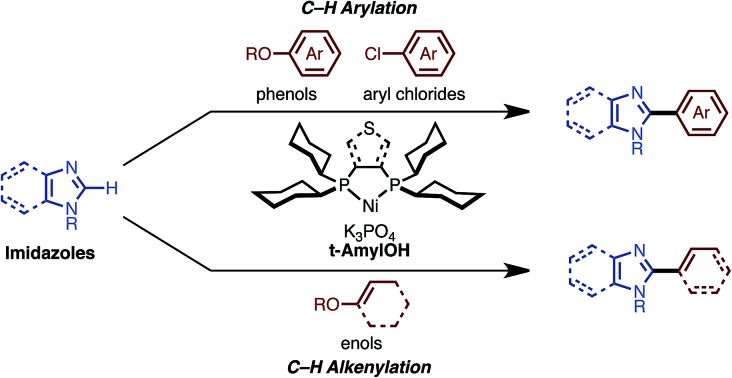
The first nickel-catalyzed C–H arylations and alkenylations of imidazoles with phenol and enol derivatives are described.

## Introduction

**Fig. 1:**
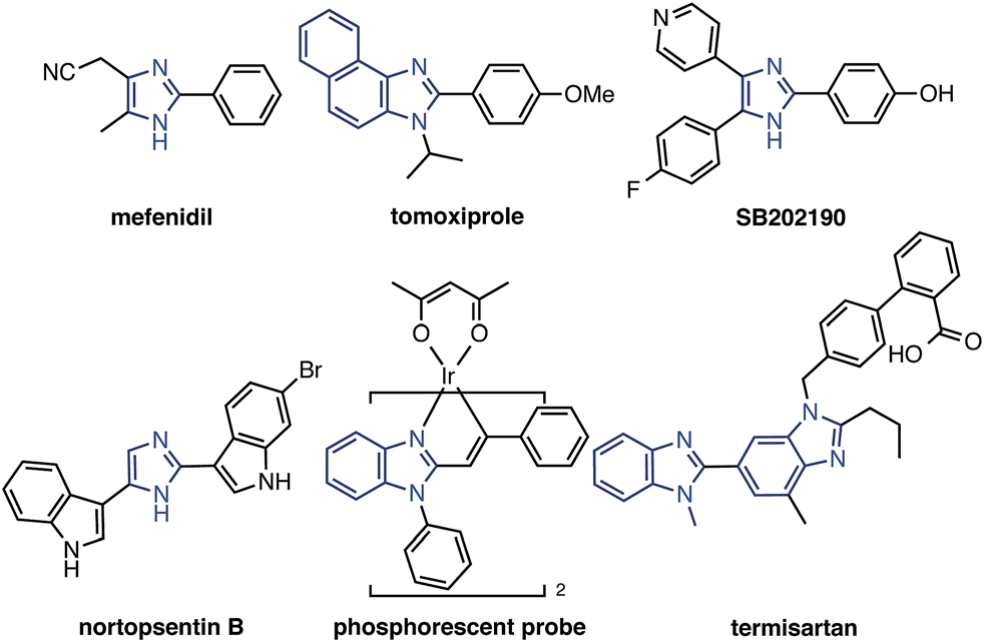
C2-arylated and alkenylated imidazoles and benzimidazoles in natural products, pharmaceuticals, and organic materials.

Imidazoles, including benzimidazoles, are recognized as important chemical motifs since they are frequently found in a range of natural products, and are exploited as core structures in pharmaceuticals, agrochemicals, and organic materials. Because of the high versatility of imidazole-containing compounds (particularly C2-arylated and alkenylated imidazoles; Fig. 1),^1^ functionalization and derivatization thereof are of significant importance in synthetic organic chemistry. Numerous synthetic methods to construct C2-aryl and alkenyl imidazoles have been reported thus far. Although cyclization and annulation reactions have found wide use, these methods often suffer from multi-step reaction sequences.^2^ Transition metal-catalyzed cross-coupling reactions of arylmetal compounds and aryl halides have also been employed, albeit requiring the pre-functionalization of metalated or halogenated imidazoles prior to the coupling reactions.^3^

In recent years, the transition metal-catalyzed C–H functionalization approach has attracted attention as it enables rapid and straightforward synthesis of various functional heteroarenes.^4^ Within this class of reactions, C–H arylations and alkenylations of imidazoles using transition-metal catalysts have been reported, mainly involving palladium^5^ and rhodium.^6^ In 2009, Daugulis and coworkers discovered the Cu-catalyzed C–H arylation of imidazoles, which allowed the use of an inexpensive transition metal catalyst.^7^

**Fig. 2:**
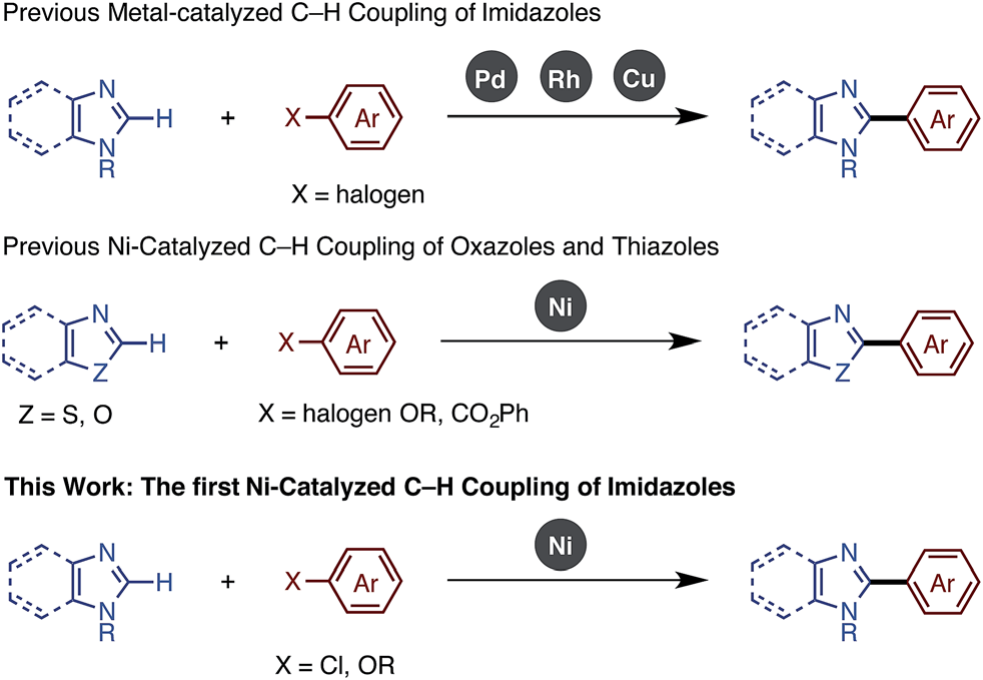
Transition metal-catalyzed C–H arylation of imidazoles and benzimidazoles.

In our studies of catalytic C–H functionalization,^8^ we have developed several unique nickel catalysts^9^ that facilitate the C–H arylation of 1,3-azoles, such as oxazoles and thiazoles, with haloarenes (C–H/C–X coupling),^10^ phenol derivatives (C–H/C–O coupling),^11^ and arenecarboxylates (decarboxylative C–H coupling).^12^ The advantages of our recent nickel-based catalytic systems^11^ are not only their low cost, but also their ability to activate and couple phenol derivatives (C–O electrophiles).^13^,^14^ However, imidazoles and benzimidazoles still remained challenging substrates for our nickel-catalyzed C–H coupling campaign. Herein, we report the first general protocol for Ni-catalyzed C–H arylation as well as alkenylation of imidazoles (Fig. 2).

## Results and discussion

### C–H arylation of imidazoles with phenol derivatives

Among the myriad of nickel catalysts for C–H arylation reported over the past decade from our group,^10^–^12^ Miura,^15^ and Chatani,^16^ our Ni(cod)_2_/dcype (cod = 1,5-cyclooctadiene; dcype = 1,2-bis(dicyclohexylphosphino)ethane) catalyst with Cs_2_CO_3_ as the base in 1,4-dioxane as the solvent is well suited for the direct coupling of 1,3-azoles and phenol derivatives (C–H/C–O coupling). The selection of an appropriate ligand is crucial, as this reaction proceeds only when dcype is used. In contrast, this catalytic system was not affected by the choice of base and solvent since C2-functionalized azoles could be formed in the absence of base. Through several mechanistic investigations including the isolation of a catalytic intermediate, kinetic studies,11*c* and theoretical calculations,11*d* we have previously identified the ligand effect and a plausible catalytic cycle for the reaction. In particular, DFT calculations provided significant insight regarding the mechanism of the C–H nickelation steps: the formation of a Cs–Ni cluster was found to play a key roll in accelerating C–H nickelation. However, experimentally and computationally, it has also been revealed that new catalytic conditions need to be developed for the C–H coupling of imidazoles.

With these considerations in mind, we focused on the investigation of base and solvent effects by using *N*-methylbenzimidazole (**1A**) and phenyl carbamate **2a** as model substrates (Table 1). Our previous Ni(cod)_2_/dcype-based catalytic conditions, employing Cs_2_CO_3_ and 1,4-dioxane, afforded no coupling products (entry 1). An intensive and thorough investigation led to the discovery that the combination of K_3_PO_4_ and *t*-amyl alcohol (*t*-amylOH) could facilitate the C–H arylation of **1A** to afford **3Aa** in 83% yield (entry 2). The replacement of *t*-amylOH with *t*-BuOH gave **3Aa** in slightly lower yield (entry 3). When the base and/or the solvent were changed, the reaction efficiency diminished. For example, the use of aprotic solvents and secondary alcohol solvents was completely ineffective for this reaction (entries 4–6). Additionally, the reaction in the presence of Cs_2_CO_3_ or LiO*t*-Bu resulted in lower yields than with K_3_PO_4_ (entries 7 and 8). While we initially thought that *in situ*-generated potassium tertiary alkoxides might be the reactive species, this seems not to be the case; the use of KO*t*-Bu completely shut down the catalytic activity (entry 9). Regarding the ligand effect, PCy_3_ and an *N*-heterocyclic carbene ligand were inactive for the present reaction (entries 10 and 11). Our thiophene-based diphosphine ligand, dcypt (3,4-bis(dicyclohexylphosphino)thiophene),^17^ as well as other dcype derivatives such as **L1** and **L2** furnished **3Aa** in moderate yields (entries 12–14). To our delight, the replacement of Ni(cod)_2_ with Ni(OTf)_2_ as the pre-catalyst maintained the catalytic activity (entry 15). Considering the significant advantage of using air-stable Ni(OTf)_2_, we decided to use the Ni(OTf)_2_/dcype catalyst and K_3_PO_4_ in *t*-amylOH for further studies.

**Table 1:** Screening of the reaction conditions^*a*^

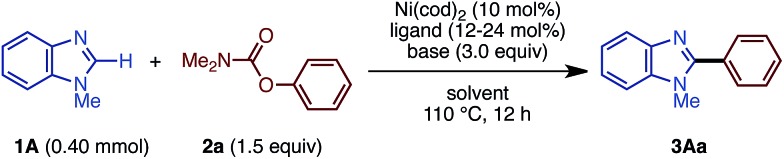				
Entry	Ligand	Base	Solvent	**3Aa** ^*b*^ (%)
1	dcype	Cs_2_CO_3_	1,4-Dioxane	0
2	dcype	K_3_PO_4_	*t*-AmylOH	83
3	dcype	K_3_PO_4_	*t*-BuOH	70
4	dcype	K_3_PO_4_	1,4-Dioxane	0
5	dcype	K_3_PO_4_	DMF	0
6	dcype	K_3_PO_4_	i-PrOH	0
7	dcype	Cs_2_CO_3_	*t*-AmylOH	44
8	dcype	LiO*t*-Bu	*t*-AmylOH	59
9	dcype	KO*t*-Bu	*t*-AmylOH	0
10	PCy_3_	K_3_PO_4_	*t*-AmylOH	0
11	IPr·HCl	K_3_PO_4_	*t*-AmylOH	0
12	dcypt	K_3_PO_4_	*t*-AmylOH	55
13	**L1**	K_3_PO_4_	*t*-AmylOH	53
14	**L2**	K_3_PO_4_	*t*-AmylOH	64
15^*c*^	dcype	K_3_PO_4_	*t*-AmylOH	82
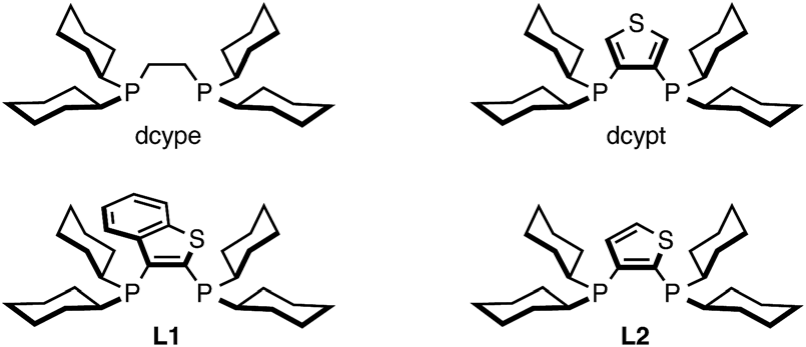				

^*a*^Unless otherwise noted, the reaction conditions were as follows: **1A** (0.40 mmol), **2a** (1.5 equiv.), Ni(cod)_2_ (10 mol%), ligand (bidentate: 12 mol%, monodentate: 24 mol%), base (3.0 equiv.), solvent (1.6 mL), 110 °C, and 12 h.

^*b*^GC yield.

^*c*^Ni(OTf)_2_ (10 mol%) was used.

**Scheme 1:**
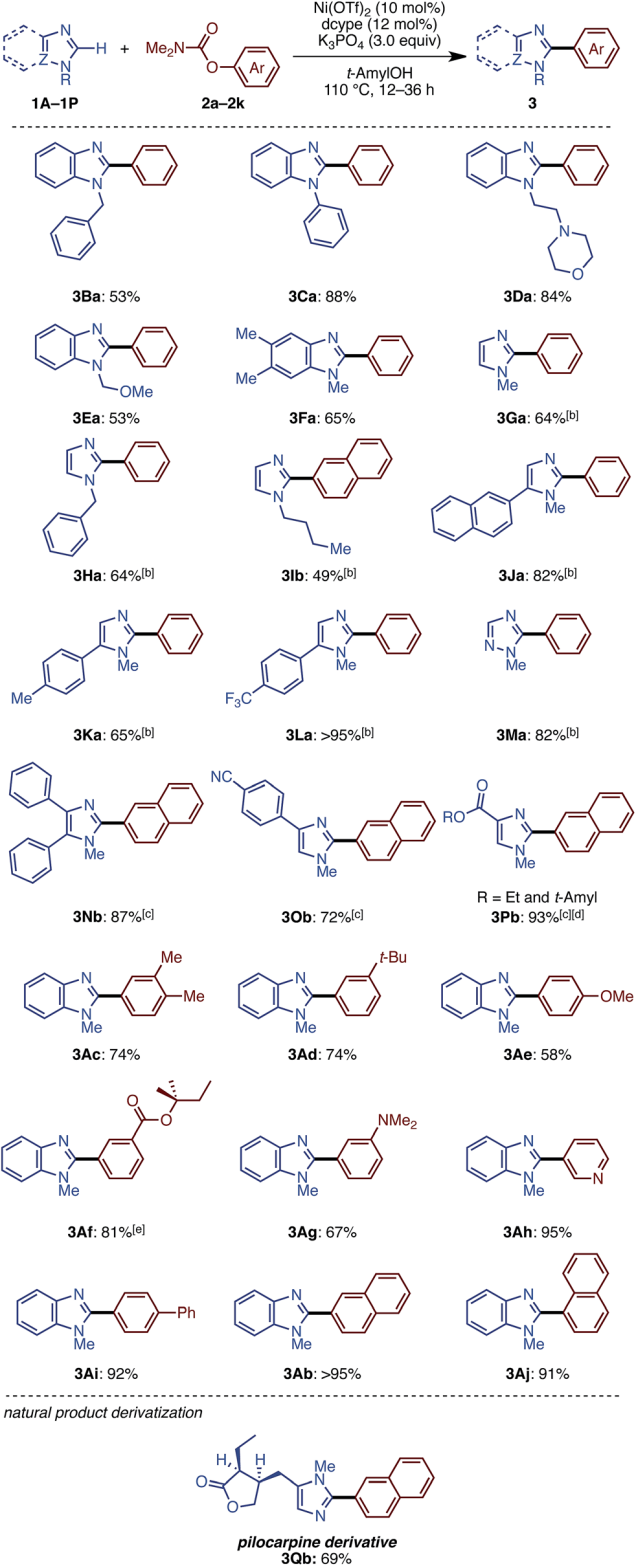
Substrate scope of imidazole C–H arylation with phenol derivatives. ^a^ Unless otherwise noted, the reaction conditions were as follows: **1** (0.40 mmol), **2** (1.5 equiv.), Ni(OTf)_2_ (10 mol%), dcype (12 mol%), K_3_PO_4_ (3.0 equiv.), *t*-amylOH (1.6 mL), 110 °C, and 12–36 h. ^b^ Ni(cod)_2_ (10 mol%) was used. ^c^ Ni(cod)_2_ (10 mol%) and dcypt (12 mol%) were used. ^d^ Starting from ethyl 4-imidazolecarboxylate **1P**. (R = Et: 33%; *t*-amyl: 60%). ^e^ Starting from methyl 3-((dimethylcarbamoyl)oxy)benzoate (**2f**).

We then examined the substrate scope of the Ni(OTf)_2_/dcype-catalyzed C–H arylation (C–H/C–O coupling) of imidazoles **1** with carbamates of phenol derivatives **2** (Scheme 1). Several *N*-substituted benzimidazoles such as *N*-benzyl (**1B**), phenyl (**1C**), morpholinoethyl (**1D**), and methoxymethyl benzimidazoles (**1E**) underwent C–H/C–O coupling to afford the corresponding products in moderate to excellent yields. In addition to benzimidazoles, imidazoles were also successfully coupled with phenol derivatives **2**, although it was necessary to use Ni(cod)_2_ as the catalyst precursor as well as a longer reaction time. In general, the coupling proceeded smoothly with the substrates having electron-withdrawing groups on the phenyl rings at the C5 position of the imidazole ring. For example, the reaction of trifluoromethyl-substituted 5-arylimidazole furnished triaryl **3La** in a superior yield (>95%) than that for the methyl-substituted triaryl **3Ka** (65%). Although the reason remains unclear at this stage, the dcypt ligand gave better results for the coupling of C4-substituted imidazoles (**1N**, **1O**, and **1P**). Notably, triazole **1M** also underwent C–H/C–O coupling with **2a** to afford 5-phenyl *N*-methyl-1,2,4-triazole (**3Ma**) in 82% yield. Regarding aryl electrophiles, a broad functional group tolerance was observed. An amino group or nitrogen heterocycle, which often behaves as a catalyst-deactivating group, did not inhibit the reaction; **3Da**, **3Ag**, and **3Ah** were obtained in good to excellent yields. Although transesterification took place with *t*-amylOH when the ethoxycarbonyl-substituted phenol electrophile **2f** was used, the product **3Af** was generated in good yield. Delightfully, we could directly functionalize pilocarpine (**1Q**), which is a drug for the treatment of a dry mouth,^18^ in 69% yield without any lactone opening or epimerization at the α-position of the carbonyl group. It should be emphasized that this coupling reaction proceeds with high regioselectivity at the C2 position of the imidazole rings.

### C–H alkenylation of imidazoles with enol derivatives

**Scheme 2:**
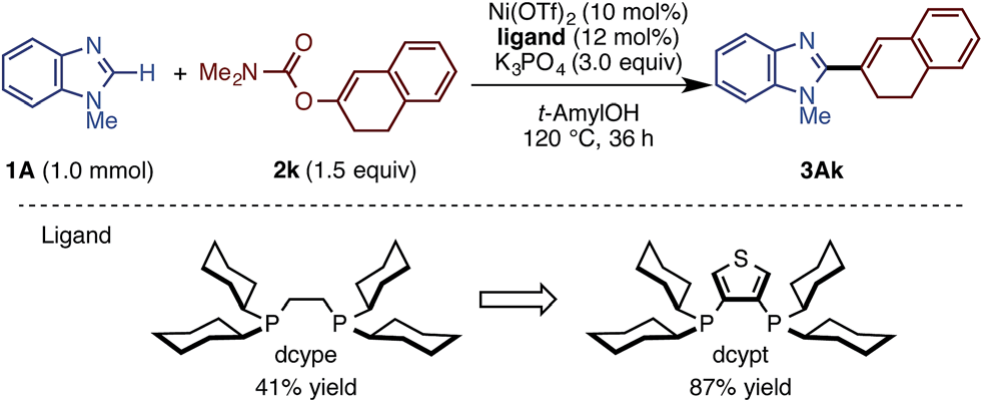
Dramatic effect of dcypt ligand in the C–H alkenylation of imidazoles.

Following our previous success of the Ni-catalyzed C–H alkenylation of oxazoles with enol derivatives,11b,^19^ we envisioned that alkenylation of imidazoles would be feasible under a Ni(OTf)_2_/diphosphine/K_3_PO_4_/*t*-amylOH system. The use of C–O alkenyl electrophiles for coupling reactions is advantageous because they can be easily prepared from the corresponding ketones and aldehydes. Motivated by the fact that the alkenyl group is a versatile platform in organic synthesis, we next embarked on the development of the C–H/C–O alkenylation of imidazoles (Scheme 2). Although the coupling reaction of **1A** and **2k** under Ni(OTf)_2_/dcype catalysis is feasible, it provided the alkenylated product **3Ak** in only 41% yield. While changes in base and solvent did not lead to the improvement of the reaction yield, the Ni(OTf)_2_/dcypt catalyst dramatically boosted the reaction efficiency, providing the coupling product **3Ak** in 87% yield.

**Scheme 3:**
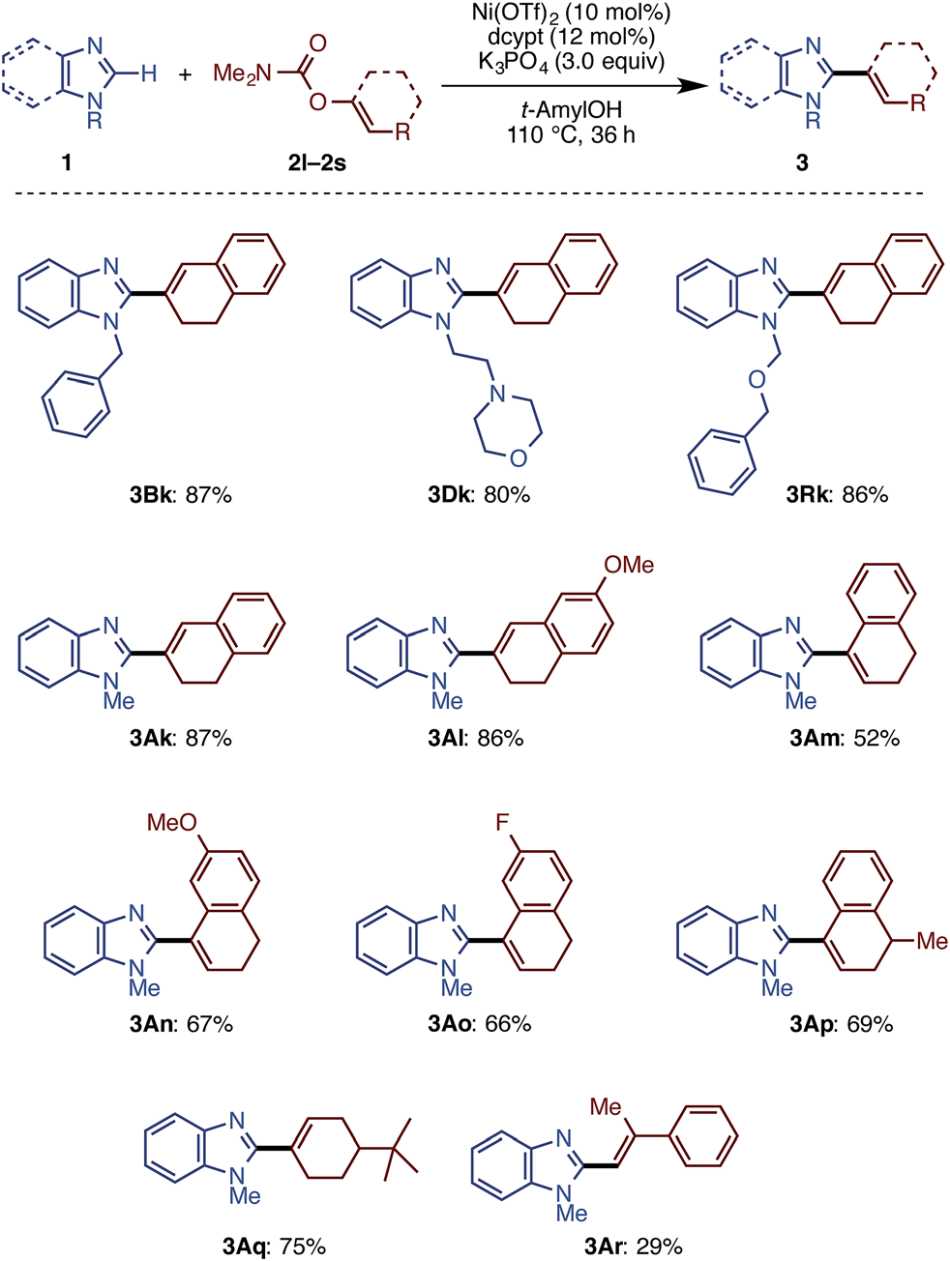
Scope of imidazole C–H alkenylation with Ni(OTf)_2_/dcypt. ^a^ Unless otherwise noted, the reaction conditions were as follows: **1** (1.0 mmol), **2** (1.5 equiv.), Ni(OTf)_2_ (10 mol%), dcypt (12 mol%), K_3_PO_4_ (3.0 equiv.), *t*-amylOH (4.0 mL), 120 °C, and 36 h.

It turns out that this newly discovered Ni(OTf)_2_/dcypt catalytic system can effectively facilitate the imidazole C–H alkenylation with broad scope (Scheme 3). As with the case of biaryl couplings, several *N*-alkylated benzimidazoles (such as **1D** and **1R**) could couple with enol carbamates. Both of the enol carbamates synthesized from α- and β-tetralones were reactive with the Ni(OTf)_2_/dcypt catalyst to afford the coupling products **3Al–3AP**. Cyclohexenyl benzimidazoles could be synthesized from cyclohexanone derived electrophiles. The aldehyde-derived enol derivative **2r** also coupled with **1A**, but its rather fast decomposition under the reaction conditions turned out to be somewhat problematic. It is known that C–OMe14a,e and C–F bonds^20^ can be cleaved with nickel complexes, but these groups were completely tolerated in the present Ni-catalyzed coupling reaction.

### C–H arylation of imidazoles with chloroarenes

**Scheme 4:**
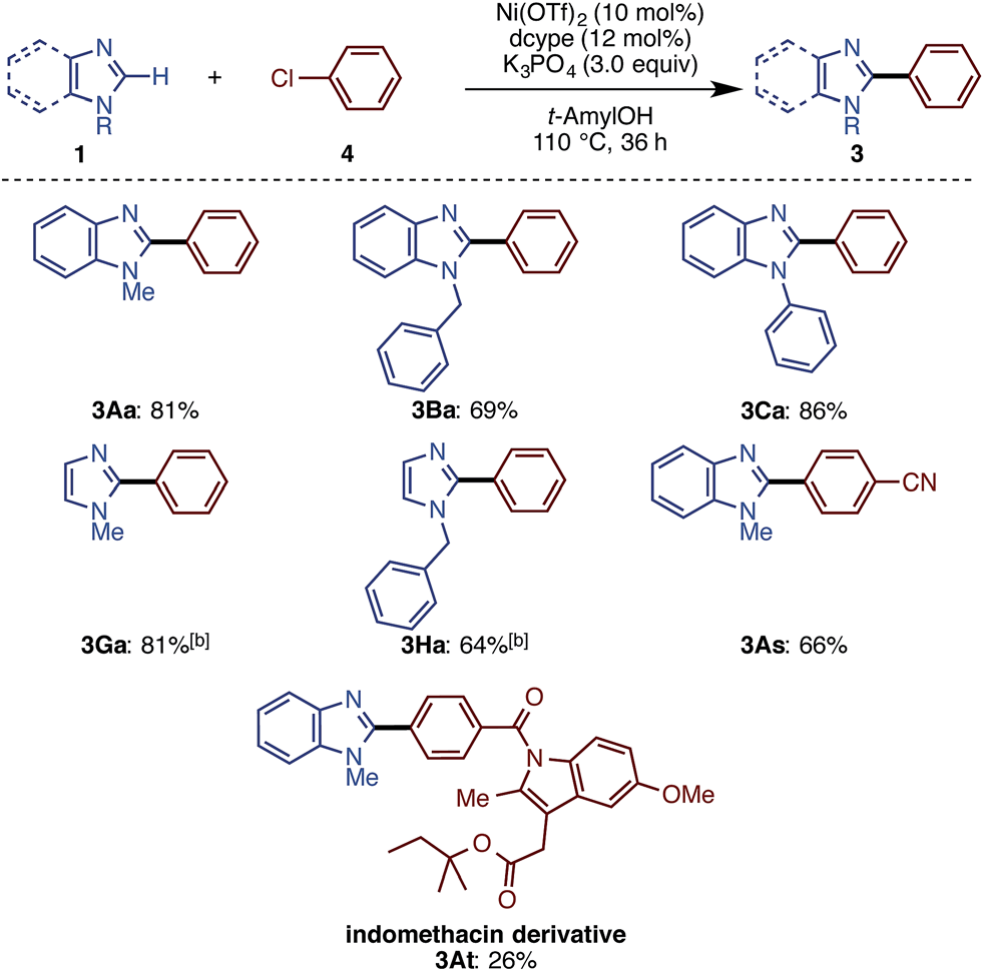
C–H arylation of imidazoles with chloroarenes with Ni(OTf)_2_/dcype. ^a^ Unless otherwise noted, the reaction conditions were as follows: 1 (0.40 mmol), **4** (1.5 equiv.), Ni(OTf)_2_ (10 mol%), dcype (12 mol%), K_3_PO_4_ (3.0 equiv.), *t*-amylOH (1.6 mL), 110 °C, and 36 h. ^b^ Ni(cod)_2_ (10 mol%) was used.

While we mainly focused on the use of C–O electrophiles in this study, it was found that the newly developed Ni(OTf)_2_/dcype/K_3_PO_4_/*t*-amylOH system is also effective for imidazole arylation with chloroarenes. As shown in Scheme 4, a range of imidazoles and benzimidazoles cross-coupled with chlorobenzene derivatives under the standard conditions. *N*-Methyl, -phenyl, and -benzyl benzimidazoles underwent C–H arylation with chlorobenzene (**4a**) to deliver phenylated imidazoles in good yield. *N*-Methyl and -benzyl imidazoles also reacted as well. Nitrile-substituted aryl chloride **4s** furnished the corresponding product **3As** in good yield. Although the reaction yield was low (26%), we could apply the indomethacin derivative **4t** to the reaction to give **3At**, but with significant amounts of a homodimerization by-product. Very interestingly, the reactions of aryl iodides and bromides in turn resulted in poor or zero yields of product.

### C–H arylation and alkenylation of other 1,3-azoles

**Scheme 5:**
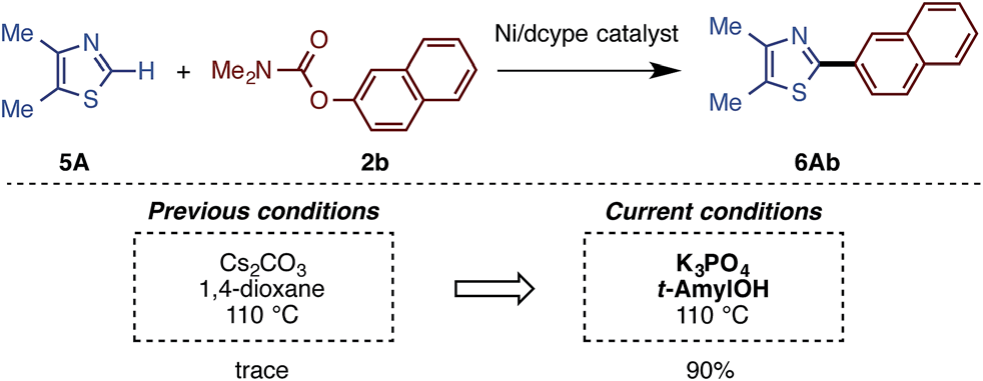
Comparison of the previous and current conditions for the reaction of thiazole **5A**.

The present catalytic protocols were found to be applicable not only for imidazoles, but also for thiazoles and oxazoles. While our previous catalyst, employing Ni(cod)_2_/dcype/Cs_2_CO_3_ in 1,4-dioxane, was effective for oxazoles and thiazoles (in particular for benzo-fused substrates),^11^ coupling was not efficient for relatively electron-rich azoles. For example, the reaction of 4,5-dimethylthiazole (**5A**) with naphthyl carbamate **2b** under the previous conditions furnished no coupling product. Thus, we applied our new protocol with K_3_PO_4_ and *t*-amylOH to the reaction of the previously unreactive azole **5A**. Gratifyingly, **5A** and **2b** cross-coupled very smoothly under the present conditions to furnish **6Ab** in 90% yield (Scheme 5).

**Scheme 6:**
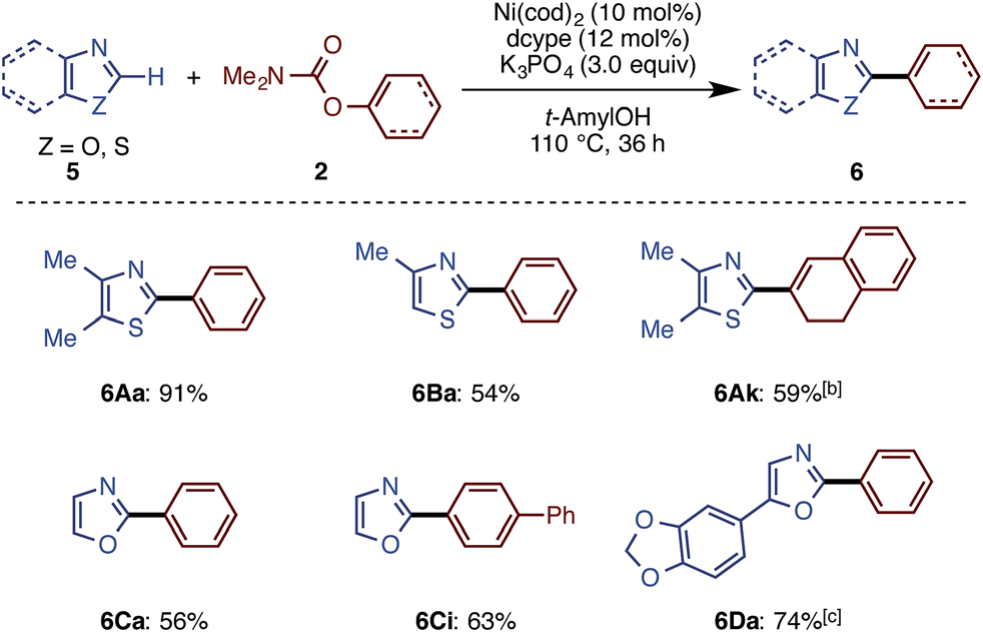
C–H arylation and alkenylation of oxazoles and thiazoles with Ni(OTf)_2_/dcype/K_3_PO_4_ in *t*-amylOH. ^a^ Unless otherwise noted, the reaction conditions were as follows: **5** (0.40 mmol), **2** (1.5 equiv.), Ni(OTf)_2_ (10 mol%), dcypt (12 mol%), K_3_PO_4_ (3.0 equiv.), *t*-amylOH (1.6 mL), 110 °C, and 36 h. ^b^ Ni(OTf)_2_ (10 mol%) and dcypt (12 mol%) were used. ^c^ Ni(OTf)_2_ (10 mol%) and dcypt (12 mol%) were used.

Depicted in Scheme 6 are the results of the Ni-catalyzed reactions of the other 1,3-azoles. In addition to thiazoles, oxazoles were also found to be good substrates, generating the corresponding coupling products in good yields. Although previous alkenylation reactions with C–O electrophiles were limited to the reaction of oxazoles, the present Ni-catalyzed reaction in *t*-amylOH was also applicable to thiazoles.

### Plausible mechanism

**Fig. 3:**
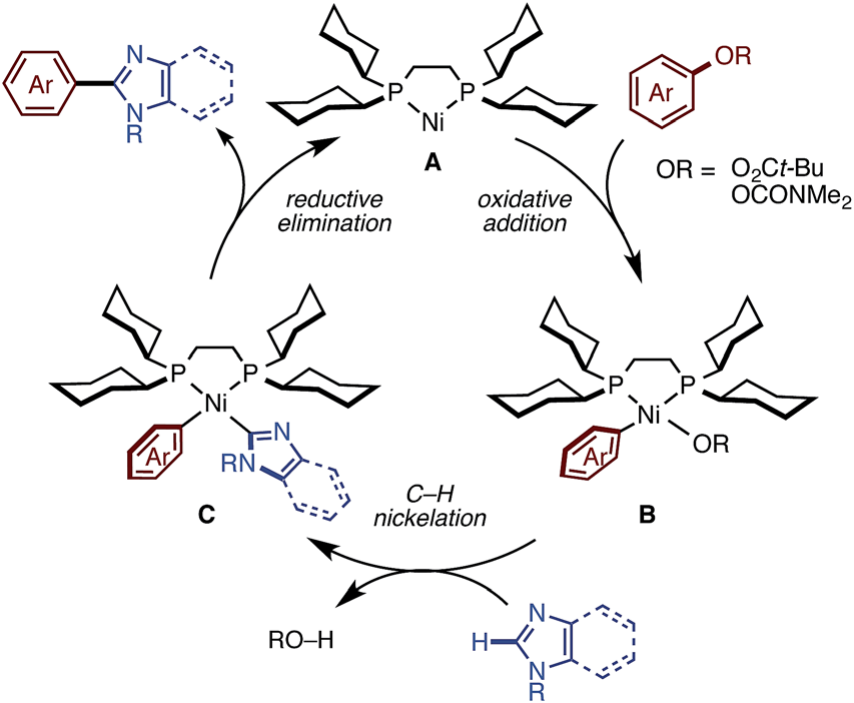
A plausible catalytic cycle.

We succeeded in developing new catalytic systems to significantly expand the C–H/C–O coupling of 1,3-azoles with phenol/enol-based electrophiles, but we feel that the basic catalytic cycle with Ni(OTf)_2_/diphosphine/K_3_PO_4_/*t*-amylOH should be similar to those operating under the previous conditions. First, Ni(OTf)_2_ should be reduced to a nickel(0) species by the action of diphosphine (dcype or dcypt) and/or an imidazole substrate to initiate a Ni(0)/Ni(ii) redox cycle as shown in Fig. 3. Oxidative addition of the C–O or C–Cl bond of the electrophile to Ni(0) **A** affords intermediate **B**. Then, base-promoted C–H nickelation of imidazoles, followed by reductive elimination would furnish the coupling products with regeneration of the active Ni(0) species. Previously, we successfully isolated and characterized intermediate **B** by using naphthalen-2-yl-pivalate as a C–O electrophile,11*c* which supports our hypothesized catalytic cycle. The remaining question is how the new conditions (particularly the activation modes of K_3_PO_4_ and *t*-amylOH) allow imidazoles to participate in this catalytic cycle. We are currently focusing on uncovering these phenomena experimentally and theoretically.

## Conclusions

In summary, we have established a general protocol for the nickel-catalyzed C–H coupling of imidazoles. The newly discovered conditions, employing a catalytic amount of Ni(OTf)_2_/dcype or Ni(OTf)_2_/dcypt and K_3_PO_4_ in *t*-amylOH, enable direct C–C bond-forming reactions of imidazoles including C–H/C–O arylations and alkenylations. The C–H arylation of imidazoles with chloroarenes as well as that of thiazoles and oxazoles with phenol/enol derivatives can also be achieved with this catalytic system. The key to the success of the new nickel-catalyzed system is the choice of a tertiary alcohol as solvent, as neither aprotic solvents nor secondary alcohols were effective. We believe that the present method provides significant opportunities to synthesize and derivatize valuable functionalized imidazoles.

## Supplementary Material

Supplementary information
