# Supplementary material for: C–H arylation and alkenylation of imidazoles by nickel catalysis: solvent-accelerated imidazole C–H activation
Source: Chem Sci. 2015 Sep 8;6(12):6792–8. doi: 10.1039/c5sc02942b (PMC5951105; doi:10.1039/c5sc02942b)
Supplement: Supplementary file 1 [file SC-006-C5SC02942B-s001.pdf]

Electronic Supplementary Material (ESI) for Chemical Science.
This journal is © The Royal Society of Chemistry 2015

Supplementary Information

C–H Arylation and Alkenylation of Imidazoles by Nickel Catalysis: Solvent-accelerated Imidazole C–H Activation

Kei Muto,[a] Taito Hatakeyama,[b] Junichiro Yamaguchi,[a*] and Kenichiro Itami[a,c*]

[a] *Institute of Transformative Bio-Molecules (WPI-ITbM) and Graduate School of Science, Nagoya University, Chikusa, Nagoya 464-8602, Japan*

[b] *Central Research Laboratory Technology and Development Division, Kanto Chemicals Co. Inc., Saitama 340-0003, Japan*

[c] *JST, ERATO, Itami Molecular Nanocarbon Project, Nagoya University, Chikusa, Nagoya 464-8602, Japan*

E-mail: junichiro@chem.nagoya-u.ac.jp, itami@chem.nagoya-u.ac.jp

Table of Contents

1. General S2–S3
2. Substrate Structure of Imidazoles, Carbamates, Chloroarenes, Thiazoles, and Oxazoles S4–S5
3. Preparation of Starting Materials S6–S12
4. Ni-Catalyzed C–H Arylation of Imidazoles with Phenol Derivatives (C–H/C–O Coupling) and Chloroarenes (C–H/C–Cl Coupling) S13–S23
5. Ni-Catalyzed C–H Alkenylation of Imidazoles with Enol Derivatives S24–S28
6. Ni-Catalyzed C–H Coupling of Thiazoles and Oxazoles S28–S31
7. ^{1}H NMR and ^{13}C NMR Spectra S32–S153

1. General

Unless otherwise noted, all materials including dry solvents were obtained from commercial suppliers and used as received. Ni(cod)$_2$ and K_3PO_4 were obtained from Wako Chemicals. 1,2-Bis(dicyclohexylphosphino)ethane (dcype) was obtained from Kanto Chemical. Dry *t*-AmylOH was purchased from Sigma-Aldrich and used as received. 1-Benzyl-1*H*-benzo[*d*]imidazole (**1B**)[1], 1-phenyl-1*H*-benzo[*d*]imidazole (**1C**)[2], 4-(2-(1*H*-benzo[*d*]imidazol-1-yl)ethyl)morpholine (**1D**)[3], 1-(methoxymethyl)-1*H*-benzo[*d*]imidazole (**1E**)[4], 1,5,6-trimethyl-1*H*-benzo[*d*]imidazole (**1F**)[5], 1-benzyl-1*H*-imidazole (**1H**)[6], 1-methyl-4,5-diphenyl-1*H*-imidazole (**1N**)[7], 4-(1-methyl-1*H*-imidazol-4-yl)benzonitrile (**1O**)[8], 1-((benzyloxy)methyl)-1*H*-benzo[*d*]imidazole (**1Q**)[9], phenyl dimethylcarbamate (**2a**)[10], naphthalen-2-yl dimethylcarbamate (**2b**)[10], naphthalen-1-yl dimethylcarbamate (**2j**)[10], 4-methoxyphenyl dimethylcarbamate (**2e**)[11], methyl 3-((dimethylcarbamoyl)oxy)benzoate (**2f**)[11], [1,1'-biphenyl]-4-yl dimethylcarbamate (**2l**)[10], 3,4-dihydronaphthalen-2-yl dimethylcarbamate (**2k**)[12], 3,4-dihydronaphthalen-1-yl dimethylcarbamate (**2m**)[13], 5-(benzo[*d*][1,3]dioxol-5-yl)-2-phenyloxazole (**5D**)[14], and 3,4-bis(dicyclohexylphosphino)thiophene (dcypt)[15] were synthesized according to procedures reported in the literature. Unless otherwise noted, all reactions were performed with dry solvents under an atmosphere of argon in flame-dried glassware using standard vacuum-line techniques. All C–H coupling reactions were performed in 20-mL glass vessel tubes equipped with J. Young® O-ring tap and heated in an 8-well reaction block (heater + magnetic stirrer) unless otherwise noted. All work-up and purification procedures were carried out with reagent-grade solvents in air.

Analytical thin-layer chromatography (TLC) was performed using E. Merck silica gel 60 F_{254} precoated plates (0.25 mm). The developed chromatogram was analyzed by UV lamp (254 nm). Flash column chromatography was performed with E. Merck silica gel 60 (230–400 mesh) or Biotage Isolera® equipped with Biotage SNAP Cartridge KP-Sil columns using hexane/EtOAc as an eluent.

---

1 O. Kose and S. Saito, *Org. Biomol. Chem.*, 2010, **8**, 896.
2 L.-H. Zou, J. Reball, J. Mottweiler and C. Bolm, *Chem. Commun.*, 2012, **48**, 11307.
3 İ. Özdemir, N. Şahin, Y. Gök, S. Demir and B. Çetinkaya, *J. Mol. Catal. A: Chem.*, 2005, **234**, 181.
4 A. Rivera, M. Maldonado, J. Ríos-Motta, M. A. Navarro and D. González-Salas, *Tetrahedron Lett*, 2009, **51**, 102.
5 E. A. Collins, P. Garcia-Losada, C. Hamdouchi, P. A. Hipskind, J. Lu and T. Takakuwa, Patent WO 2006-US12072.
6 W. Tang, W.-G. Yuan, B. Zhao, H.-L. Zhang, F. Xiong, L.-H. Jing and D.-B. Qin, *J. Organomet. Chem.*, 2013, **743**, 147.
7 F. J. LaRonde and M. A. Brook, *Inorg. Chim. Acta*, 1999, **296**, 208.
8 L. S. Lin, M. D. Chioda, P. Liu and R. P. Nargund, Patent WO 2009-US46241.
9 P. W. Glunz, N. Wurtz and X. Cheng, Patent WO 2005-US44113.
10 X. Yang, Y. Sun, Z. Chen and Y. Rao, *Adv. Synth. Catal.*, 2014, **356**, 1625.
11 X. Zhao, C. S. Yeung and V. M. Dong, *J. Am. Chem. Soc.*, 2010, **132**, 5832.
12 R. A. Olofson and J. Cuomo, *Tetrahedron Lett.,* 1980, **21**, 819.
13 T.-J. Gong, W. Su, Z.-J. Liu, W.-M. Cheng, B. Xiao and Y. Fu, *Org. Lett.,* 2014, **16**, 330.
14 A. C. Giddens, H. I. M. Boshoff, S. G. Franzblau, C. E. Barry and B. R. Copp,. *Tetrahedron Lett.*, 2005, **46**, 7355.
15 R. Takise, K. Muto, J. Yamaguchi and K. Itami, *Angew. Chem. Int. Ed.*, 2014, **53**, 6791.

Preparative thin-layer chromatography (PTLC) was performed using Wakogel B5-F silica coated plates (0.75 mm) prepared in our laboratory. High-resolution mass spectra (HRMS) were obtained from a JMS-T100TD instrument (DART) and Thermo Fisher Scientific Exactive (ESI). Nuclear magnetic resonance (NMR) spectra were recorded on a JEOL JNM-ECA-600 (^{1}H 600 MHz, ^{13}C 150 MHz) spectrometer and a JEOL JNM-ECA-400 (^{1}H 400 MHz, ^{13}C 100 MHz) spectrometer. Chemical shifts for ^{1}H NMR are expressed in parts per million (ppm) relative to tetramethylsilane (δ 0.00 ppm). Chemical shifts for ^{13}C NMR are expressed in ppm relative to $CDCl_3$ (δ 77.0 ppm). Data are reported as follows: chemical shift, multiplicity (s = singlet, d = doublet, dd = doublet of doublets, t = triplet, q = quartet, m = multiplet, br = broad signal), coupling constant (Hz), and integration.

2. Substrate Structure of Imidazoles, Carbamates, Chloroarenes, Thiazoles, and Oxazoles

Imidazoles **1**

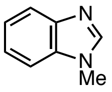 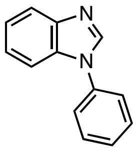 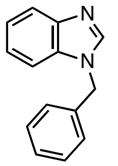 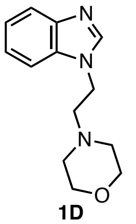 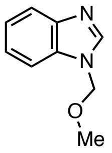 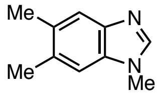

1A **1B** **1C** **1D** **1E** **1F**

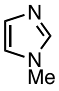 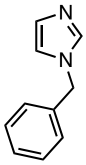 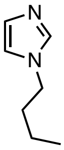 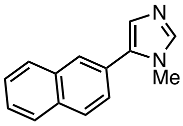 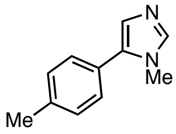 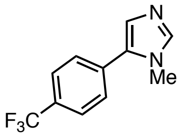

1G **1H** **1I** **1J** **1K** **1L**

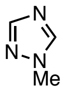 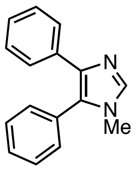 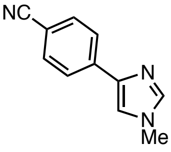 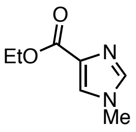 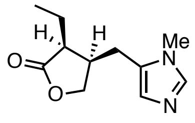 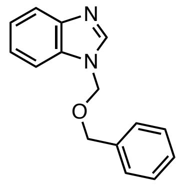

1M **1N** **1O** **1P** **1Q** **1R**

Carbamates **2**

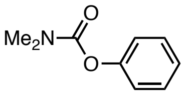
2a

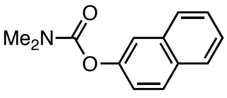
2b

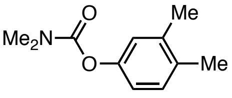
2c

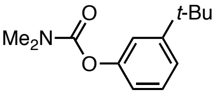
2d

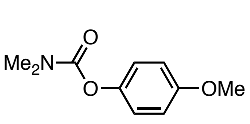
2e

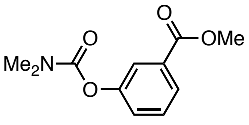
2f

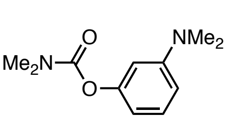
2g

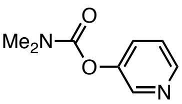
2h

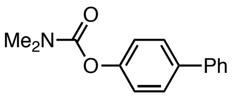
2i

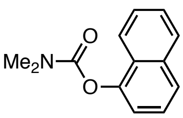
2j

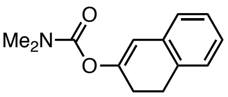
2k

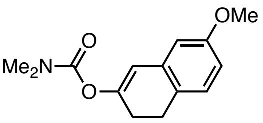
2l

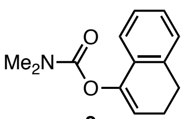
2m

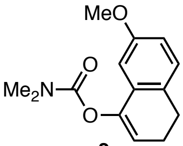
2n

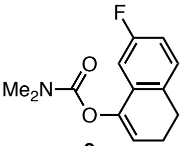
2o

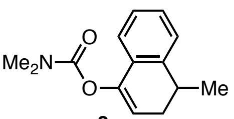
2p

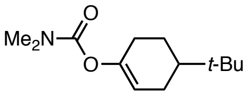
2q

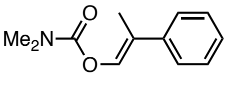
2r

Chloroarenes **4**

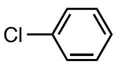
4a

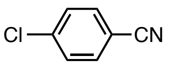
4s

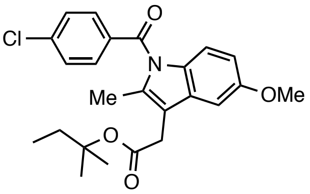
4t

Thiazoles and Oxazoles **5**

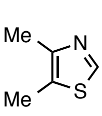
5A

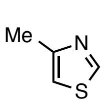
5B

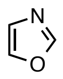
5C

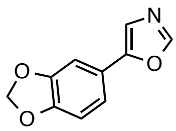
5D

3. Preparation of Starting Materials

Ethyl 1-methyl-1*H*-imidazole-4-carboxylate (1P)

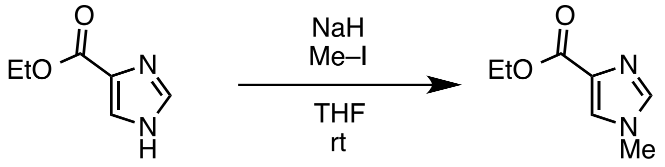

1P: 80%

To a solution of ethyl 1*H*-imidazole-4-carboxylate (700 mg, 5.0 mmol, 1.0 equiv) in THF (15 mL) was added NaH (60% dispersion in paraffin liquid: 300 mg, 7.5 mmol, 1.5 equiv) at 0 °C. After stirring the mixture for 30 min, methyl iodide (781 mg, 5.5 mmol, 1.1 equiv) was added at 0 °C and the solution was stirred overnight at room temperature. The mixture was quenched by the addition of water, then extracted several times with EtOAc, dried over Na_2SO_4, and filtrated. The solution was concentrated *in vacuo*. The crude mixture was purified by Isolera® (hexane/EtOAc = 1:1 to EtOAc) to afford **1P** as a yellow solid (615 mg, 80% yield). ^{1}H NMR (400 MHz, $CDCl_3$): δ 7.58 (s, 1H), 7.45 (s, 1H), 4.36 (q, J = 7.2 Hz, 2H), 3.74 (s, 3H), 1.39 (t, J = 7.2 Hz, 3H); ^{13}C NMR (100 MHz, $CDCl_3$): δ 162.5, 138.3, 133.8, 125.9, 60.1, 33.5, 14.1; HRMS (ESI) *m/z* calcd for $C_7H_{10}N_2O_2Na$ $[M+Na]^+$: 177.0634, found 177.0633.

3,4-Dimethylphenyl dimethylcarbamate (2c)

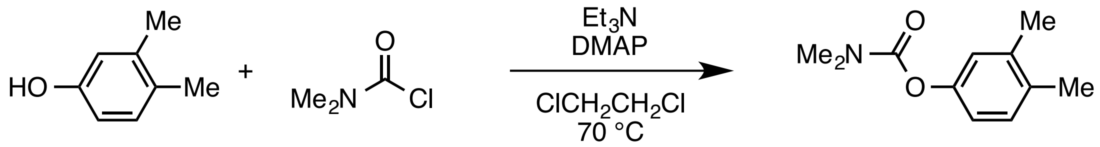

2c: quant

To a solution of 3,4-dimethylphenol (611 mg, 5.0 mmol, 1.0 equiv) and *N*,*N*-dimethyl-4-aminopyridine (DMAP: 6.1 mg, 0.050 mmol, 1 mol%) in dichloroethane (4 mL) were added Et_3N (843 μL, 6.0 mmol, 1.2 equiv) and *N*,*N*-dimethylcarbamoyl chloride (505 μL, 5.5 mmol, 1.1 equiv). This mixture was stirred overnight at 70 °C. The reaction mixture was quenched by the addition of saturated $NaHCO_3$ aq, and then the mixture was extracted four times with CH_2Cl_2. The combined organic layer was dried over Na_2SO_4, and concentrated *in vacuo*. The residue was purified by flash silica-gel column chromatography (hexane/EtOAc = 10:1) to afford **2c** as a white solid (980 mg, quant). ^{1}H NMR (400 MHz, $CDCl_3$): δ 7.09 (d, J = 8.0 Hz, 1H), 6.89 (s, 1H), 6.83 (d, J = 8.0 Hz, 1H), 3.08 (s, 3H), 2.99 (s, 3H), 2.23 (s, 3H), 2.22 (s, 3H); ^{13}C NMR (100 MHz, $CDCl_3$): δ 155.3, 149.4, 137.5, 133.4, 130.1, 122.7, 118.8, 36.6, 36.4, 19.8, 19.1; HRMS (ESI) *m/z* calcd for $C_{11}H_{15}NO_2Na$ $[M+Na]^+$: 216.0995, found 216.0991.

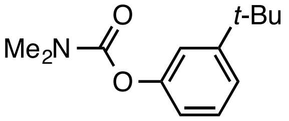

2d: 97%

3-(*tert*-Butyl)phenyl dimethylcarbamate (2d): Purification by flash silica-gel column chromatography (hexane/EtOAc = 10:1) afforded **2d** as a white solid (751 mg, 97% yield). ^{1}H NMR (400 MHz, $CDCl_3$): δ 7.29 (dd, *J* = 8.4, 7.6 Hz, 1H), 7.21 (d, *J* = 7.6 Hz, 1H), 7.10 (s, 1H), 6.93 (d, *J* = 8.4 Hz, 1H), 3.10 (s, 3H), 3.01 (s, 3H), 1.32 (s, 9H); ^{13}C NMR (100 MHz, $CDCl_3$): δ 155.0, 152.7, 151.3, 128.6, 122.2, 118.74, 118.71, 36.6, 36.4, 34.7, 31.2; HRMS (DART) *m/z* calcd for $C_{13}H_{20}NO_2$ $[M+H]^+$: 222.1494, found 222.1490.

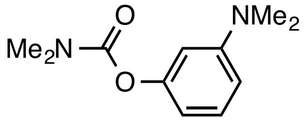

2g: 87%

3-(Dimethylamino)phenyl dimethylcarbamate (2g): Purification by flash silica-gel column chromatography (hexane/EtOAc = 4:1) afforded **2g** as a brown liquid (900 mg, 87% yield). ^{1}H NMR (400 MHz, $CDCl_3$): δ 7.18 (t, *J* = 8.0 Hz, 1H), 6.55 (d, *J* = 8.0 Hz, 1H), 6.45 (d, *J* = 8.0 Hz, 1H), 6.44 (s, 1H), 3.09 (s, 3H), 3.00 (s, 3H), 2.93 (s, 6H); ^{13}C NMR (100 MHz, $CDCl_3$): δ 155.1, 152.5, 151.6, 129.4, 109.6, 109.5, 105.9, 40.5, 36.6, 36.4; HRMS (ESI) *m/z* calcd for $C_{11}H_{16}N_2O_2Na$ $[M+Na]^+$: 231.1104, found 231.1100.

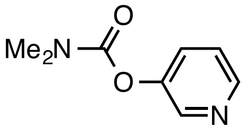

2h: 87%

Pyridin-3-yl dimethylcarbamate (2h): Purification by flash silica-gel column chromatography (hexane/EtOAc = 1:1 to EtOAc) afforded **2h** as a yellow liquid (721 mg, 87% yield). ^{1}H NMR (400 MHz, $CDCl_3$): δ 8.46–8.42 (m, 2H), 7.52 (dd, *J* = 8.4, 4.0 Hz, 1H), 7.30 (dd, *J* = 8.4, 4.0 Hz, 1H), 3.12 (s, 3H), 3.03 (s, 3H); ^{13}C NMR (100 MHz, $CDCl_3$): δ 154.0, 148.1, 146.2, 143.5, 129.3, 123.6, 36.7, 36.4; HRMS (ESI) *m/z* calcd for $C_8H_{10}N_2O_2Na$ $[M+Na]^+$: 189.0634, found 189.0630.

7-Methoxy-3,4-dihydronaphthalen-2-yl dimethylcarbamate (2l):

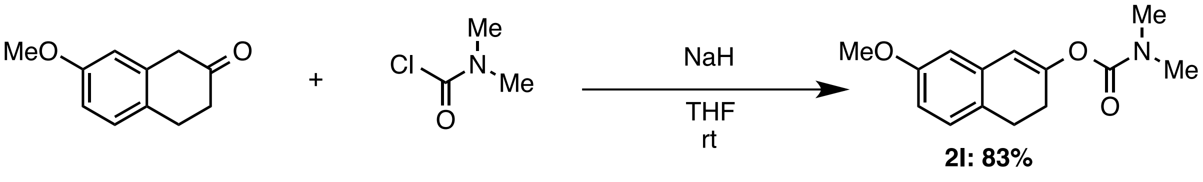

2l: 83%

NaH (55% dispersion in paraffin liquid: 1.15 g, 27 mmol, 1.8 equiv) was placed in a 300-mL flask under a stream of argon, and THF (24 mL) was added to the flask. To this suspension, 7-methoxy-3,4-dihydronaphthalen-2(1*H*)-one (2.82 g, 16 mmol, 1.0 equiv) in THF (8 mL) was added dropwise. The resulting mixture was stirred at room temperature for 5 min. A solution of *N*,*N*-dimethylcarbamoyl chloride (2.21 mL, 24 mmol, 1.5 equiv) in THF (4 mL) was added, and then the mixture was stirred for an additional 30 min. The reaction was quenched by the addition of water. The mixture was extracted three times with *tert*-butyl methyl ether, and the combined organic layer was washed with water and brine, dried over Na_2SO_4, and then filtered. The filtrate was concentrated *in vacuo*. The crude residue was purified by flash silica-gel column chromatography (hexane/EtOAc = 3:1) to afford **2l** as a pale yellow oil (3.26 g, 83% yield). ^{1}H NMR (400 MHz, $CDCl_3$): δ 7.01 (d, *J* = 7.6 Hz, 1H), 6.63 (dd, *J* = 7.6, 2.4 Hz, 1H), 6.57 (d, *J* = 2.4 Hz, 1H), 6.17 (s, 1H), 3.77 (s, 3H), 3.01 (s, 3H), 2.97 (s, 3H), 2.92 (t, *J* = 8.0 Hz, 2H), 2.51 (t, *J* = 8.0 Hz, 2H); ^{13}C NMR (100 MHz, $CDCl_3$): δ 158.3, 154.2, 152.1, 134.7, 127.7, 125.3, 113.9, 111.9, 111.2, 55.2, 36.44, 36.35, 27.7, 27.0; HRMS (DART) *m/z* calcd for $C_{14}H_{18}NO_3$ $[M+H]^+$: 248.1287, found 248.1287.

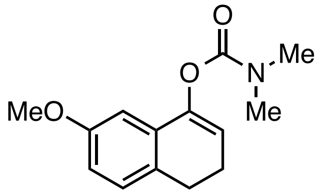

2n

7-Methoxy-3,4-dihydronaphthalen-1-yl dimethylcarbamate (2n): Purification by flash silica-gel column chromatography (hexane/EtOAc = 3:1) afforded **2n** as a pale yellow oil (1.45 g, 39% yield). ^{1}H NMR (400 MHz, $CDCl_3$): δ 7.05 (d, *J* = 8.0 Hz, 1H), 6.72–6.68 (m, 2H), 5.73 (t, *J* = 4.4 Hz, 1H), 3.78 (s, 3H), 3.13 (s, 3H) 2.99 (s, 3H), 2.79 (t, *J* = 8.0 Hz, 2H), 2.41 (dt, *J* = 8.0, 4.4 Hz, 2H); ^{13}C NMR (100 MHz, $CDCl_3$): δ 158.1, 154.5, 145.6, 132.2, 128.5, 128.0, 115.6, 111.6, 107.6, 55.1, 36.6, 36.2, 26.5, 22.3; HRMS (DART) *m/z* calcd for $C_{14}H_{18}NO_3$ $[M+H]^+$: 248.1287, found 248.1282.

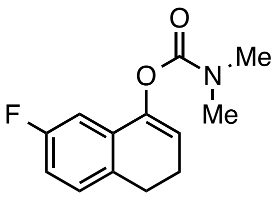

2o

7-Fluoro-3,4-dihydronaphthalen-1-yl dimethylcarbamate (2o): Purification by flash silica-gel column chromatography (hexane/Et_2O = 1:1) afforded **2o** as a yellow oil (1.02 g, 71% yield). ^{1}H NMR (400 MHz, $CDCl_3$): δ 7.10–7.04 (m, 1H), 6.88–6.80 (m, 2H), 5.77 (t, *J* = 4.4 Hz, 1H), 3.12 (s, 3H),

2.99 (s, 3H), 2.81 (t, J = 7.6 Hz, 2H), 2.43 (dt, J = 7.6, 4.4 Hz, 2H); ^{13}C NMR (100 MHz, $CDCl_3$): δ 161.7 (d, $J_{C–F}$ = 241.2 Hz), 154.4, 145.1 (d, $J_{C–F}$ = 1.9 Hz), 133.0 (d, $J_{C–F}$ = 8.6 Hz), 131.7 (d, $J_{C–F}$ = 2.9 Hz), 128.5 (d, $J_{C–F}$ = 7.6 Hz), 116.3, 113.8 (d, $J_{C–F}$ = 20.9 Hz), 107.9 (d, $J_{C–F}$ = 23.9 Hz), 36.6, 36.3, 26.6, 22.1; HRMS (DART) *m/z* calcd for $C_{13}H_{15}FNO_2$ $[M+H]^+$: 236.1087, found 236.1087.

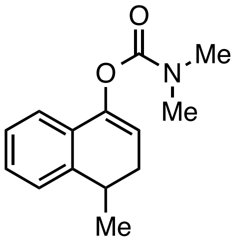

2p

4-Methyl-3,4-dihydronaphthalen-1-yl dimethylcarbamate (2p): Purification by flash silica-gel column chromatography (hexane/EtOAc = 4:1) afforded **2p** as a white solid (2.34 g, 65% yield). ^{1}H NMR (400 MHz, $CDCl_3$): δ 7.26–7.10 (m, 4H), 5.64 (t, J = 4.4 Hz, 1H), 3.13 (s, 3H), 3.07–2.93 (m, 4H), 2.60 (ddd, J = 16.8, 6.4, 4.4 Hz, 1H), 2.23 (ddd, J = 16.8, 6.4, 4.4 Hz, 1H), 1.31 (d, J = 6.4 Hz, 3H); ^{13}C NMR (100 MHz, $CDCl_3$): δ 154.9, 145.4, 141.4, 130.4, 127.9, 126.2, 126.1, 120.7, 113.6, 36.7, 36.4, 31.9, 29.9, 20.1; HRMS (DART) *m/z* calcd for $C_{14}H_{18}NO_2$ $[M+H]^+$: 232.1338, found 232.1339.

4-(*tert*-Butyl)cyclohex-1-en-1-yl dimethylcarbamate (2q)

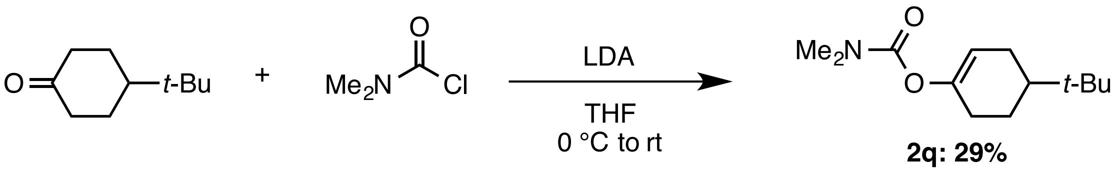

To a solution of 4-(*tert*-butyl)cyclohexanone (462.8 mg, 3.0 mmol, 1.0 equiv) in THF (4.5 mL) was slowly added lithium diisopropylamide [LDA: prepared from diisopropylamine (333.9 mg, 3.3 mmol, 1.1 equiv) and 1.6 M of *n*-BuLi in hexane (2.06 mL, 3.3 mmol, 1.1 equiv) in 4.5 mL of THF] at 0 °C. After stirring the solution for 1 h, *N*,*N*-dimethylcarbamoyl chloride (413.6 mL, 4.5 mmol, 1.5 equiv) was added at 0 °C and the mixture was allowed to warm up to room temperature. The solution was stirred overnight, and then $NaHCO_3$ aq was added to quench the reaction. The mixture was extracted three times with EtOAc, washed with brine, dried over Na_2SO_4, and then filtered. The mixture was concentrated *in vacuo*. The crude mixture was purified by flash silica-gel column chromatography (hexane/EtOAc = 50:1 to 10:1) to afford **2q** as a colorless liquid (193 mg, 29% yield). ^{1}H NMR (400 MHz, $CDCl_3$): δ 5.35 (t, J = 2.8 Hz, 1H), 2.95 (s, 3H), 2.93 (s, 3H), 2.38–2.21 (m, 1H), 2.17–2.05 (m, 2H), 1.96–1.80 (m, 2H), 1.42–1.30 (m, 2H), 0.89 (s, 9H); ^{13}C NMR (100 MHz, $CDCl_3$): δ 155.0, 148.7,

113.5, 43.4, 36.4, 36.2, 32.1, 28.2, 27.3, 25.0, 24.0; HRMS (DART) *m/z* calcd for $C_{13}H_{24}NO_2$ $[M+H]^+$: 226.1807, found 226.1810.

(*E*)-2-Phenylprop-1-en-1-yl dimethylcarbamate (2r)

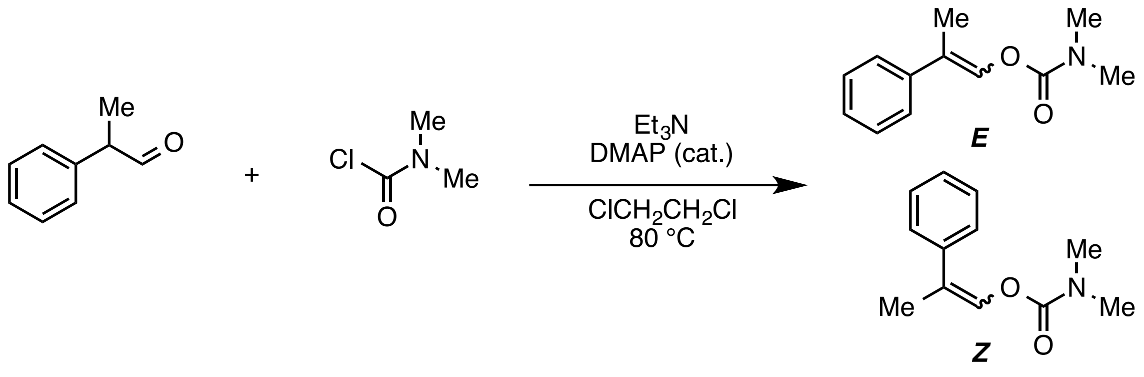

2r: 85% (*E*/*Z* = 5/3)

To a solution of 2-phenylpropionaldehyde (2.7 mL, 20.2 mmol, 1.0 equiv) in dichloroethane (40 mL) were added Et_3N (4.8 mL, 34.4 mmol, 1.7 equiv), *N*,*N*-dimethyl-4-aminopyridine (DMAP: 610.8 mg, 5 mmol, 25 mol%) and *N*,*N*-dimethylcarbamoyl chloride (2.5 mL, 27.2 mmol, 1.3 equiv). This mixture was stirred overnight at 80 °C. The reaction mixture was quenched by the addition of saturated NH_4Cl aq, and then the mixture was extracted three times with CH_2Cl_2. The combined organic layer was washed with water and brine, dried over Na_2SO_4, concentrated *in vacuo*. The residue was purified by flash silica-gel column chromatography (hexane/EtOAc = 5:1) to afford **2r** as a colorless oil (3.48 g, 85% yield, *E*/*Z* = 5:3). ^{1}H NMR (400 MHz, $CDCl_3$): δ 7.49–7.21 (m, 15H, (*E* and *Z*)), 7.16 (d, *J* = 1.4 Hz, 1H, (*Z*)), 3.01 (s, 5H, (*E*)), 2.96 (s, 5H, (*E*)), 2.91 (s, 3H, (*Z*)), 2.84 (s, 3H, (*Z*)), 2.07 (d, *J* = 1.4 Hz, 5H, (*E*)), 1.99 (d, *J* = 1.4 Hz, 3H, (*Z*)); ^{13}C NMR (100 MHz, $CDCl_3$): δ 153.5, 139.2, 137.8, 133.8, 131.8, 128.2, 127.7, 127.6, 126.6, 125.4, 118.7, 116.8, 36.35, 36.27, 35.74, 35.70, 18.5, 13.3; HRMS (DART) *m/z* calcd for $C_{12}H_{16}NO_2$ $[M+H]^+$: 206.1181, found 206.1184.

***tert*-Pentyl 2-(1-(4-chlorobenzoyl)-5-methoxy-2-methyl-1*H*-indol-3-yl)acetate (4t)**

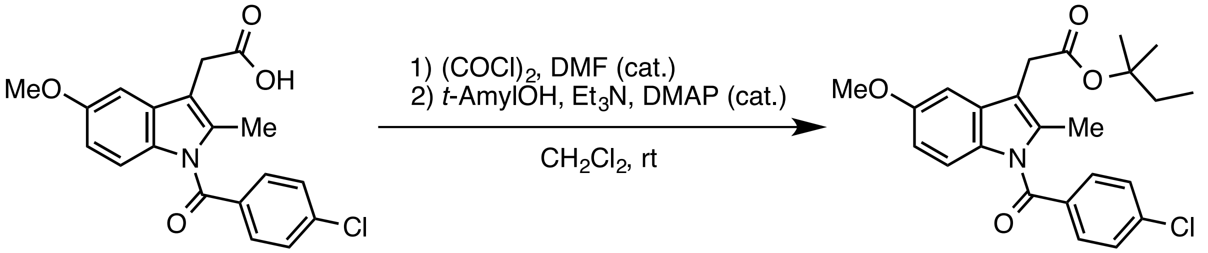

indomethacin **4t: 33% yield**

To a solution of indomethacin (3.58 g, 10 mmol, 1.0 equiv) in CH_2Cl_2 (10 mL) were added oxalyl chloride (1.29 mL, 15 mmol, 1.5 equiv) and a few drops of DMF at room temperature, and then this solution was stirred for 1 h. To the mixture were added *t*-AmylOH (3.24 mL, 30 mmol, 3.0 equiv),

Et_3N (3.06 mL, 22 mmol, 2.2 equiv), and *N,N*-dimethyl-4-aminopyridine (DMAP: 12.2 mg, 0.10 mmol, 1.0 equiv) at 0 °C. This solution was allowed to warm up to room temperature and stirred for 3 h. The reaction was quenched by the addition of $NaHCO_3$ aq and extracted three times with CH_2Cl_2, dried over Na_2SO_4, and then filtered. The resulting solution was concentrated *in vacuo*. The crude mixture was purified by flash silica-gel column chromatography (hexane/EtOAc = 10:1 to 4:1) to afford **4t** as a yellow solid (1.41 g, 33% yield). ^{1}H NMR (400 MHz, $CDCl_3$): δ 7.65 (d, *J* = 8.8 Hz, 2H), 7.46 (d, *J* = 8.8 Hz, 2H), 6.96 (d, *J* = 2.0 Hz, 1H), 6.88 (d, *J* = 9.2 Hz, 1H), 6.66 (dd, *J* = 9.2, 2.0 Hz, 1H), 3.83 (s, 3H), 3.58 (s, 2H), 2.37 (s, 3H), 1.75 (q, *J* = 8.0 Hz, 2H), 1.41 (s, 6H), 0.81 (t, *J* = 8.0 Hz, 3H); ^{13}C NMR (100 MHz, $CDCl_3$): δ 170.0, 168.2, 155.9, 139.1, 135.6, 134.0, 131.1, 130.8, 130.7, 129.0, 114.9, 113.3, 111.6, 101.2, 83.5, 55.6, 33.3, 31.6, 25.5, 13.3, 8.1; HRMS (DART) *m/z* calcd for $C_{24}H_{27}ClNO_4$ $[M+H]^+$: 428.1629, found 428.1627.

Benzo[*b*]thiophene-2,3-diylbis(dicyclohexylphosphine) (L1)

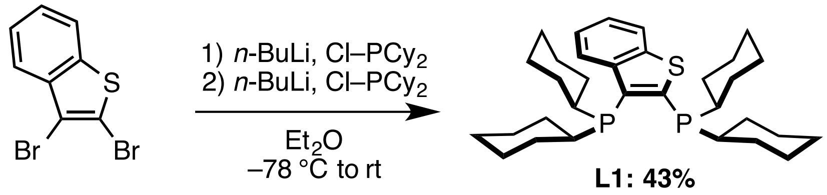

To a 50-mL round-bottom glass flask containing a magnetic stirring bar were added 2,3-dibromobenzo[*b*]thiophene (1.46 g, 5.0 mmol, 1.0 equiv) and dry Et_2O (5 mL). After cooling at –78 °C, a solution of *n*-BuLi in hexane (1.6 M, 3.2 mL, 1.0 equiv) was added at –78 °C over 10 min. After stirring the mixture at –78 °C for 1 h, a solution of chlorodicyclohexylphosphine (1.22 g, 5.25 mmol, 1.05 equiv) in Et_2O (1.5 mL) was added at –78 °C over 15 min. The resulting mixture was further stirred at –78 °C for 30 min. Then a solution of *n*-BuLi in hexane (1.6 M, 3.2 mL, 1.0 equiv) was added at –78 °C over 10 min. After stirring the mixture at –78 °C for 1 h, a solution of chlorodicyclohexylphosphine (1.22 g, 5.25 mmol, 1.05 equiv) in Et_2O (1.5 mL) was added at –78 °C over 10 min. The resulting mixture was further stirred at –78 °C for 30 min. After warming to room temperature, the reaction was quenched with water, extracted with hexane, washed with brine, dried over Na_2SO_4, and concentrated *in vacuo*. The reaction mixture was purified by reprecipitation with toluene (0.50 mL) and methanol (25 mL) to afford benzo[*b*]thiophene-2,3-diylbis(dicyclohexylphosphine) (**L1**) as a white solid (1.13 g, 43% yield). ^{1}H NMR (400 MHz, $CDCl_3$) δ 8.07 (brs, 1H), 7.86 (dd, *J* = 8.4, 4.0 Hz, 1H), 7.42–7.31 (m, 2H), 2.49 (brs, 2H), 2.00 (brs, 6H), 1.90–1.50 (m, 14H), 1.49–0.95 (m, 22H); ^{31}P NMR (162 MHz, $CDCl_3$) δ –13.2 (d, *J* = 160 Hz, 1P), –16.2 (d, *J* = 160 Hz, 1P); HRMS (DART) *m/z* calcd for $C_{32}H_{49}P_2S$ $[M+H]^+$: 527.3030 found 527.3034.

2,3-Bis(dicyclohexylphosphino)thiophene (L2)

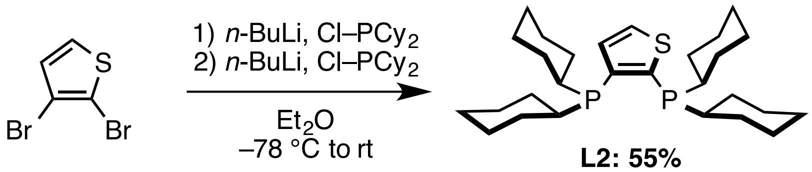

L2: 55%

To a 50-mL round-bottom glass flask containing a magnetic stirring bar were added 2,3-dibromothiophene (967 mg, 4.0 mmol, 1.0 equiv) and dry Et_2O (4 mL). After cooling at –78 °C, a solution of *n*-BuLi in hexane (1.6 M, 2.5 mL, 1.0 equiv) was added at –78 °C over 10 min. After stirring the mixture at –78 °C for 1 h, a solution of chlorodicyclohexylphosphine (977 mg, 4.2 mmol, 1.05 equiv) in Et_2O (1.25 mL) was added at –78 °C over 15 min. The resulting mixture was further stirred at –78 °C for 30 min. Then a solution of *n*-BuLi in hexane (1.6 M, 2.5 mL, 1.0 equiv) was added at –78 °C over 10 min. After stirring the mixture at –78 °C for 1 h, a solution of chlorodicyclohexylphosphine (977 mg, 4.2 mmol, 1.05 equiv) in Et_2O (1.25 mL) was added at –78 °C over 10 min. The resulting mixture was further stirred at –78 °C for 30 min. After warming up to room temperature, the reaction was quenched with water, extracted with hexane, washed with brine, dried over Na_2SO_4, and concentrated *in vacuo*. The reaction mixture was purified by reprecipitation with toluene (0.40 mL) and methanol (20 mL) to afford 2,3-bis(dicyclohexylphosphino)thiophene (**L2**) as a white solid (1.05 g, 55% yield). ^{1}H NMR (400 MHz, $CDCl_3$) δ 7.56 (d, *J* = 5.2 Hz, 1H), 7.17 (d, *J* = 5.2 Hz, 1H) 1.93–1.80 (m, 8H), 1.78–1.47 (m, 16H), 1.32–1.00 (m, 20H); ^{31}P NMR (162 MHz, $CDCl_3$) δ –19.0 (d, *J* = 129 Hz, 1P), –20.8 (d, *J* = 129 Hz, 1P); HRMS (DART) *m/z* calcd for $C_{28}H_{47}P_2S$ $[M+H]^+$: 477.2874 found 477.2867.

3. Ni-Catalyzed C–H Arylation of Imidazoles with Phenol Derivatives (C–H/C–O Coupling) and Chloroarenes (C–H/C–Cl Coupling)

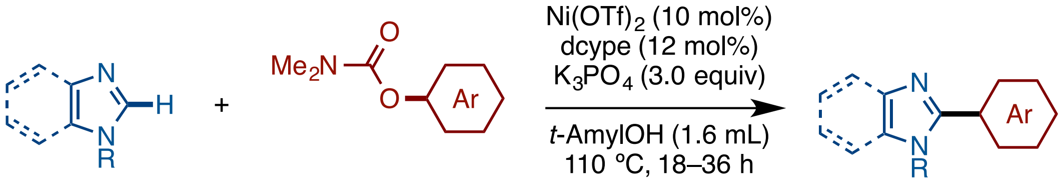

1 (0.40 mmol) **2** (0.60 mmol) **3**

General Procedure: A 20-mL glass vessel equipped with a J. Young® O-ring tap containing a magnetic stirring bar and K_3PO_4 (255.0 mg, 1.20 mmol, 3.0 equiv) was dried with a heatgun for 3 min *in vacuo* and filled with N_2 after cooling to room temperature. To this vessel were added $Ni(OTf)_2$ (14.2 mg, 0.040 mmol, 10 mol%), imidazole **1** (0.40 mmol, 1.0 equiv) and aryl carbamate **2** (0.60 mmol, 1.5 equiv). Then the vessel was introduced into an argon-atmosphere glovebox. To the reaction vessel was added 1,2-bis(dicyclohexylphosphino)ethane (dcype: 20.6 mg, 0.050 mmol, 12 mol%). The vessel was taken out of the glovebox, then dry *t*-AmylOH (1.6 mL) was added under a stream of N_2. The vessel was sealed with an O-ring tap and then heated at 110 °C for 18–36 h in an 8-well reaction block with stirring. After cooling the reaction mixture to room temperature, the mixture was passed through a silica gel pad with EtOAc as the eluent. The filtrate was concentrated and the residue was subjected to PTLC or flash silica-gel column chromatography to afford 2-arylimidazole **3**.

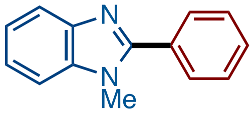

3Aa: 82% (C–H/C–O coupling)
81% (C–H/C–Cl coupling)

1-Methyl-2-phenyl-1*H*-benzo[*d*]imidazole (3Aa)[16]: Purification by PTLC (hexane/EtOAc = 4:1) afforded **3Aa** as a white solid (68.2 mg, 82% yield by C–H/C–O coupling: 67.3 mg, 81% yield by C–H/C–Cl coupling). ^{1}H NMR (400 MHz, $CDCl_3$): δ 7.85–7.81 (m, 1H), 7.79–7.75 (m, 2H), 7.55–7.49 (m, 3H), 7.41–7.37 (m, 1H), 7.35–7.28 (m, 2H), 3.86 (s, 3H); ^{13}C NMR (100 MHz, $CDCl_3$): δ 153.6, 142.9, 136.5, 130.1, 129.6, 129.3, 128.5, 122.6, 122.3, 119.7, 109.5, 31.5; HRMS (DART) *m/z* calcd for $C_{14}H_{13}N_2$ $[M+H]^+$: 209.1079, found 209.1076.

16 J. Huang, J. Chan, Y. Chen, C. J. Borths, K. D. Baucom, R. D. Larsen and M. M. Faul, *J. Am. Chem. Soc.*, 2010, **132**, 3674.

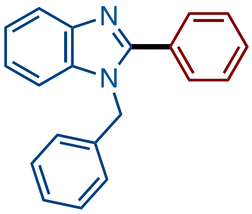

3Ba: 53% (C–H/C–O coupling)
69% (C–H/C–Cl coupling)

1-Benzyl-2-phenyl-1*H*-benzo[*d*]imidazole (3Ba)[17]: Purification by Isolera® (hexane/EtOAc = 10:1 to EtOAc) afforded **3Ba** as a white solid (60.5 mg, 53% yield by C–H/C–O coupling: 78.1 mg, 69% yield by C–H/C–Cl coupling). ^{1}H NMR (400 MHz, $CDCl_3$): δ 7.87 (d, *J* = 8.0 Hz, 1H), 7.68 (d, *J* = 8.0 Hz, 2H), 7.48–7.38 (m, 3H), 7.35–7.24 (m, 4H), 7.22 (dd, *J* = 7.2, 5.4 Hz, 2H), 7.09 (d, *J* = 5.4 Hz, 2H), 5.44 (s, 2H); ^{13}C NMR (100 MHz, $CDCl_3$): δ 154.1, 143.2, 136.3, 136.0, 130.1, 129.8, 129.2, 129.0, 128.7, 127.7, 125.9, 123.0, 122.6, 119.9, 110.5, 48.3; HRMS (DART) *m/z* calcd for $C_{20}H_{17}N_2$ $[M+H]^+$: 285.1392, found 285.1390

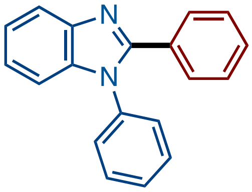

3Ca: 88% (C–H/C–O coupling)
86% (C–H/C–Cl coupling)

1,2-Diphenyl-1*H*-benzo[*d*]imidazole (3Ca)[18]: Purification by Isolera® (hexane/EtOAc = 10:1 to EtOAc) afforded **3Ca** as a white solid (95.0 mg, 88% yield by C–H/C–O coupling: 93.2 mg, 86% yield by C–H/C–Cl coupling). ^{1}H NMR (400 MHz, $CDCl_3$): δ 7.89 (d, *J* = 7.2 Hz, 1H), 7.56 (d, *J* = 8.4 Hz, 2H), 7.53–7.41 (m, 3H), 7.38–7.22 (m, 8H); ^{13}C NMR (100 MHz, $CDCl_3$): δ 152.3, 143.0, 137.2, 136.9, 129.9, 129.8, 129.39, 129.36, 128.5, 128.2, 127.3, 123.3, 122.9, 119.8, 110.4; HRMS (DART) *m/z* calcd for $C_{19}H_{15}N_2$ $[M+H]^+$: 271.1235, found 271.1230.

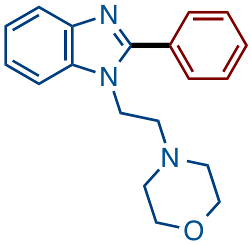

3Da: 84%

17 M. M. Guru, M. A. Ali and T. Punniyamurthy, *J. Org. Chem.*, 2011, **76**, 5295.
18 P. Saha, T. Ramana, N. Purkait, M. A. Ali, R. Paul and T. Punniyamurthy, *J. Org. Chem.*, 2009, **74**, 8719.

4-(2-(2-Phenyl-1*H*-benzo[*d*]imidazol-1-yl)ethyl)morpholine (3Da): Purification by PTLC (EtOAc) afforded **3Da** as a yellow oil (103.3 mg, 84% yield). ^{1}H NMR (400 MHz, $CDCl_3$): δ 7.83 (dd, J = 7.6, 2.4 Hz, 1H), 7.79–7.74 (m, 2H), 7.55–7.45 (m, 3H), 7.43–7.38 (m, 1H), 7.34–7.26 (m, 2H), 4.35 (t, J = 6.8 Hz, 2H), 3.54 (t, J = 4.4 Hz, 4H), 2.70 (t, J = 6.8 Hz, 2H), 2.30 (t, J = 4.4 Hz, 4H); ^{13}C NMR (100 MHz, $CDCl_3$): δ 154.0, 143.0, 135.4, 130.5, 129.7, 129.4, 128.6, 122.7, 122.4, 119.9, 109.9, 66.6, 57.4, 53.7, 42.2; HRMS (DART) *m/z* calcd for $C_{19}H_{22}N_3O$ [M+H]$^+$: 308.1763, found 308.1767

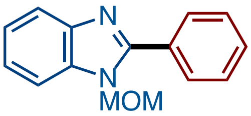

3Ea: 53%

1-(Methoxymethyl)-2-phenyl-1*H*-benzo[*d*]imidazole (3Ea): Purification by PTLC (hexane/EtOAc = 4:1) afforded **3Ea** as a yellow solid (50.1 mg, 53% yield). ^{1}H NMR (400 MHz, $CDCl_3$): δ 7.95–7.88 (m, 2H), 7.86–7.81 (m, 1H), 7.55–7.47 (m, 4H), 7.35–7.29 (m, 2H), 5.45 (s, 2H), 3.39 (s, 3H); ^{13}C NMR (100 MHz, $CDCl_3$): δ 154.4, 142.8, 136.0, 130.0, 129.7, 129.5, 128.6, 123.2, 122.9, 119.9, 110.0, 75.0, 56.5; HRMS (DART) *m/z* calcd for $C_{15}H_{15}N_2O$ [M+H]$^+$: 239.1184, found 239.1183

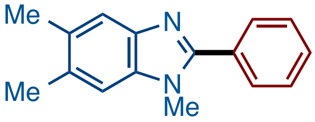

3Fa: 65%

1,5,6-Trimethyl-2-phenyl-1*H*-benzo[*d*]imidazole (3Fa): Purification by Isolera® (hexane/EtOAc = 10:1 to 1:1) afforded **3Fa** as a white solid (69.1 mg, 65% yield). ^{1}H NMR (400 MHz, $CDCl_3$): δ 7.72 (d, J = 7.6 Hz, 2H), 7.57 (s, 1H), 7.51–7.41 (m, 3H), 7.12 (s, 1H), 3.76 (s, 3H), 2.40 (s, 3H), 2.39 (s, 3H); ^{13}C NMR (100 MHz, $CDCl_3$): δ 152.8, 141.5, 135.1, 131.8, 131.1, 130.4, 129.3, 129.2, 128.5, 119.7, 109.8, 31.5, 20.5, 20.2; HRMS (DART) *m/z* calcd for $C_{16}H_{17}N_2$ [M+H]$^+$: 237.1392, found 237.1396.

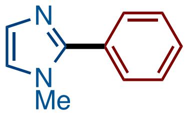

3Ga: 64% (C–H/C–O coupling)
81% (C–H/C–Cl coupling)

1-Methyl-2-phenyl-1*H*-imidazole (3Ga)[19]: The reaction was performed by using $Ni(cod)_2$ (11.0 mg, 0.040 mmol, 10 mol%) instead of $Ni(OTf)_2$ for 36 h. Purification by PTLC (EtOAc) afforded **3Ga** as a colorless oil (40.5 mg, 64% yield by C–H/C–O coupling: 51.8 mg, 81% yield by C–H/C–Cl coupling).

19 H.-Q. Do, R. M. K. Khan and O. Daugulis, *J. Am. Chem. Soc.*, 2008, **130**, 15185.

^{1}H NMR (400 MHz, CDCl$_3$): δ 7.63 (d, J = 8.0 Hz, 2H), 7.49–7.38 (m, 3H), 7.13 (d, J = 1.6 Hz, 1H), 6.97 (d, J = 1.6 Hz, 1H), 3.76 (s, 3H); ^{13}C NMR (100 MHz, CDCl$_3$): δ 147.7, 130.5, 128.51, 128.47, 128.4, 128.3, 122.2, 34.4; HRMS (DART) m/z calcd for $C_{10}H_{11}N_2$ $[M+H]^+$: 159.0922, found 159.0920.

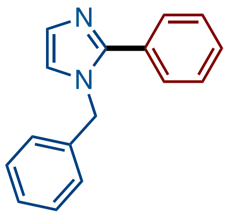

3Ha: 64% (C–H/C–O coupling)
64% (C–H/C–Cl coupling)

1-Benzyl-2-phenyl-1*H*-imidazole (3Ha): The reaction was performed by using Ni(cod)$_2$ (11.0 mg, 0.040 mmol, 10 mol%) instead of Ni(OTf)$_2$ for 36 h. Purification by PTLC (EtOAc) afforded **3Ha** as a yellow solid (59.8 mg, 64% yield by C–H/C–O coupling: 60.4 mg, 64% yield by C–H/C–Cl coupling). ^{1}H NMR (400 MHz, CDCl$_3$): δ 7.59–7.51 (m, 2H), 7.42–7.25 (m, 6H), 7.18 (s, 1H), 7.07 (d, J = 7.2 Hz, 2H), 6.95 (s, 1H), 5.20 (s, 2H); ^{13}C NMR (100 MHz, CDCl$_3$): δ 148.1, 136.9, 130.4, 128.9, 128.8, 128.68, 128.66, 128.4, 127.8, 126.4, 121.2, 50.2; HRMS (DART) m/z calcd for $C_{16}H_{15}N_2$ $[M+H]^+$: 235.1235, found 235.1230.

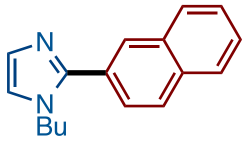

3Ib: 49%

1-Butyl-2-(naphthalen-2-yl)-1*H*-imidazole (3Ib): The reaction was performed by using Ni(cod)$_2$ (11.0 mg, 0.040 mmol, 10 mol%) instead of Ni(OTf)$_2$ for 36 h. Purification by Isolera® (hexane/EtOAc = 10:1 to EtOAc) afforded **3Ib** as a yellow solid (48.7 mg, 49% yield). ^{1}H NMR (400 MHz, CDCl$_3$): δ 8.04 (s, 1H), 7.95–7.81 (m, 3H), 7.71 (d, J = 7.6 Hz, 1H), 7.55–7.46 (m, 2H), 7.19 (s, 1H), 7.05 (s, 1H), 4.06 (t, J = 7.6 Hz, 2H), 1.79–1.70 (m, 2H), 1.33–1.21 (m, 2H), 0.86 (t, J = 7.6 Hz, 3H); ^{13}C NMR (100 MHz, CDCl$_3$): δ 147.6, 133.0, 128.6, 128.3, 128.22, 128.17, 128.1, 127.7, 126.5, 126.4, 126.3, 120.5, 46.6, 33.1, 19.2, 13.5 (one peak is overlapping); HRMS (DART) m/z calcd for $C_{17}H_{19}N_2$ $[M+H]^+$: 251.1548, found 251.1542.

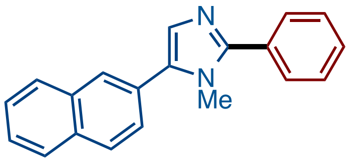

3Ja: 82%

1-Methyl-5-(naphthalen-2-yl)-2-phenyl-1*H*-imidazole (3Ja): The reaction was performed by using Ni(cod)$_2$ (11.0 mg, 0.040 mmol, 10 mol%) instead of Ni(OTf)$_2$ for 36 h. Purification by flash silica-gel column chromatography (hexane/EtOAc = 4:1 to EtOAc) afforded **3Ja** as a white solid (93.3 mg, 82% yield). ^{1}H NMR (400 MHz, CDCl$_3$): δ 7.95–7.84 (m, 4H), 7.73 (d, *J* = 8.0 Hz, 2H), 7.57 (d, *J* = 8.0 Hz, 1H), 7.56–7.46 (m, 4H), 7.42 (t, *J* = 8.0 Hz, 1H), 7.32 (s, 1H), 3.73 (s, 3H); ^{13}C NMR (100 MHz, CDCl$_3$): δ 149.6, 135.4, 133.3, 132.6, 130.9, 128.8, 128.7, 128.5, 128.4, 128.0, 127.7, 127.6, 127.4, 126.6, 126.44, 126.36, 33.9 (one peak is overlapping); HRMS (DART) *m/z* calcd for $C_{20}H_{17}N_2$ [M+H]$^+$: 285.1392, found 285.1397.

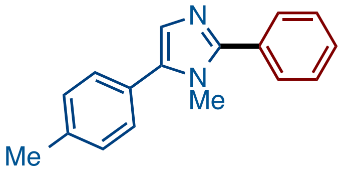

3Ka: 65%

1-Methyl-2-phenyl-5-(*p*-tolyl)-1*H*-imidazole (3Ka): The reaction was performed by using Ni(cod)$_2$ (11.0 mg, 0.040 mmol, 10 mol%) instead of Ni(OTf)$_2$ for 36 h. Purification by flash silica-gel column chromatography (hexane/EtOAc = 4:1 to 1:1) afforded **3Ka** as a white solid (65.0 mg, 65% yield). ^{1}H NMR (400 MHz, CDCl$_3$): δ 7.69 (d, *J* = 8.4 Hz, 2H), 7.46 (dd, *J* = 8.4, 7.6 Hz, 2H), 7.40 (t, *J* = 7.6 Hz, 1H), 7.34 (d, *J* = 8.0 Hz, 2H), 7.26 (d, *J* = 8.0 Hz, 2H), 7.17 (s, 1H), 3.66 (s, 3H), 2.40 (s, 3H); ^{13}C NMR (100 MHz, CDCl$_3$): δ 149.1, 137.7, 135.4, 131.0, 129.4, 128.7, 128.52, 128.50, 128.4, 127.3, 127.2, 33.6, 21.2; HRMS (DART) *m/z* calcd for $C_{17}H_{17}N_2$ [M+H]$^+$: 249.1392, found 249.1395.

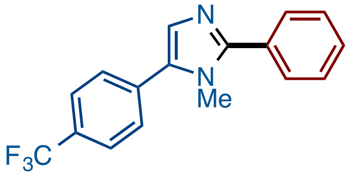

3La: >95%

1-Methyl-2-phenyl-5-(4-(trifluoromethyl)phenyl)-1*H*-imidazole (3La)[20]**:** The reaction was performed by using Ni(cod)$_2$ (11.0 mg, 0.040 mmol, 10 mol%) instead of Ni(OTf)$_2$ for 36 h. Purification by PTLC (hexane/EtOAc = 1:1) afforded **3La** as a white solid (122.4 mg, >95% yield). ^{1}H NMR (400 MHz, CDCl$_3$): δ 7.74–7.67 (m, 4H), 7.58 (d, *J* = 8.0 Hz, 2H), 7.52–7.41 (m, 3H), 7.28 (s, 1H), 3.70 (s, 3H); ^{13}C NMR (100 MHz, CDCl$_3$): δ 150.4, 134.0, 133.8, 130.5, 129.7 (q, $J_{C–F}$ = 32.5 Hz), 129.0, 128.8, 128.7, 128.6, 128.5, 125.7 (q, $J_{C–F}$ = 4.0 Hz), 124.0 (q, $J_{C–F}$ = 273 Hz), 33.9; HRMS (DART) *m/z* calcd for $C_{17}H_{14}F_3N_2$ [M+H]$^+$: 303.1109, found 303.1108.

20 F. Shibahara, E. Yamaguchi and T. Murai, *J. Org. Chem.*, 2011, **76**, 2680.

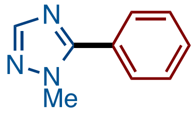

3Ma: 82%

1-Methyl-5-phenyl-1*H*-1,2,4-triazole (3Ma)[19]**:** The reaction was performed by using Ni(cod)$_2$ (11.0 mg, 0.040 mmol, 10 mol%) instead of Ni(OTf)$_2$ for 36 h. Purification by PTLC (EtOAc) afforded **3Ma** as a white solid (52.2 mg, 82% yield). ^{1}H NMR (400 MHz, CDCl$_3$): δ 7.94 (s, 1H), 7.70–7.66 (m, 2H), 7.54–7.48 (m, 3H), 3.99 (s, 3H); ^{13}C NMR (100 MHz, CDCl$_3$): δ 154.5, 150.6, 130.0, 128.8, 128.5, 127.8, 36.9; HRMS (DART) *m/z* calcd for $C_9H_{10}N_3$ [M+H]$^+$: 160.0875, found 160.0875.

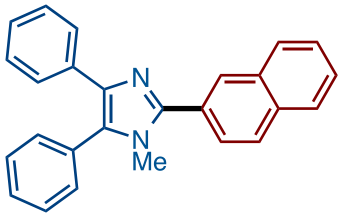

3Nb: 87%

1-Methyl-2-(naphthalen-2-yl)-4,5-diphenyl-1*H*-imidazole (3Nb): The reaction was performed by using Ni(cod)$_2$ (11.0 mg, 0.040 mmol, 10 mol%) and dcypt (22.9 mg, 0.050 mmol, 12 mol%) instead of Ni(OTf)$_2$/dcype for 36 h. Purification by Isolera® (hexane/EtOAc = 10:1 to EtOAc) afforded **3Nb** as a white solid (125.1 mg, 87% yield). ^{1}H NMR (400 MHz, CDCl$_3$): δ 8.21 (s, 1H), 7.98–7.85 (m, 4H), 7.58 (d, J = 8.4 Hz, 2H), 7.56–7.41 (m, 7H), 7.23 (t, J = 8.0 Hz, 2H), 7.15 (t, J = 7.6 Hz, 1H), 3.58 (s, 3H); ^{13}C NMR (100 MHz, CDCl$_3$): δ 147.9, 138.0, 134.7, 133.2, 133.1, 131.2, 130.9, 130.7, 129.0, 128.6, 128.4, 128.33, 128.26, 128.1, 127.8, 127.0, 126.6, 126.52, 126.49, 126.3, 33.3 (one peak is overlapping); HRMS (DART) *m/z* calcd for $C_{26}H_{21}N_2$ [M+H]$^+$: 361.1705, found 361.1708.

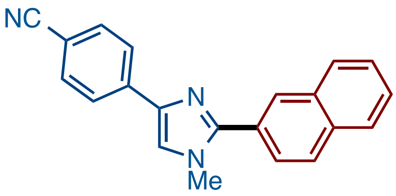

3Ob: 72%

4-(1-Methyl-2-(naphthalen-2-yl)-1*H*-imidazol-4-yl)benzonitrile (3Ob): The reaction was performed by using Ni(cod)$_2$ (11.0 mg, 0.040 mmol, 10 mol%) and dcypt (22.9 mg, 0.050 mmol, 12 mol%) instead of Ni(OTf)$_2$/dcype for 36 h. Purification by Isolera® (hexane/EtOAc = 10:1 to EtOAc) afforded **3Ob** as a yellow solid (89.2 mg, 72% yield). ^{1}H NMR (400 MHz, CDCl$_3$): δ 8.12 (s, 1H), 7.98–7.86 (m, 5H), 7.80 (dd, J = 8.4, 1.6 Hz, 1H), 7.64 (d, J = 8.4 Hz, 2H), 7.58–7.50 (m, 2H), 7.39 (s,

1H), 3.83 (s, 3H); ^{13}C NMR (100 MHz, $CDCl_3$): δ 148.9, 139.4, 138.6, 133.3, 133.0, 132.4, 128.4, 128.3, 128.2, 127.8, 127.3, 126.9, 126.7, 126.0, 125.1, 120.1, 119.3, 109.6, 34.9; HRMS (DART) *m/z* calcd for $C_{21}H_{16}N_3$ $[M+H]^+$: 310.1344, found 310.1349.

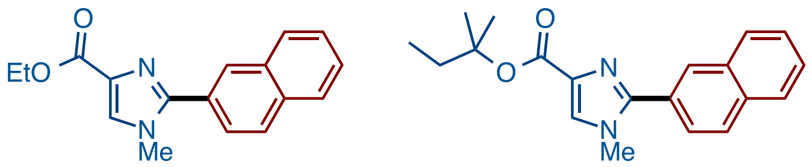

3Pb **3Pb'**

93% (combined yield)

Ethyl 1-methyl-2-(naphthalen-2-yl)-1*H*-imidazole-4-carboxylate (3Pb), *tert*-Pentyl 1-methyl-2-(naphthalen-2-yl)-1*H*-imidazole-4-carboxylate (3Pb'): The reaction was performed by using $Ni(cod)_2$ (11.0 mg, 0.040 mmol, 10 mol%) and dcypt (22.9 mg, 0.050 mmol, 12 mol%) instead of $Ni(OTf)_2$/dcype and **1P** as a starting material for 36 h. Purification by Isolera® (hexane/EtOAc = 10:1 to EtOAc) afforded **3Pb** (37.5 mg, 33% yield, white solid) and **3Pb'** (77.6 mg, 60% yield, white solid).

3Pb: ^{1}H NMR (400 MHz, $CDCl_3$): δ 8.11 (s, 1H), 7.93–7.83 (m, 3H), 7.76 (d, *J* = 8.8 Hz, 1H), 7.69 (s, 1H), 7.55–7.48 (m, 2H), 4.41 (q, *J* = 7.2 Hz, 2H), 3.80 (s, 3H), 1.39 (t, *J* = 7.2 Hz, 3H); ^{13}C NMR (100 MHz, $CDCl_3$): δ 163.0, 148.8, 133.2, 132.9, 132.7, 128.6, 128.3, 128.2, 127.7, 126.9, 126.8, 126.6, 126.0, 60.4, 35.0, 14.4 (one peak is overlapping); HRMS (DART) *m/z* calcd for $C_{17}H_{17}N_2O_2$ $[M+H]^+$: 281.1290, found 281.1289.

3Pb': ^{1}H NMR (400 MHz, $CDCl_3$): δ 8.13 (s, 1H), 7.93–7.85 (m, 3H), 7.79 (d, *J* = 8.0 Hz, 1H), 7.58 (s, 1H), 7.54–7.50 (m, 2H), 3.82 (s, 3H), 1.94 (q, *J* = 7.2 Hz, 2H), 1.57 (s, 6H), 0.97 (t, *J* = 7.2 Hz, 3H); ^{13}C NMR (100 MHz, $CDCl_3$): δ 162.1, 148.6, 134.2, 133.3, 132.9, 128.5, 128.3, 128.2, 127.7, 127.6, 127.2, 126.9, 126.6, 126.2, 83.2, 35.0, 33.6, 25.8, 8.4; HRMS (DART) *m/z* calcd for $C_{20}H_{23}N_2O_2$ $[M+H]^+$: 323.1760, found 323.1757.

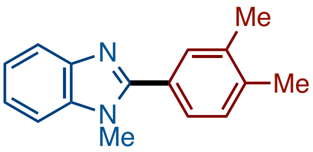

3Ac: 74%

2-(3,4-Dimethylphenyl)-1-methyl-1*H*-benzo[*d*]imidazole (3Ac): Purification by PTLC (hexane/EtOAc = 4:1) afforded **3Ac** as a white solid (69.6 mg, 74% yield). ^{1}H NMR (400 MHz, $CDCl_3$): δ 7.84–7.79 (m, 1H), 7.57 (s, 1H), 7.42 (d, *J* = 7.6 Hz, 1H), 7.35–7.22 (m, 4H), 3.79 (s, 3H), 2.324 (s, 3H), 2.317 (s, 3H); ^{13}C NMR (100 MHz, $CDCl_3$): δ 153.9, 142.8, 138.4, 137.0, 136.5, 130.5, 129.6, 127.5, 126.5, 122.4, 122.1, 119.5, 109.4, 31.5, 19.6 (one peak is overlapping); HRMS (DART) *m/z* calcd for $C_{16}H_{17}N_2$ $[M+H]^+$: 237.1392, found 237.1387.

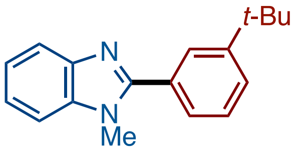

3Ad: 74%

2-(3-(*tert*-Butyl)phenyl)-1-methyl-1*H*-benzo[*d*]imidazole (3Ad): Purification by PTLC (hexane/EtOAc = 4:1) afforded **3Ad** as a white solid (78.0 mg, 74% yield). ^{1}H NMR (400 MHz, $CDCl_3$): δ 7.87–7.83 (m, 1H), 7.82–7.77 (m, 1H), 7.57–7.48 (m, 2H), 7.46 (dd, *J* = 8.0, 7.6 Hz, 1H), 7.42–7.36 (m, 1H), 7.35–7.27 (m, 2H), 3.86 (s, 3H), 1.39 (s, 9H); ^{13}C NMR (100 MHz, $CDCl_3$): δ 154.4, 151.7, 143.0, 136.6, 129.9, 128.3, 126.8, 126.6, 126.5, 122.6, 122.3, 119.8, 109.5, 34.9, 31.6, 31.3; HRMS (DART) *m/z* calcd for $C_{18}H_{21}N_2$ [M+H]$^+$: 265.1705, found 265.1700

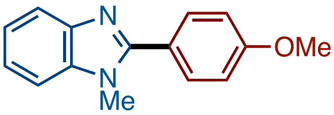

3Ae: 58%

2-(4-Methoxyphenyl)-1-methyl-1*H*-benzo[*d*]imidazole (3Ae): Purification by PTLC (hexane/EtOAc = 1:1) afforded **3Ae** as a white solid (57.2 mg, 58% yield). ^{1}H NMR (400 MHz, $CDCl_3$): δ 7.80 (dd, *J* = 8.0, 3.2 Hz, 1H), 7.67 (d, *J* = 8.0 Hz, 2H), 7.35–7.25 (m, 3H), 7.00 (d, *J* = 8.0 Hz, 2H), 3.84 (s, 3H), 3.78 (s, 3H); ^{13}C NMR (100 MHz, $CDCl_3$): δ 160.6, 153.6, 142.8, 136.4, 130.7, 122.4, 122.3, 122.1, 119.4, 114.0, 109.4, 55.2, 31.5; HRMS (DART) *m/z* calcd for $C_{15}H_{15}N_2O$ [M+H]$^+$: 239.1184, found 239.1181.

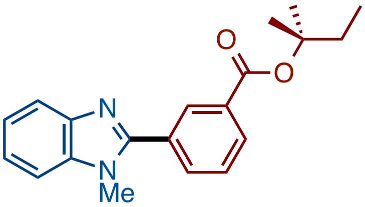

3Af: 81%

***tert*-Pentyl 3-(1-methyl-1*H*-benzo[*d*]imidazol-2-yl)benzoate (3Af):** Purification by PTLC (hexane/EtOAc = 10:1) and GPC afforded **3Af** as a colorless liquid (69.1 mg, 81% yield). ^{1}H NMR (400 MHz, $CDCl_3$): δ 8.37 (s, 1H), 8.13 (d, *J* = 6.8 Hz, 1H), 7.95 (d, *J* = 6.8 Hz, 1H), 7.84 (d, *J* = 4.0 Hz, 1H), 7.59 (t, *J* = 6.8 Hz, 1H), 7.42–7.30 (m, 3H), 3.85 (s, 3H), 1.94 (q, *J* = 7.2 Hz, 2H), 1.58 (s, 6H), 0.98 (t, *J* = 7.2 Hz, 3H); ^{13}C NMR (100 MHz, $CDCl_3$): δ 164.8, 152.7, 142.8, 136.4, 133.3, 132.4, 130.4, 130.3, 129.9, 128.6, 122.8, 122.4, 119.7, 109.6, 83.9, 33.5, 31.5, 25.5, 8.2; HRMS (DART) *m/z* calcd for $C_{20}H_{23}N_2O_2$ [M+H]$^+$: 323.1760, found 323.1756.

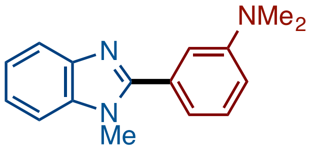

3Ag: 67%

***N*,*N*-Dimethyl-3-(1-methyl-1*H*-benzo[*d*]imidazol-2-yl)aniline (3Ag):** Purification by Isolera® (hexane/EtOAc = 10:1 to 1:2) afforded **3Ag** as a yellow oil (67.0 mg, 67% yield). ^{1}H NMR (400 MHz, $CDCl_3$): δ 7.86–7.80 (m, 1H), 7.41–7.27 (m, 4H), 7.11 (s, 1H), 7.00 (d, *J* = 8.4 Hz, 1H), 6.85 (d, *J* = 8.4 Hz, 1H), 3.84 (s, 3H), 3.01 (s, 6H); ^{13}C NMR (100 MHz, $CDCl_3$): δ 154.7, 150.7, 142.9, 136.5, 130.8, 129.0, 122.5, 122.2, 119.7, 117.2, 113.6, 113.4, 109.5, 40.5, 31.6; HRMS (DART) *m/z* calcd for $C_{16}H_{18}N_3$ $[M+H]^+$: 252.1501, found 252.1501.

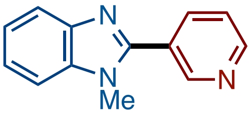

3Ah: 95%

1-Methyl-2-(pyridin-3-yl)-1*H*-benzo[*d*]imidazole (3Ah): Purification by Isolera® (hexane/EtOAc = 1:1 to EtOAc) afforded **3Ah** as a white solid (79.6 mg, 95% yield). ^{1}H NMR (400 MHz, $CDCl_3$): δ 9.02 (s, 1H), 8.75 (dd, *J* = 4.0, 1.6 Hz, 1H), 8.14 (dd, *J* = 8.0, 1.6 Hz, 1H), 7.85–7.80 (m, 1H), 7.52–7.30 (m, 4H), 3.89 (s, 3H); ^{13}C NMR (100 MHz, $CDCl_3$): δ 150.6, 149.8, 143.0, 136.8, 136.5, 126.5, 123.5, 123.3, 122.7, 120.0, 109.7, 31.6; HRMS (DART) *m/z* calcd for $C_{13}H_{12}N_3$ $[M+H]^+$: 210.1031, found 210.1025.

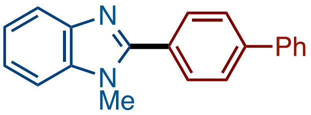

3Ai: 92%

2-([1,1'-Biphenyl]-4-yl)-1-methyl-1*H*-benzo[*d*]imidazole (3Ai)[21]**:** Purification by Isolera® (hexane/EtOAc = 5:1 to EtOAc) afforded **3Ai** as a white solid (105.2 mg, 92% yield). ^{1}H NMR (400 MHz, $CDCl_3$): δ 7.98–7.80 (m, 3H), 7.72 (d, *J* = 8.4 Hz, 2H), 7.63 (d, *J* = 8.0 Hz, 2H), 7.46 (t, *J* = 8.0 Hz, 2H), 7.40–7.27 (m, 4H), 3.85 (s, 3H); ^{13}C NMR (100 MHz, $CDCl_3$): δ 153.4, 142.9, 142.3, 140.0, 136.6, 129.7, 128.9, 128.8, 127.8, 127.2, 127.1, 122.7, 122.4, 119.7, 109.6, 31.7; HRMS (DART) *m/z* calcd for $C_{20}H_{17}N_2$ $[M+H]^+$: 285.1392, found 285.1393.

21 J. Sluiter and J. Christoffers, *Synlett*, 2009, 63.

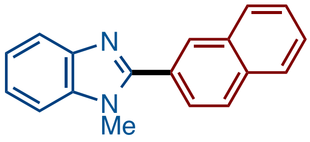

3Ab: >95%

1-Methyl-2-(naphthalen-2-yl)-1*H*-benzo[*d*]imidazole (3Ab): Purification by Isolera® (hexane/EtOAc = 10:1 to EtOAc) afforded **3Ab** as a white solid (103.0 mg, >95% yield). ^{1}H NMR (400 MHz, $CDCl_3$): δ 8.15 (s, 1H), 7.91–7.78 (m, 5H), 7.51–7.45 (m, 2H), 7.32–7.24 (m, 3H), 3.75 (s, 3H); ^{13}C NMR (100 MHz, $CDCl_3$): δ 153.5, 142.9, 136.5, 133.4, 132.7, 129.1, 128.3, 128.2, 127.6, 127.3, 127.0, 126.5, 126.1, 122.6, 122.3, 119.6, 109.5, 31.5; HRMS (DART) *m/z* calcd for $C_{18}H_{15}N_2$ $[M+H]^+$: 259.1235, found 259.1235.

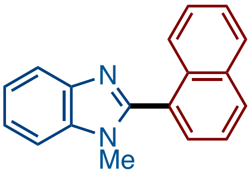

3Aj: 91%

1-Methyl-2-(naphthalen-1-yl)-1*H*-benzo[*d*]imidazole (3Aj): Purification by PTLC (hexane/EtOAc = 20:1 to EtOAc) afforded **3Aj** as a white solid (98.6 mg, 91% yield). ^{1}H NMR (400 MHz, $CDCl_3$): δ 8.01 (d, J = 8.4 Hz, 1H), 7.93 (d, J = 8.4 Hz, 1H), 7.92–7.87 (m, 1H), 7.73 (d, J = 8.0 Hz, 1H), 7.67 (d, J = 8.0 Hz, 1H), 7.59 (t, J = 8.4 Hz, 1H), 7.56–7.41 (m, 3H), 7.40–7.32 (m, 2H), 3.60 (s, 3H); ^{13}C NMR (100 MHz, $CDCl_3$): δ 152.9, 143.2, 135.9, 133.5, 132.1, 130.2, 128.8, 128.4, 127.8, 127.2, 126.3, 125.4, 125.0, 122.7, 122.3, 120.0, 109.5, 31.0; HRMS (DART) *m/z* calcd for $C_{18}H_{15}N_2$ $[M+H]^+$: 259.1235, found 259.1230.

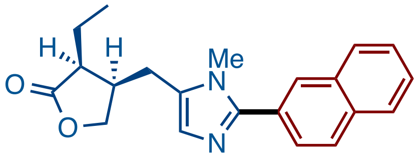

pilocarpine derivative
3Qb: 69%

(3*S*,4*R*)-3-Ethyl-4-((1-methyl-2-(naphthalen-2-yl)-1*H*-imidazol-5-yl)methyl)dihydrofuran-2(3*H*)-one (3Qb): Purification by Isolera® (hexane/EtOAc = 10:1 to EtOAc) afforded **3Qb** as a colorless oil (92.2 mg, 69% yield). ^{1}H NMR (400 MHz, $CDCl_3$): δ 8.03 (s, 1H), 7.93–7.83 (m, 3H), 7.71 (dd, J = 8.4, 2.0 Hz, 1H), 7.54–7.48 (m, 2H), 6.94 (s, 1H), 4.47 (dd, J = 9.2, 7.2 Hz, 1H), 3.98 (dd, J = 9.2, 7.2 Hz, 1H), 3.66 (s, 3H), 2.92 (dd, J = 15.6, 5.2 Hz, 1H), 2.81–2.63 (m, 2H), 2.33 (q, J = 6.4 Hz, 1H), 1.85–1.73 (m, 2H), 1.08 (t, J = 8.0 Hz, 3H); ^{13}C NMR (100 MHz, $CDCl_3$): δ 178.1, 148.8, 133.3, 133.2, 129.8, 128.34, 128.28, 128.1, 127.8, 126.81, 126.76, 126.7, 126.6, 126.3, 71.1, 46.7, 39.2, 32.0, 28.5, 22.6, 11.1; HRMS (DART) *m/z* calcd for $C_{21}H_{23}N_2O_2$ $[M+H]^+$: 335.1760, found 335.1762.

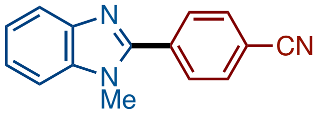

3As: 66%

4-(1-Methyl-1*H*-benzo[*d*]imidazol-2-yl)benzonitrile (3As): The reaction was performed by using Ni(cod)$_2$ (11.0 mg, 0.040 mmol, 10 mol%) instead of Ni(OTf)$_2$ for 36 h with 4-chlorobenzonitrile. Purification by Isolera® (hexane/EtOAc = 10:1 to EtOAc) afforded **3As** as a white solid (62.0 mg, 66% yield). ^{1}H NMR (400 MHz, CDCl$_3$): δ 7.92 (d, *J* = 8.4 Hz, 2H), 7.86–7.78 (m, 3H), 7.45–7.31 (m, 3H), 3.90 (s, 3H); ^{13}C NMR (100 MHz, CDCl$_3$): δ 151.4, 142.9, 136.7, 134.6, 132.4, 129.9, 123.6, 123.0, 120.2, 118.2, 113.3, 109.8, 31.8; HRMS (DART) *m/z* calcd for $C_{15}H_{12}N_3$ [M+H]$^+$: 234.1031, found 234.1038.

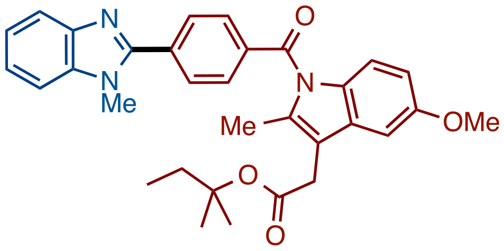

indomethacin derivative
3At: 26%

***tert*-Pentyl 2-(5-methoxy-2-methyl-1-(4-(1-methyl-1*H*-benzo[*d*]imidazol-2-yl)benzoyl)-1*H*-indol-3-yl)acetate (3At):** Purification by Isolera® (hexane/EtOAc = 100:1 to EtOAc) afforded **3At** as a white solid (55.0 mg, 26% yield). ^{1}H NMR (400 MHz, CDCl$_3$): δ 7.92 (d, *J* = 8.4 Hz, 2H), 7.87–7.82 (m, 3H), 7.45–7.30 (m, 3H), 7.00–6.96 (m, 2H), 6.67 (dd, *J* = 8.8, 2.0 Hz, 1H), 3.91 (s, 3H), 3.83 (s, 3H), 3.59 (s, 2H), 2.40 (s, 3H), 1.76 (q, *J* = 8.0 Hz, 2H), 1.42 (s, 6H), 0.82 (t, *J* = 8.0 Hz, 3H); ^{13}C NMR (100 MHz, CDCl$_3$): δ 170.0, 168.6, 156.0, 152.2, 142.9, 136.7, 135.6, 134.2, 130.8, 129.9, 129.7, 123.4, 122.8, 120.1, 115.1, 113.6, 111.7, 109.7, 101.4, 83.5, 55.6, 33.3, 31.8, 31.6, 25.5, 13.4, 8.1 (two peaks are overlapping); HRMS (ESI) *m/z* calcd for $C_{32}H_{33}N_3O_4Na$ [M+Na]$^+$: 546.2363, found 546.2360.

5. Ni-Catalyzed C–H Alkenylation of Imidazoles with Enol Derivatives

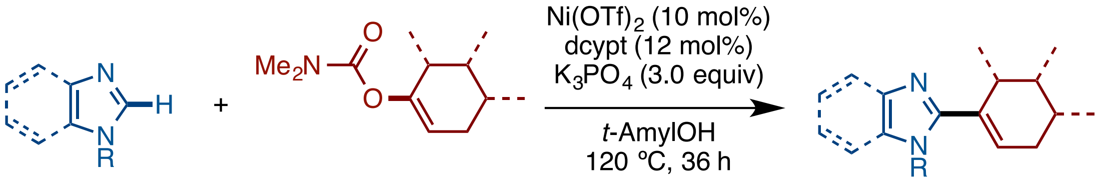

A 50-mL glass Schlenk tube containing a magnetic stirring bar and K_3PO_4 (670.3 mg, 3.0 mmol, 3.0 equiv) was dried with a heatgun for 3 min *in vacuo* and filled with argon after cooling to room temperature. $Ni(OTf)_2$ (35.7 mg, 0.10 mmol, 10 mol%), imidazole **1** (1.0 mmol, 1.0 equiv), alkenyl carbamate **2** (1.5 mmol, 1.5 equiv), and 3,4-bis(dicyclohexylphosphino)thiophene (dcypt: 57.2 mg, 0.12 mmol, 12 mol%) were placed in a 20-mL glass Schlenk tube under an argon atmosphere. Then, to it was added dry degassed *t*-AmylOH (4.0 mL) under a stream of argon. The resultant *t*-AmylOH solution was transferred into the 50-mL Schlenk tube under a stream of argon via cannula. The reaction mixture was stirred at 120 °C for 36 h. After cooling the reaction mixture to room temperature, the mixture was diluted with EtOAc, and the organic layer was washed with water and brine, dried over Na_2SO_4, and filtered. The filtrate was concentrated *in vacuo* and the residue was purified by flash silica-gel column chromatography to afford 2-alkenylimidazole **3**.

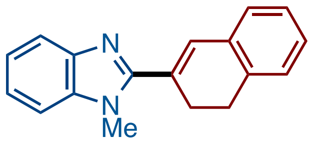

3Ak: 87%

2-(3,4-Dihydronaphthalen-2-yl)-1-methyl-1*H*-benzo[*d*]imidazole (3Ak): Purification by flash silica-gel column chromatography (hexane/EtOAc = 3:1) afforded **3Ak** as a white solid (225.7 mg, 87% yield). ^{1}H NMR (400 MHz, $CDCl_3$): δ 7.82 (d, J = 7.2 Hz, 1H), 7.40–7.20 (m, 7H), 6.99 (s, 1H), 3.96 (s, 3H), 3.08–2.97 (m, 4H); ^{13}C NMR (100 MHz, $CDCl_3$): δ 153.8, 142.7, 136.6, 135.6, 133.1, 131.1, 129.1, 128.3, 127.6, 127.3, 126.7, 122.8, 122.3, 119.6, 109.4, 32.1, 27.8, 26.3; HRMS (DART) *m/z* calcd for $C_{18}H_{17}N_2$ $[M+H]^+$: 261.1392, found 261.1392.

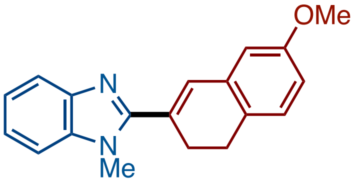

3Al: 86%

2-(7-Methoxy-3,4-dihydronaphthalen-2-yl)-1-methyl-1*H*-benzo[*d*]imidazole (3Al): Purification by flash silica-gel column chromatography (hexane/EtOAc = 2:1) afforded **3Al** as a white solid (249.2

mg, 86% yield). ^{1}H NMR (400 MHz, $CDCl_3$): δ 7.79 (dd, *J* = 6.8, 2.4 Hz, 1H), 7.40–7.27 (m, 3H), 7.13 (d, *J* = 8.0 Hz, 1H), 6.93 (s, 1H), 6.81–6.75 (m, 2H), 3.94 (s, 3H), 3.83 (s, 3H), 3.00–2.90 (m, 4H); ^{13}C NMR (100 MHz, $CDCl_3$): δ 159.4, 153.8, 142.6, 136.6, 134.0, 131.1, 129.7, 128.4, 127.7, 122.9, 122.3, 119.6, 113.4, 112.9, 109.4, 55.3, 32.1, 26.9, 26.8; HRMS (DART) *m/z* calcd for $C_{19}H_{19}N_2O$ $[M+H]^+$: 291.1497, found 291.1496.

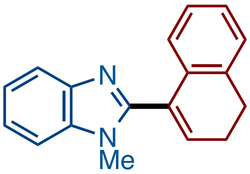

3Am: 52%

2-(3,4-Dihydronaphthalen-1-yl)-1-methyl-1*H*-benzo[*d*]imidazole (3Am): Purification by flash silica-gel column chromatography (hexane/EtOAc = 2:1) afforded **3Am** as a pale brown solid (134.7 mg, 52% yield). ^{1}H NMR (400 MHz, $CDCl_3$): δ 7.82 (d, *J* = 6.4 Hz, 1H), 7.40–7.27 (m, 3H), 7.24–7.16 (m, 2H), 7.10 (t, *J* = 7.2 Hz, 1H), 6.78 (d, *J* = 7.2 Hz, 1H), 6.53 (t, *J* = 4.8 Hz, 1H), 3.60 (s, 3H), 2.94 (t, *J* = 8.4 Hz, 2H), 2.60–2.50 (m, 2H); ^{13}C NMR (100 MHz, $CDCl_3$): δ 153.0, 143.0, 135.8, 135.4, 134.6, 132.8, 129.7, 127.8, 127.7, 126.8, 124.7, 122.6, 122.1, 119.9, 109.4, 31.0, 27.5, 23.4; HRMS (DART) *m/z* calcd for $C_{18}H_{17}N_2$ $[M+H]^+$: 261.1392, found 261.1391.

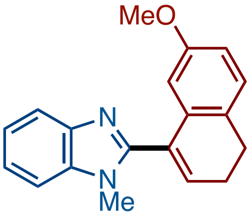

3An: 67%

2-(7-Methoxy-3,4-dihydronaphthalen-1-yl)-1-methyl-1*H*-benzo[*d*]imidazole (3An): Purification by flash silica-gel column chromatography (hexane/EtOAc = 2:1) afforded **3An** as a yellow oil (193.4 mg, 67% yield). ^{1}H NMR (400 MHz, $CDCl_3$): δ 7.81 (d, *J* = 6.4 Hz, 1H), 7.40–7.27 (m, 3H), 7.13 (d, *J* = 8.4 Hz, 1H), 6.74 (dd, *J* = 8.0, 2.8 Hz, 1H), 6.55 (t, *J* = 4.4 Hz, 1H), 6.38 (d, *J* = 2.8 Hz, 1H), 3.56 (s, 3H), 2.61 (s, 3H), 2.86 (t, *J* = 7.6 Hz, 2H), 2.56–2.48 (m, 2H); ^{13}C NMR (100 MHz, $CDCl_3$): δ 158.4, 152.7, 142.8, 135.7, 135.2, 133.7, 129.5, 128.4, 127.4, 122.5, 122.0, 119.7, 112.2, 111.0, 109.3, 55.2, 30.9, 26.5, 23.7; HRMS (DART) *m/z* calcd for $C_{19}H_{19}N_2O$ $[M+H]^+$: 291.1497, found 291.1493.

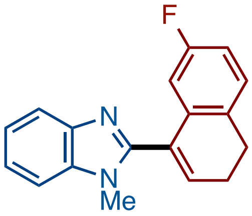

3Ao: 66%

2-(7-Fluoro-3,4-dihydronaphthalen-1-yl)-1-methyl-1*H*-benzo[*d*]imidazole (3Ao): Purification by flash silica-gel column chromatography (hexane/EtOAc = 2:1) afforded **3Ao** as a white solid (184.9 mg, 66% yield). ^{1}H NMR (400 MHz, $CDCl_3$): δ 7.82 (d, J = 6.8 Hz, 1H), 7.42–7.29 (m, 3H), 7.17 (dd, J = 8.4, 5.6 Hz, 1H), 6.88 (dt, J = 8.4, 2.8 Hz, 1H), 6.60–6.53 (m, 2H), 3.63 (s, 3H), 2.90 (t, J = 8.0 Hz, 2H), 2.59–2.51 (m, 2H); ^{13}C NMR (100 MHz, $CDCl_3$): δ 161.8 (d, $J_{C\text{–}F}$ = 241.8 Hz), 152.2, 142.8, 135.7, 134.5 (d, $J_{C\text{–}F}$ = 7.6 Hz), 130.80, 130.78, 129.1, 128.9 (d, $J_{C\text{–}F}$ = 7.6 Hz), 122.8, 122.2, 119.9, 114.1 (d, $J_{C\text{–}F}$ = 21.0 Hz), 111.8 (d, $J_{C\text{–}F}$ = 22.9 Hz), 109.5, 31.0, 26.7, 23.5; HRMS (DART) *m/z* calcd for $C_{18}H_{16}FN_2$ [M+H]$^+$: 279.1298, found 279.1293.

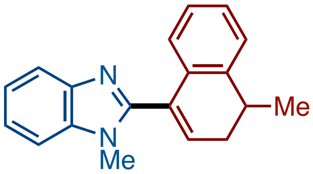

3Ap: 69%

1-Methyl-2-(4-methyl-3,4-dihydronaphthalen-1-yl)-1*H*-benzo[*d*]imidazole (3Ap): Purification by flash silica-gel column chromatography (hexane/EtOAc = 2:1) afforded **3Ap** as a yellow oil (187.9 mg, 69% yield). ^{1}H NMR (400 MHz, $CDCl_3$): δ 7.85–7.79 (m, 1H), 7.40–7.20 (m, 5H), 7.11 (dt, J = 7.8, 1.6 Hz, 1H), 6.79 (d, J = 7.8 Hz, 1H), 6.46 (t, J = 7.8 Hz, 1H), 3.60 (s, 3H), 3.08 (q, J = 6.8 Hz, 1H), 2.71 (ddd, J = 16.8, 6.8, 4.0 Hz, 1H), 2.37 (ddd, J = 16.8, 6.8, 4.0 Hz, 1H), 1.36 (d, J = 6.8 Hz, 3H); ^{13}C NMR (100 MHz, $CDCl_3$): δ 153.0, 142.9, 140.2, 135.8, 133.1, 131.9, 129.2, 128.0, 126.6, 126.4, 124.8, 122.5, 122.0, 119.8, 109.3, 31.6, 31.2, 30.9, 20.0; HRMS (DART) *m/z* calcd for $C_{19}H_{19}N_2$ [M+H]$^+$: 275.1548, found 275.1545

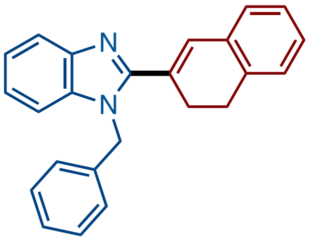

3Bk: 87%

1-Benzyl-2-(3,4-dihydronaphthalen-2-yl)-1*H*-benzo[*d*]imidazole (3Bk): Purification by flash silica-gel column chromatography (hexane/EtOAc = 3:1) afforded **3Bk** as a white solid (293.1 mg,

87% yield). ^{1}H NMR (400 MHz, $CDCl_3$): δ 7.84 (d, *J* = 7.6 Hz, 1H), 7.40–7.22 (m, 7H), 7.20–7.10 (m, 4H), 6.89 (d, *J* = 7.6 Hz, 1H), 6.76 (s, 1H), 5.54 (s, 2H), 3.00–2.93 (m, 4H); ^{13}C NMR (100 MHz, $CDCl_3$): δ 154.3, 143.0, 136.7, 136.5, 135.6, 133.1, 130.8, 129.1, 128.8, 128.4, 127.8, 127.6, 127.3, 126.6, 125.9, 123.2, 122.6, 119.8, 110.1, 48.7, 27.8, 26.4; HRMS (DART) *m/z* calcd for $C_{24}H_{21}N_2$ $[M+H]^+$: 337.1705, found 337.1705.

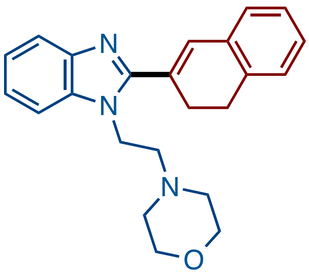

3Dk: 80%

4-(2-(2-(3,4-Dihydronaphthalen-2-yl)-1*H*-benzo[*d*]imidazol-1-yl)ethyl)morpholine (3Dk): Purification by flash silica-gel column chromatography (CH_2Cl_2/MeOH = 24:1) afforded **3Dk** as a pale yellow oil (288.6 mg, 80% yield). ^{1}H NMR (400 MHz, $CDCl_3$): δ 7.82–7.76 (m, 1H), 7.42–7.40 (m, 1H), 7.33–7.28 (m, 2H), 7.27–7.21 (m, 3H), 7.19–7.16 (m, 1H), 7.07 (s, 1H), 4.46 (t, *J* = 7.6 Hz, 2H), 3.68 (t, *J* = 4.8 Hz, 4H), 3.08–2.94 (m, 4H), 2.85 (t, *J* = 7.6 Hz, 2H), 2.51 (t, *J* = 4.8 Hz, 4H); ^{13}C NMR (100 MHz, $CDCl_3$): δ 153.9, 142.9, 135.8, 135.5, 133.1, 130.7, 129.2, 128.4, 127.7, 127.1, 126.7, 122.8, 122.4, 119.8, 109.7, 66.8, 57.5, 54.0, 42.9, 27.8, 26.7; HRMS (DART) *m/z* calcd for $C_{23}H_{26}N_3O$ $[M+H]^+$: 360.2076, found 360.2077.

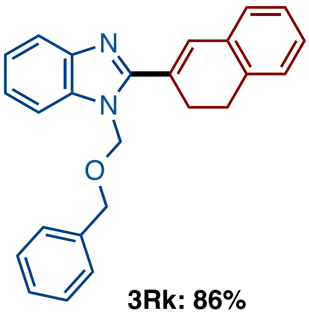

3Rk: 86%

1-((Benzyloxy)methyl)-2-(3,4-dihydronaphthalen-2-yl)-1*H*-benzo[*d*]imidazole (3Rk): Purification by flash silica-gel column chromatography (hexane/EtOAc = 4:1) afforded **3Rk** as a yellow oil (314.4 mg, 86% yield). ^{1}H NMR (400 MHz, $CDCl_3$): δ 7.87–7.83 (m, 1H), 7.45–7.30 (m, 9H), 7.25–7.18 (m, 3H), 7.15 (d, *J* = 7.6 Hz, 1H), 5.68 (s, 2H), 4.70 (s, 2H), 3.03–2.99 (m, 4H); ^{13}C NMR (100 MHz, $CDCl_3$): δ 154.4, 142.4, 136.5, 136.1, 135.6, 133.1, 131.9, 131.8, 128.4, 128.3, 128.1, 127.9, 127.5, 127.4, 126.5, 123.3, 122.8, 119.6, 109.6, 72.9, 70.3, 27.6, 26.1; HRMS (DART) *m/z* calcd for $C_{25}H_{23}N_2O$ $[M+H]^+$: 367.1810, found 367.1817.

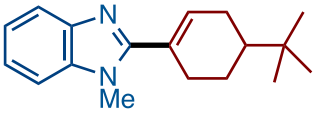

3Aq: 75%

2-(4-(*tert*-Butyl)cyclohex-1-en-1-yl)-1-methyl-1*H*-benzo[*d*]imidazole (3Aq): Purification by Isolera® (hexane/EtOAc = 10:1 to EtOAc) and GPC afforded **3Aq** as a white solid (80.4 mg, 75% yield). ^{1}H NMR (400 MHz, $CDCl_3$): δ 7.73 (d, *J* = 8.8 Hz, 1H), 7.30–7.19 (m, 3H), 6.18 (dd, *J* = 2.8, 2.4 Hz, 1H), 3.76 (s, 3H), 2.77 (d, *J* = 15.2 Hz, 1H), 2.56–2.28 (m, 2H), 2.10–1.97 (m, 2H), 1.50–1.28 (m, 2H), 0.93 (s, 9H); ^{13}C NMR (100 MHz, $CDCl_3$): δ 155.0, 142.5, 136.1, 133.4, 128.6, 122.2, 121.9, 119.4, 109.2, 43.5, 32.2, 31.4, 29.3, 27.4, 27.1, 23.9; HRMS (DART) *m/z* calcd for $C_{18}H_{25}N_2$ $[M+H]^+$: 269.2018, found 269.2018.

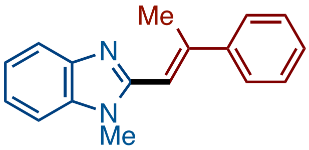

3Ar: 29%

(*E*)-1-Methyl-2-(2-phenylprop-1-en-1-yl)-1*H*-benzo[*d*]imidazole (3Ar): Purification by flash silica-gel column chromatography (hexane/Et_2O = 3:1) afforded **3Ar** as a pale yellow solid (72.8 mg, 29% yield). ^{1}H NMR (400 MHz, $CDCl_3$): δ 7.83–7.78 (m, 1H), 7.58 (d, *J* = 8.0 Hz, 2H), 7.45–7.27 (m, 6H), 6.71 (s, 1H), 3.81 (s, 3H), 2.73 (s, 3H); ^{13}C NMR (100 MHz, $CDCl_3$): δ 151.0, 147.4, 143.1, 143.0, 135.0, 128.4, 128.2, 126.1, 122.4, 122.0, 119.5, 112.7, 108.9, 29.8, 18.5; HRMS (DART) *m/z* calcd for $C_{17}H_{17}N_2$ $[M+H]^+$: 249.1392, found 249.1391.

6. Ni-Catalyzed C–H Coupling of Thiazoles and Oxazoles

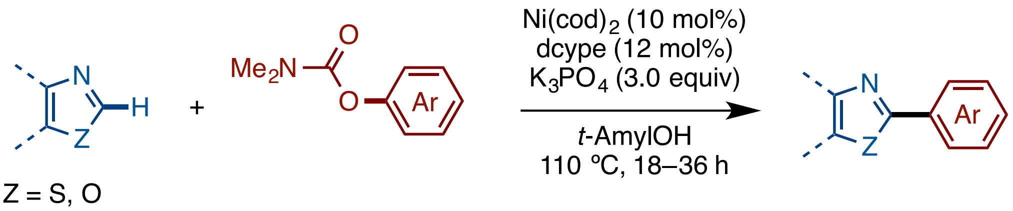

General Procedure: A 20-mL glass vessel equipped with a J. Young® O-ring tap containing a magnetic stirring bar and K_3PO_4 (255.0 mg, 1.20 mmol, 3.0 equiv) was dried with a heatgun for 3 min *in vacuo* and filled with N_2 after cooling to room temperature. To this vessel were added 1,3-azole **5** (0.40 mmol, 1.0 equiv) and aryl carbamate **2** (0.60 mmol, 1.5 equiv). Then the vessel was introduced into an argon-atmosphere glovebox. To the reaction vessel were added $Ni(cod)_2$ (11.0 mg, 0.040 mmol, 10 mol%) and 1,2-bis(dicyclohexylphosphino)ethane (dcype: 20.6 mg, 0.050 mmol, 12 mol%). The

vessel was taken out of the glovebox, then dry *t*-AmylOH (1.6 mL) was added under a stream of N_2. The vessel was sealed with an O-ring tap and then heated at 110 °C for 18–36 h in an 8-well reaction block with stirring. After cooling the reaction mixture to room temperature, the mixture was passed through a silica gel pad with EtOAc as an eluent. The filtrate was concentrated and the residue was subjected to PTLC or flash silica-gel column chromatography to afford 2-arylated azole **6**.

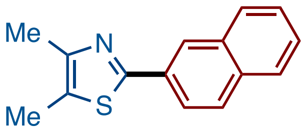

6Ab: 90%

4,5-Dimethyl-2-(naphthalen-2-yl)thiazole (6Ab): Purification by PTLC (hexane/EtOAc = 20:1) afforded **6Ab** as a pale yellow solid (86.0 mg, 90% yield). ^{1}H NMR (400 MHz, $CDCl_3$): δ 8.33 (s, 1H), 7.97 (dd, *J* = 8.8, 2.0 Hz, 1H), 7.91–7.78 (m, 3H), 7.51–7.43 (m, 2H), 2.41 (s, 3H), 2.39 (s, 3H); ^{13}C NMR (100 MHz, $CDCl_3$): δ 163.3, 149.4, 133.7, 133.3, 131.2, 128.50, 128.46, 127.7, 126.7, 126.5, 125.1, 123.8, 14.8, 11.5 (one peak is overlapping); HRMS (DART) *m/z* calcd for $C_{15}H_{14}NS$ $[M+H]^+$: 240.0847, found 240.0846.

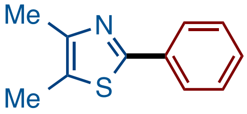

6Aa: 91%

4,5-Dimethyl-2-phenylthiazole (6Aa)[19]**:** Purification by PTLC (hexane/EtOAc = 10:1) afforded **6Aa** as a colorless liquid (69.1 mg, 91% yield). ^{1}H NMR (400 MHz, $CDCl_3$): δ 7.86 (dd, *J* = 8.0, 2.4 Hz, 2H), 7.42–7.33 (m, 3H), 2.38 (s, 6H); ^{13}C NMR (100 MHz, $CDCl_3$): δ 163.3, 149.3, 133.9, 129.3, 128.8, 126.5, 126.1, 14.8, 11.4; HRMS (DART) *m/z* calcd for $C_{11}H_{12}NS$ $[M+H]^+$: 190.0690, found 190.0686.

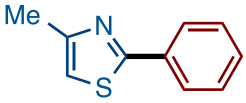

6Ba: 54%

4-Methyl-2-phenylthiazole (6Ba)[22]**:** Purification by Isolera® (hexane/EtOAc = 100:1 to 10:1) afforded **6Ba** as a yellow liquid (37.8 mg, 54% yield). ^{1}H NMR (400 MHz, $CDCl_3$): δ 7.92 (d, *J* = 7.6 Hz, 2H), 7.45–7.35 (m, 3H), 6.85 (s, 1H), 2.50 (s, 3H); ^{13}C NMR (100 MHz, $CDCl_3$): δ 167.5, 153.8,

22 T. Yamamoto, K. Muto, M. Komiyama, J. Canivet, J. Yamaguchi and K. Itami, *Chem. Eur. J.*, 2011, **17**, 10113.

133.8, 129.7, 128.8, 126.4, 113.4, 17.2; HRMS (DART) *m/z* calcd for $C_{10}H_{10}NS$ $[M+H]^+$: 176.0534, found 176.0535.

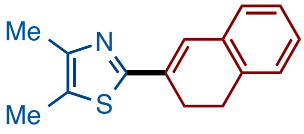

6Ak: 59%

2-(3,4-Dihydronaphthalen-2-yl)-4,5-dimethylthiazole (6Ak): Reaction was performed by using $Ni(OTf)_2$ (35.7 mg, 0.10 mmol, 10 mol%) and 3,4-bis(dicyclohexylphosphino)thiophene (dcypt: 57.2 mg, 0.12 mmol, 12 mol%) instead of $Ni(cod)_2$/dcype. Purification by flash silica-gel column chromatography (hexane/Et_2O = 6:1) afforded **6Ak** as a yellow solid (141.8 mg, 59% yield). ^1H NMR (400 MHz, $CDCl_3$): δ 7.19–7.14 (m, 5H), 2.97–2.83 (m, 4H), 2.38 (s, 3H), 2.36 (s, 3H); ^{13}C NMR (100 MHz, $CDCl_3$): δ 164.3, 148.9, 135.7, 133.7, 132.7, 127.7, 127.4, 127.1, 126.6, 126.2, 125.9, 27.8, 25.0, 14.8, 11.5; HRMS (DART) *m/z* calcd for $C_{15}H_{16}NS$ $[M+H]^+$: 242.1003, found 242.1002.

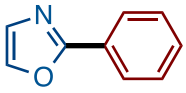

6Ca: 56%

2-Phenyloxazole (6Ca)[22]: Purification by PTLC (hexane/EtOAc = 20:1) afforded **6Ca** as a colorless liquid (32.6 mg, 56% yield). ^1H NMR (400 MHz, $CDCl_3$): δ 8.05 (d, J = 7.6 Hz, 2H), 7.69 (d, J = 2.0 Hz, 1H), 7.50–7.40 (m, 3H), 7.23 (d, J = 2.0 Hz, 1H); ^{13}C NMR (100 MHz, $CDCl_3$): δ 161.9, 138.5, 130.3, 128.7, 128.3, 127.4, 126.3; HRMS (DART) *m/z* calcd for C_9H_8NO $[M+H]^+$: 146.0606, found 146.0603.

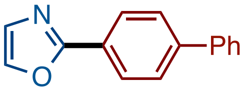

6Ci: 63%

2-([1,1'-Biphenyl]-4-yl)oxazole (6Ci): Purification by PTLC (hexane/EtOAc = 10:1) afforded **6Ci** as a white solid (55.8 mg, 63% yield). ^1H NMR (400 MHz, $CDCl_3$): δ 8.11 (d, J = 8.4 Hz, 2H), 7.69 (s, 1H), 7.66 (d, J = 8.4 Hz, 2H), 7.61 (d, J = 8.4 Hz, 2H), 7.44 (t, J = 8.4 Hz, 2H), 7.36 (t, J = 8.4 Hz, 1H), 7.24 (s, 1H); ^{13}C NMR (100 MHz, $CDCl_3$): δ 161.8, 142.9, 140.1, 138.5, 128.8, 128.4, 127.8, 127.4, 127.0, 126.7, 126.3; HRMS (DART) *m/z* calcd for $C_{15}H_{12}NO$ $[M+H]^+$: 222.0919, found 222.0920.

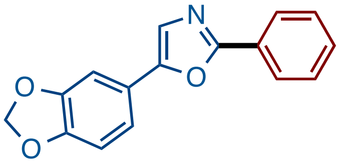

6Da: 74%

4,5-Dimethyl-2-phenylthiazole (6Da)[23]: Reaction was performed by using Ni(OTf)$_2$ (14.2 mg, 0.040 mmol, 10 mol%) instead of Ni(cod)$_2$. Purification by flash silica-gel column chromatography (hexane/EtOAc = 10:1 to 4:1) afforded **6Da** as a white solid (78.9 mg, 74% yield). ^{1}H NMR (400 MHz, CDCl$_3$): δ 8.05 (d, *J* = 8.4 Hz, 2H), 7.49–7.39 (m, 3H), 7.28 (s, 1H), 7.20 (d, *J* = 8.4 Hz, 1H), 7.14 (s, 1H), 6.85 (d, *J* = 8.4 Hz, 1H), 5.97 (s, 2H); ^{13}C NMR (100 MHz, CDCl$_3$): δ 160.5, 151.0, 148.1, 147.8, 130.1, 128.7, 127.4, 126.1, 122.3, 122,1, 118.2, 108.7, 104.7, 101.3; HRMS (DART) *m/z* calcd for $C_{16}H_{12}NO_3$ [M+H]$^+$: 266.0817, found 266.0813.

[23] K. Muto, J. Yamaguchi and K. Itami, *J. Am. Chem. Soc.*, 2012, **134**, 169.

7. ^{1}H NMR and ^{13}C NMR Spectra

^{1}H NMR of **1P** (400 MHz, $CDCl_3$)

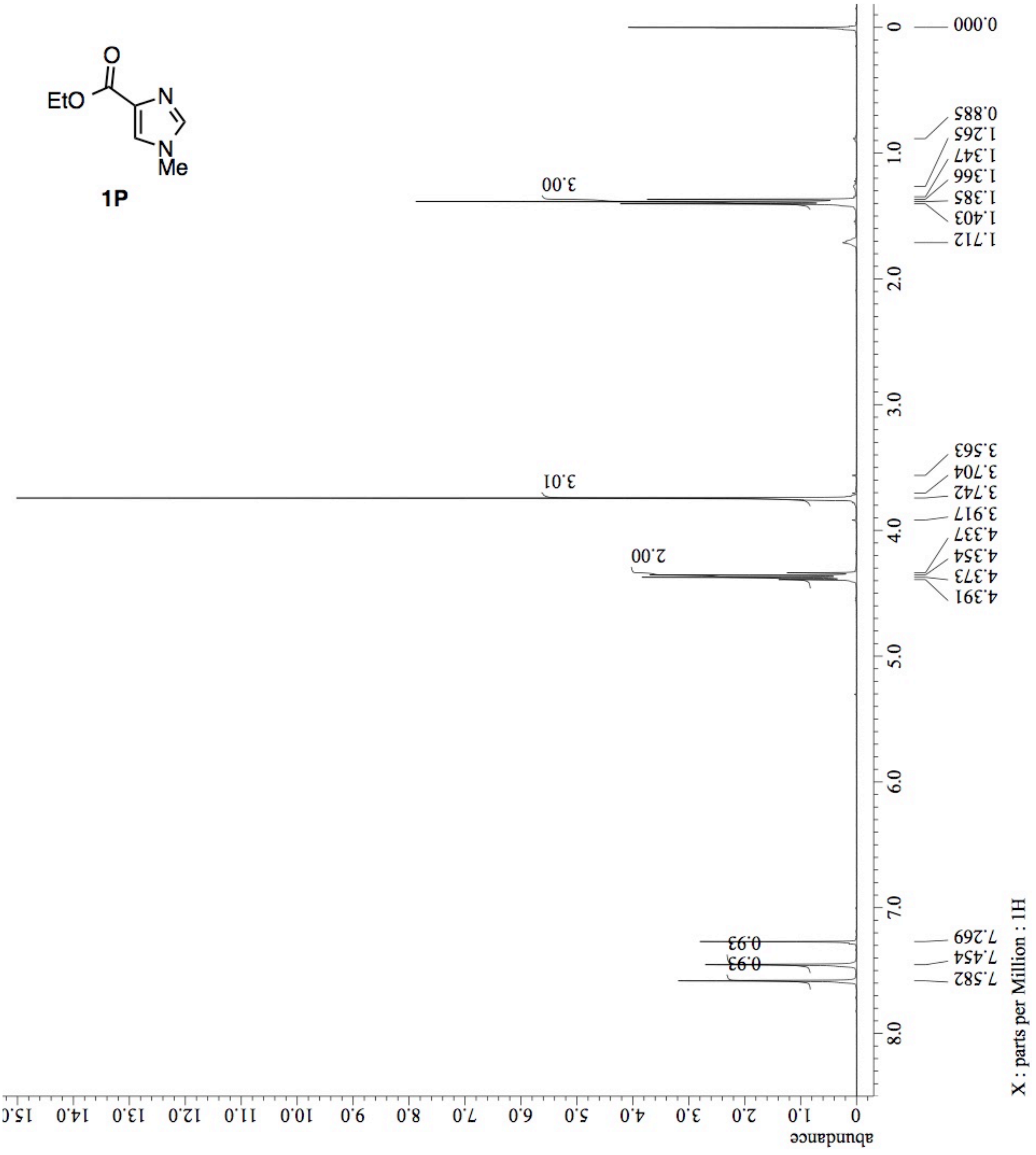

1P

^{13}C NMR of **1P** (100 MHz, $CDCl_3$)

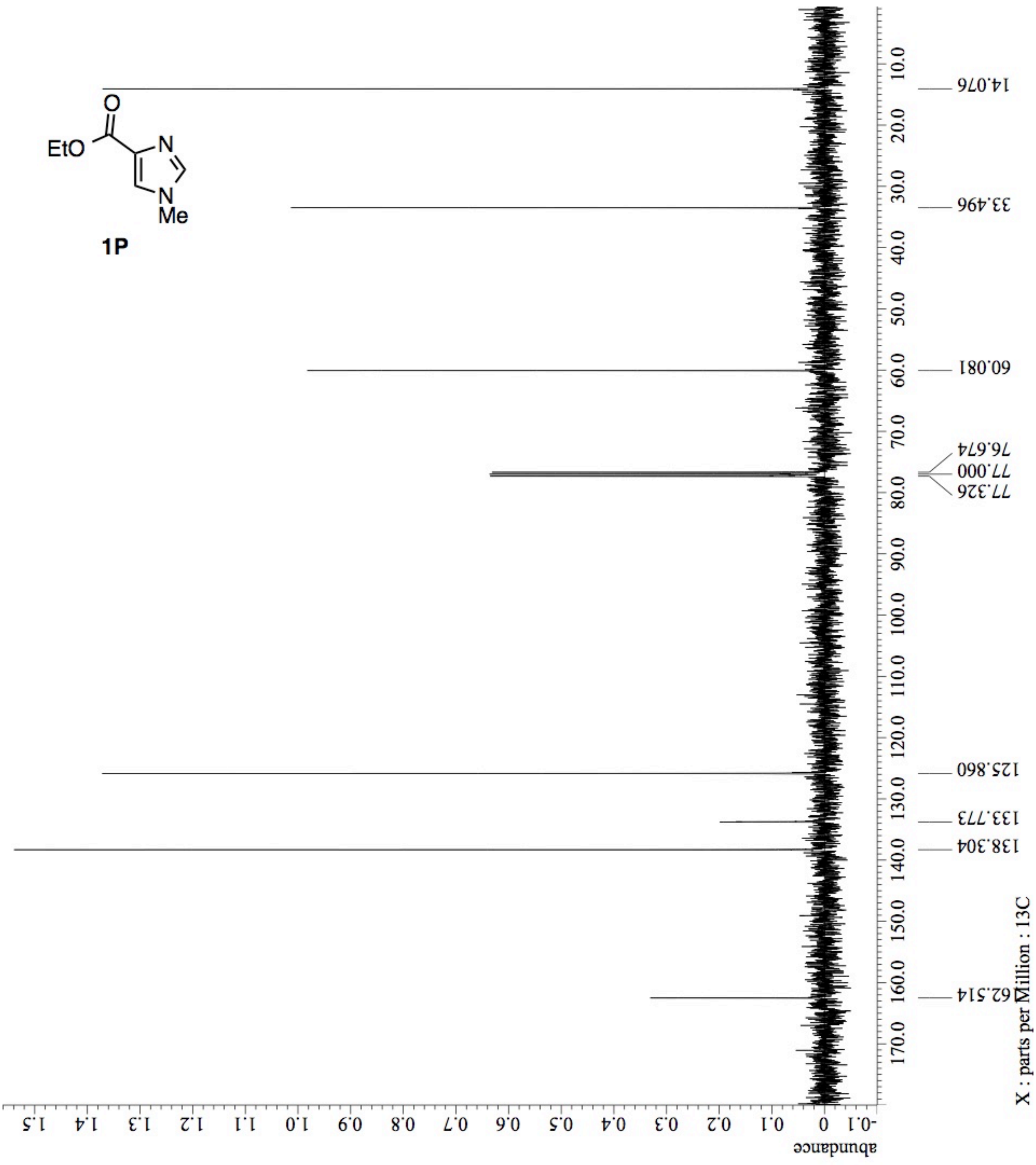

^{1}H NMR of **2c** (400 MHz, $CDCl_3$)

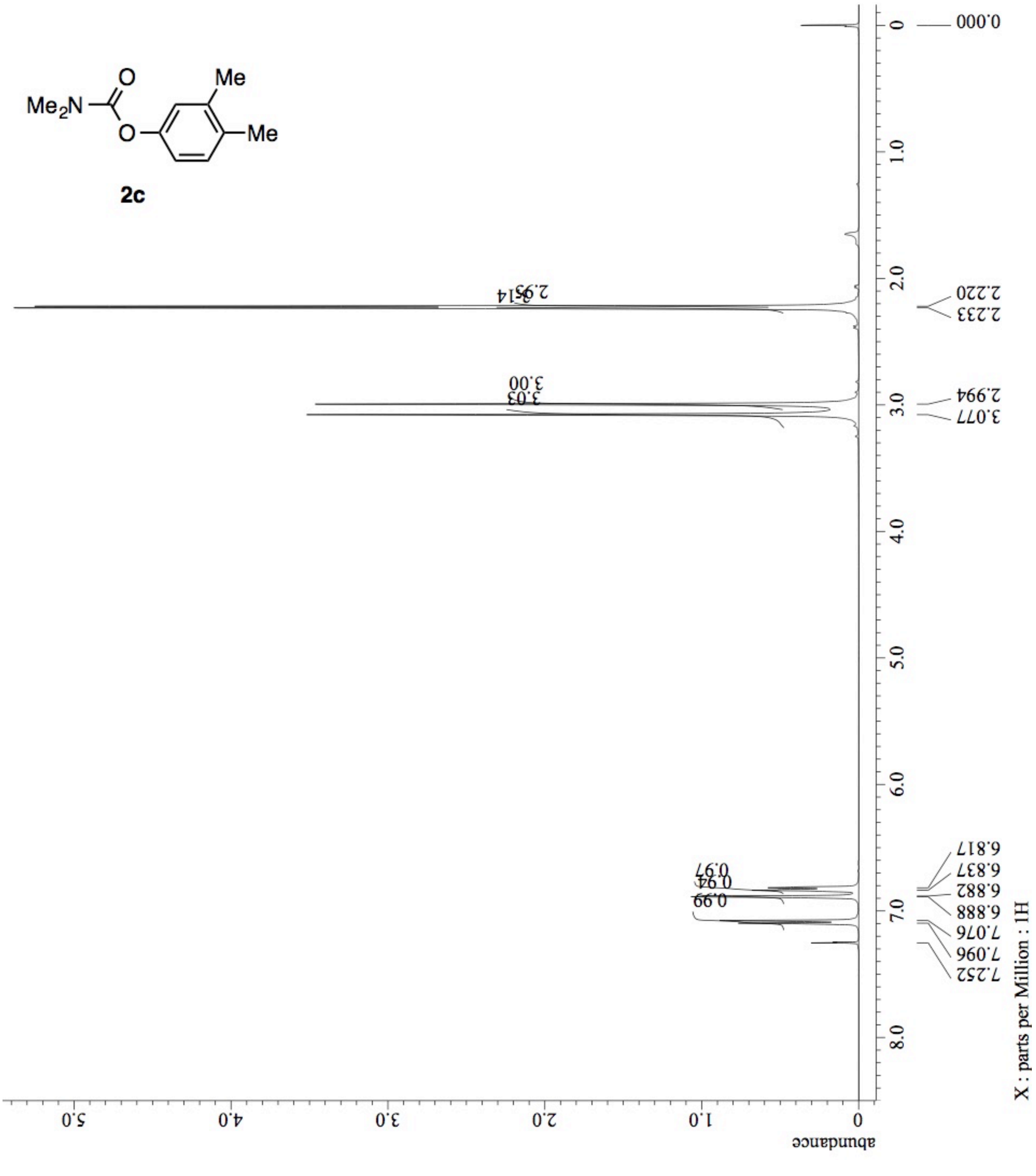

^{13}C NMR of **2c** (100 MHz, $CDCl_3$)

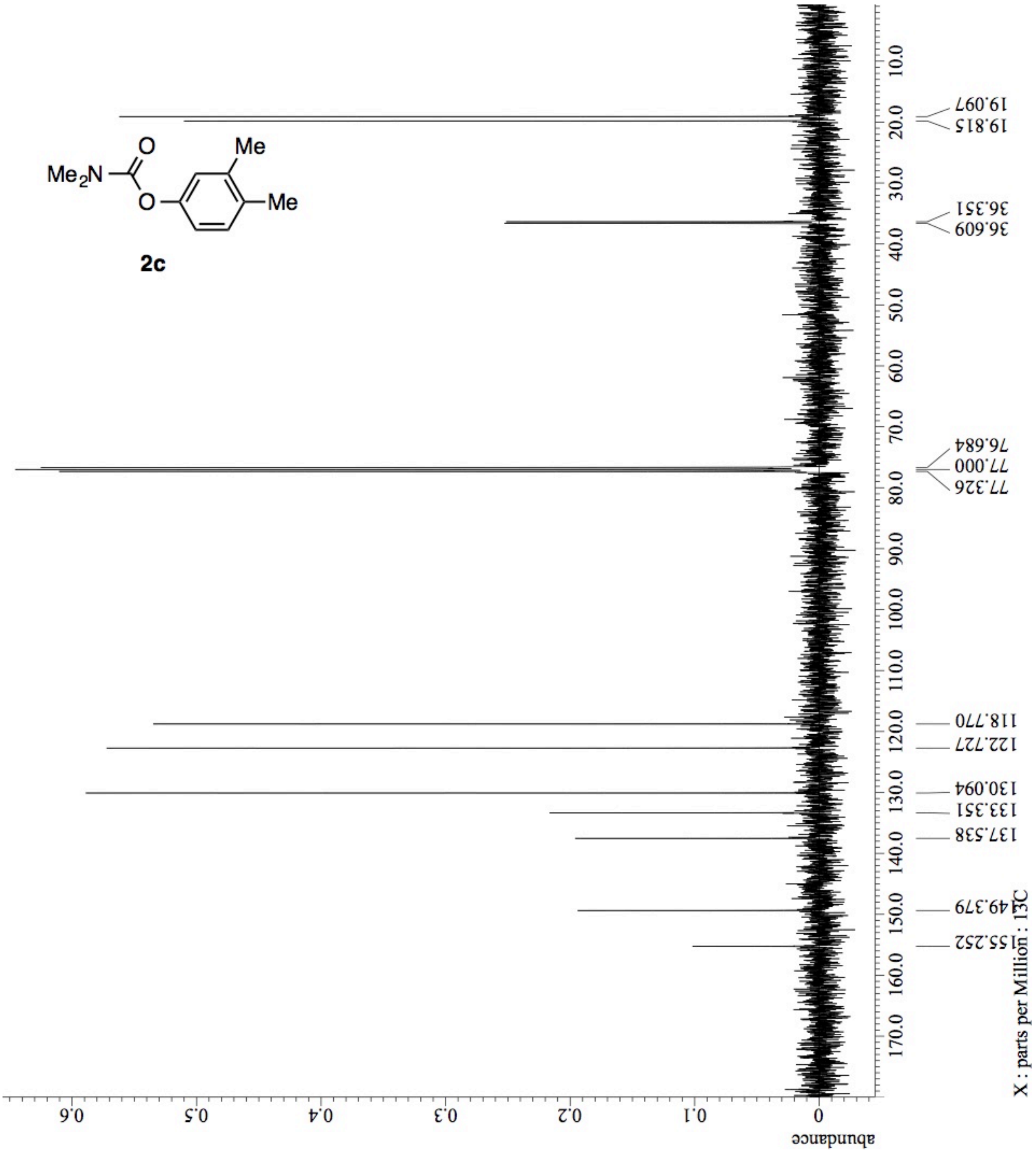

^{1}H NMR of **2d** (400 MHz, $CDCl_3$)

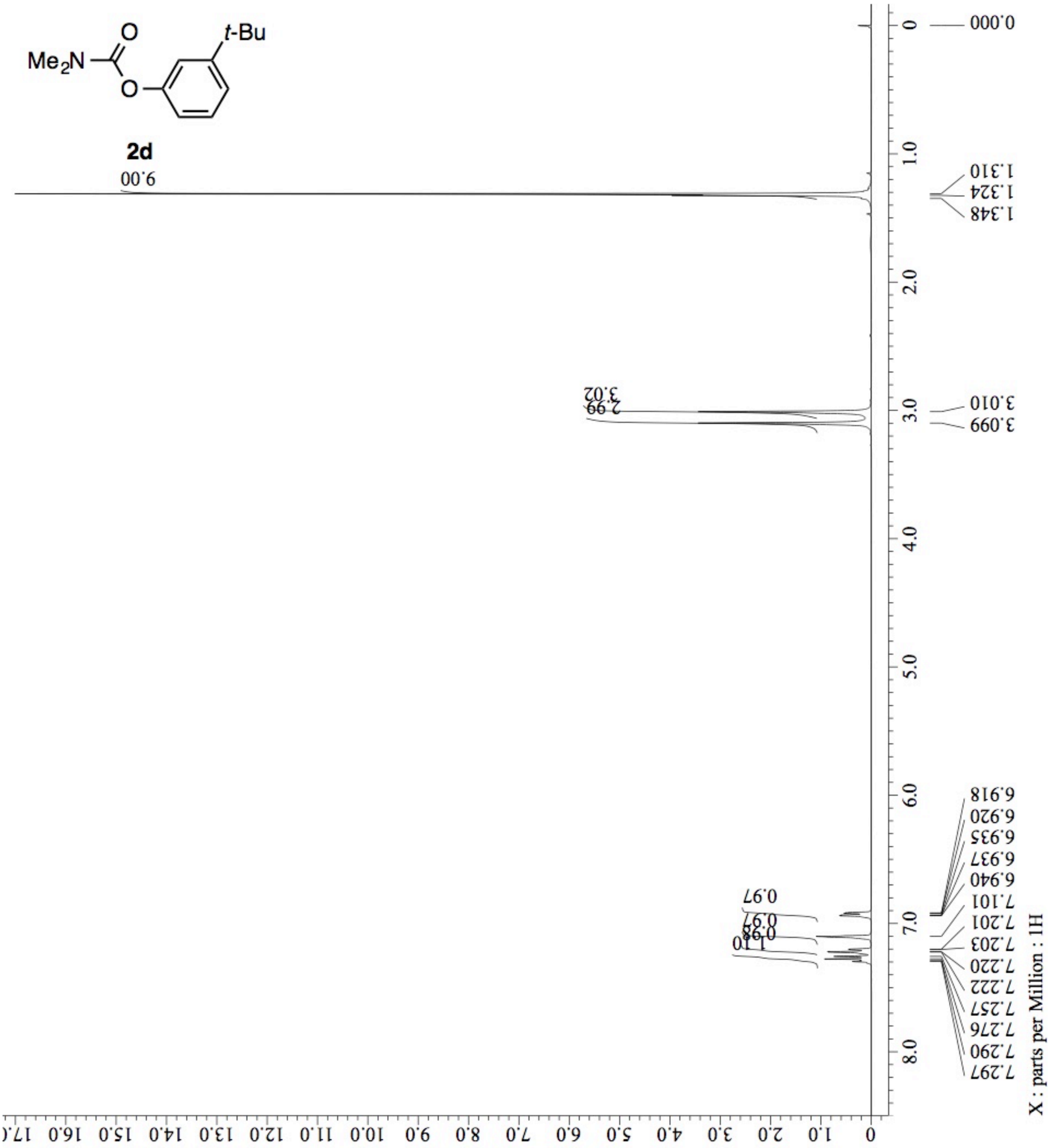

^{13}C NMR of **2d** (100 MHz, $CDCl_3$)

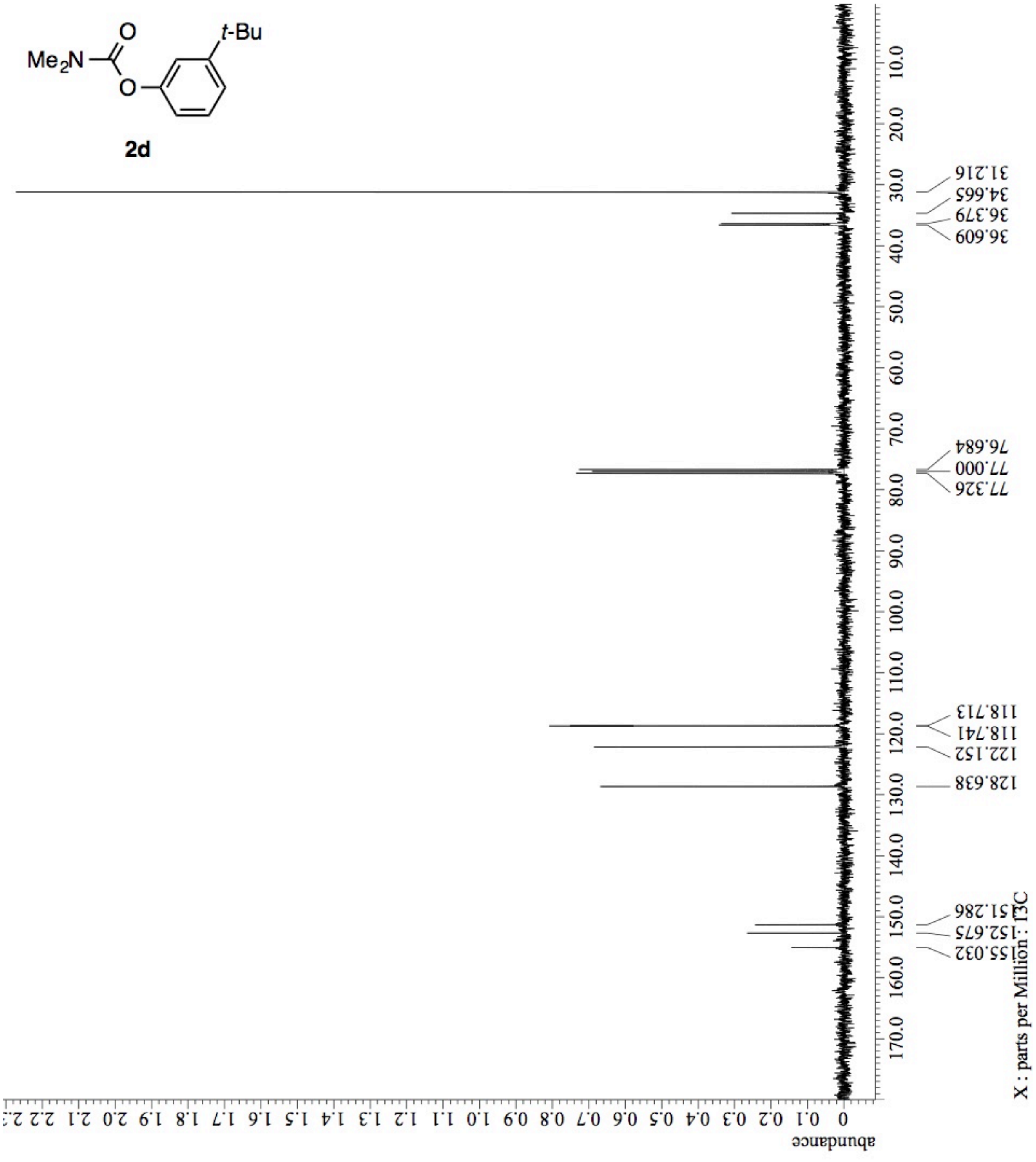

^{1}H NMR of **2g** (400 MHz, $CDCl_3$)

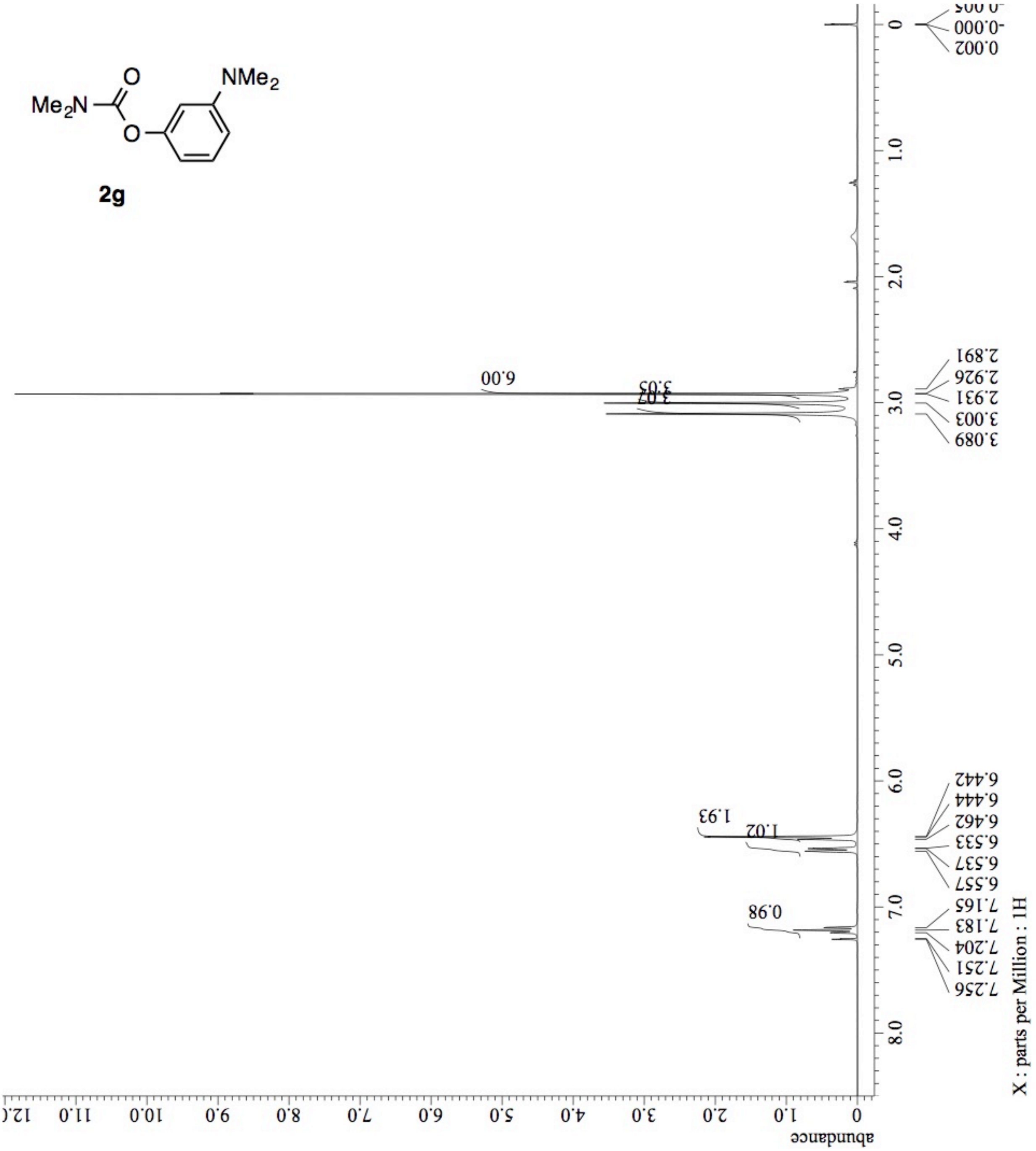

^{13}C NMR of **2g** (100 MHz, $CDCl_3$)

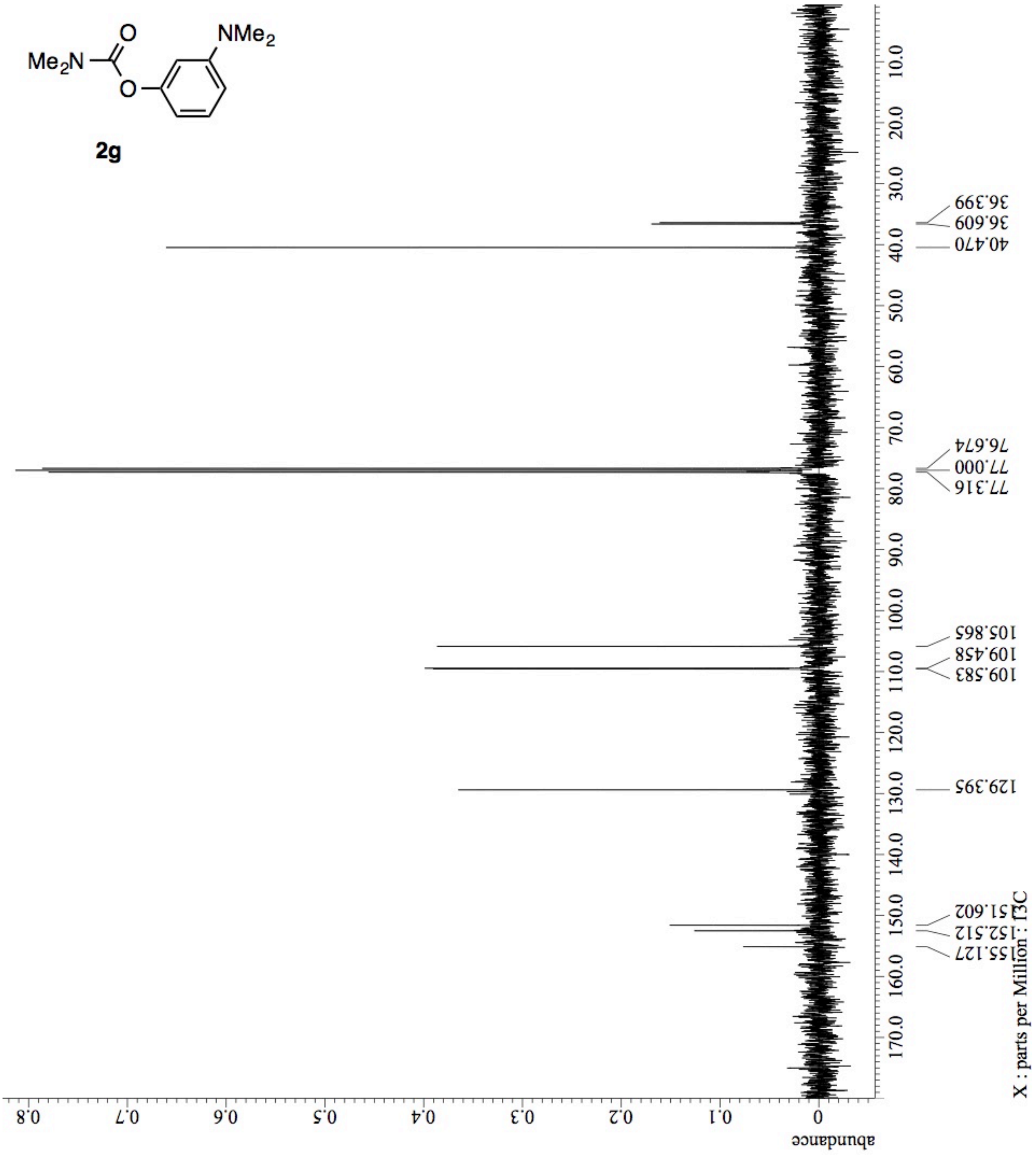

^{1}H NMR of **2h** (400 MHz, $CDCl_3$)

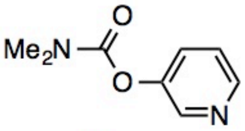

2h

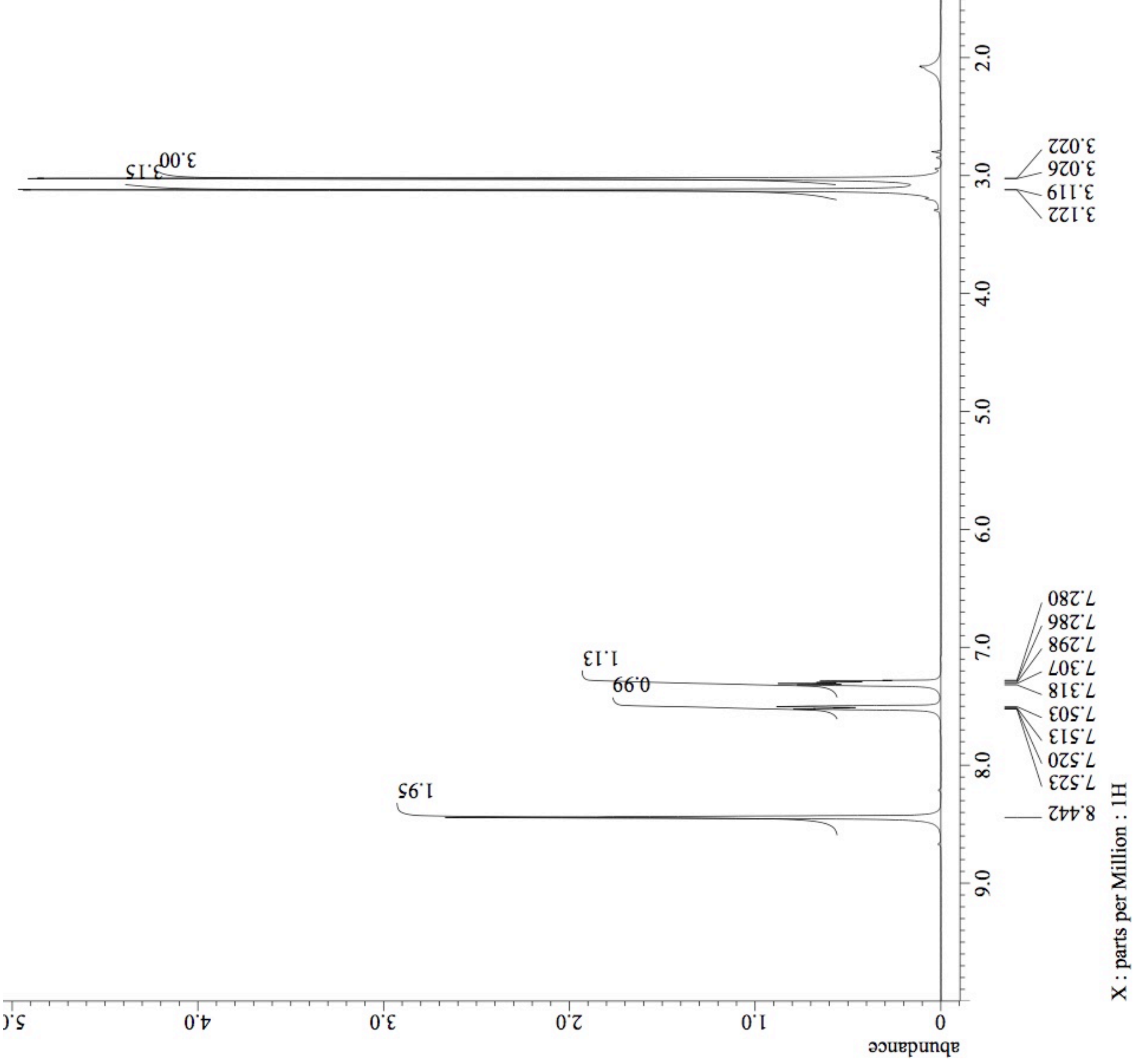

^{13}C NMR of **2h** (100 MHz, $CDCl_3$)

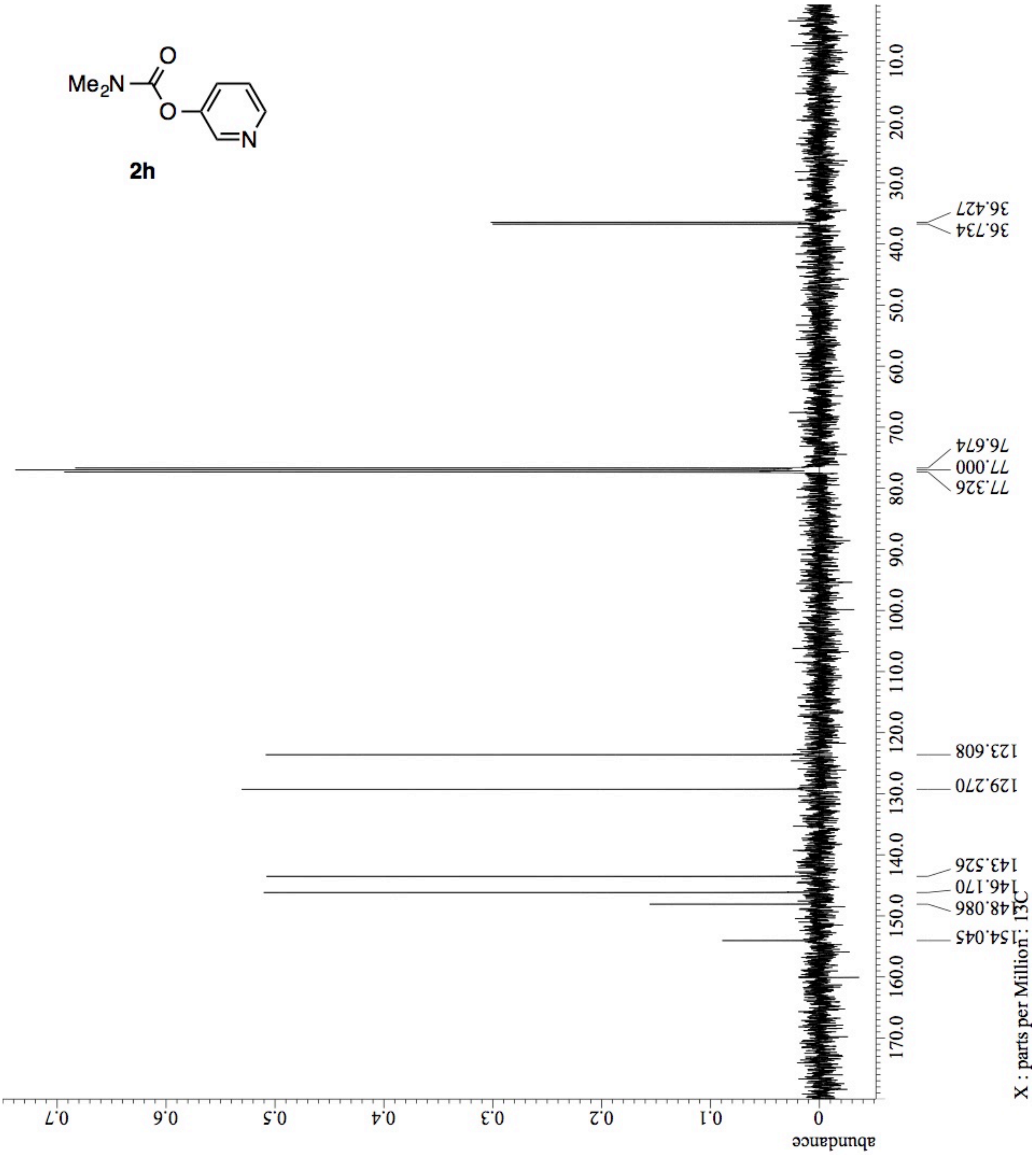

^{1}H NMR of **2l** (400 MHz, $CDCl_3$)

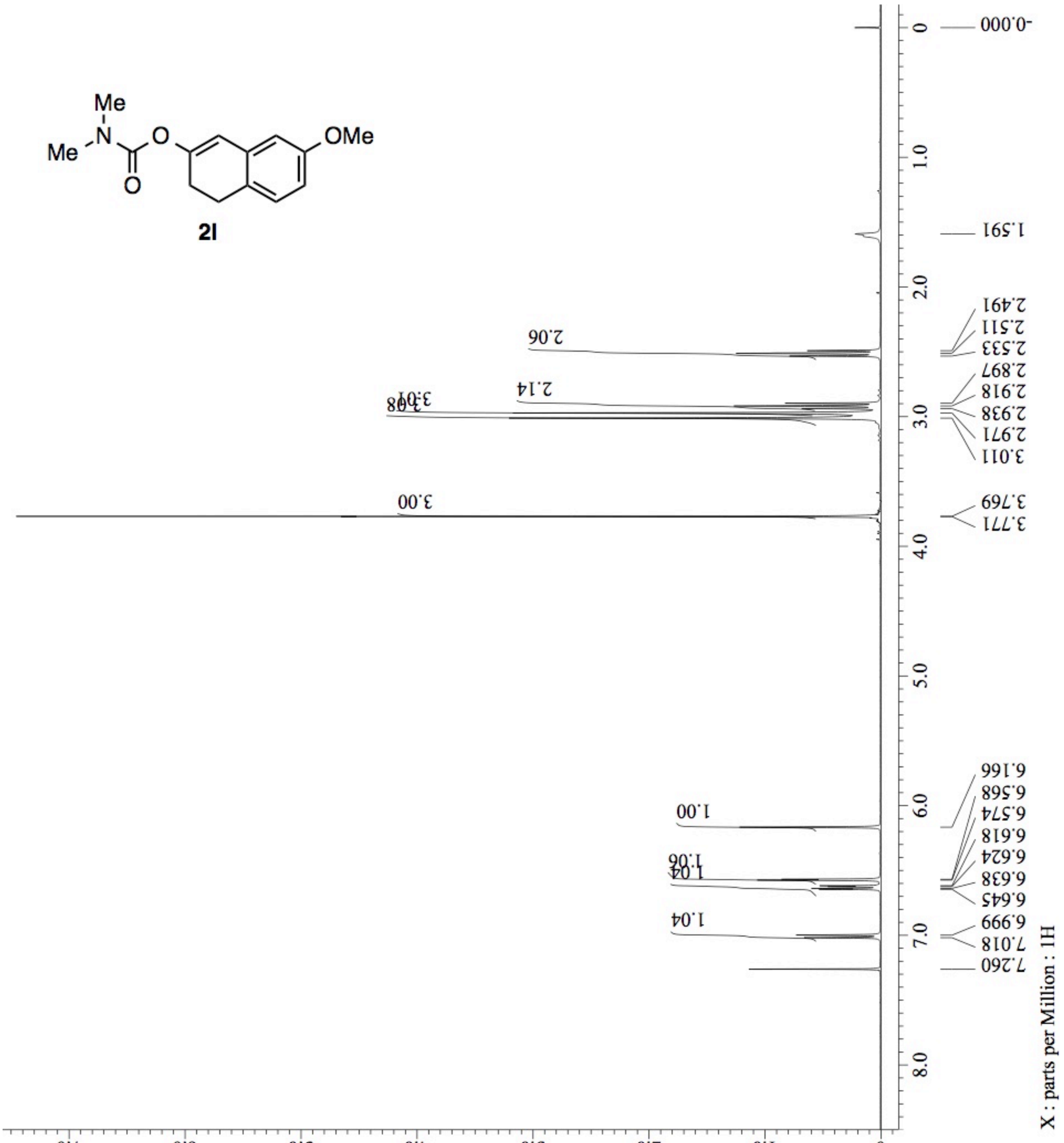

^{13}C NMR of **2l** (100 MHz, $CDCl_3$)

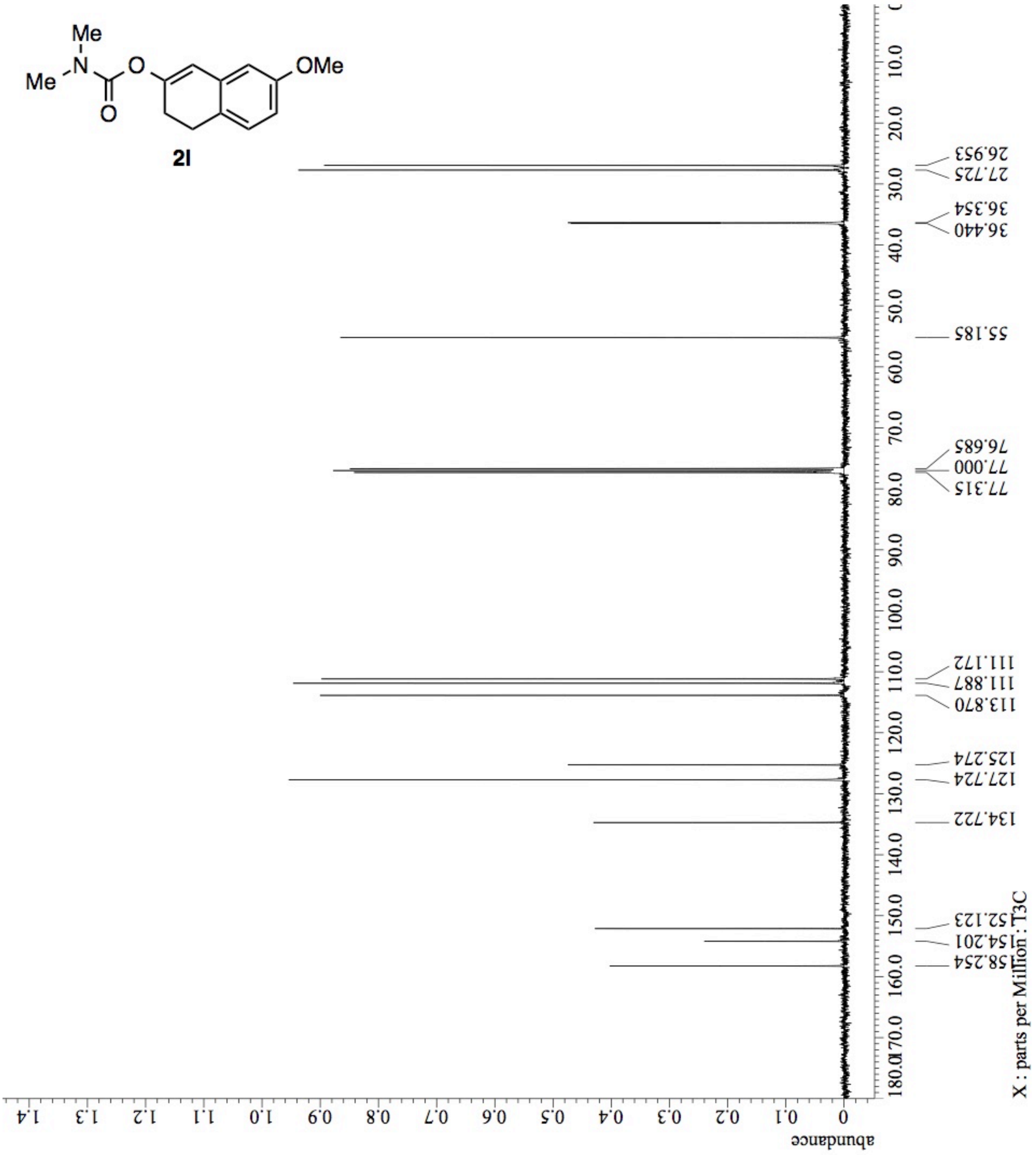

^{1}H NMR of **2n** (400 MHz, $CDCl_3$)

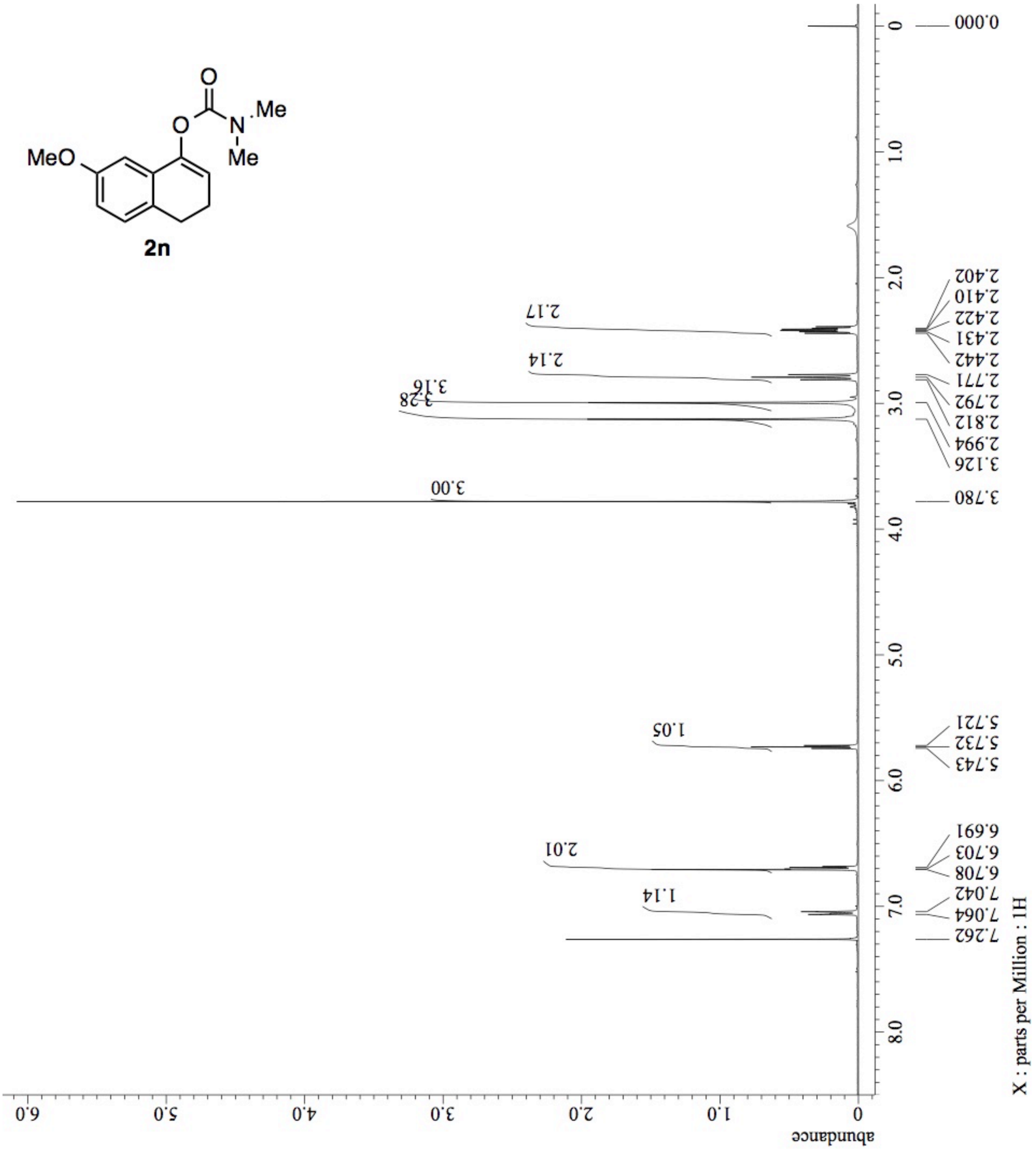

2n

^{13}C NMR of **2n** (100 MHz, $CDCl_3$)

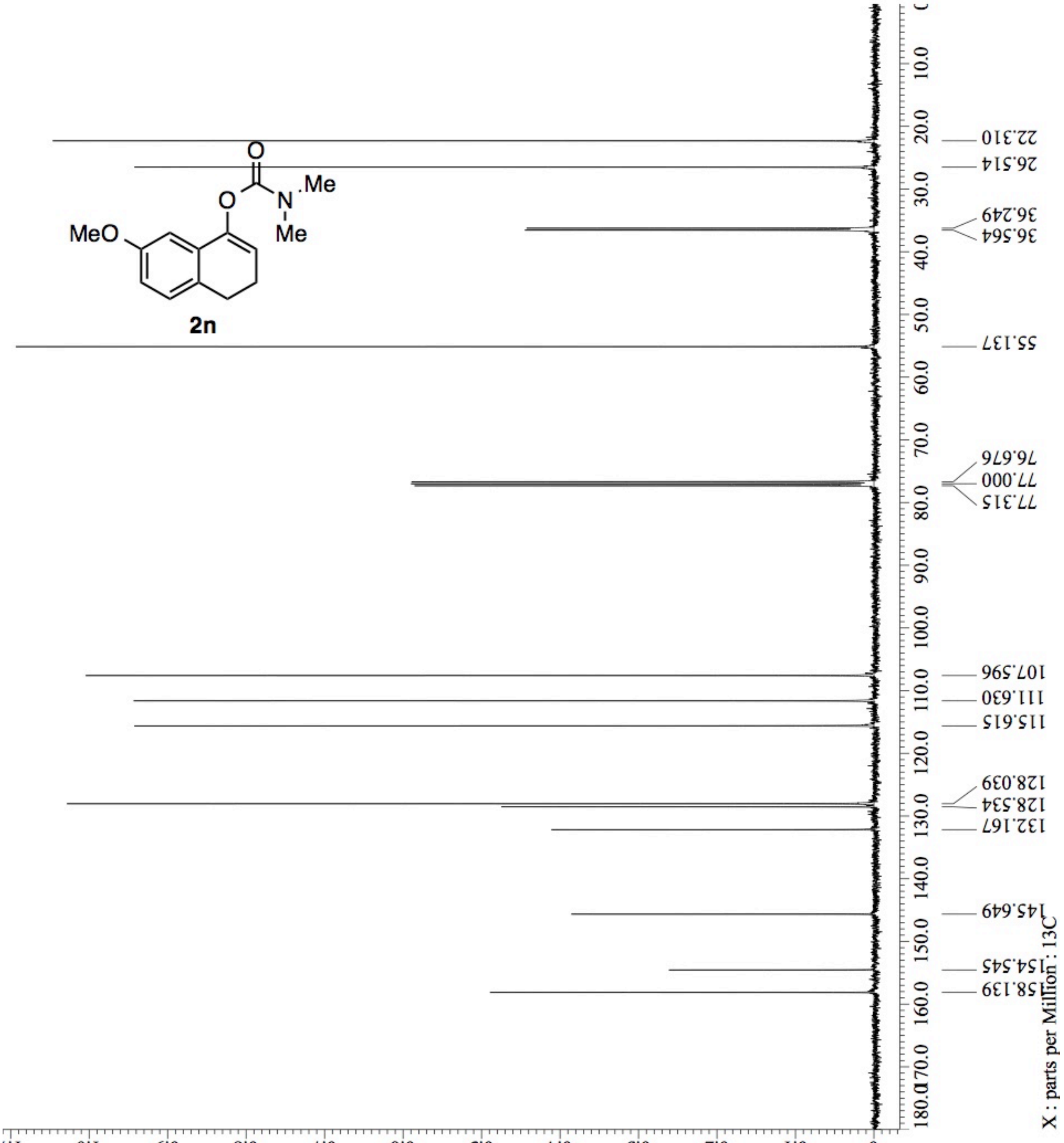

^{1}H NMR of **2o** (400 MHz, $CDCl_3$)

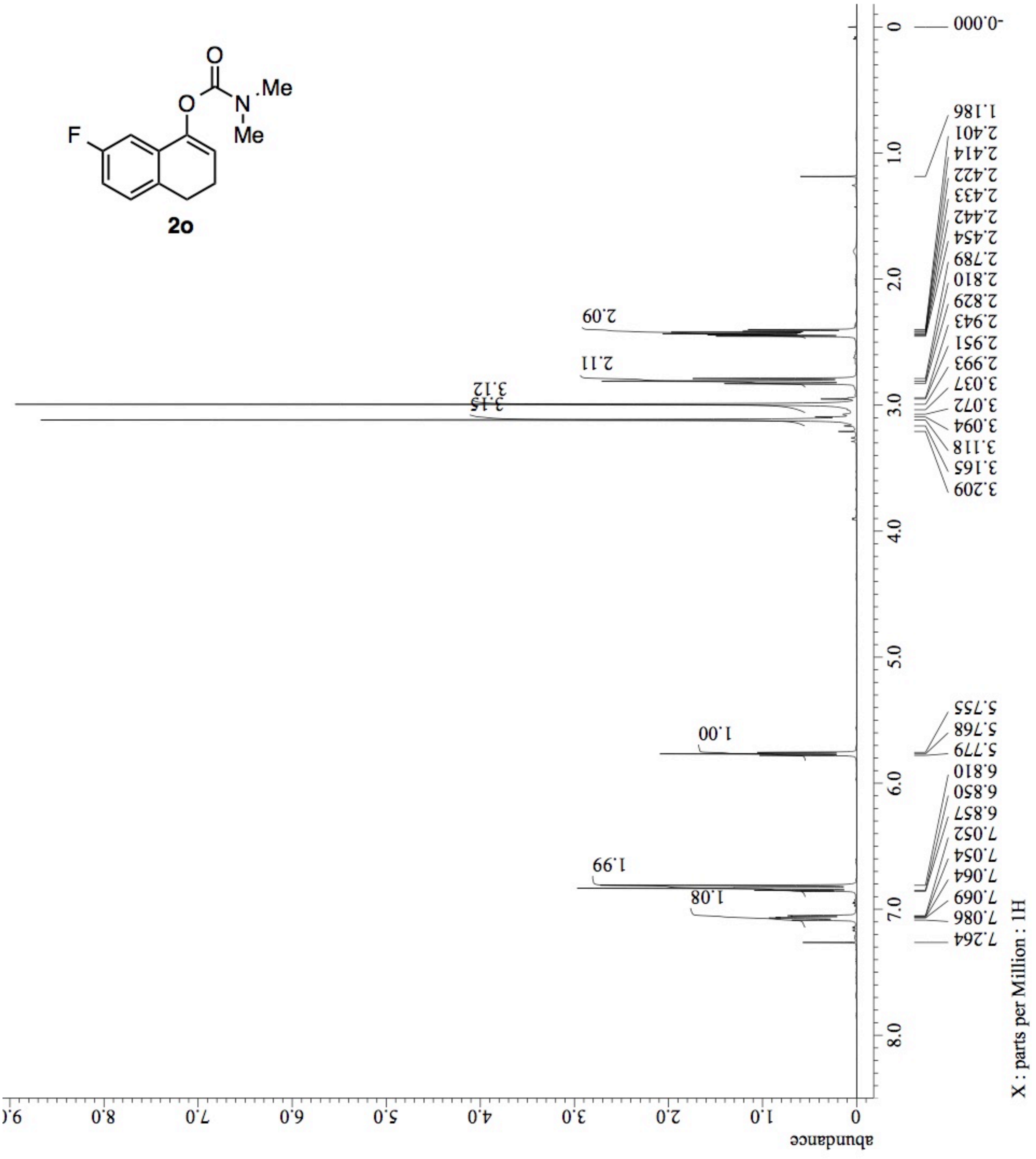

^{13}C NMR of **2o** (100 MHz, $CDCl_3$)

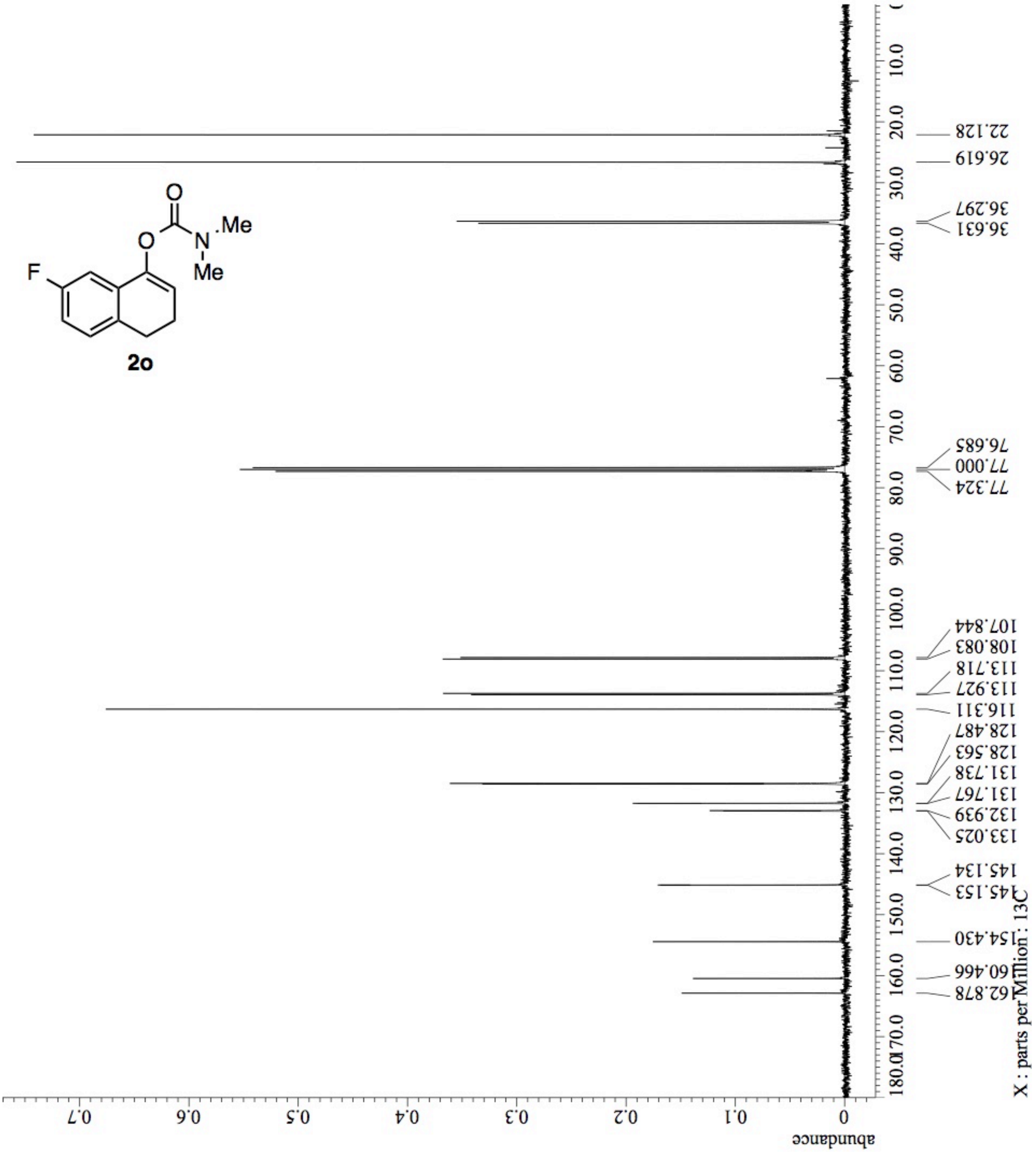

^{1}H NMR of **2p** (400 MHz, $CDCl_3$)

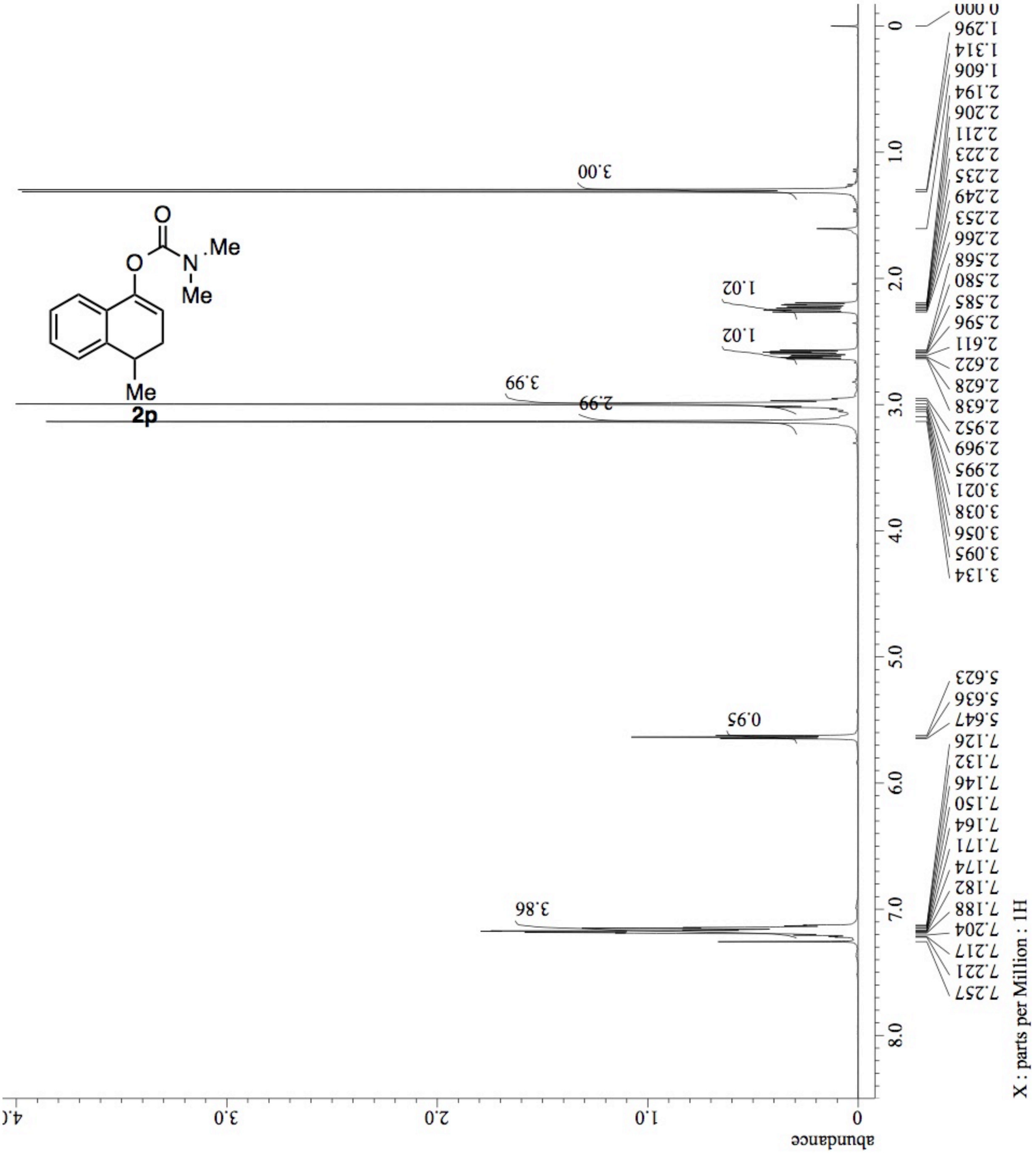

^{13}C NMR of **2p** (100 MHz, $CDCl_3$)

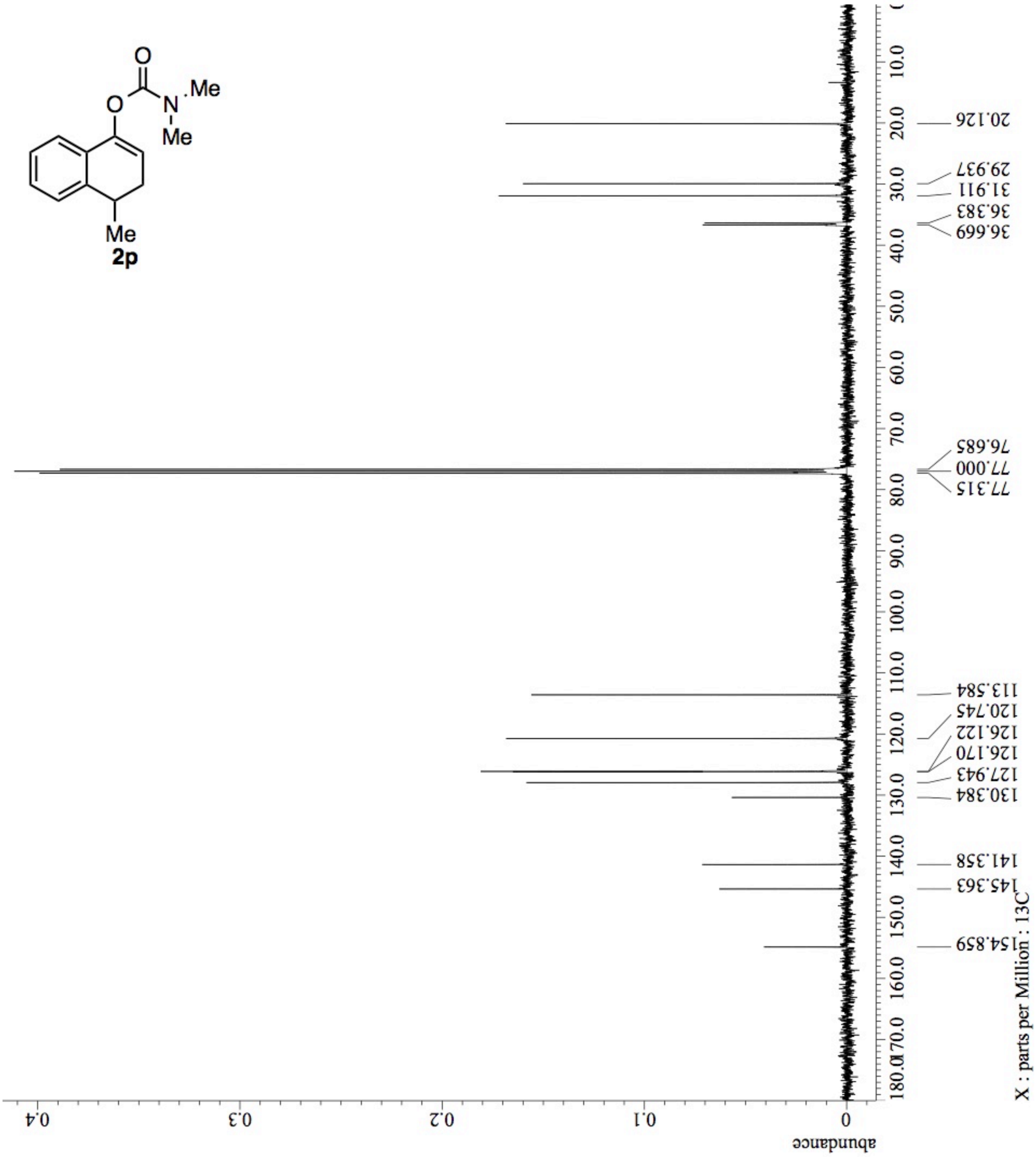

^{1}H NMR of **2q** (400 MHz, CDCl$_3$)

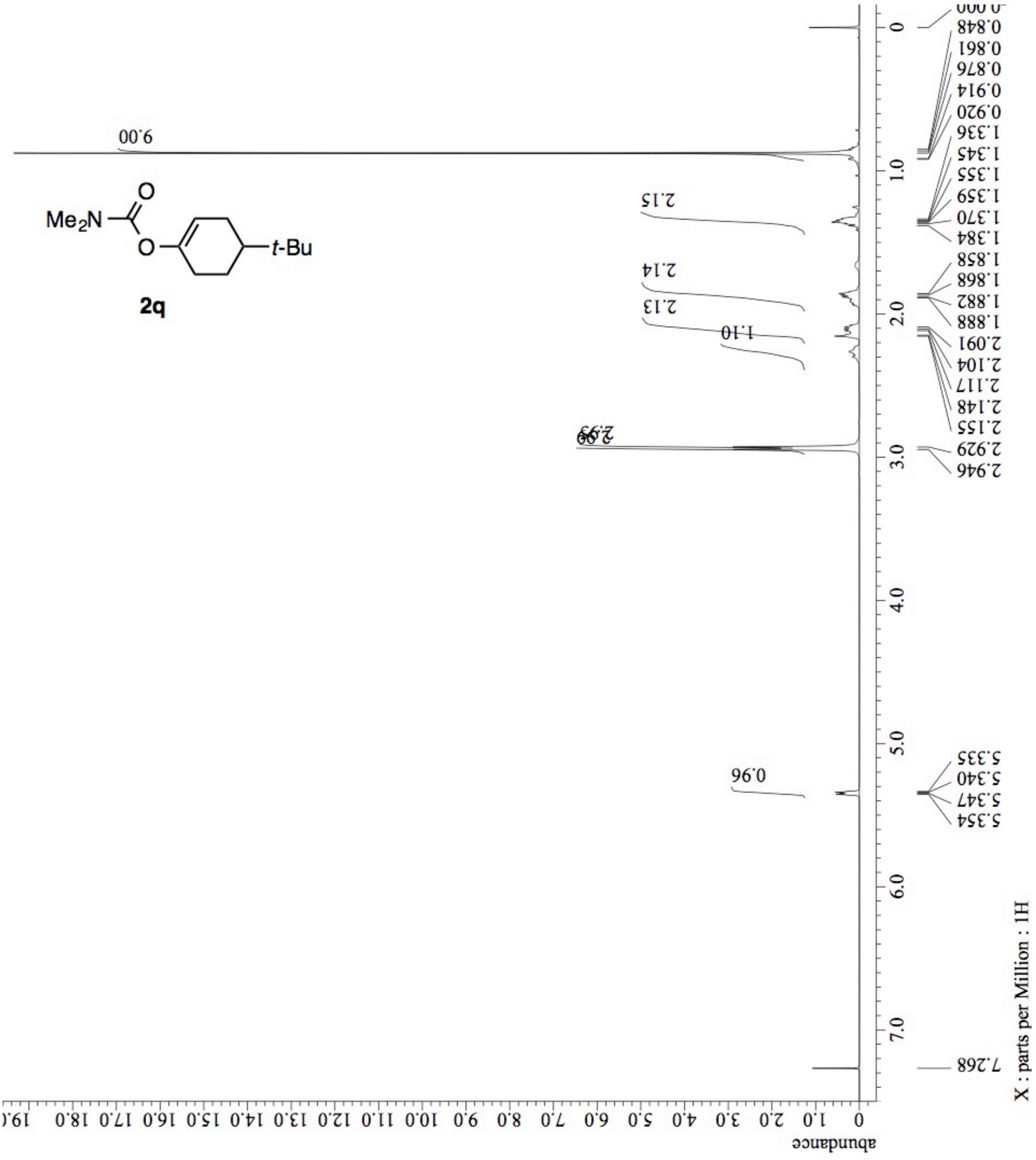

^{13}C NMR of **2q** (100 MHz, $CDCl_3$)

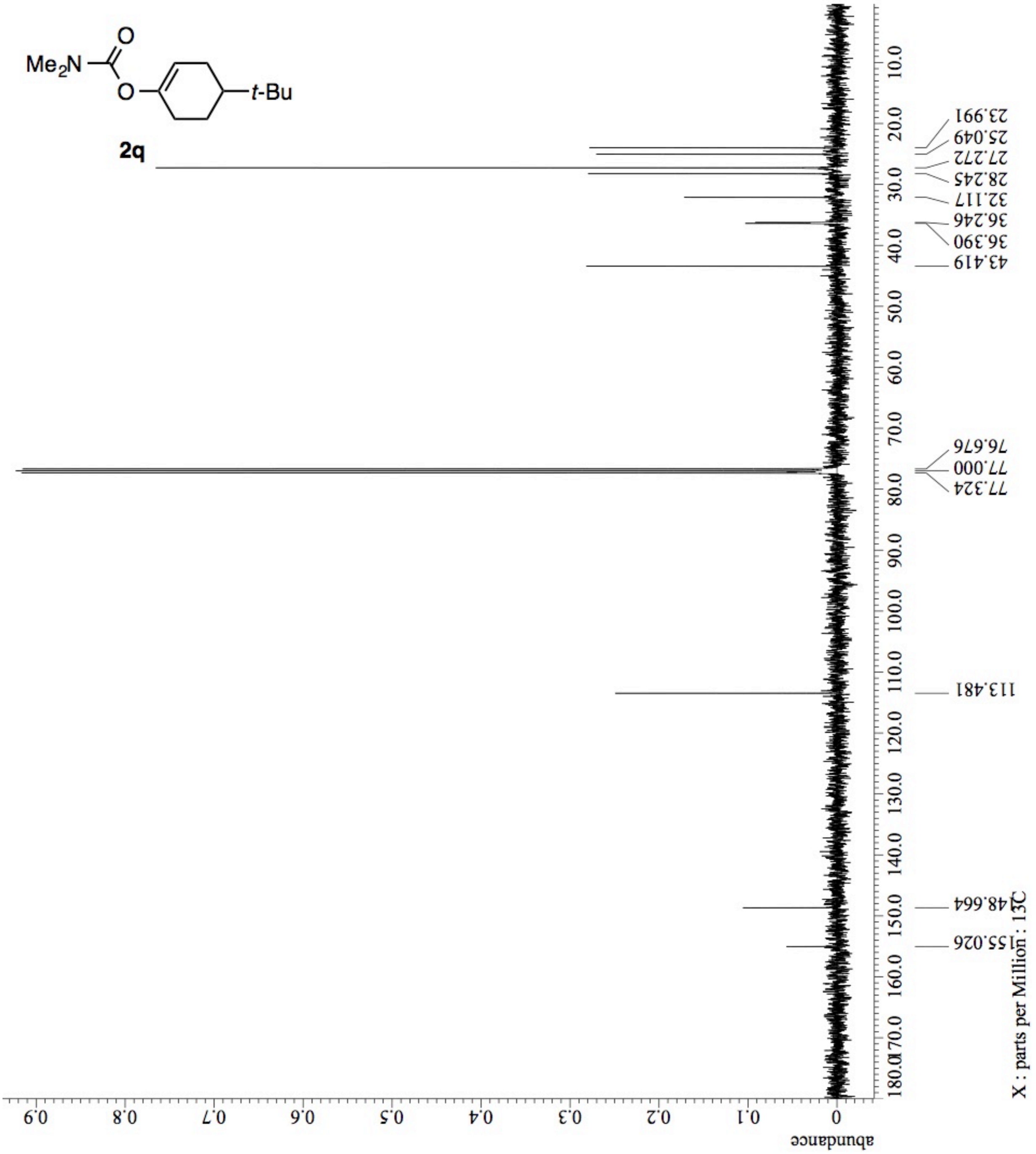

^{1}H NMR of **2r** (400 MHz, $CDCl_3$)

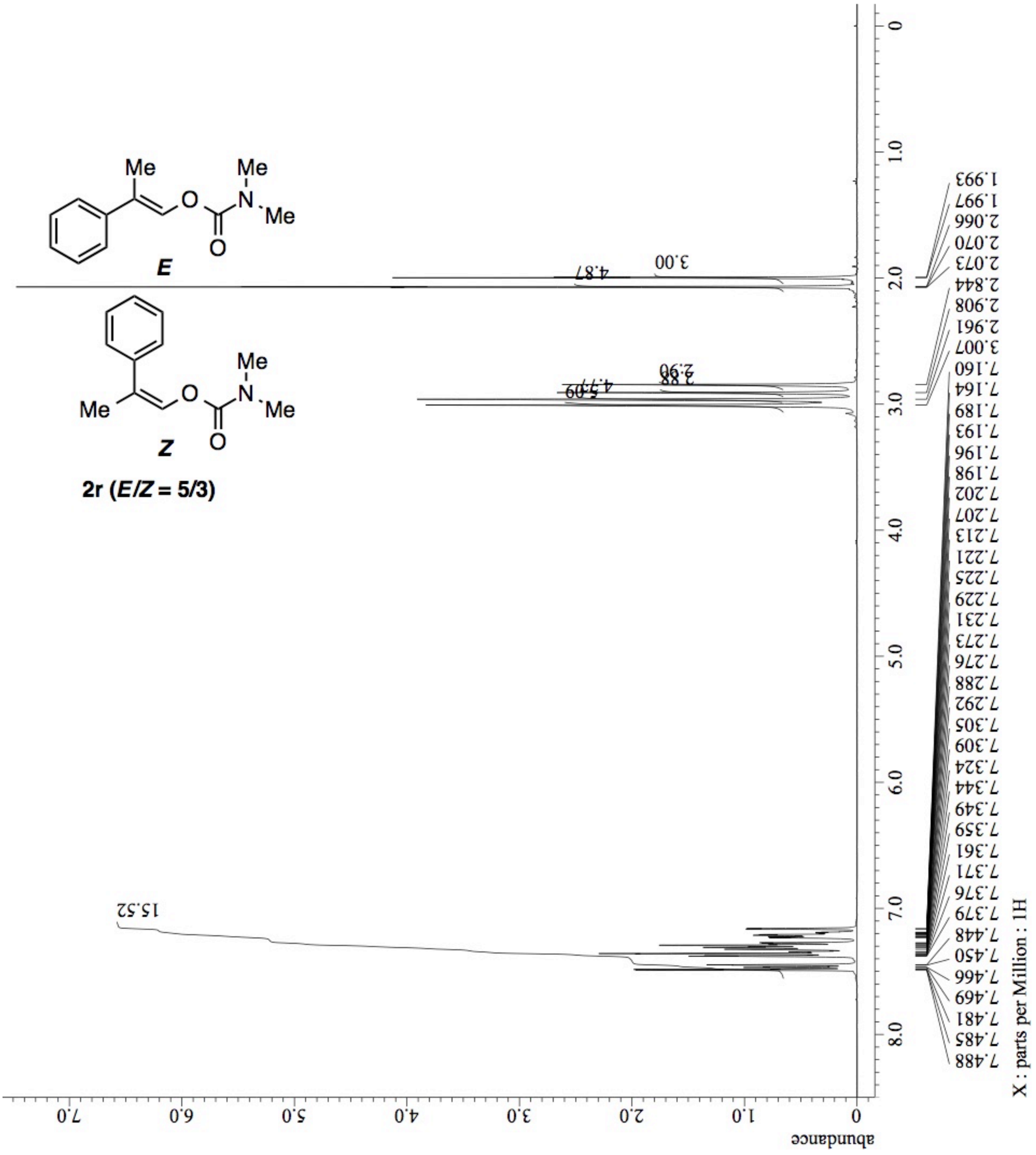

^{13}C NMR of **2r** (100 MHz, $CDCl_3$)

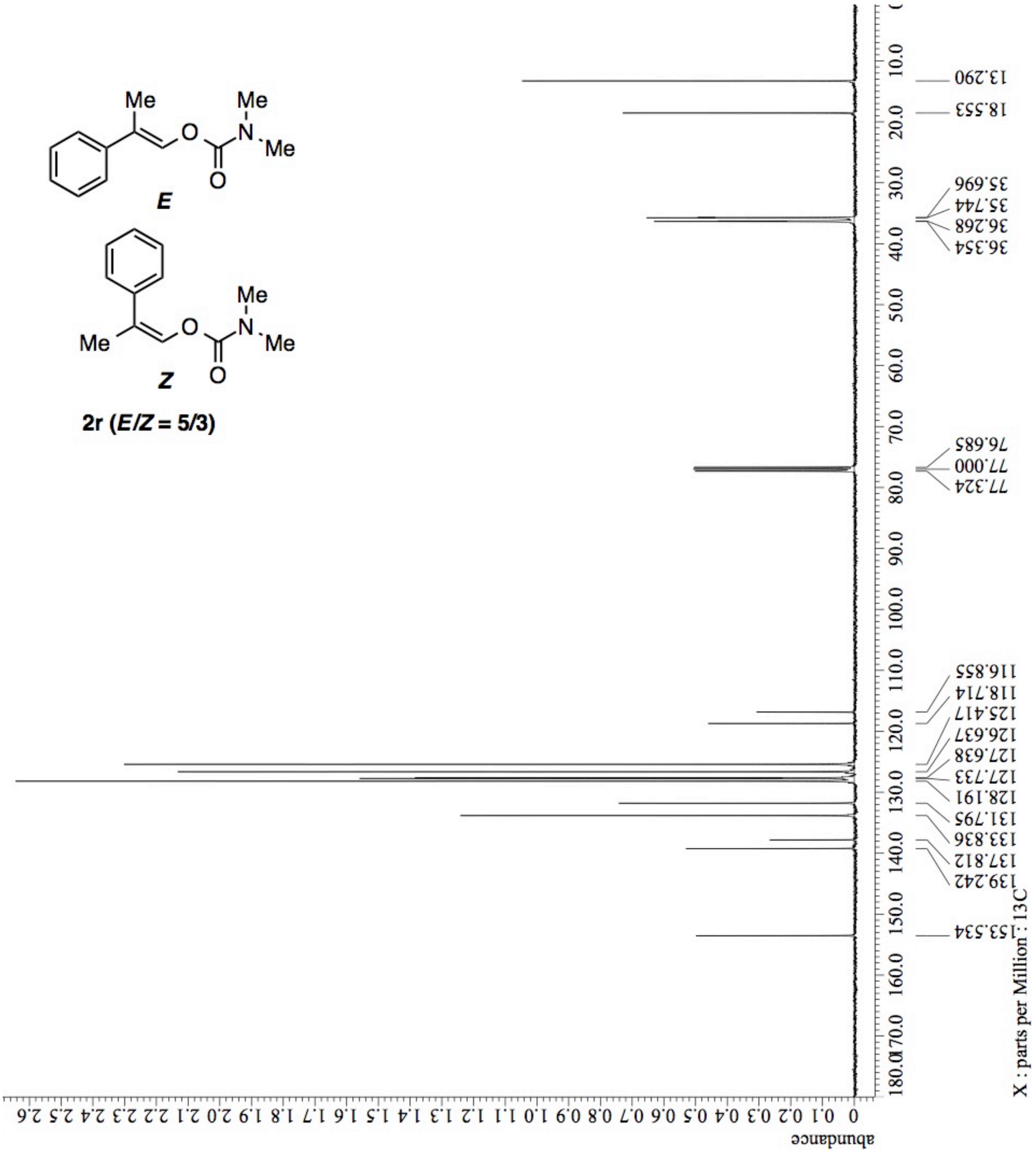

2r (***E*/*Z*** **= 5/3**)

^{1}H NMR of **4t** (400 MHz, $CDCl_3$)

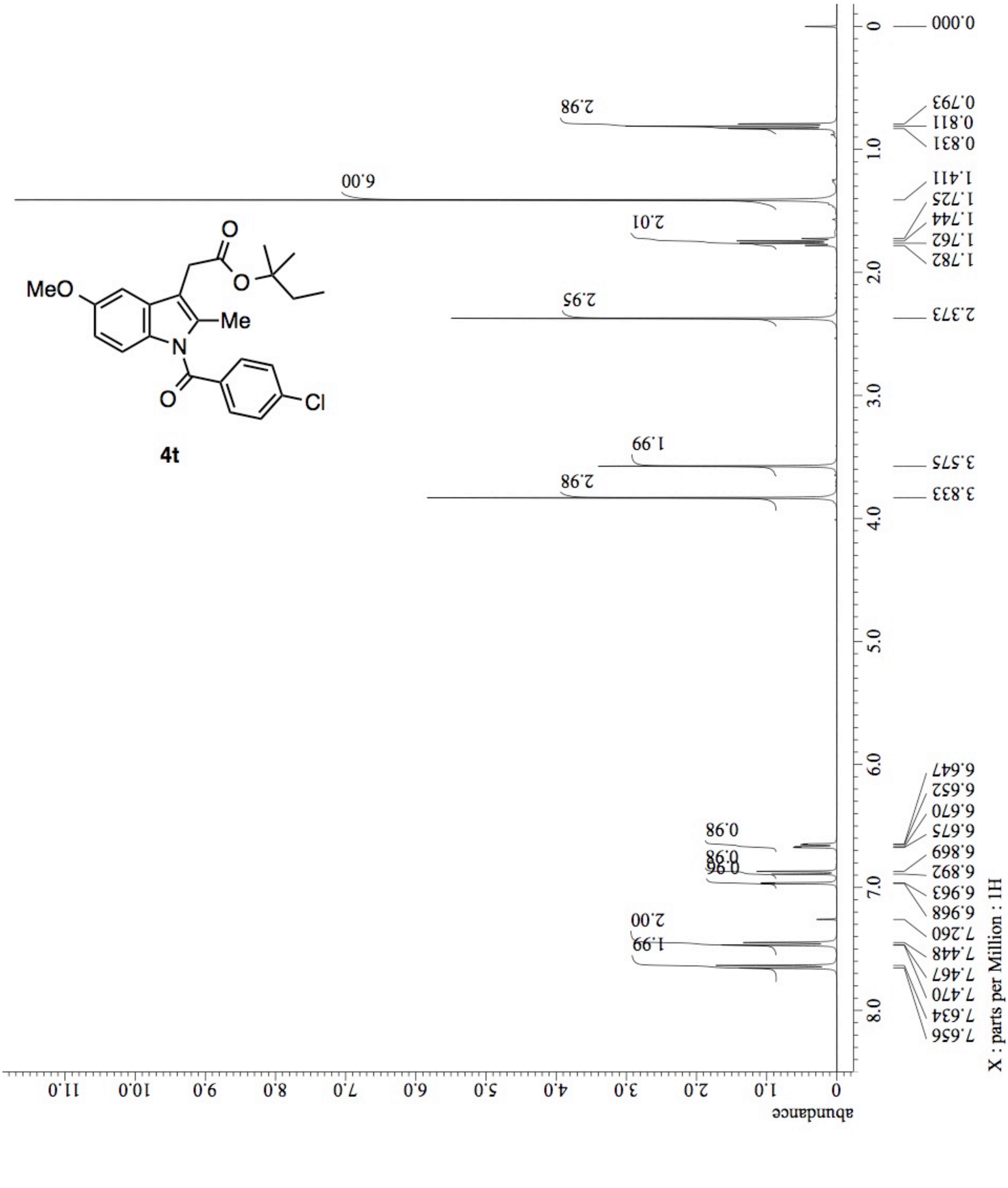

^{13}C NMR of **4t** (100 MHz, $CDCl_3$)

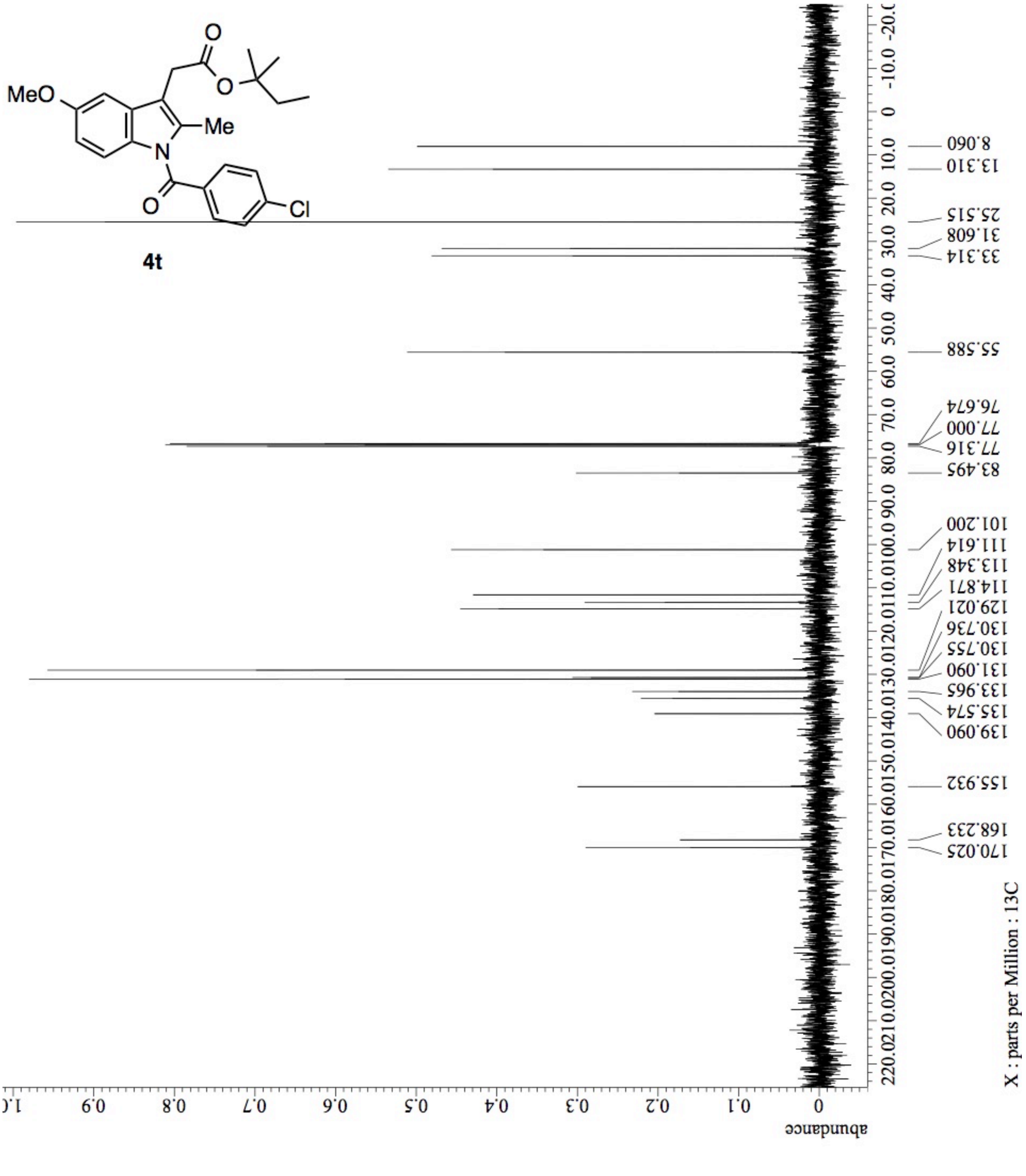

^{1}H NMR of **L1** (400 MHz, $CDCl_3$)

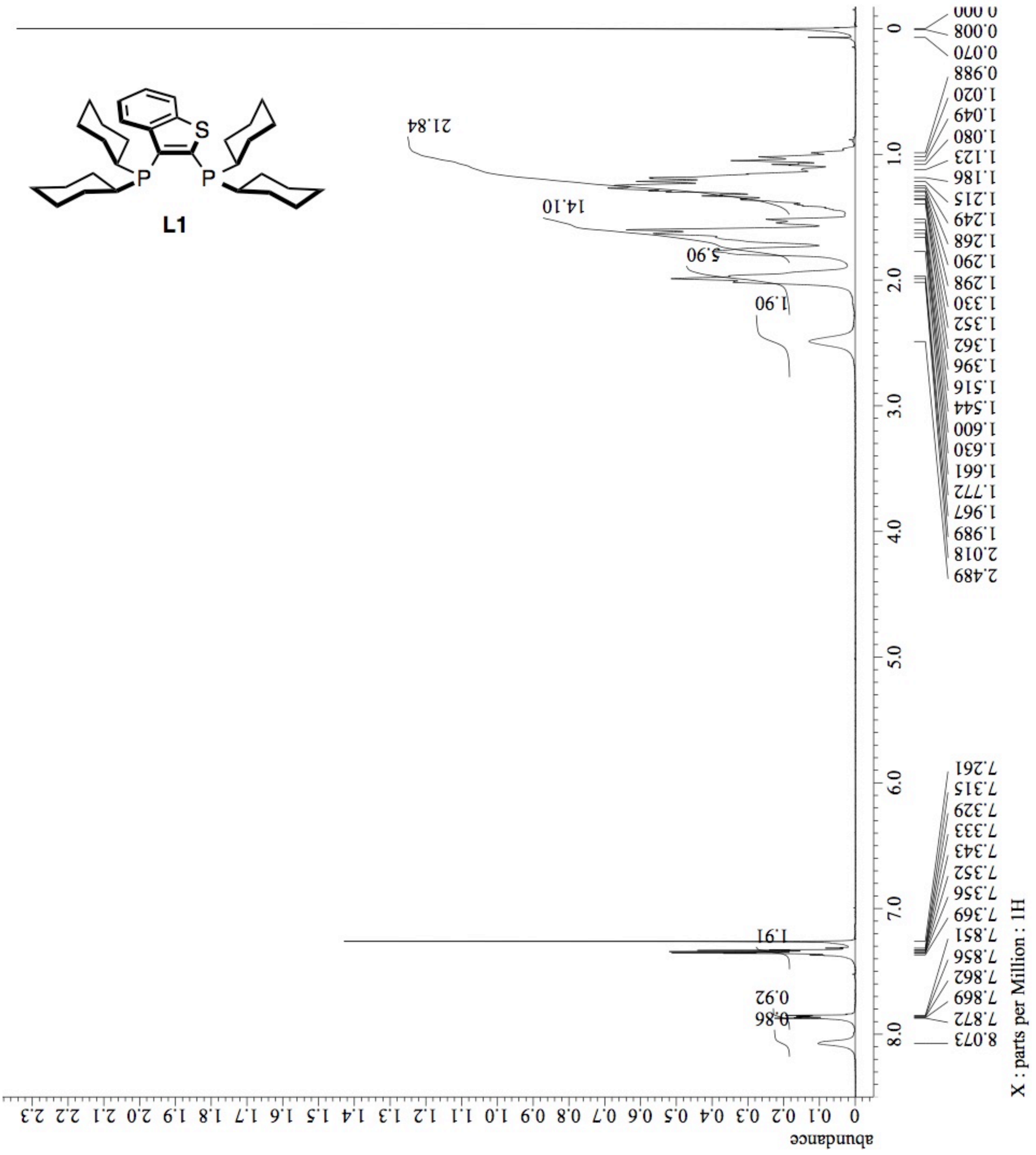

^{31}P NMR of **L1** (160 MHz, $CDCl_3$)

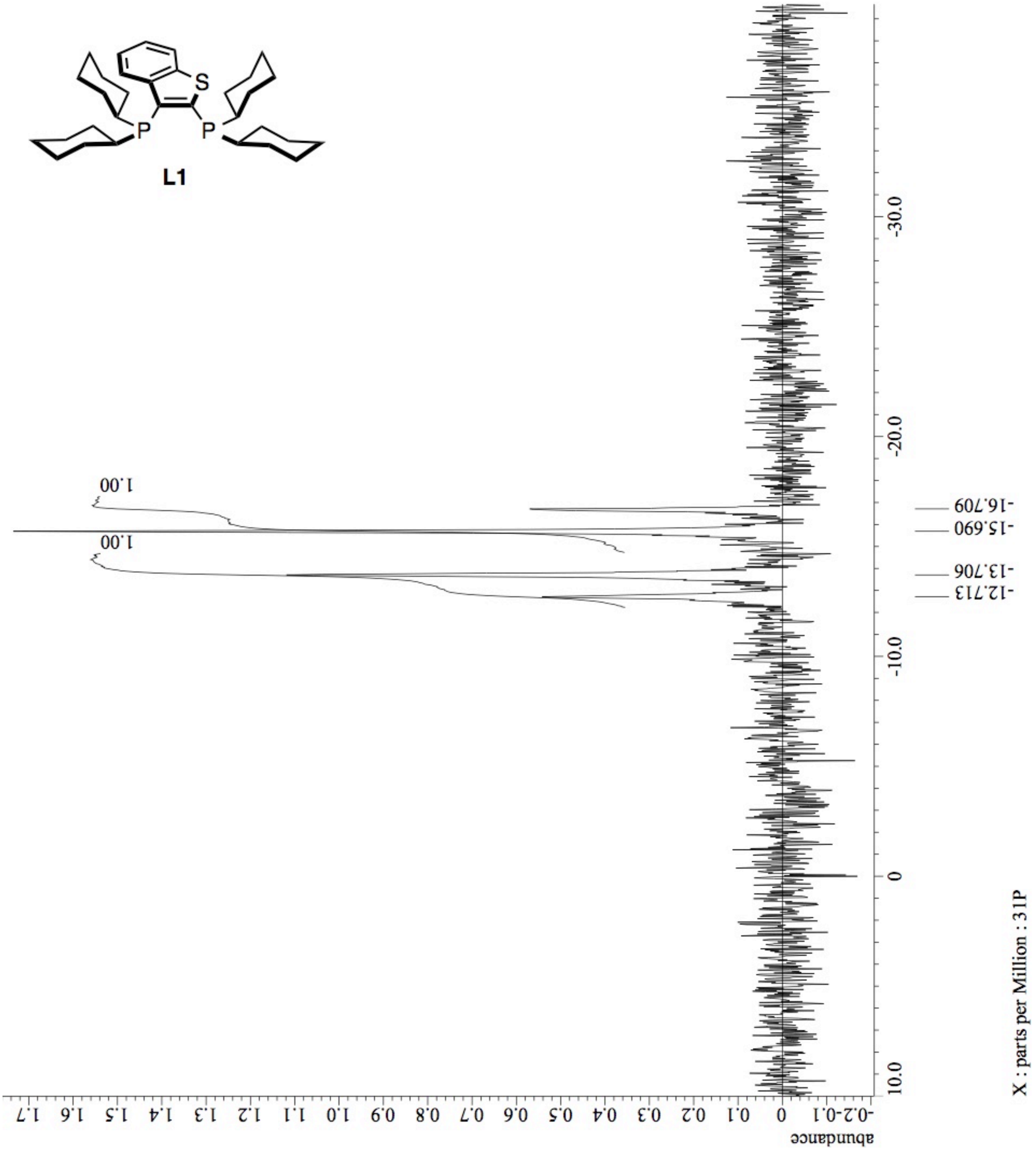

^{1}H NMR of **L2** (400 MHz, $CDCl_3$)

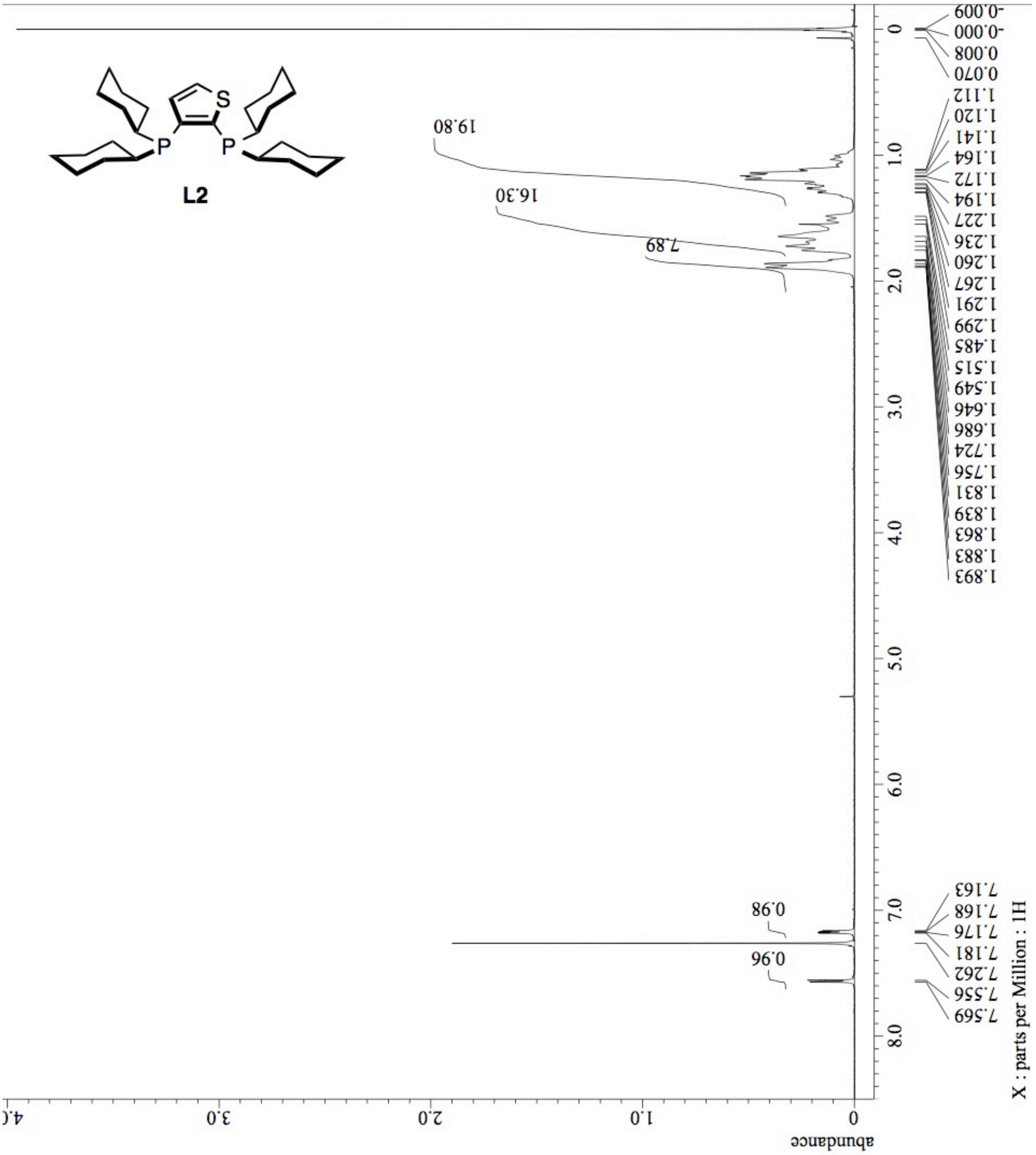

^{31}P NMR of **L2** (160 MHz, $CDCl_3$)

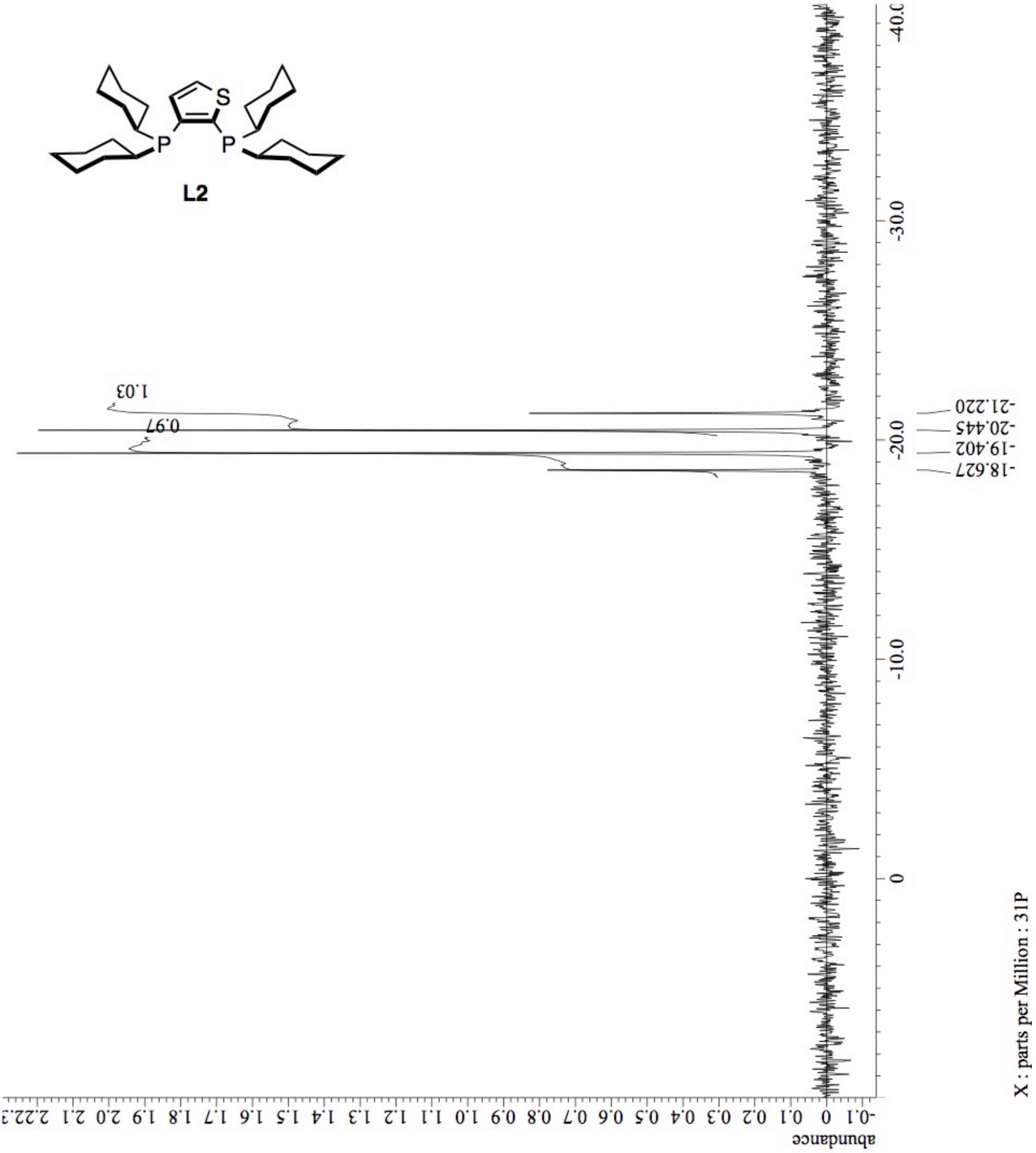

^{1}H NMR of **3Aa** (400 MHz, $CDCl_3$)

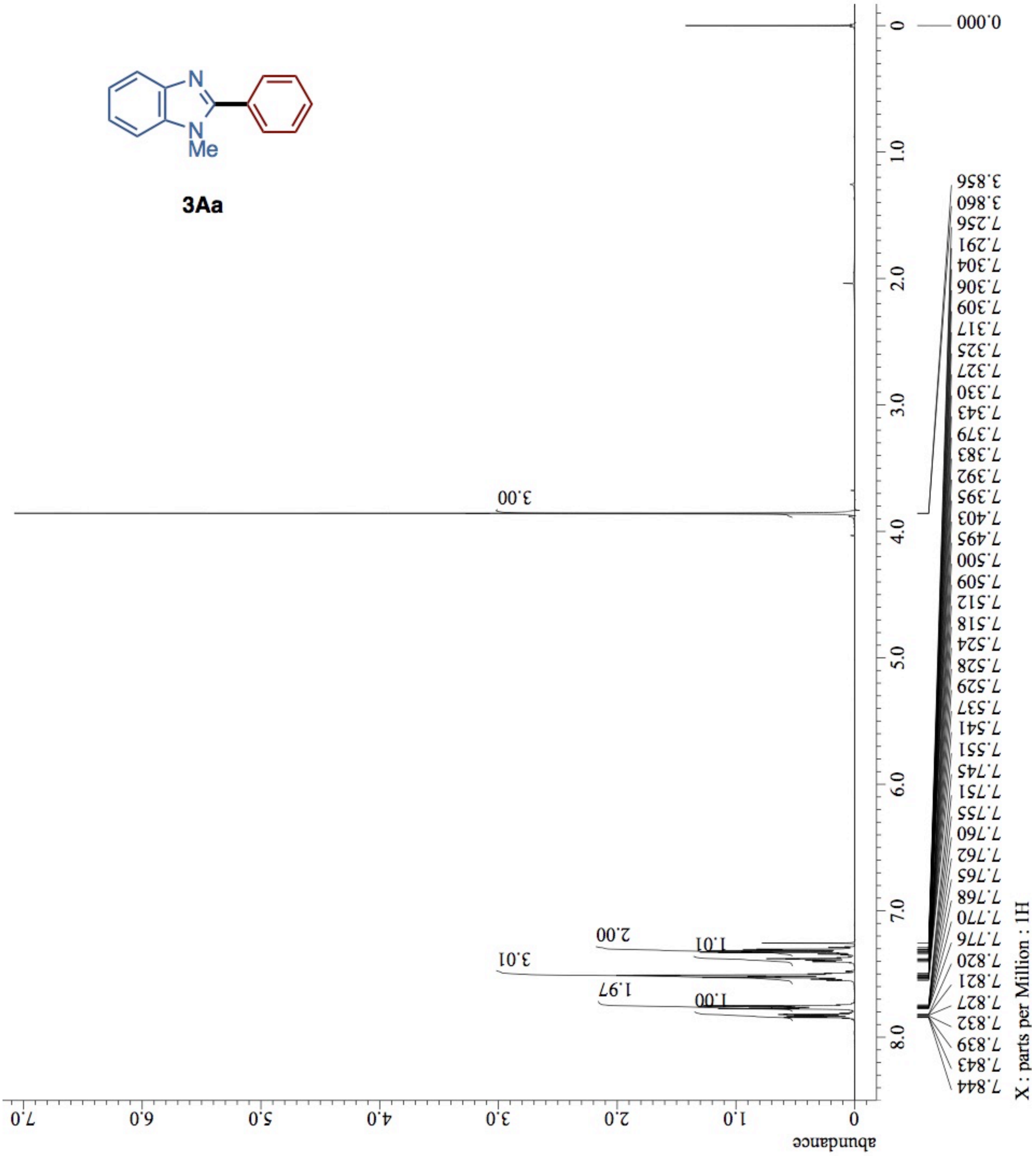

^{13}C NMR of **3Aa** (100 MHz, $CDCl_3$)

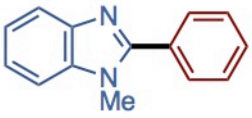

3Aa

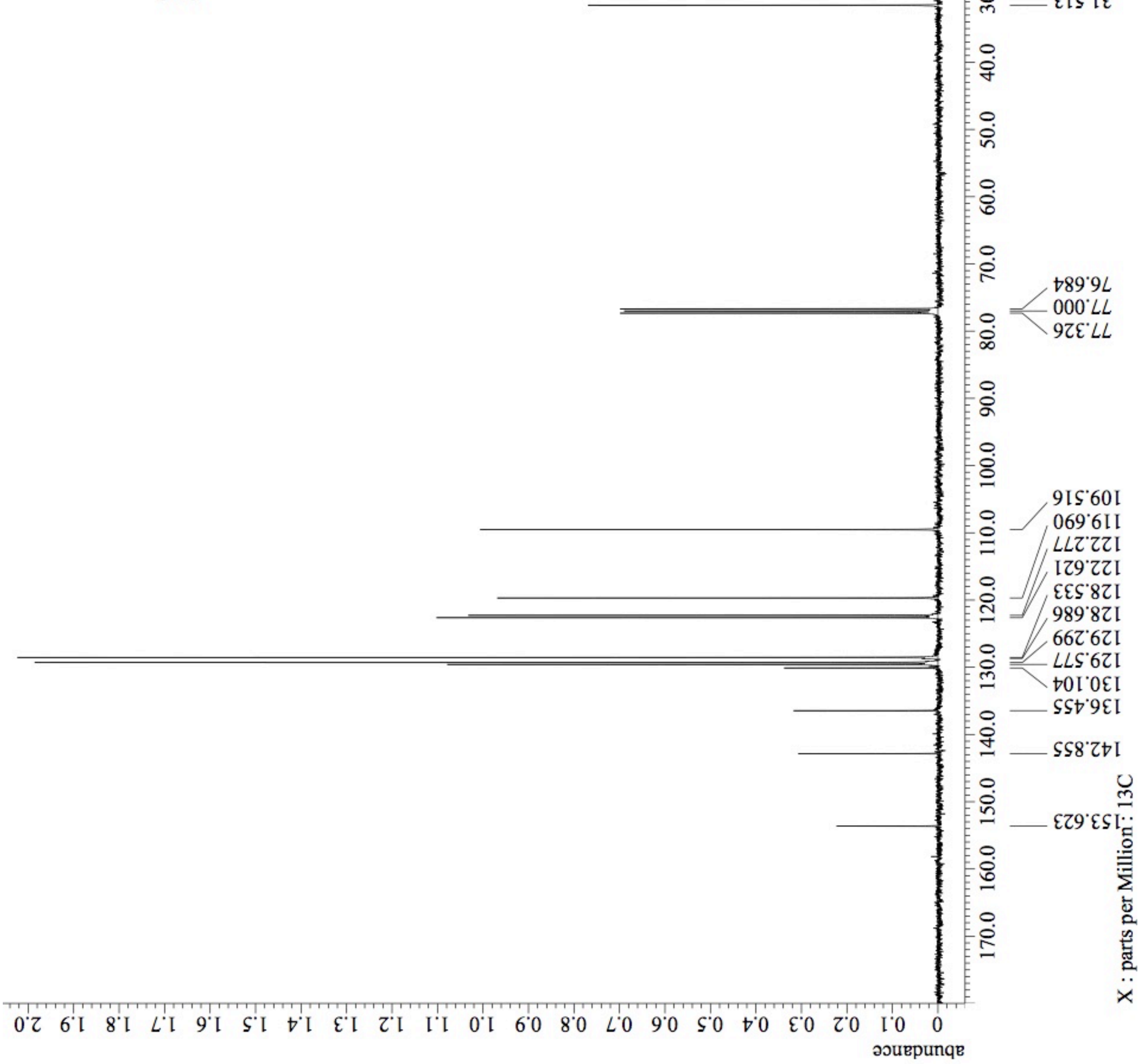

^{1}H NMR of **3Ba** (400 MHz, $CDCl_3$)

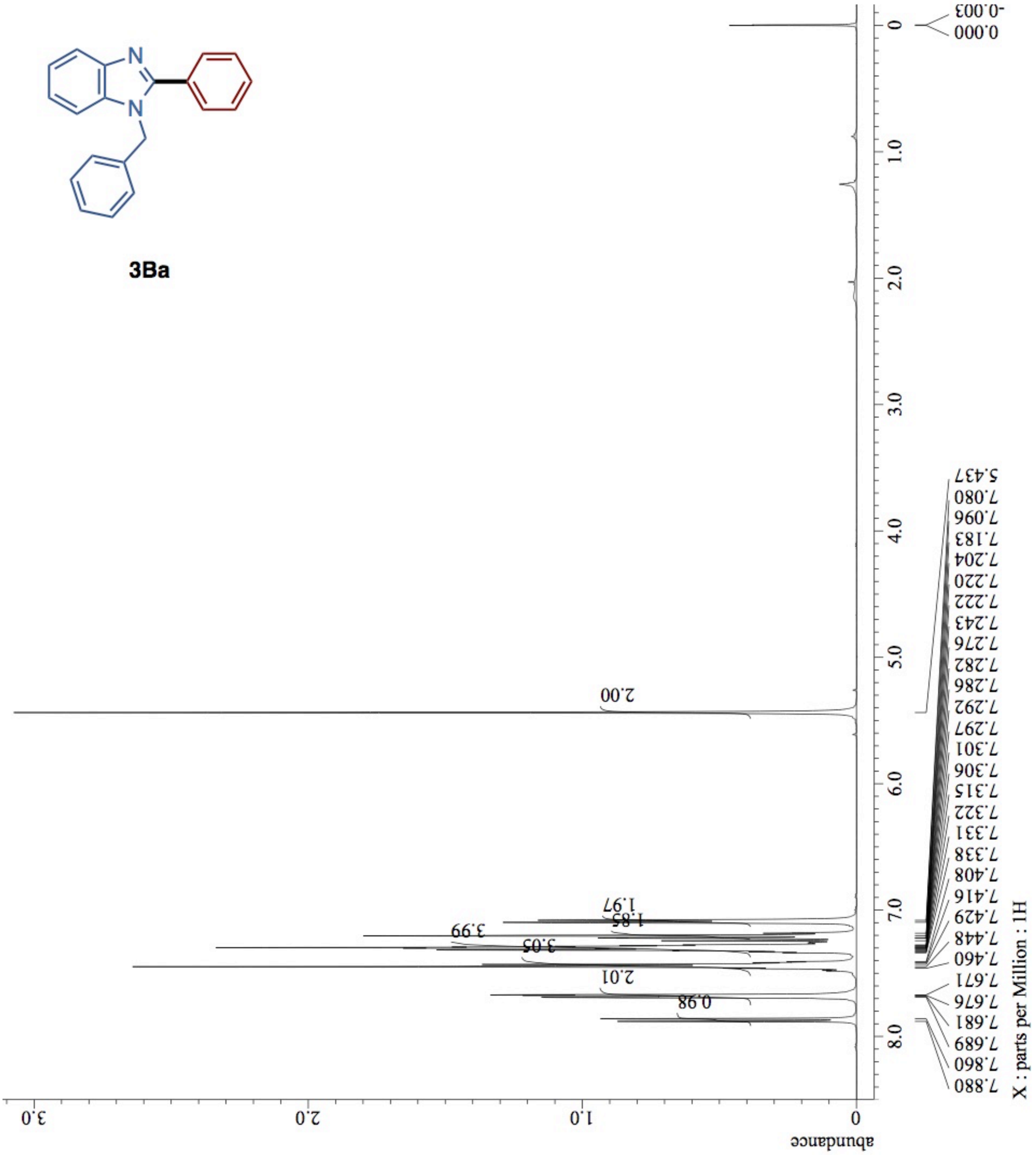

^{13}C NMR of **3Ba** (100 MHz, $CDCl_3$)

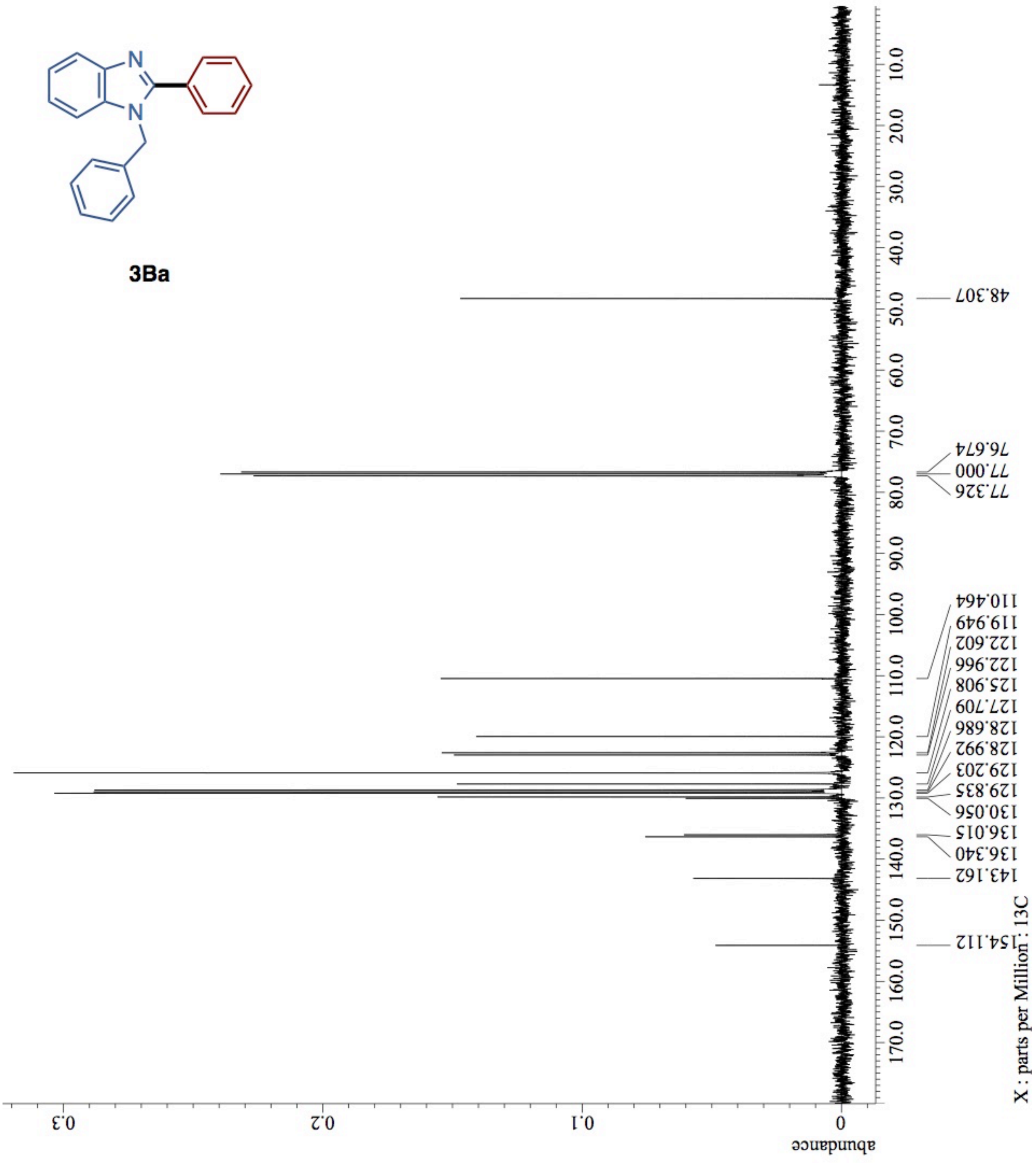

^{1}H NMR of **3Ca** (400 MHz, $CDCl_3$)

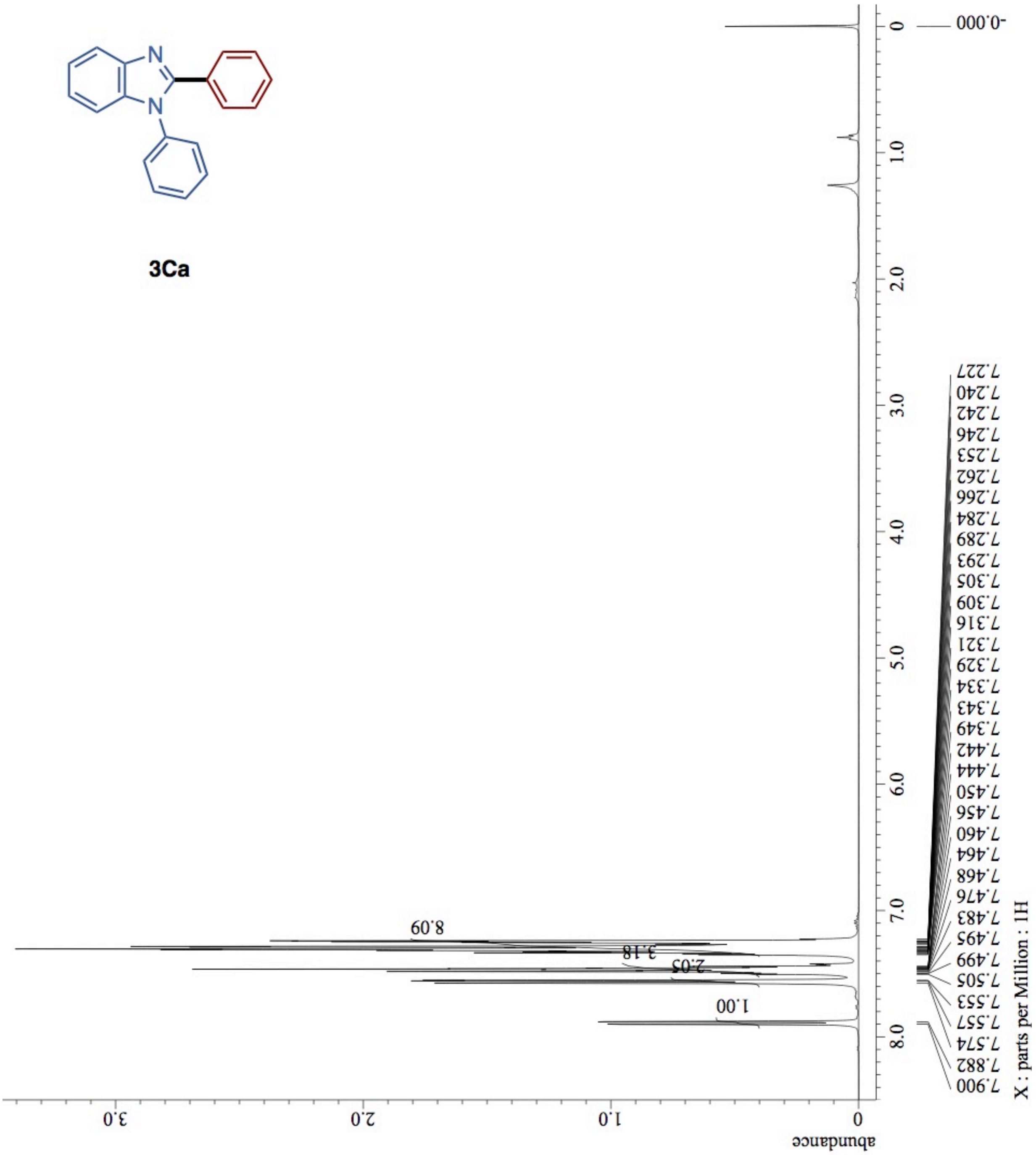

^{13}C NMR of **3Ca** (100 MHz, $CDCl_3$)

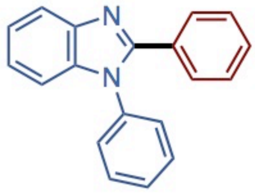

3Ca

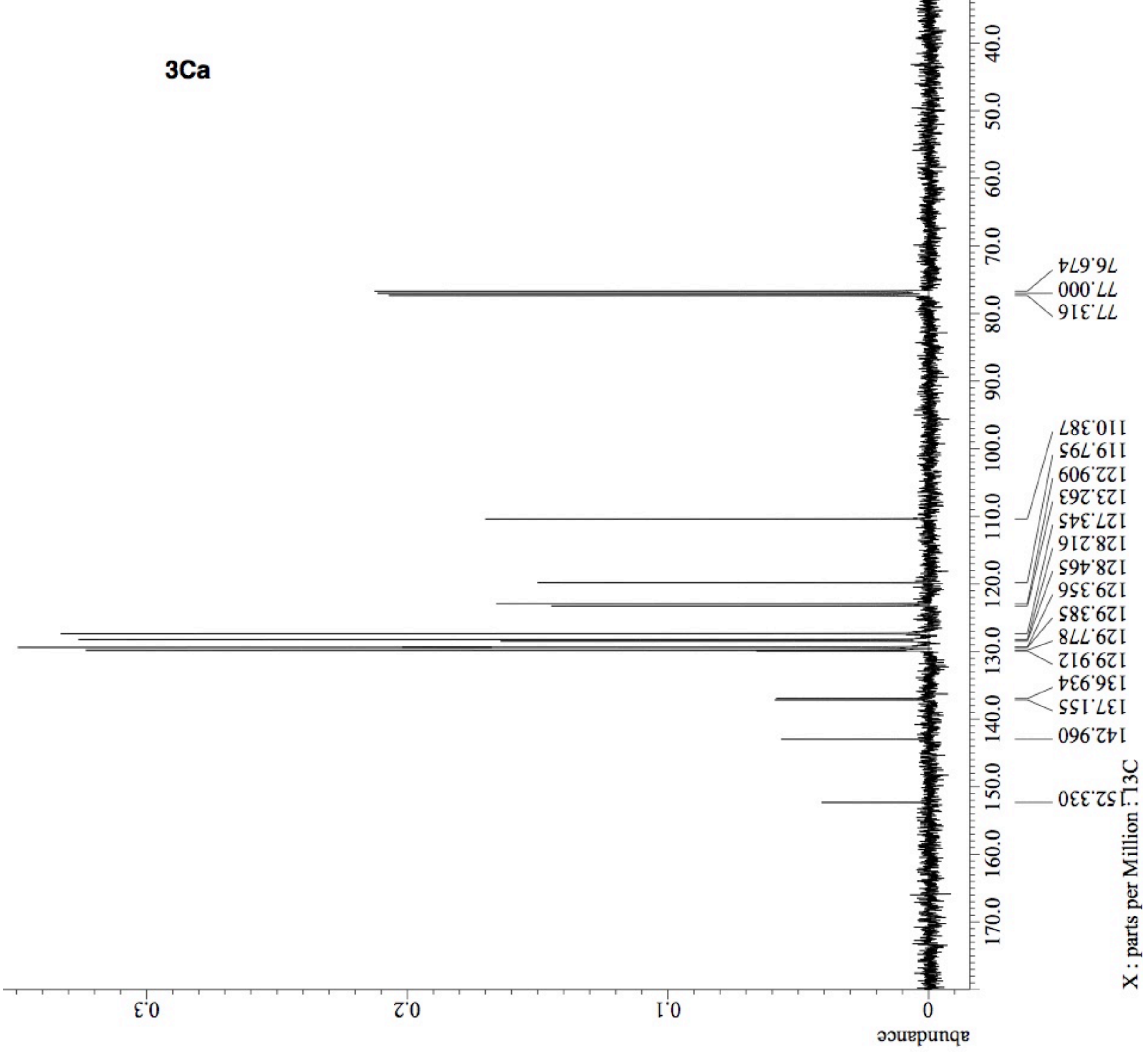

^{1}H NMR of **3Da** (400 MHz, $CDCl_3$)

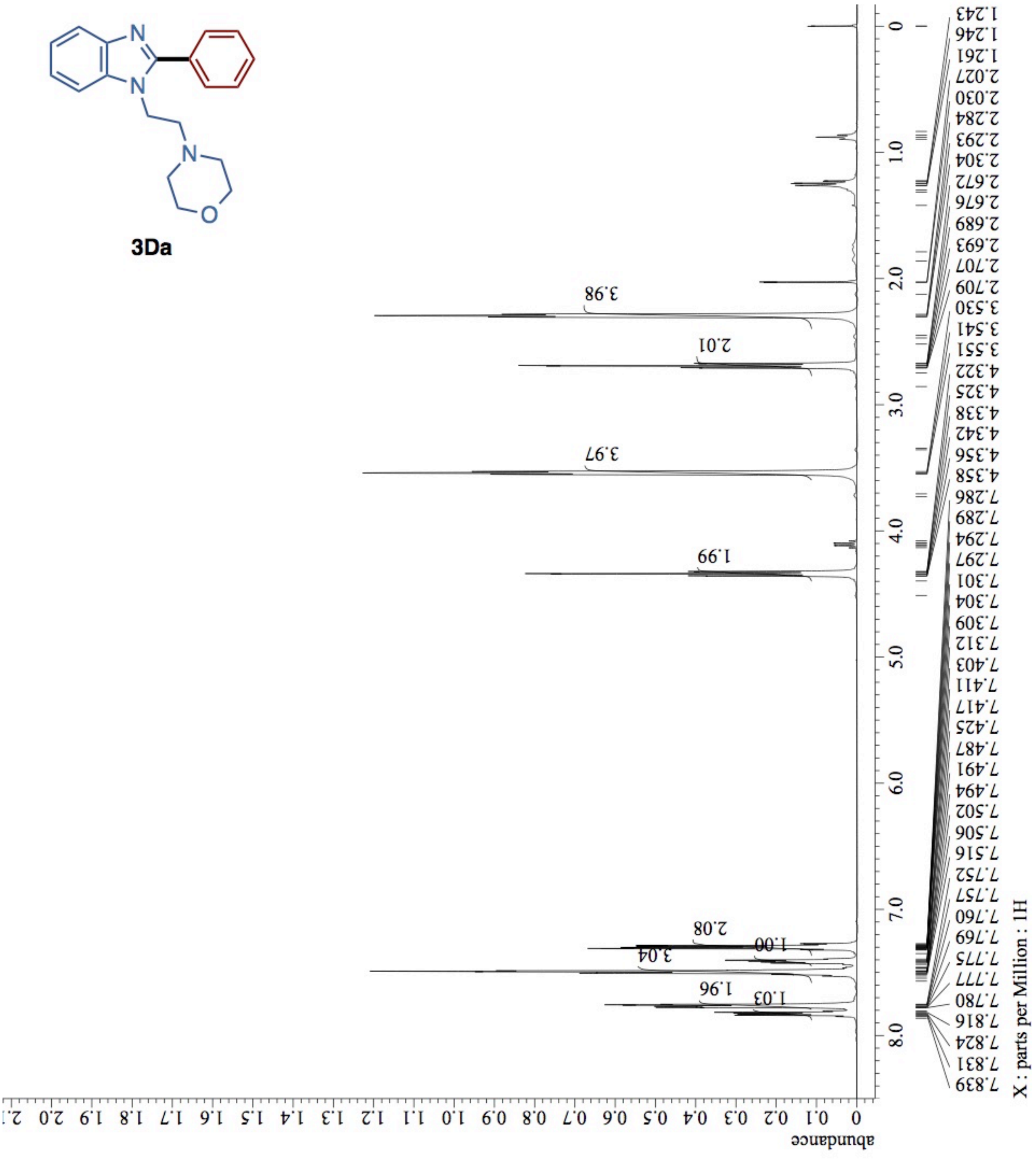

^{13}C NMR of **3Da** (100 MHz, $CDCl_3$)

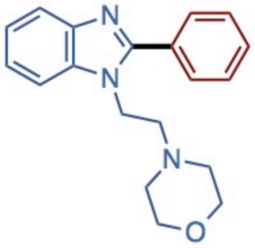

3Da

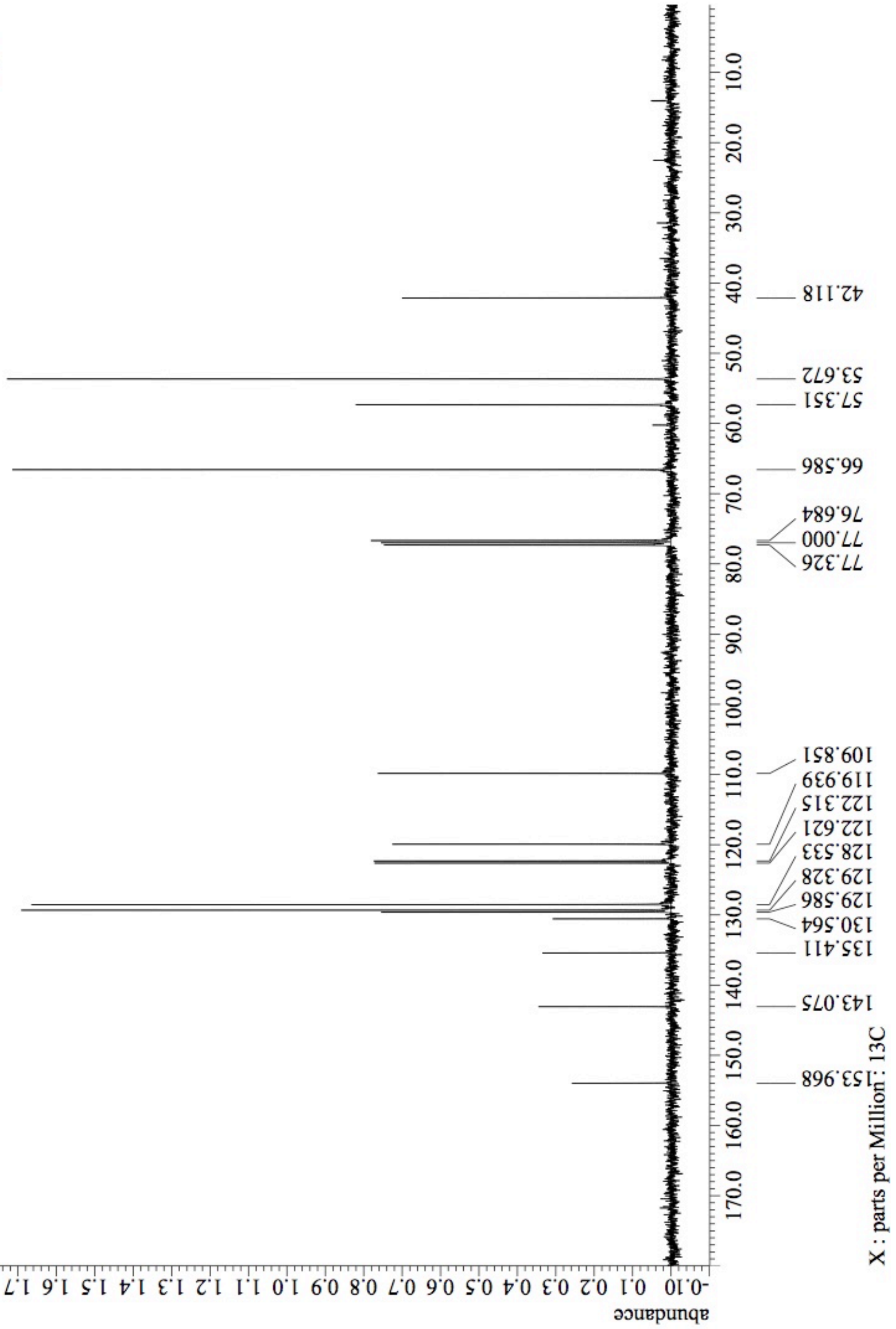

^{1}H NMR of **3Ea** (400 MHz, CDCl$_3$)

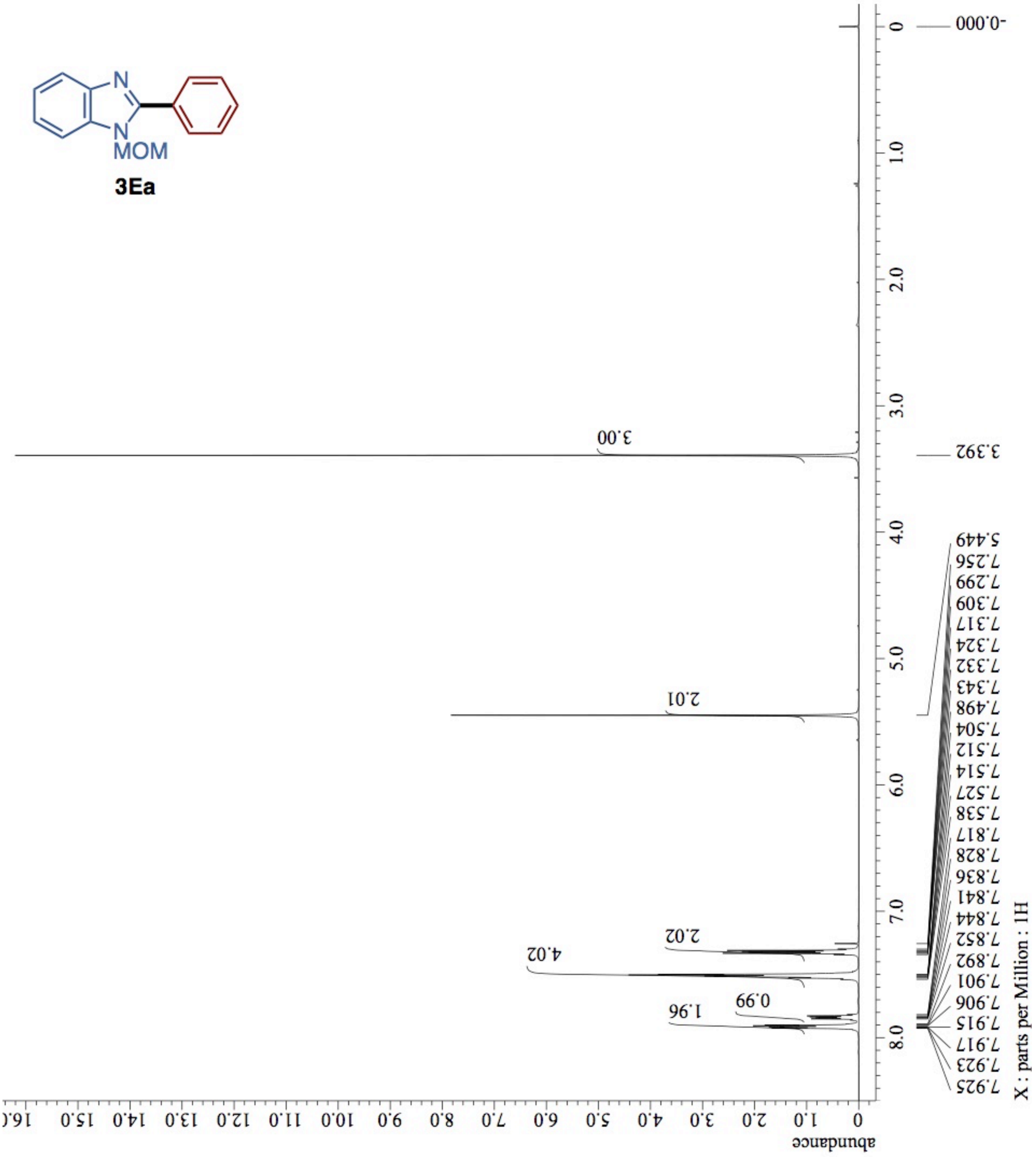

^{13}C NMR of **3Ea** (100 MHz, $CDCl_3$)

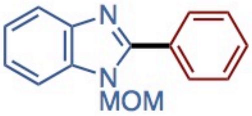

3Ea

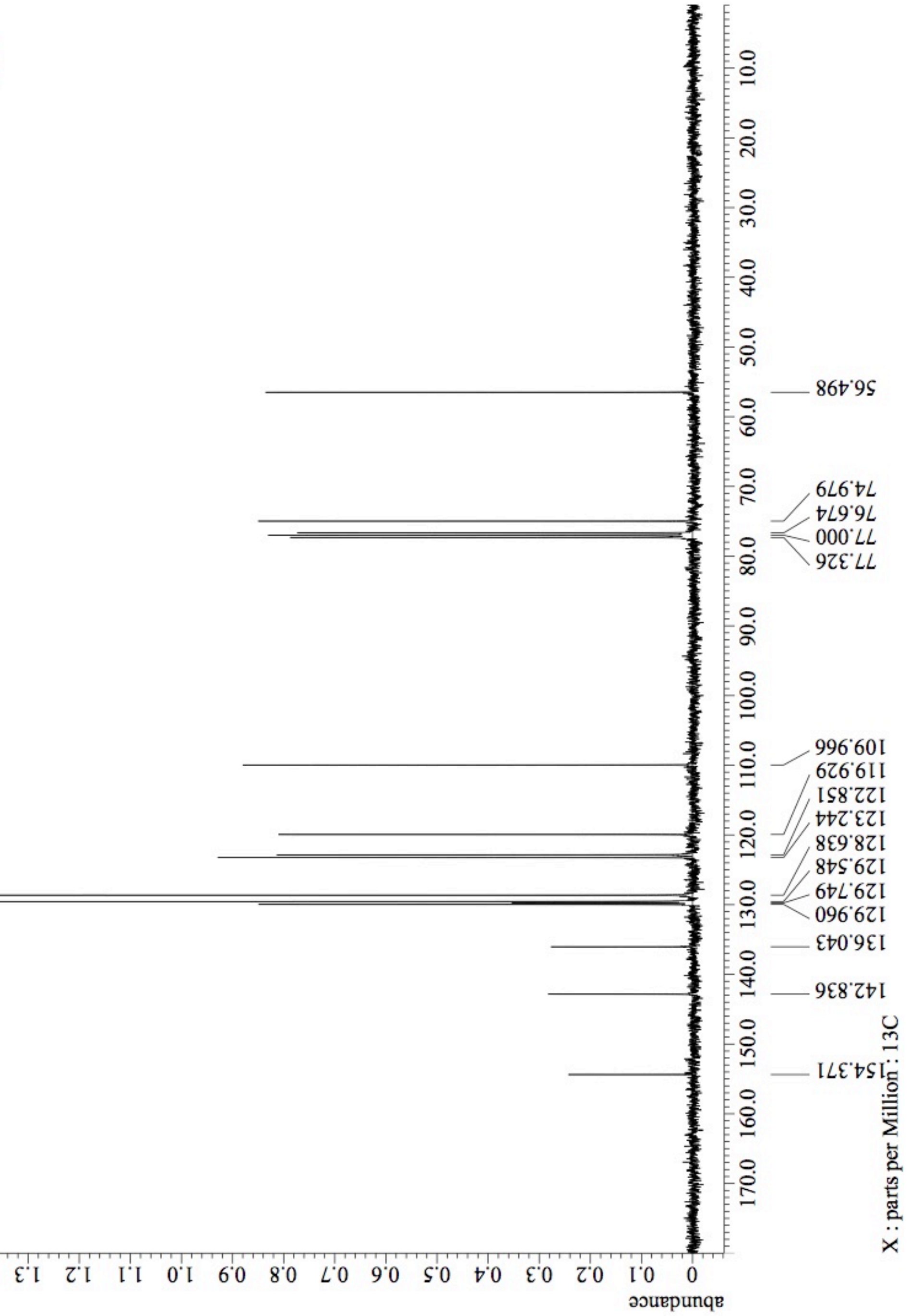

^{1}H NMR of **3Fa** (400 MHz, $CDCl_3$)

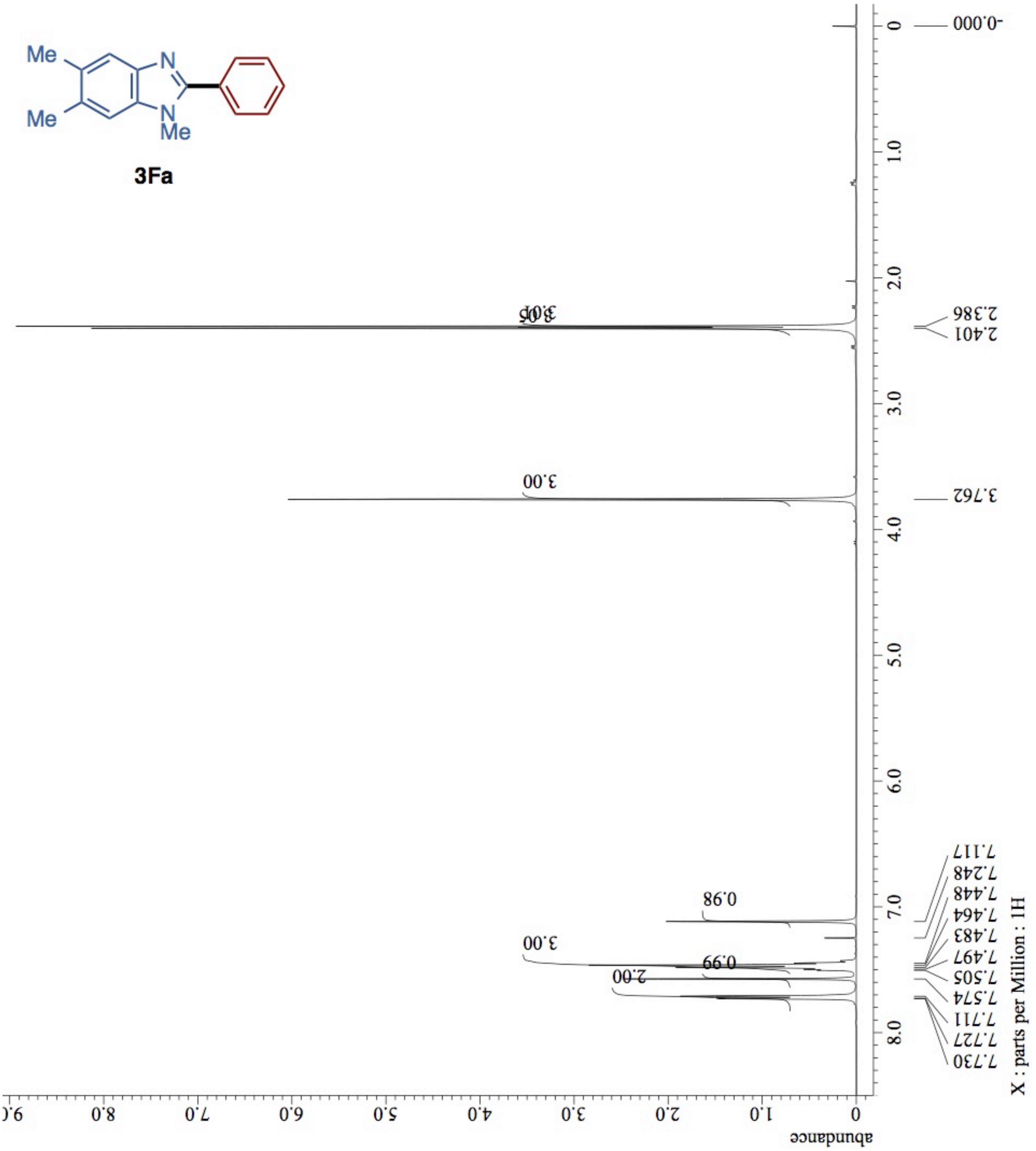

^{13}C NMR of **3Fa** (100 MHz, $CDCl_3$)

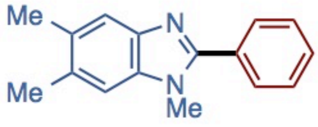

3Fa

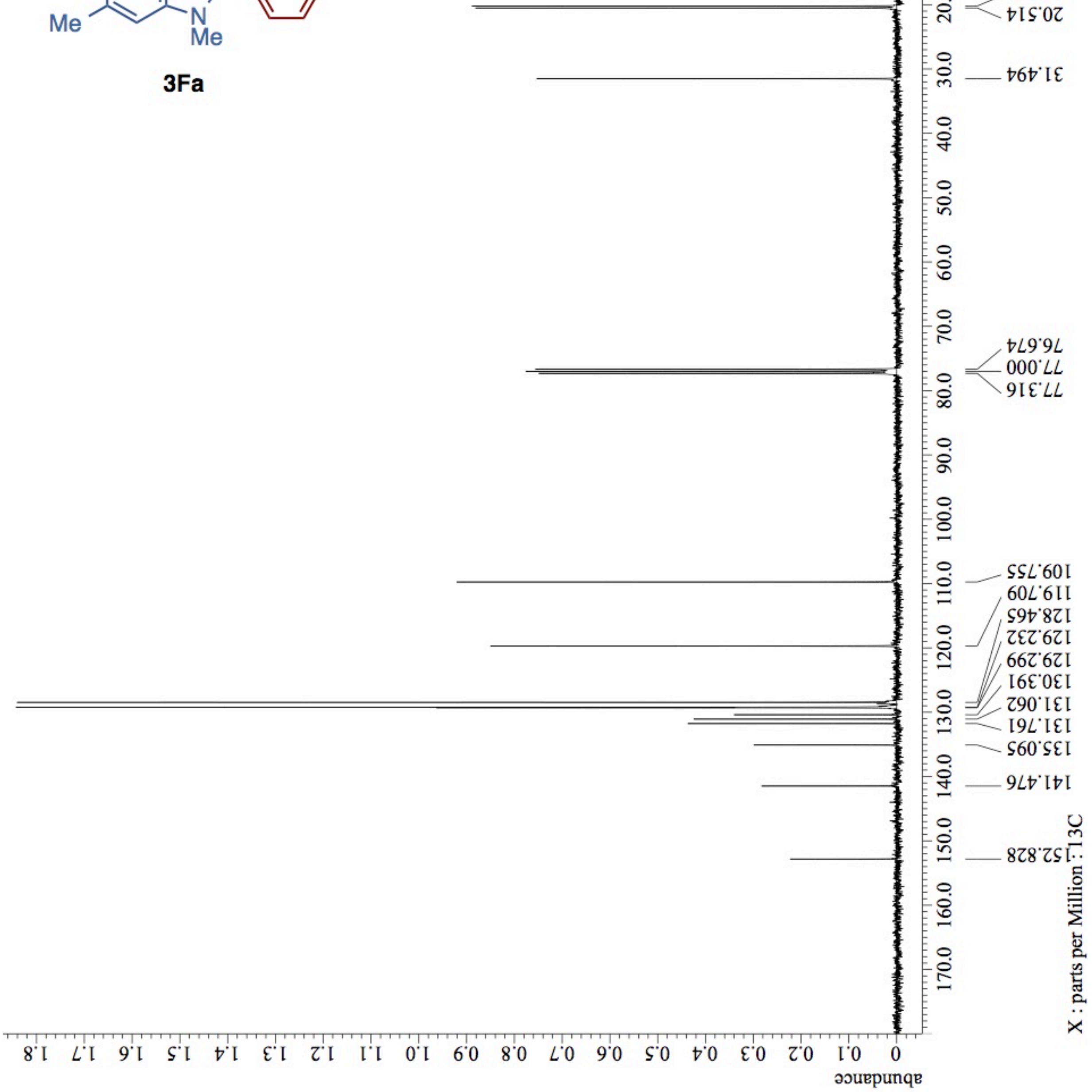

^{1}H NMR of **3Ga** (400 MHz, $CDCl_3$)

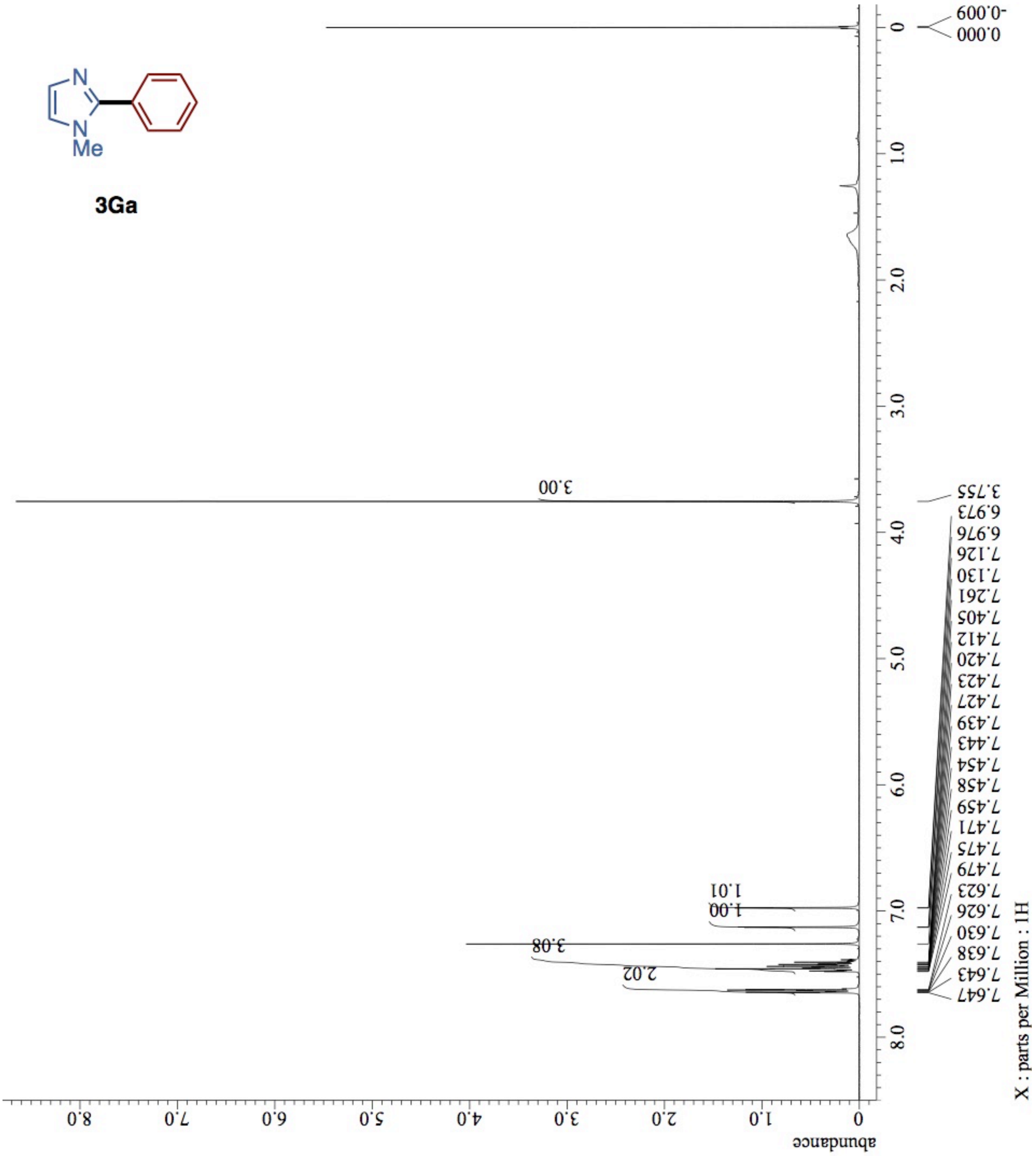

^{13}C NMR of **3Ga** (100 MHz, $CDCl_3$)

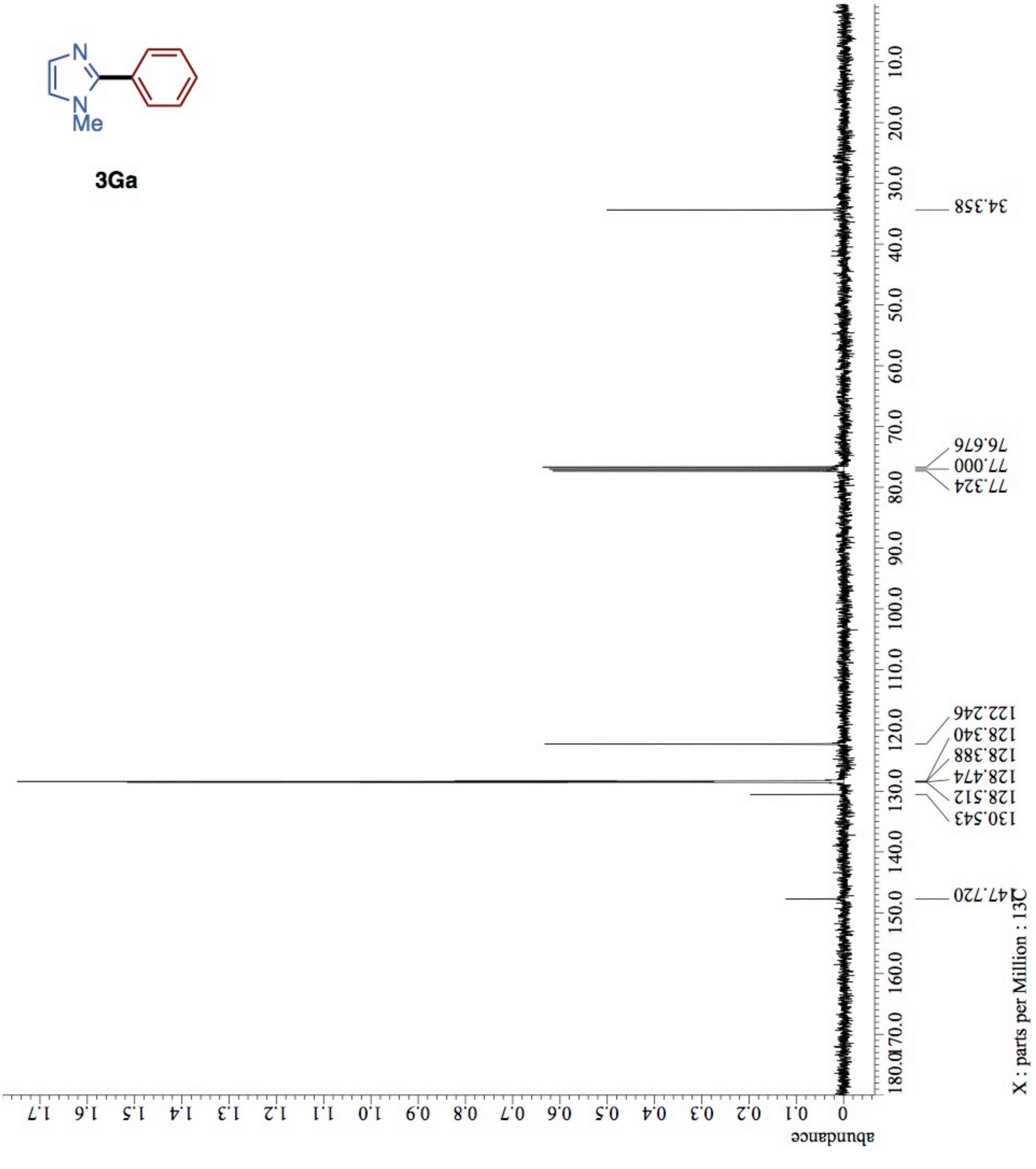

^{1}H NMR of **3Ha** (400 MHz, $CDCl_3$)

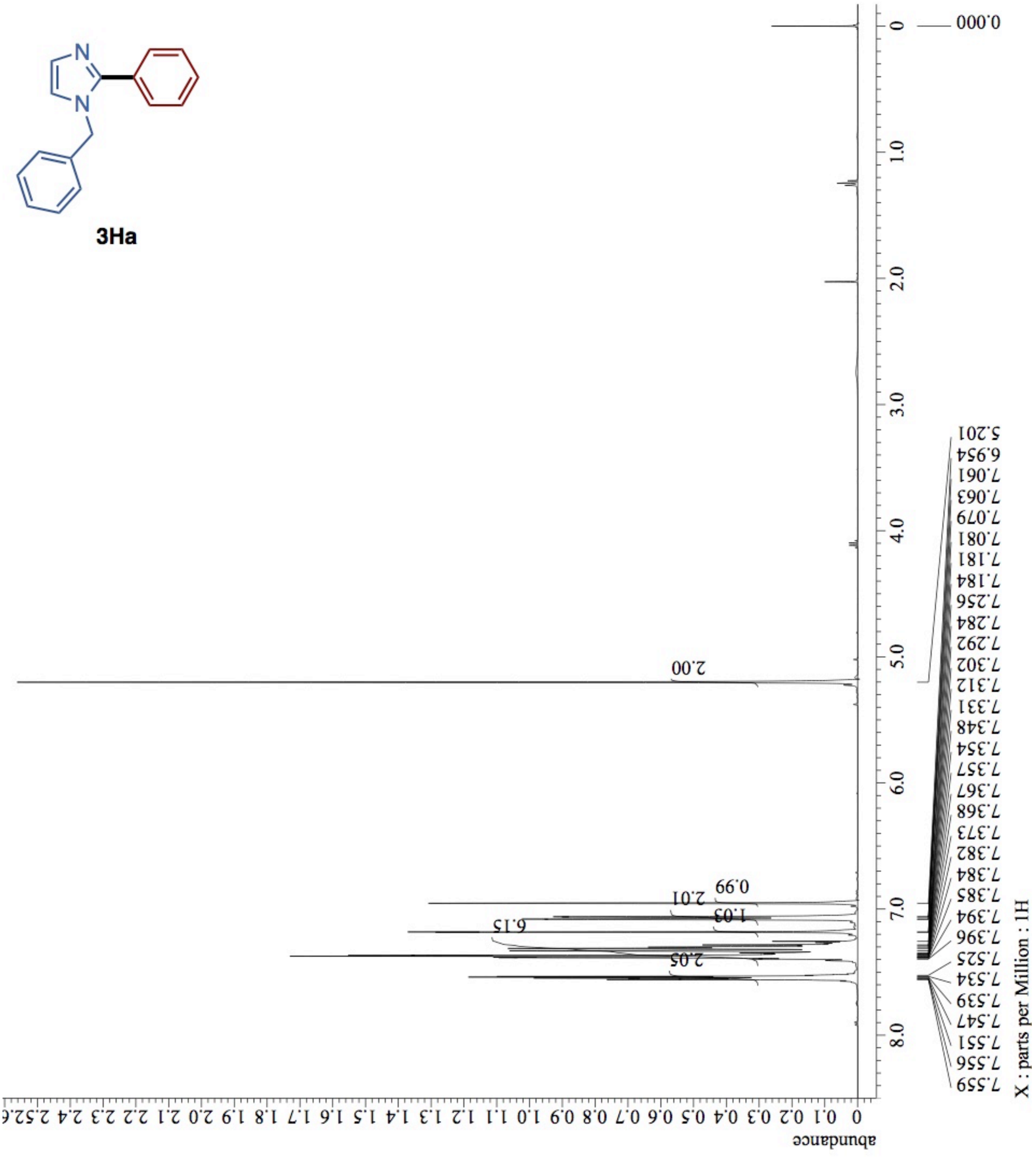

^{13}C NMR of **3Ha** (100 MHz, CDCl$_3$)

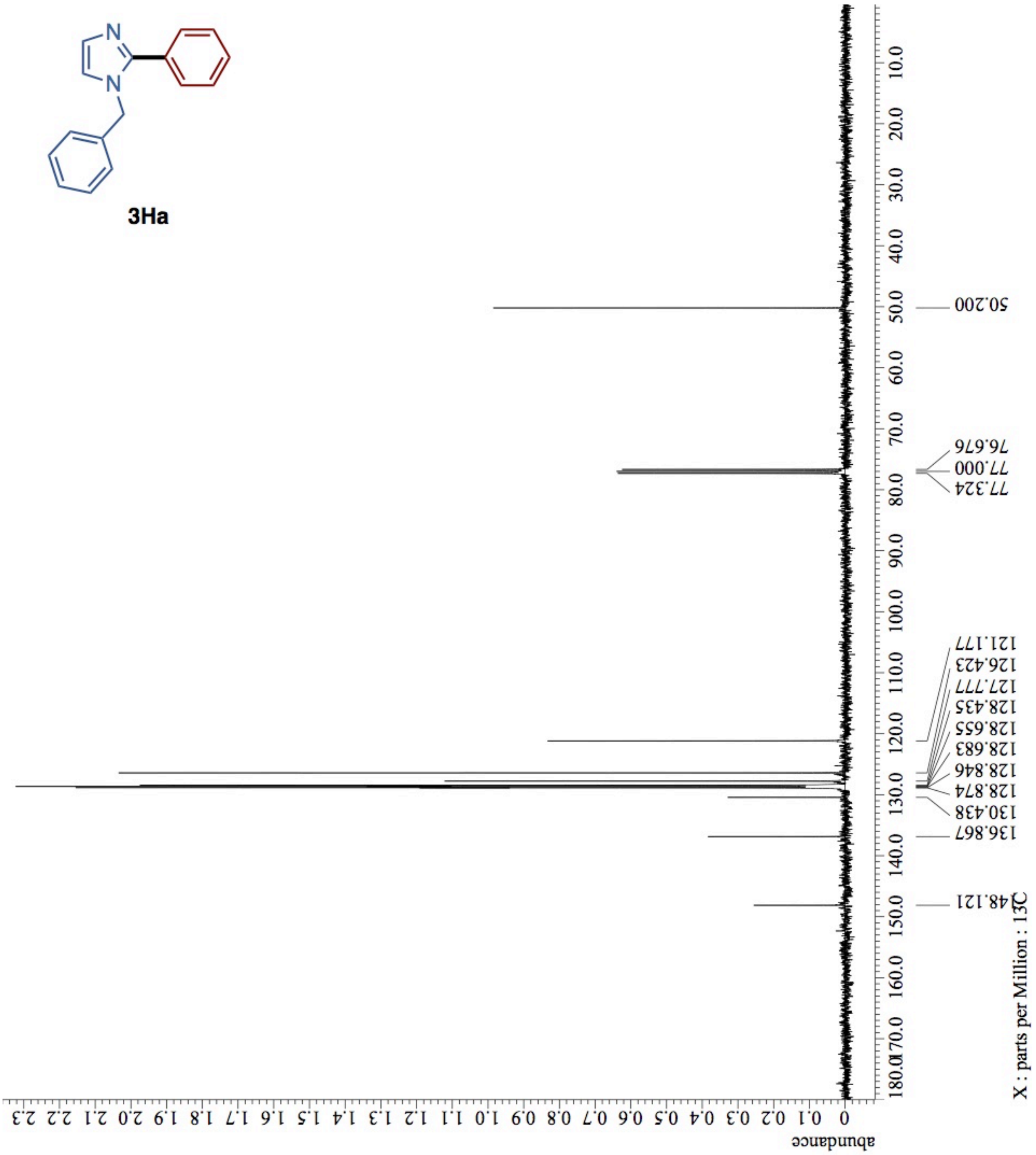

^{1}H NMR of **3lb** (400 MHz, $CDCl_3$)

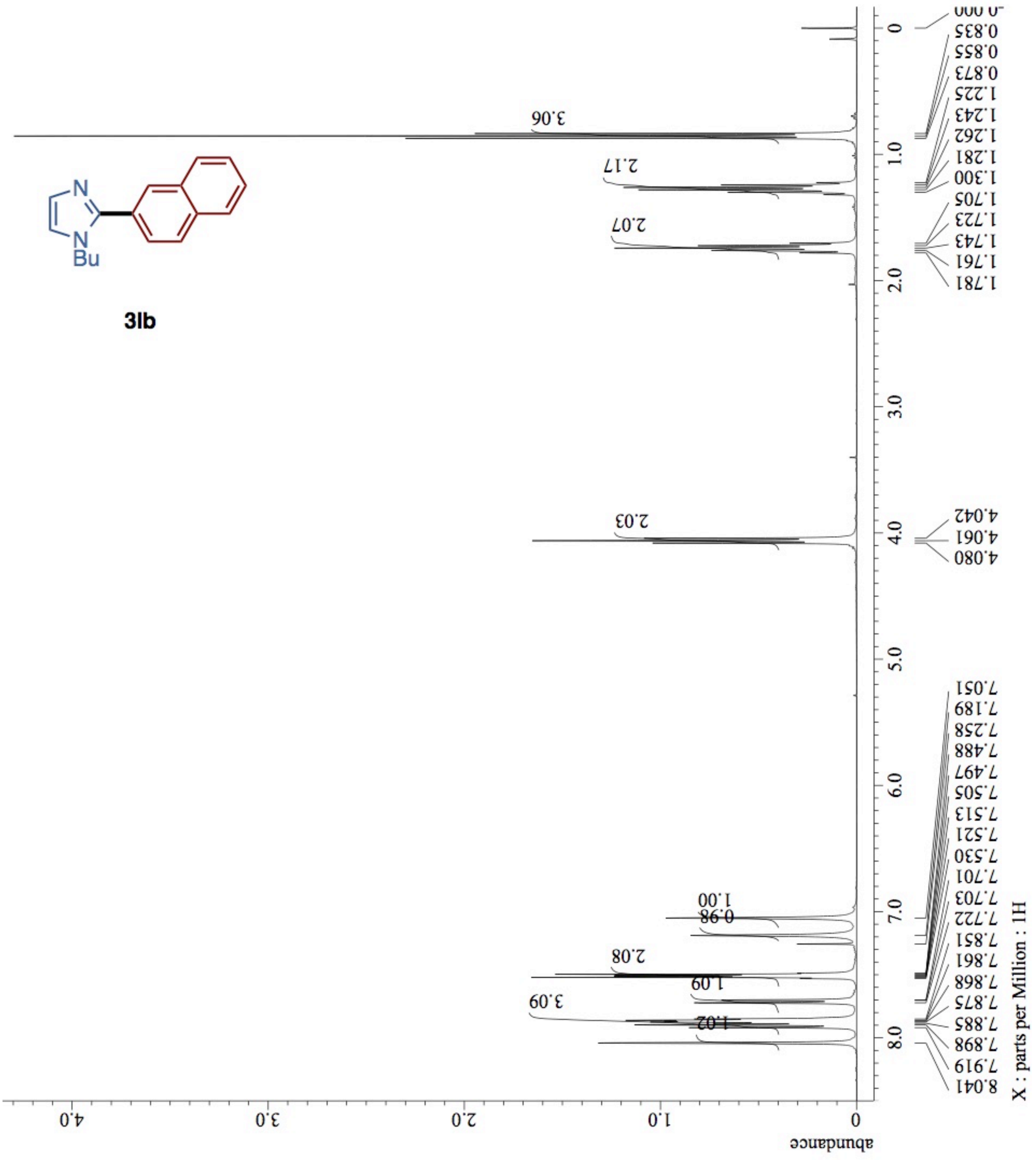

^{13}C NMR of **3lb** (100 MHz, CDCl$_3$)

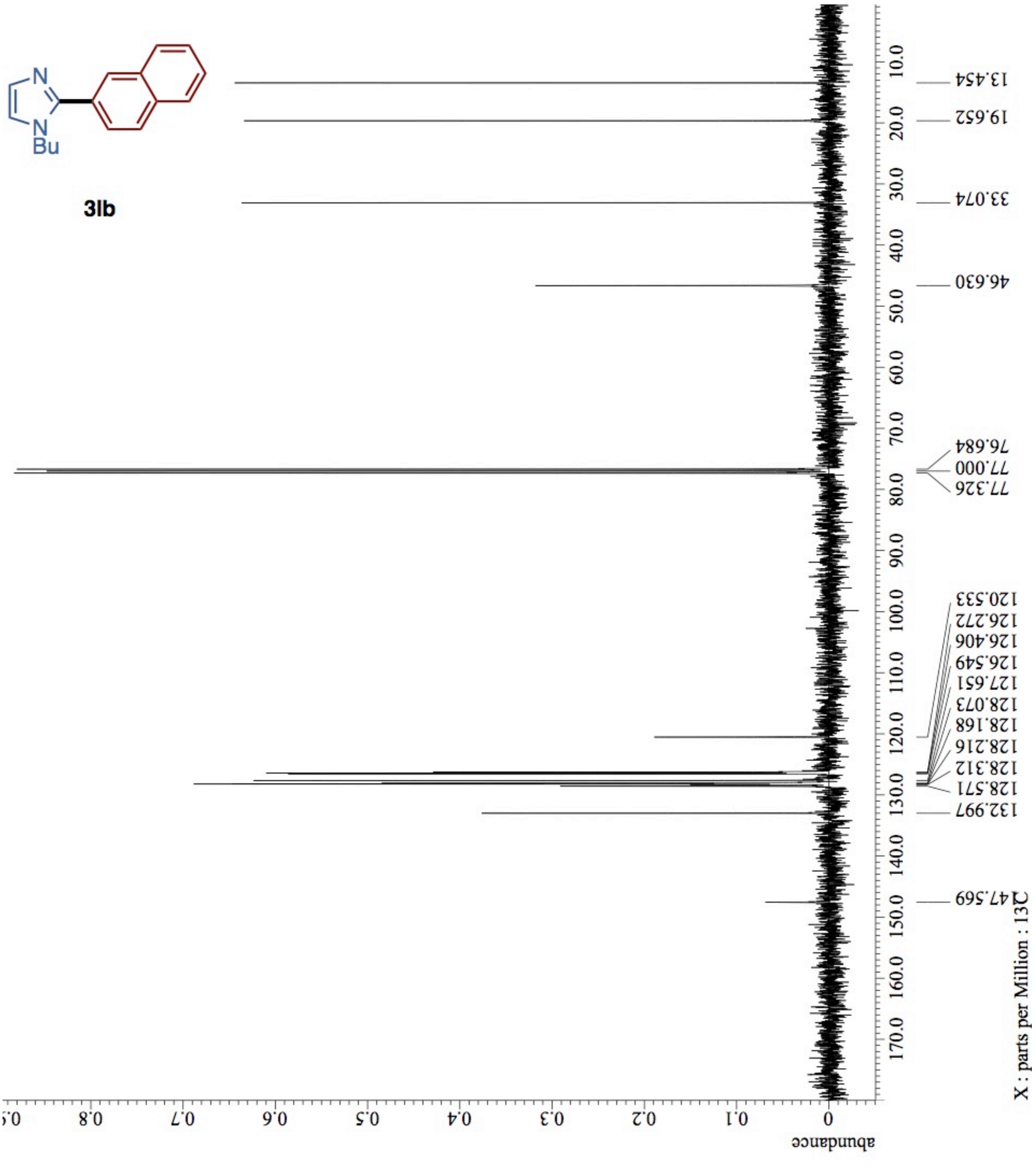

^{1}H NMR of **3Ja** (400 MHz, $CDCl_3$)

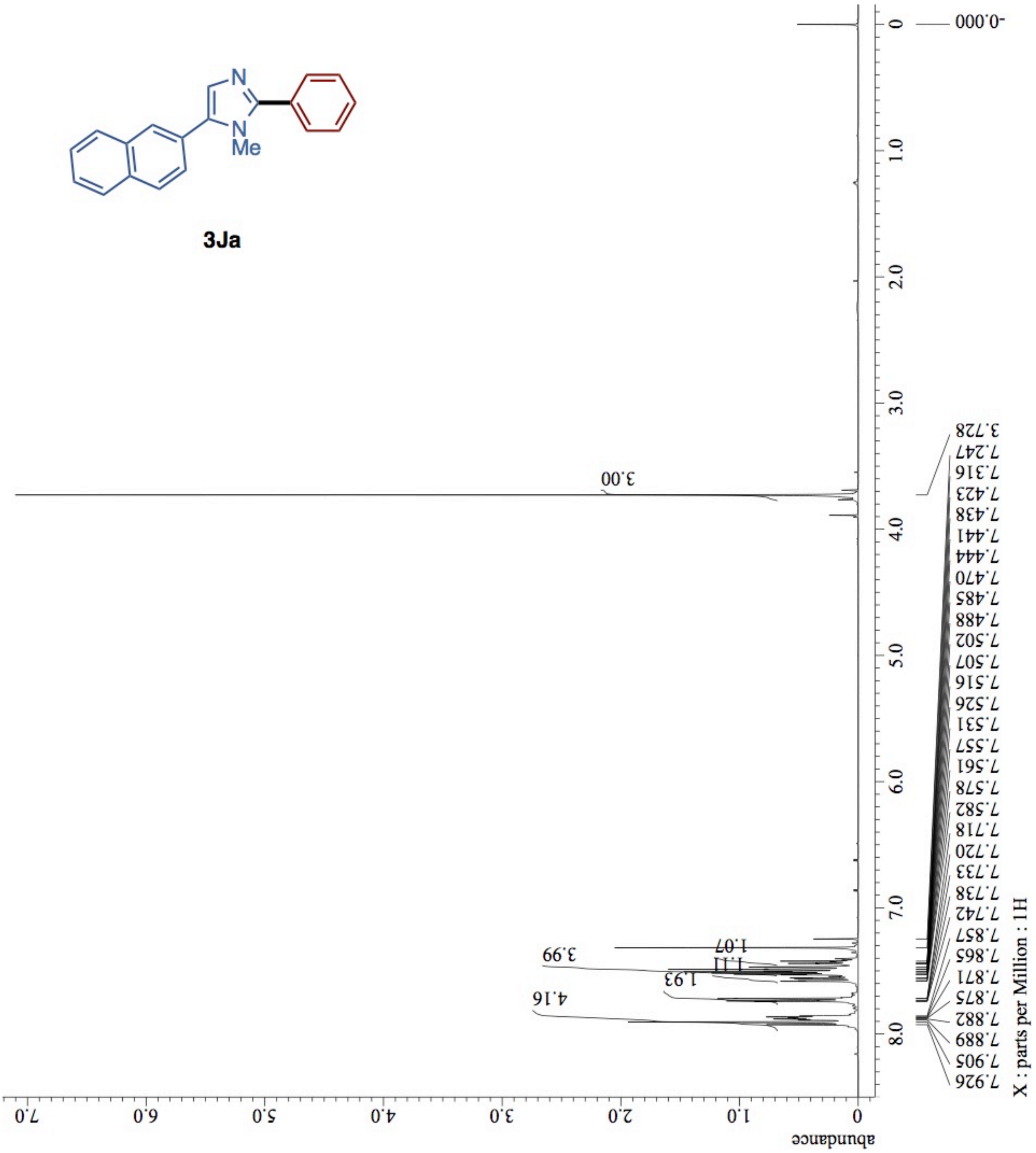

^{13}C NMR of **3Ja** (100 MHz, $CDCl_3$)

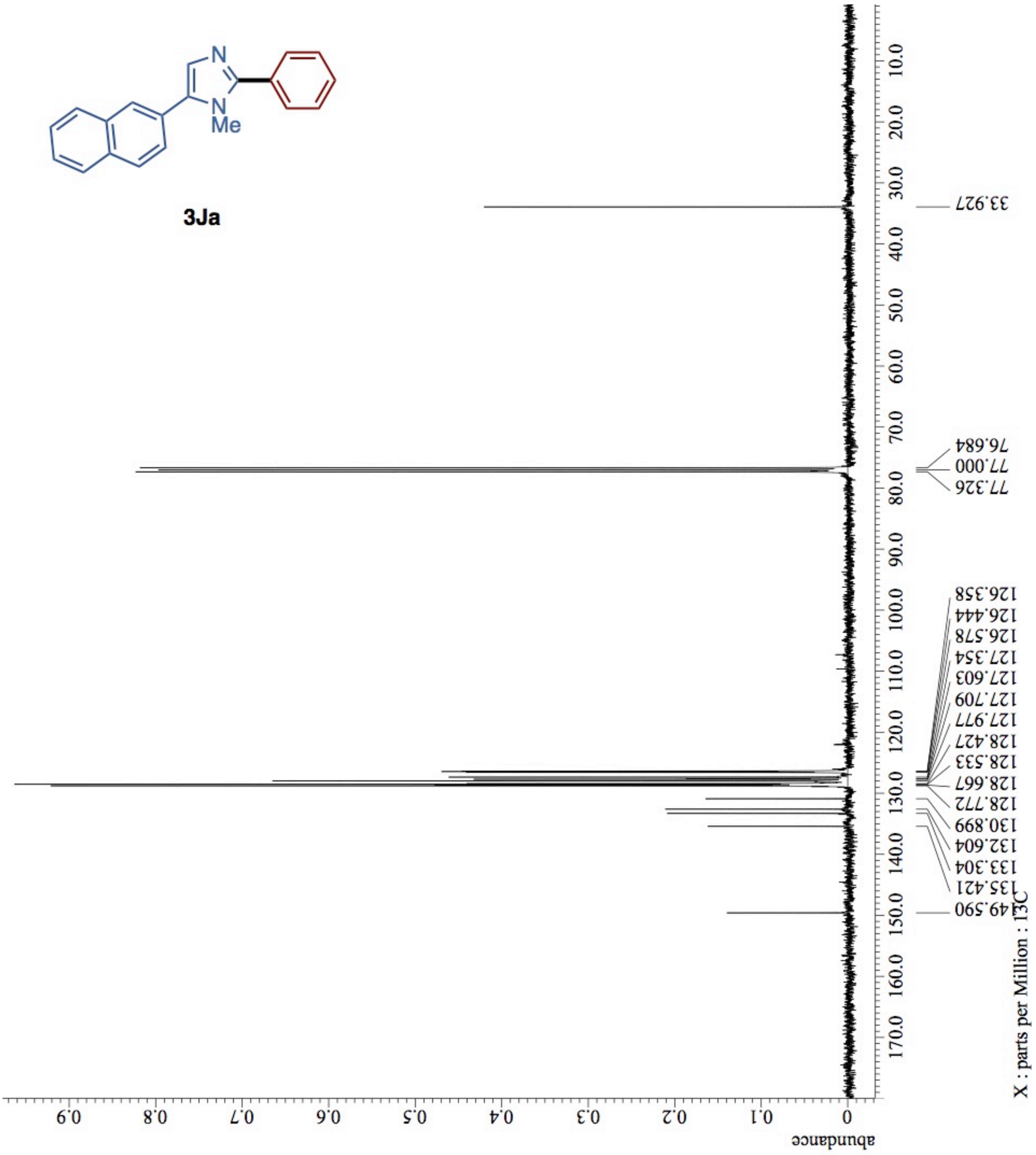

3Ja

^{1}H NMR of **3Ka** (400 MHz, $CDCl_3$)

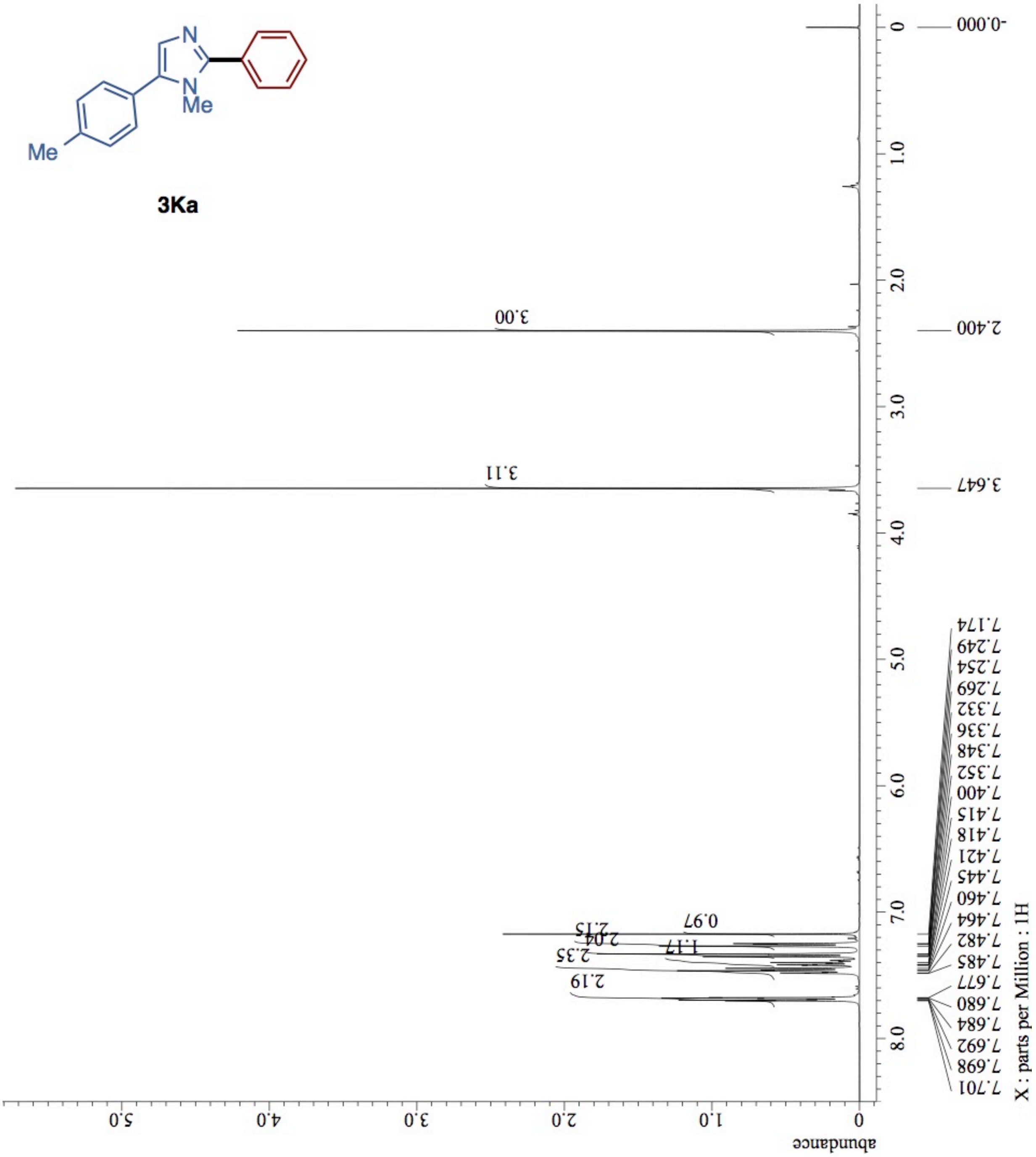

^{13}C NMR of **3Ka** (100 MHz, $CDCl_3$)

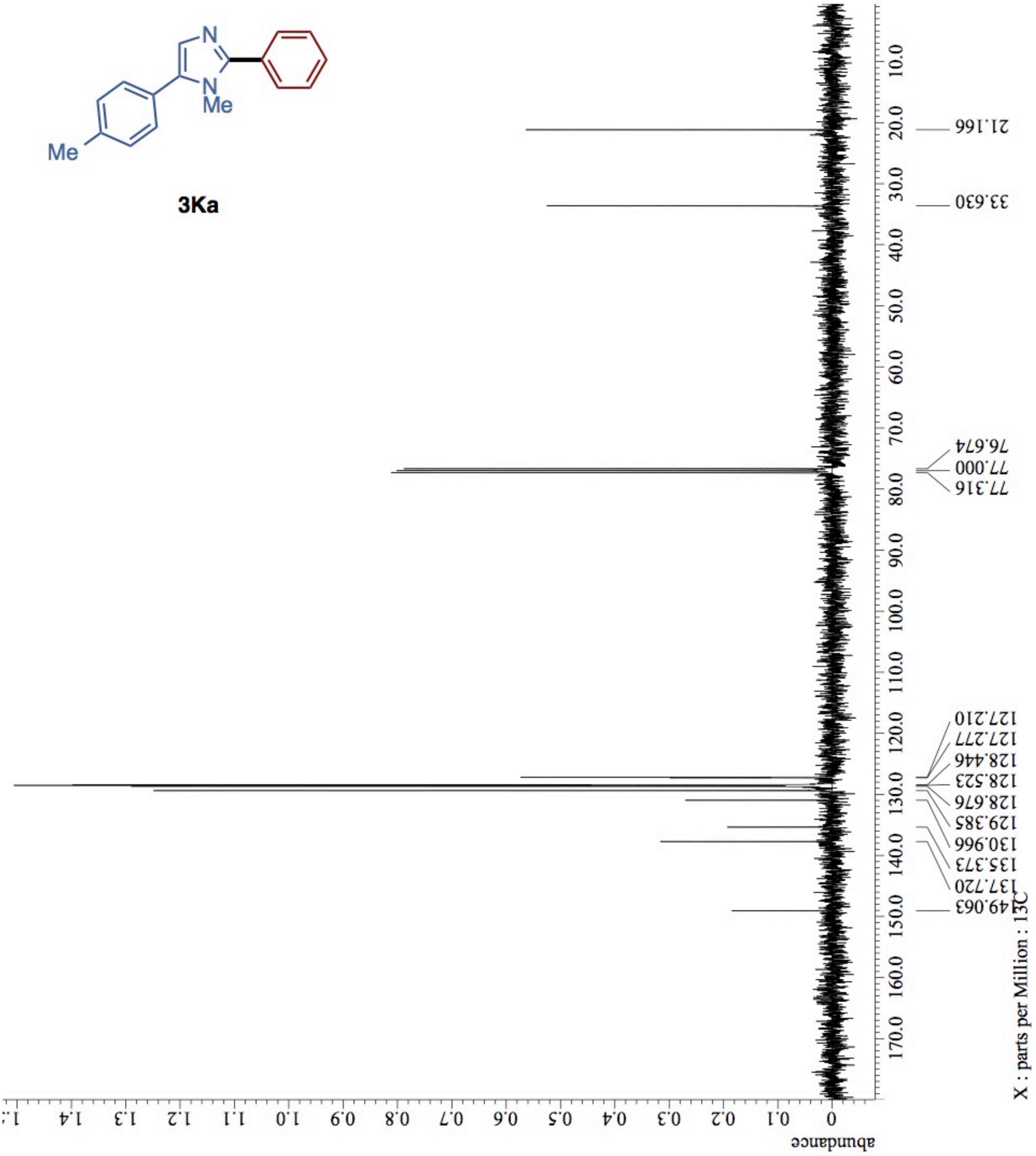

3Ka

^{1}H NMR of **3La** (400 MHz, $CDCl_3$)

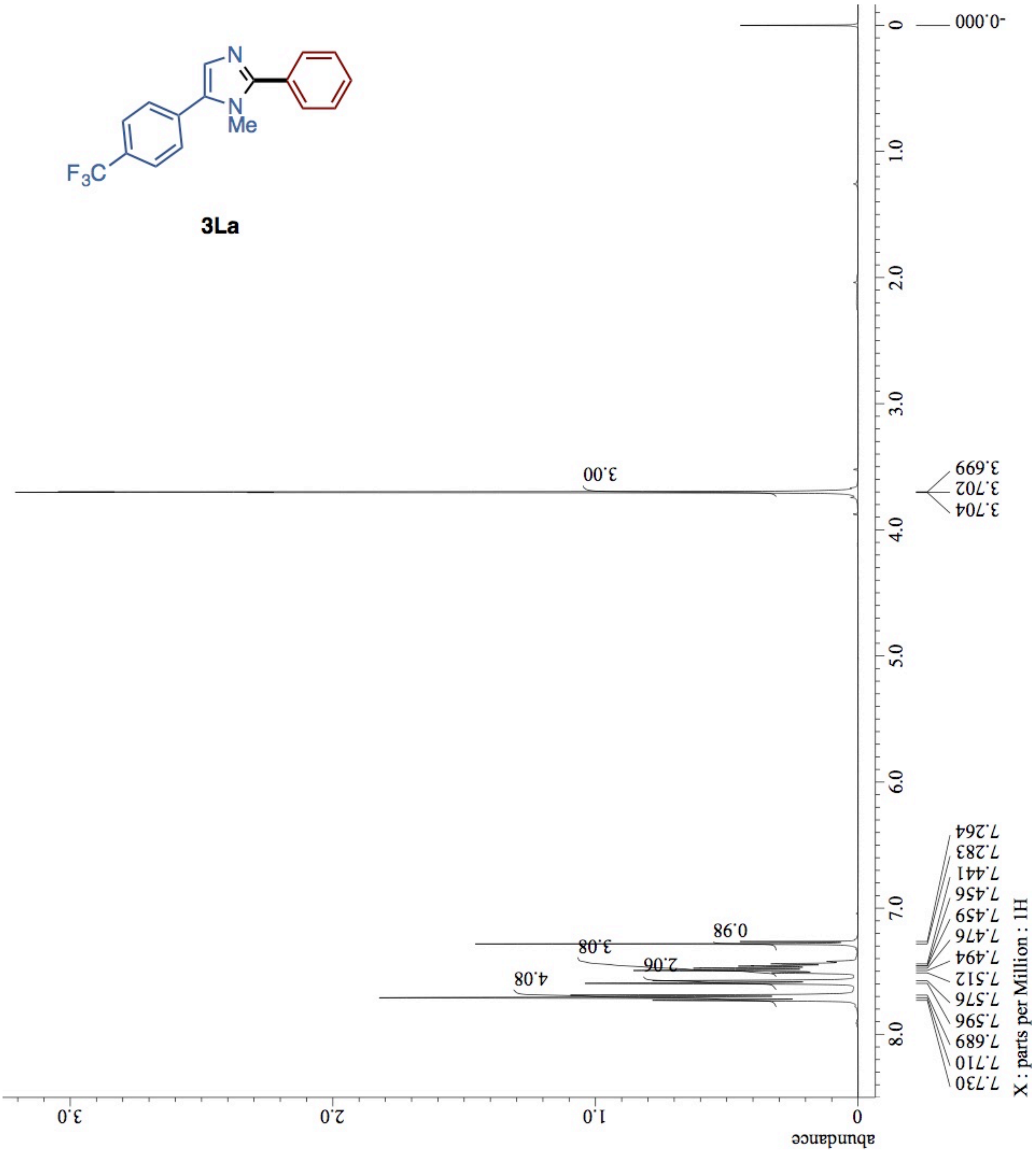

^{13}C NMR of **3La** (100 MHz, $CDCl_3$)

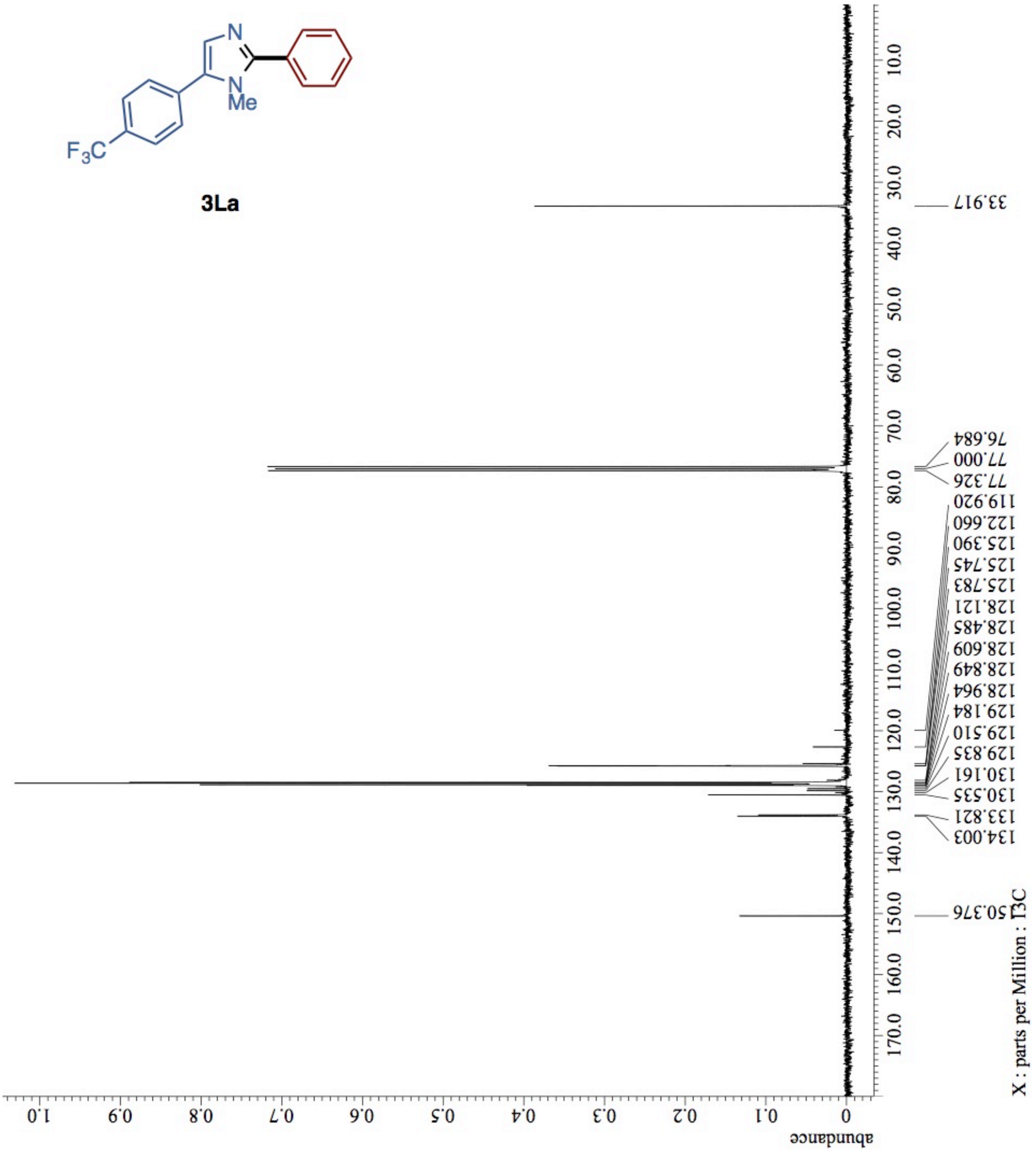

^{1}H NMR of **3Ma** (400 MHz, $CDCl_3$)

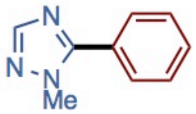

3Ma

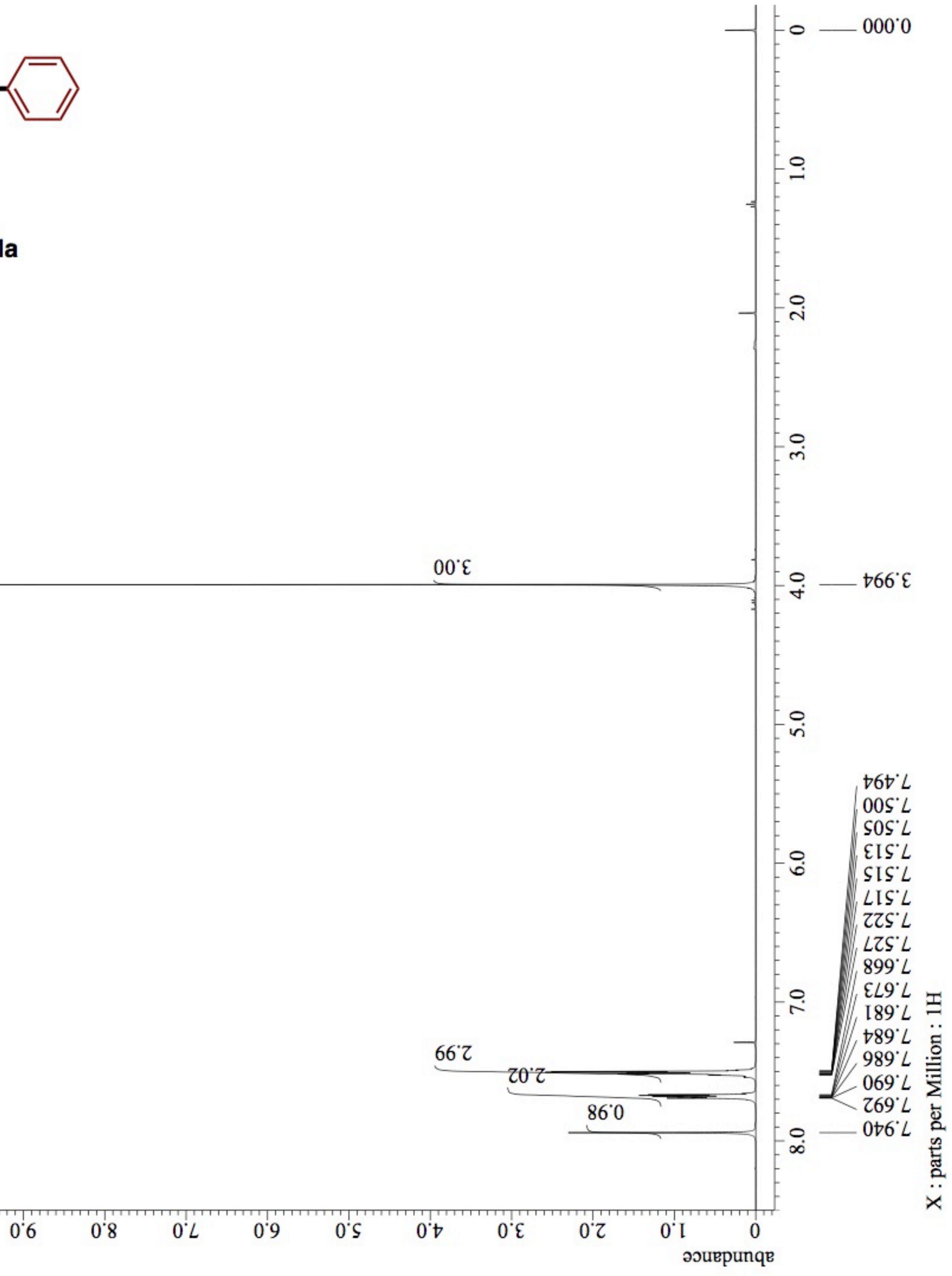

^{13}C NMR of **3Ma** (100 MHz, CDCl$_3$)

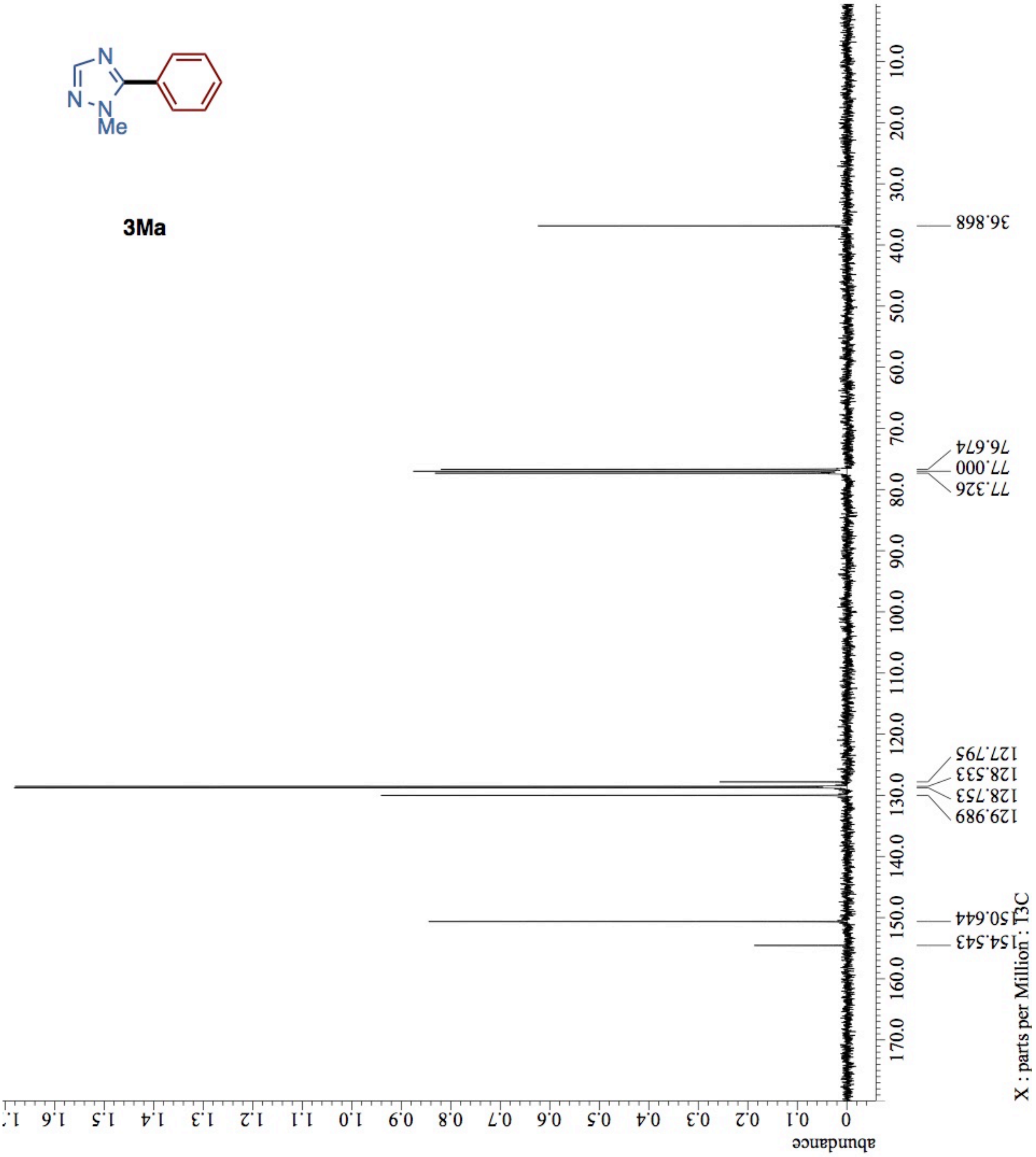

^{1}H NMR of **3Nb** (400 MHz, CDCl$_3$)

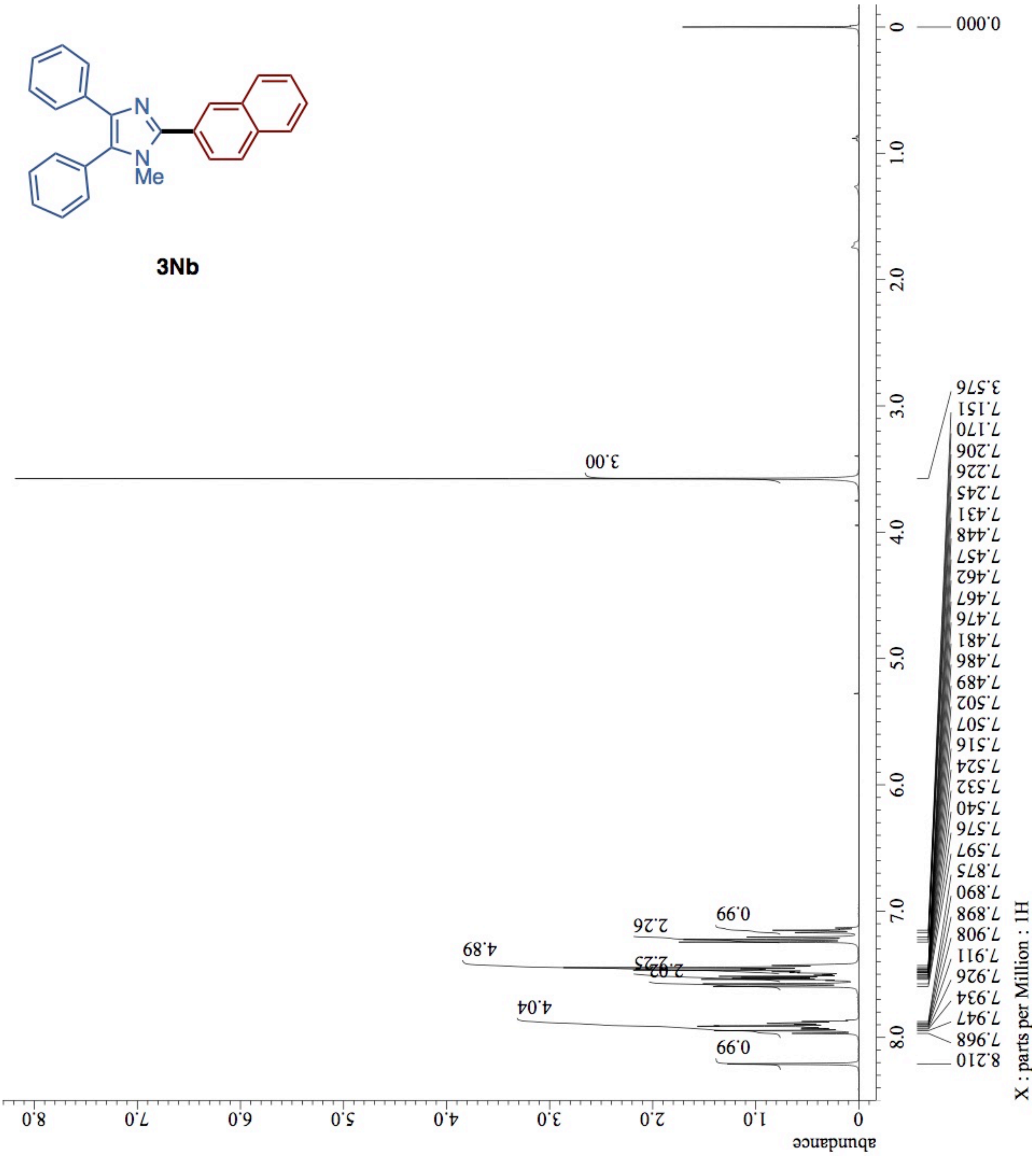

^{13}C NMR of **3Nb** (100 MHz, $CDCl_3$)

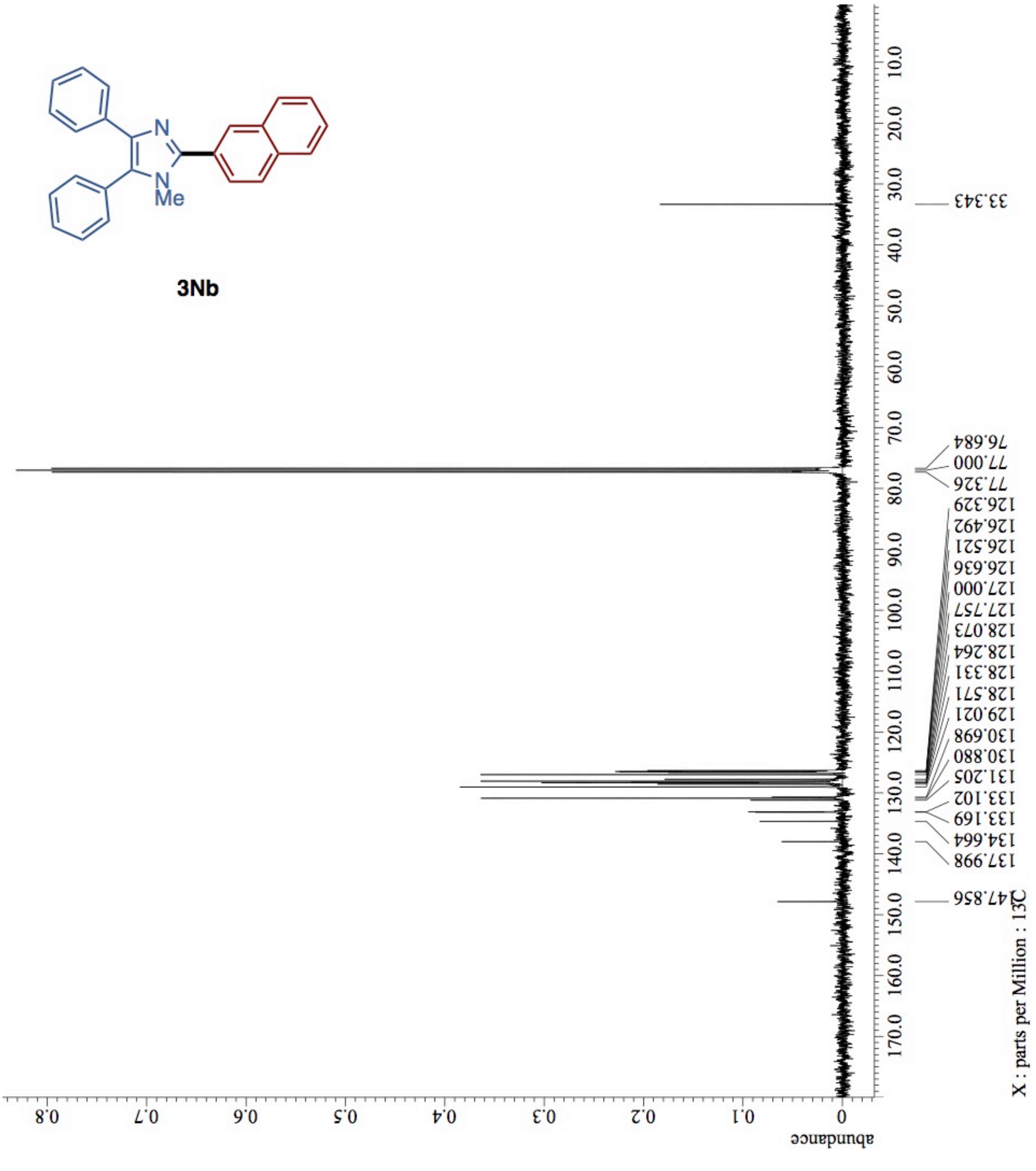

^{1}H NMR of **3Ob** (400 MHz, CDCl$_3$)

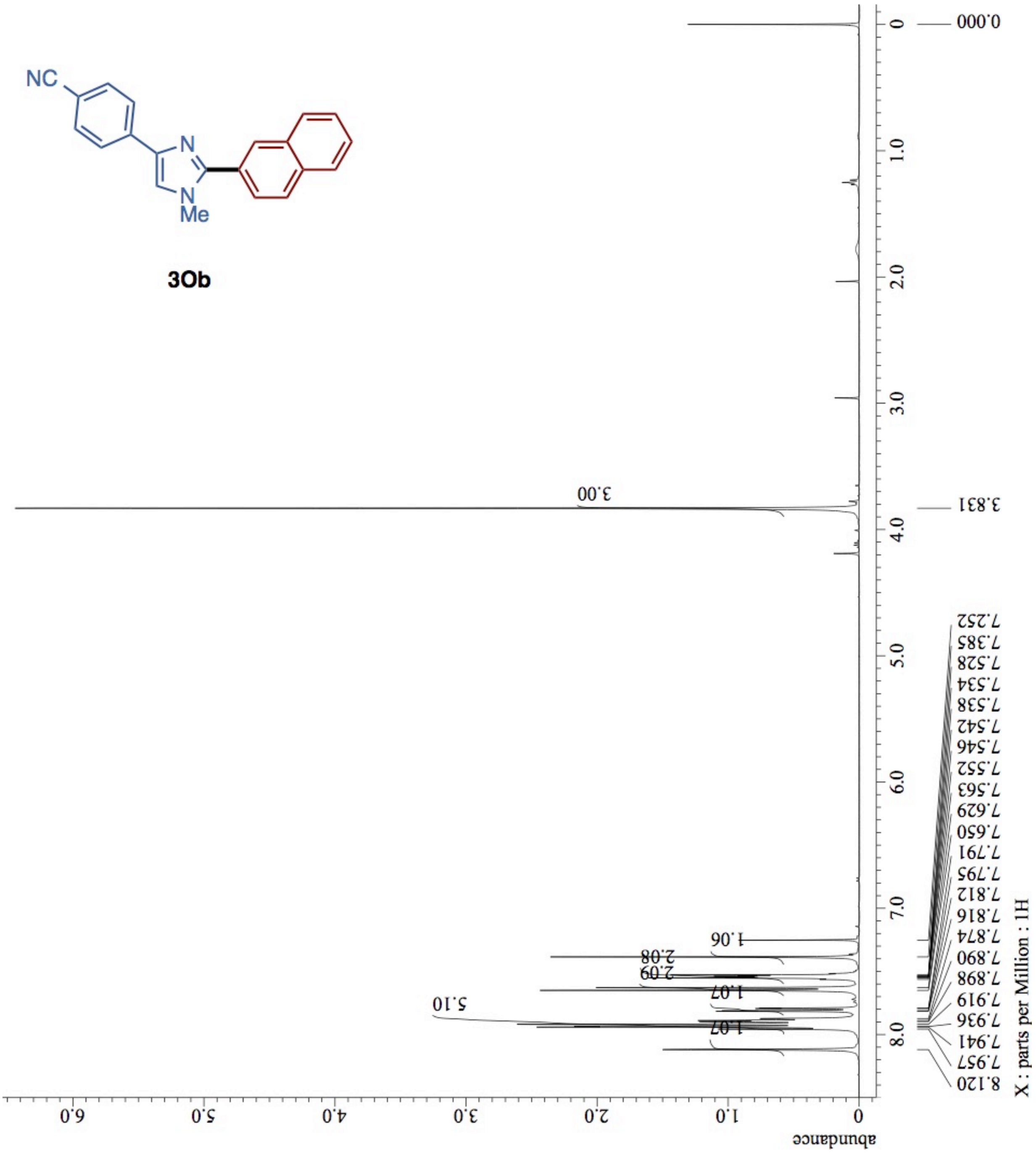

3Ob

^{13}C NMR of **3Ob** (100 MHz, $CDCl_3$)

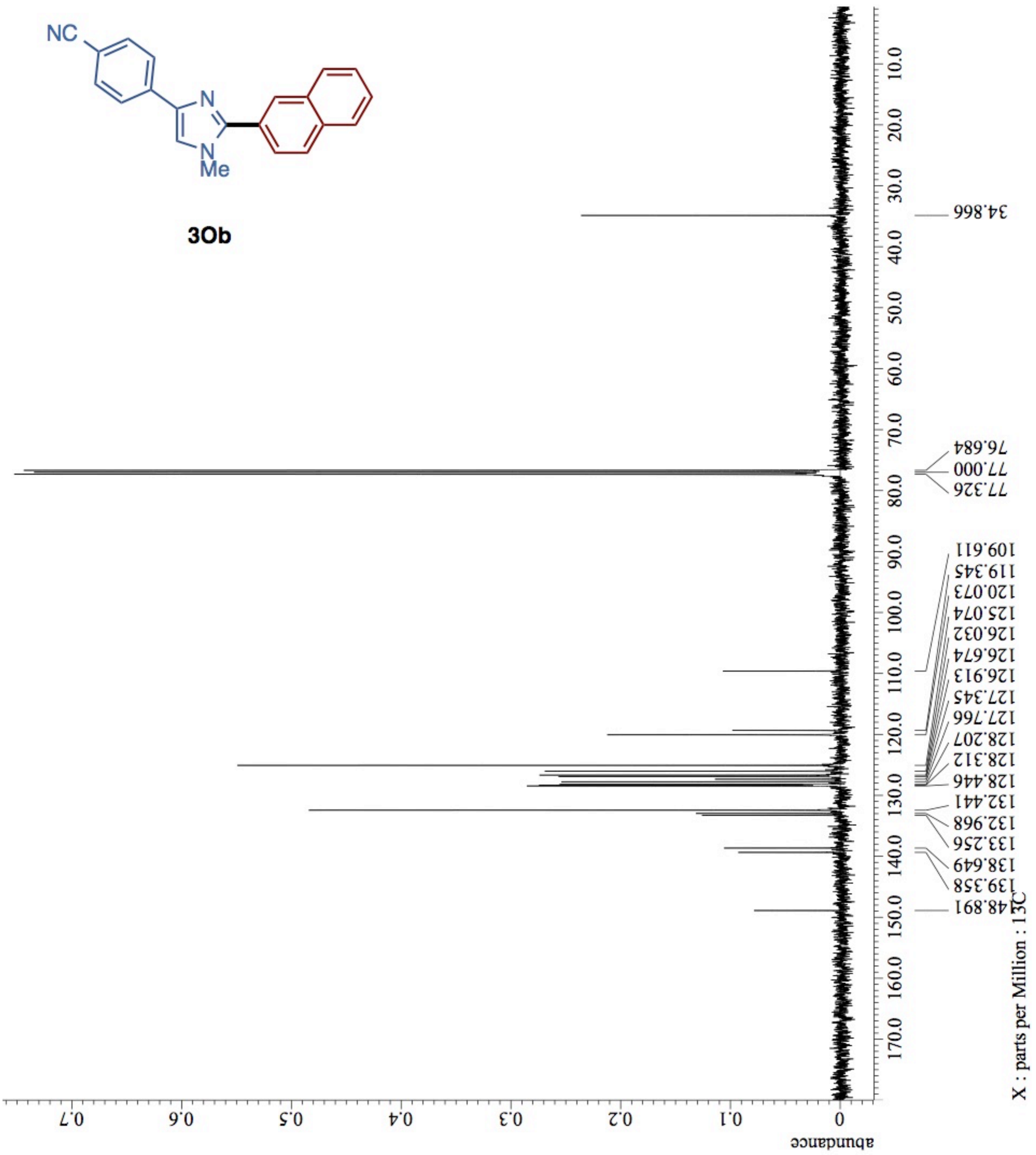

^{1}H NMR of **3Pb** (400 MHz, $CDCl_3$)

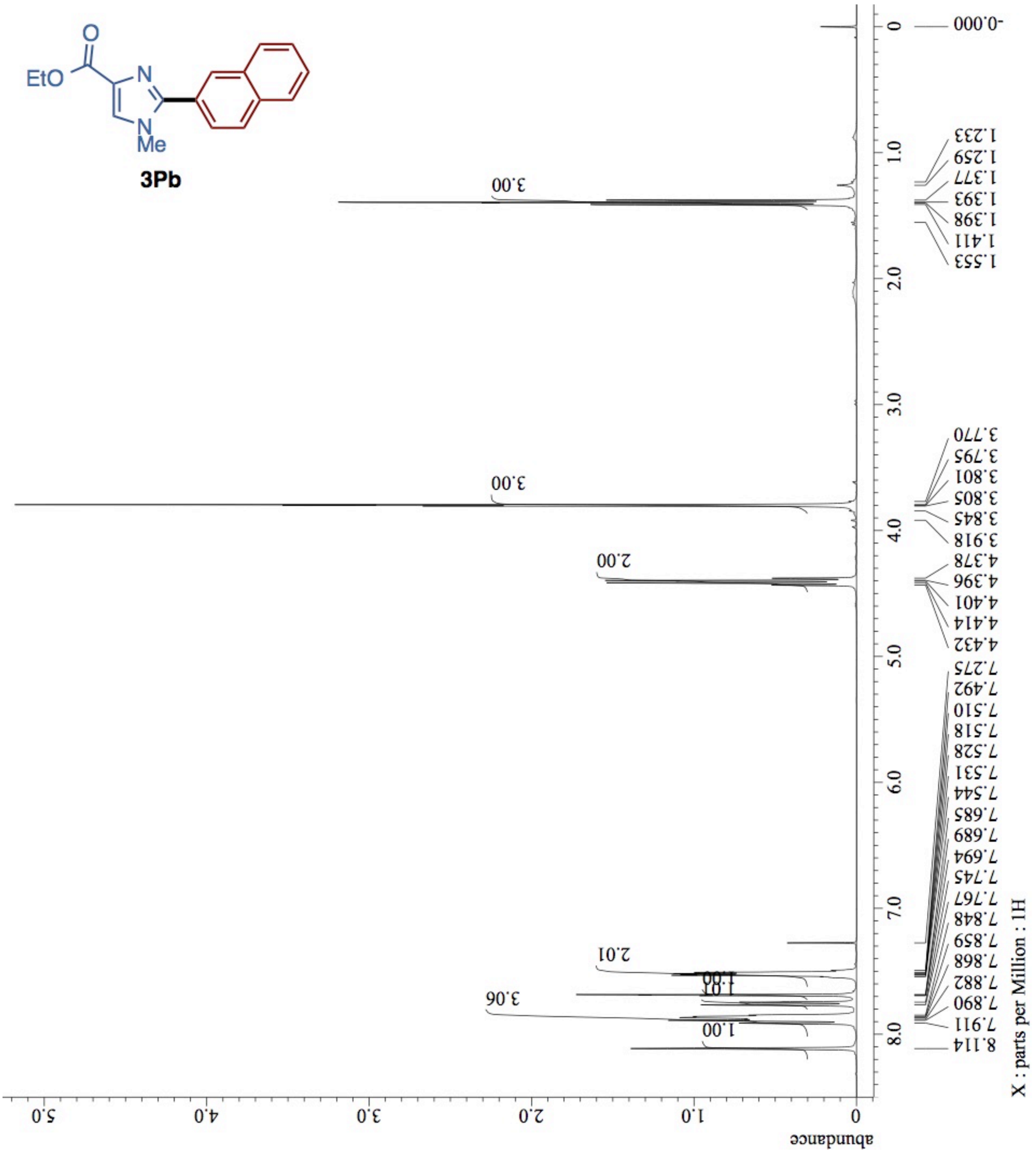

^{13}C NMR of **3Pb** (100 MHz, $CDCl_3$)

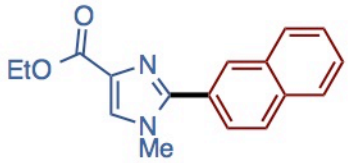

3Pb

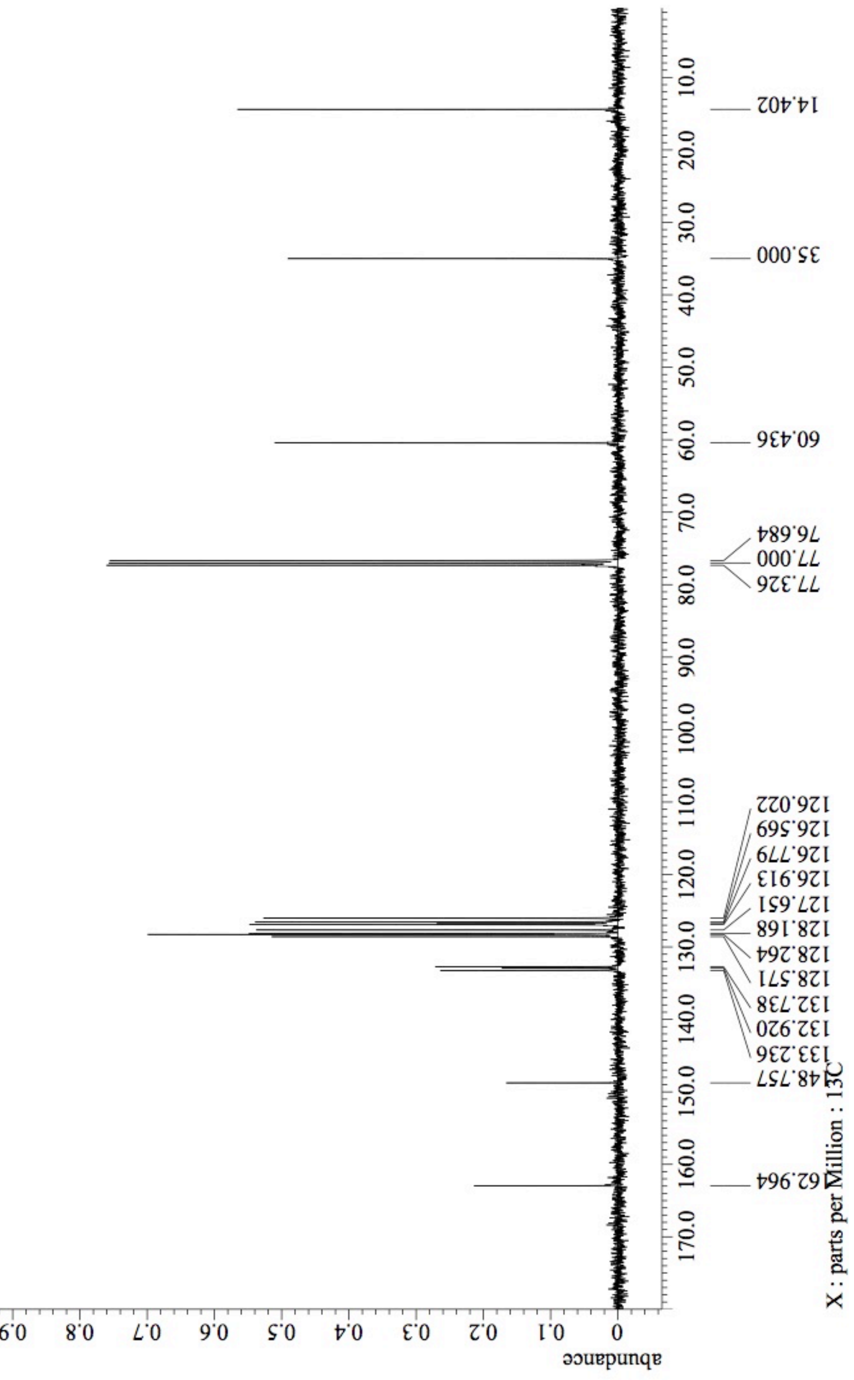

^{1}H NMR of **3Pb'** (400 MHz, $CDCl_3$)

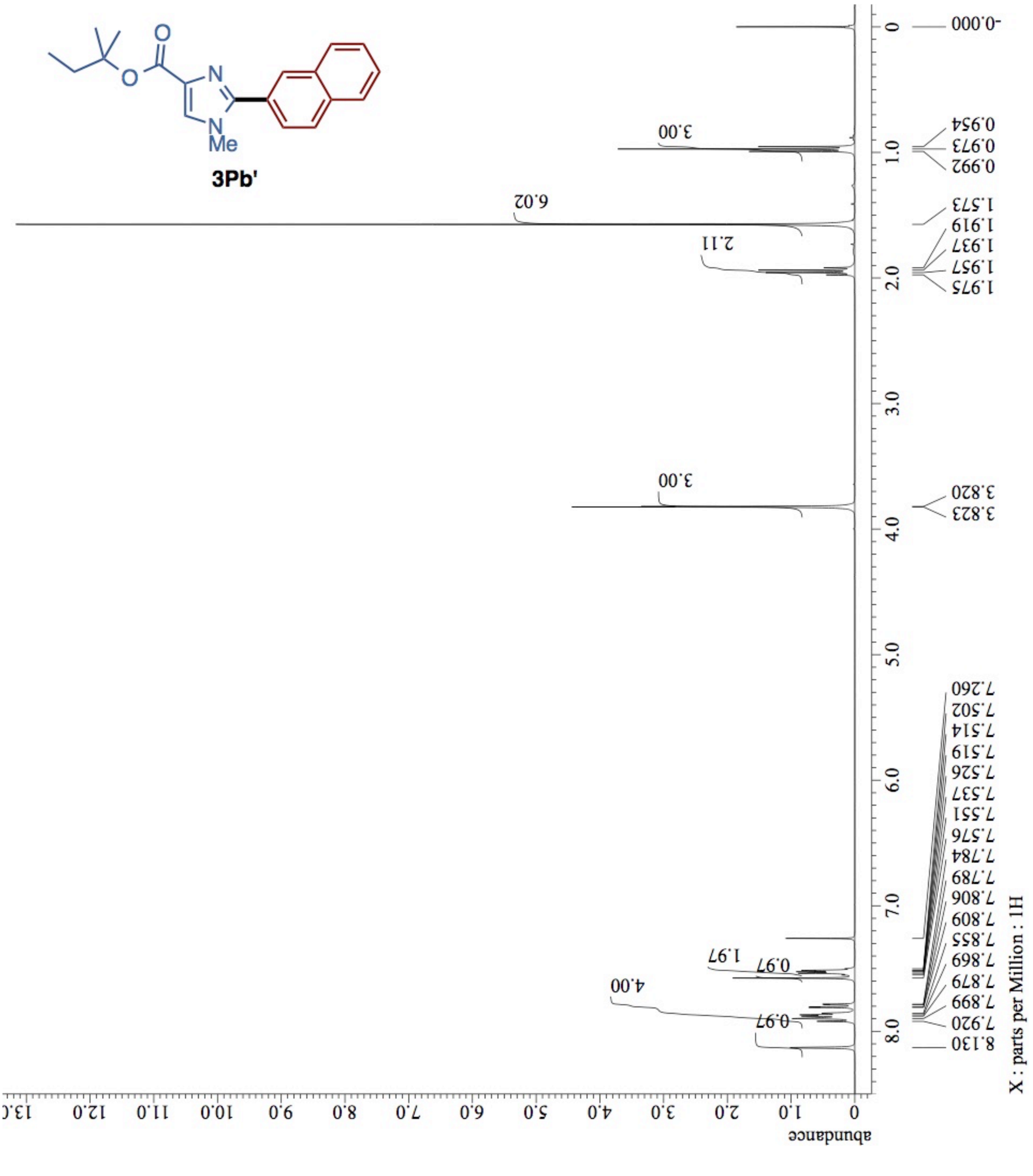

3Pb'

^{13}C NMR of **3Pb'** (100 MHz, $CDCl_3$)

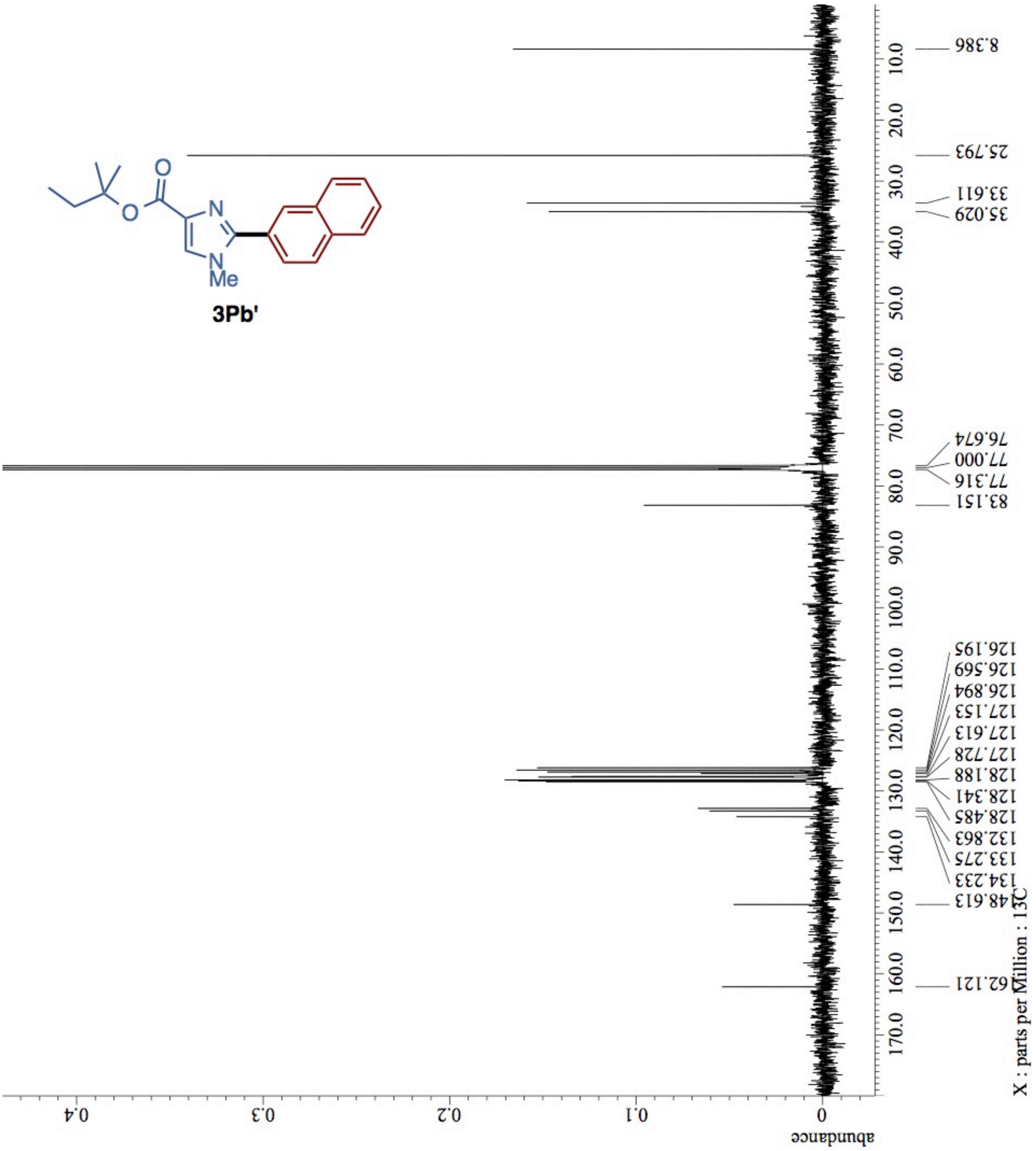

^{1}H NMR of **3Ac** (400 MHz, $CDCl_3$)

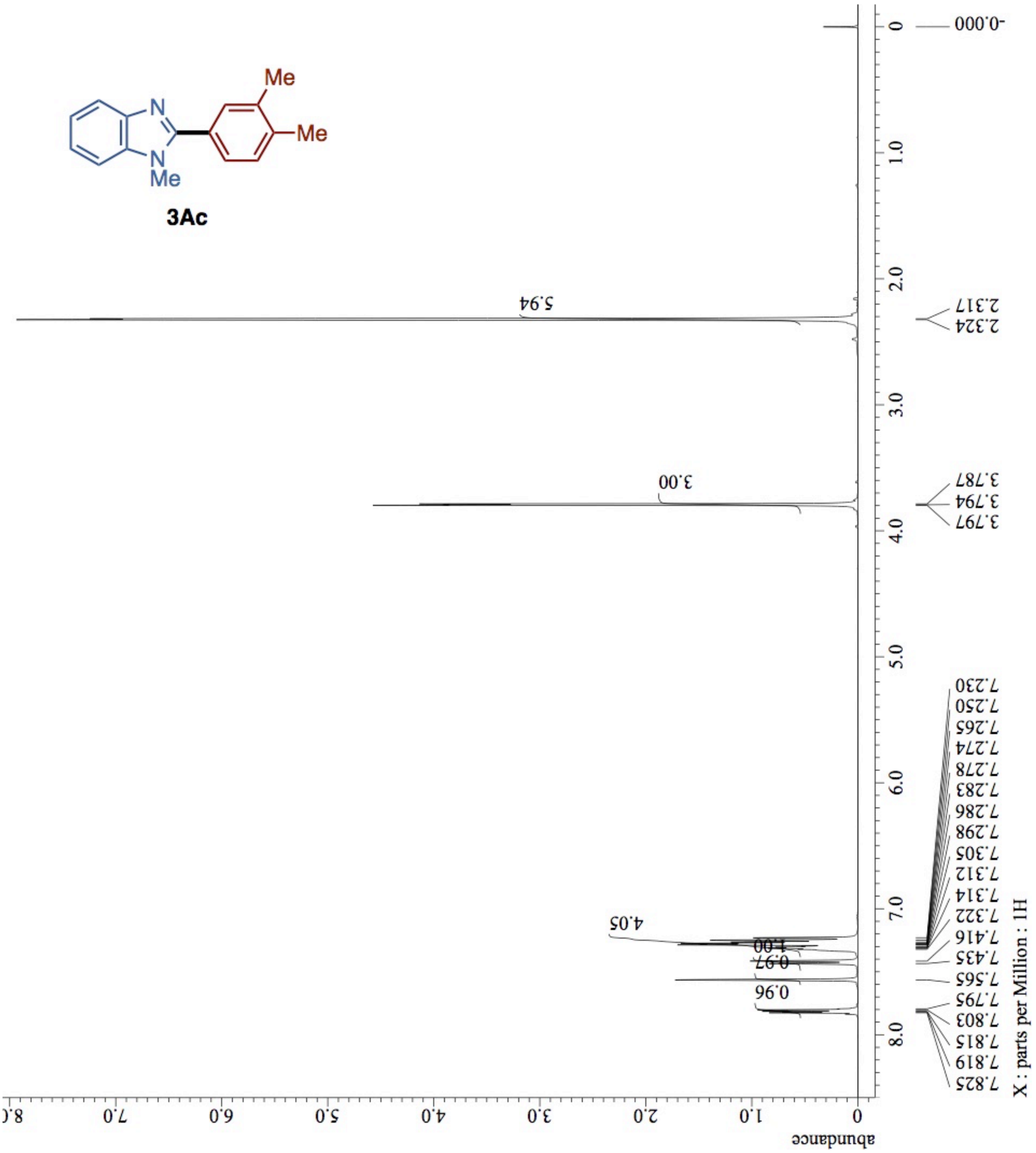

^{13}C NMR of **3Ac** (100 MHz, $CDCl_3$)

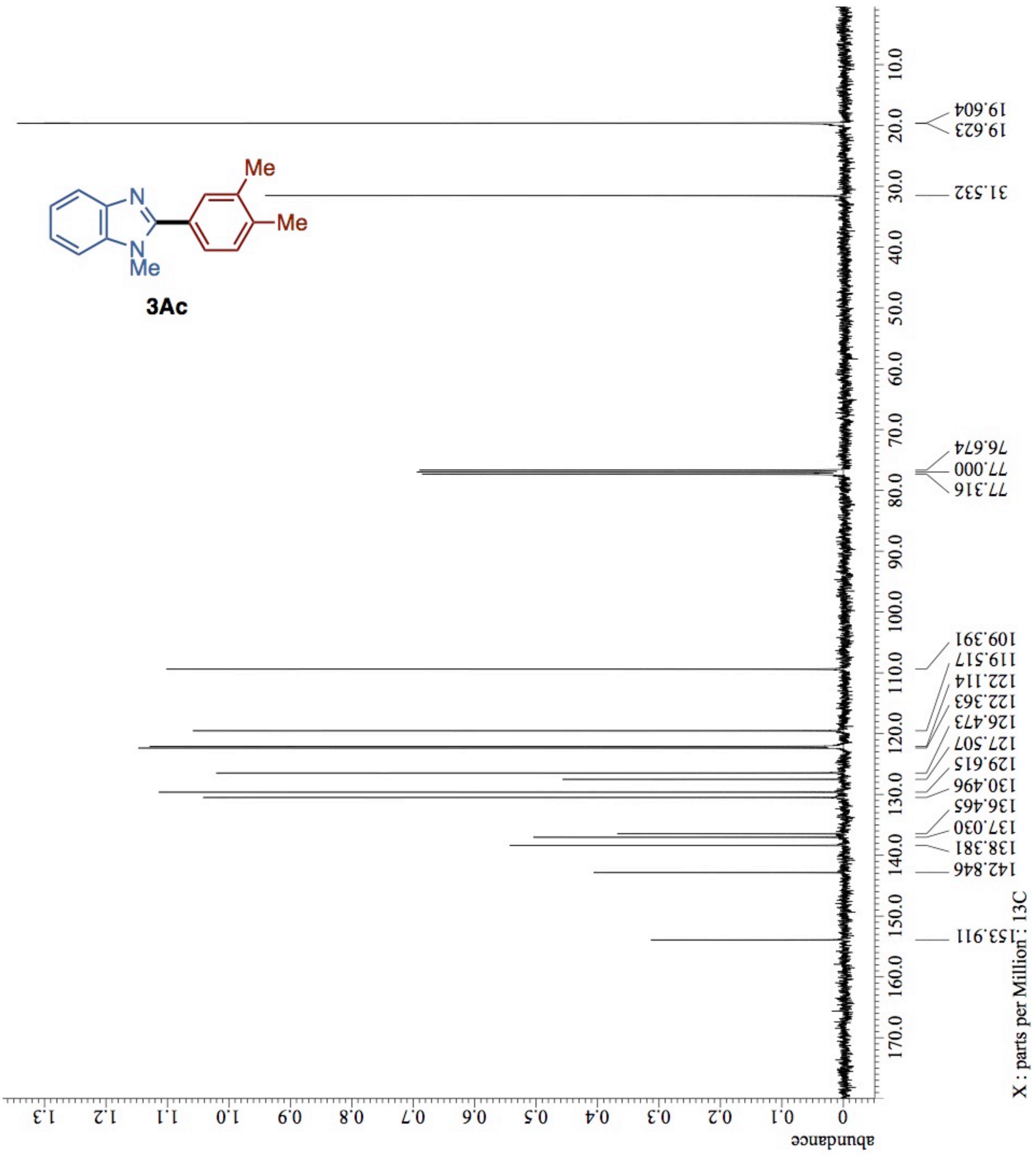

^{1}H NMR of **3Ad** (400 MHz, $CDCl_3$)

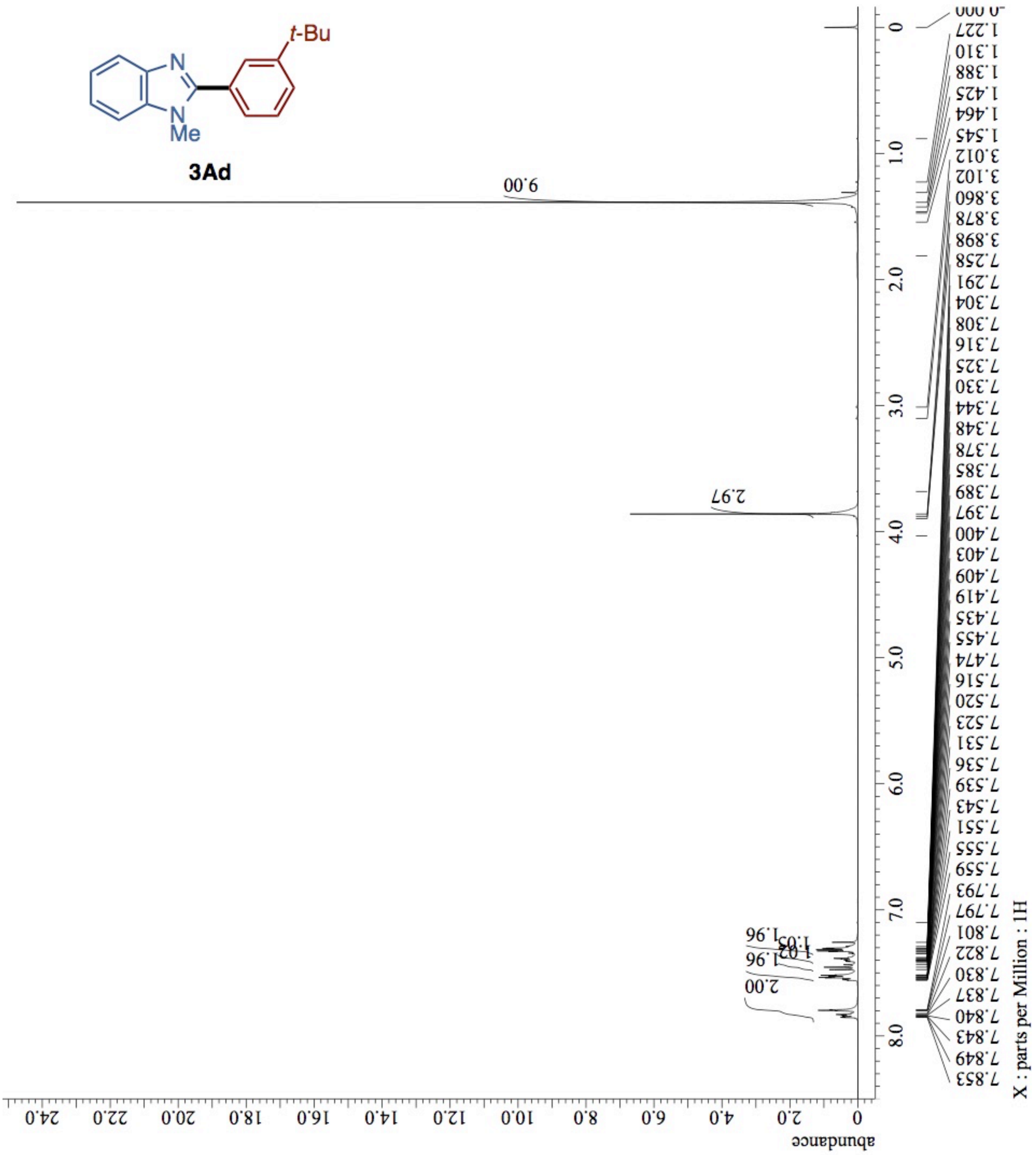

^{13}C NMR of **3Ad** (100 MHz, $CDCl_3$)

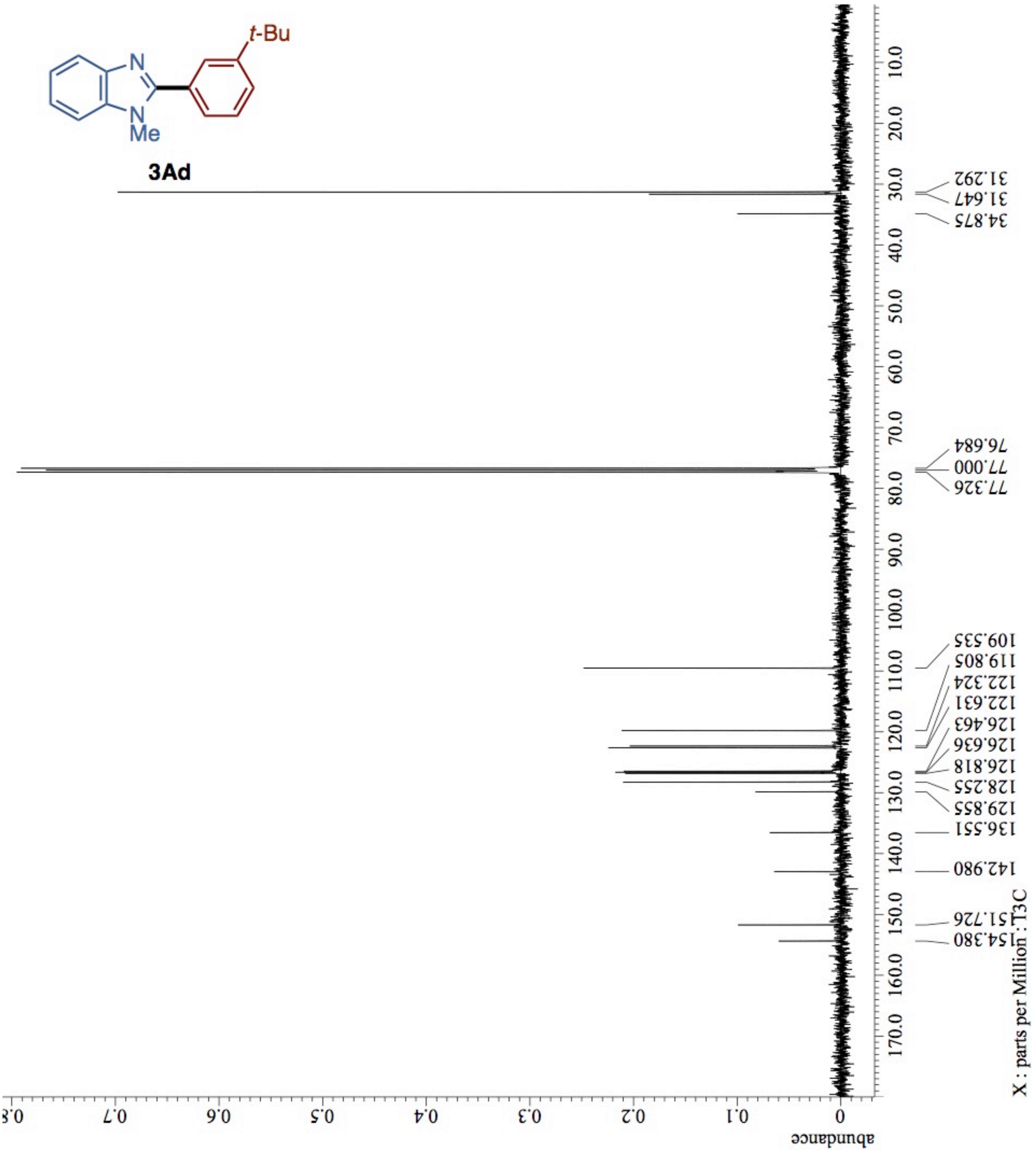

^{1}H NMR of **3Ae** (400 MHz, $CDCl_3$)

3Ae

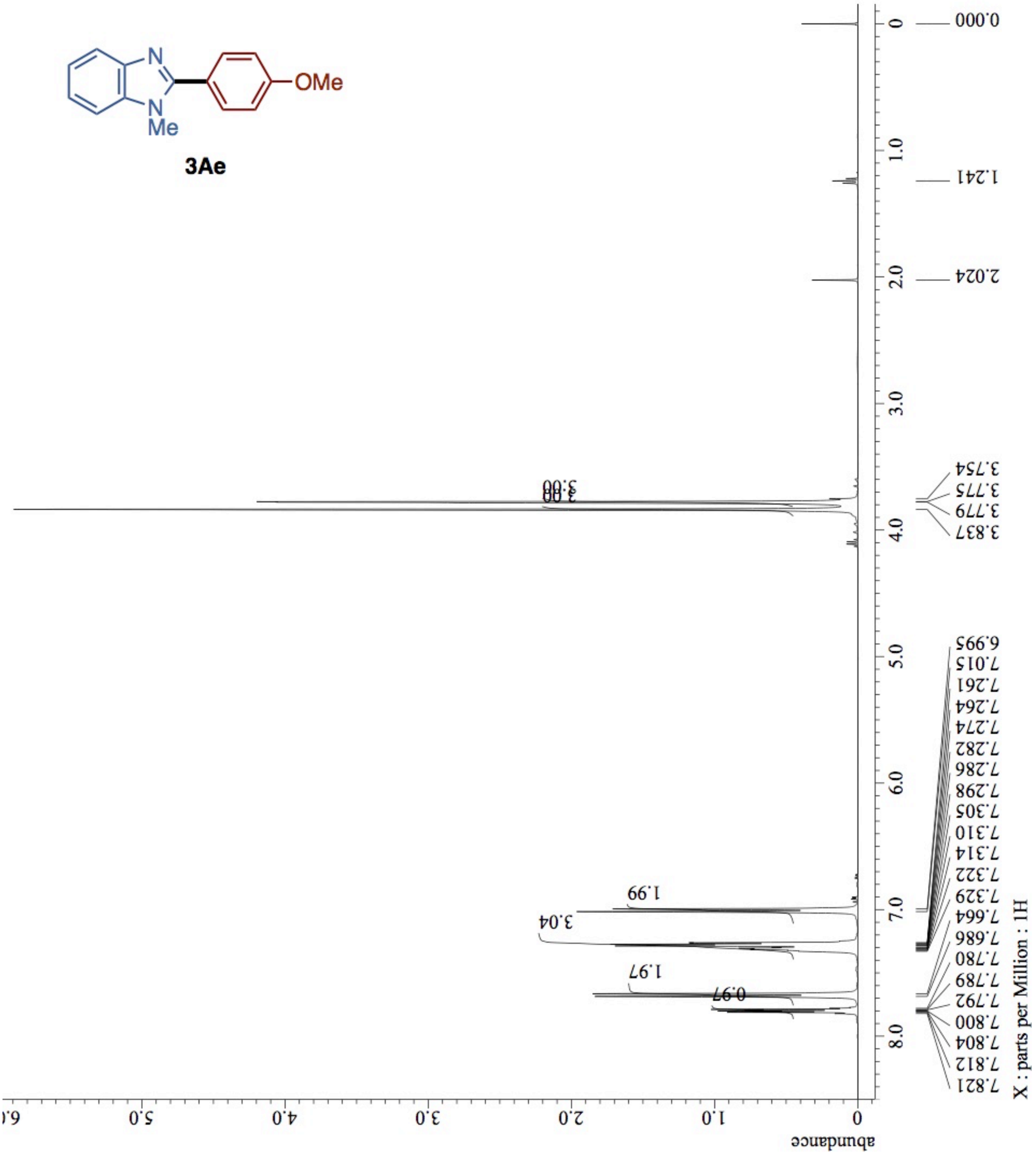

^{13}C NMR of **3Ae** (100 MHz, $CDCl_3$)

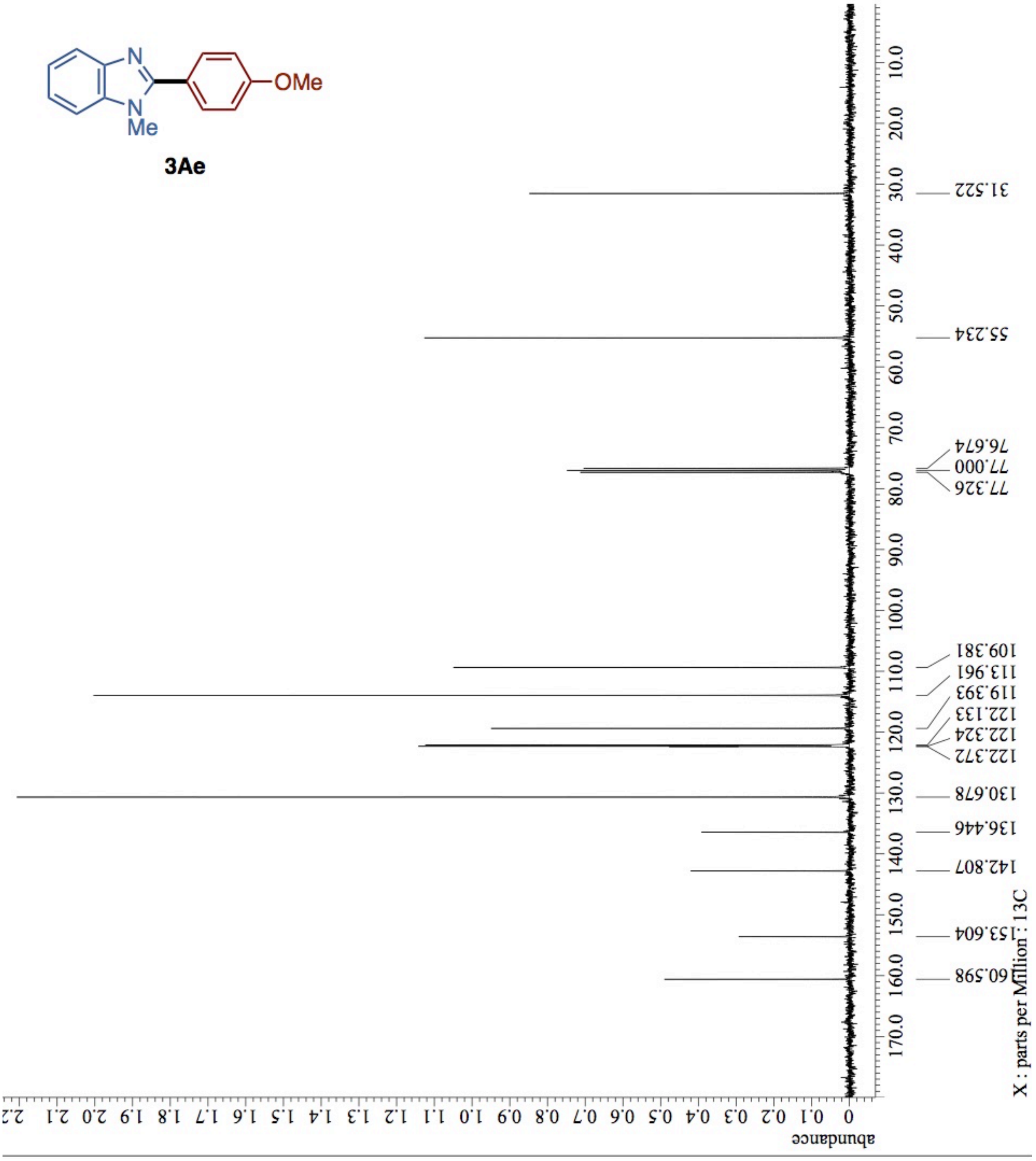

^{1}H NMR of **3Af** (400 MHz, CDCl$_3$)

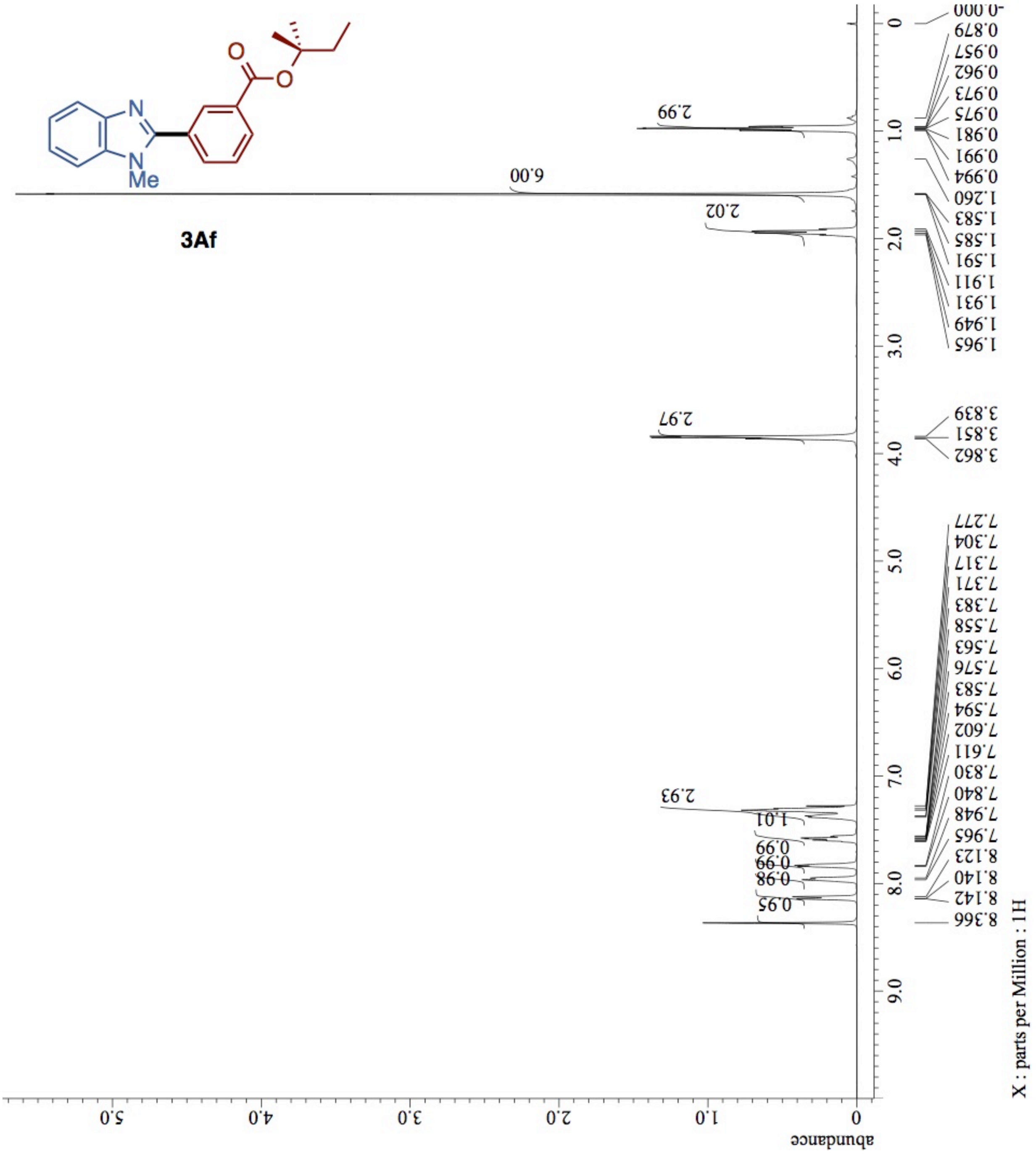

^{13}C NMR of **3Af** (100 MHz, CDCl$_3$)

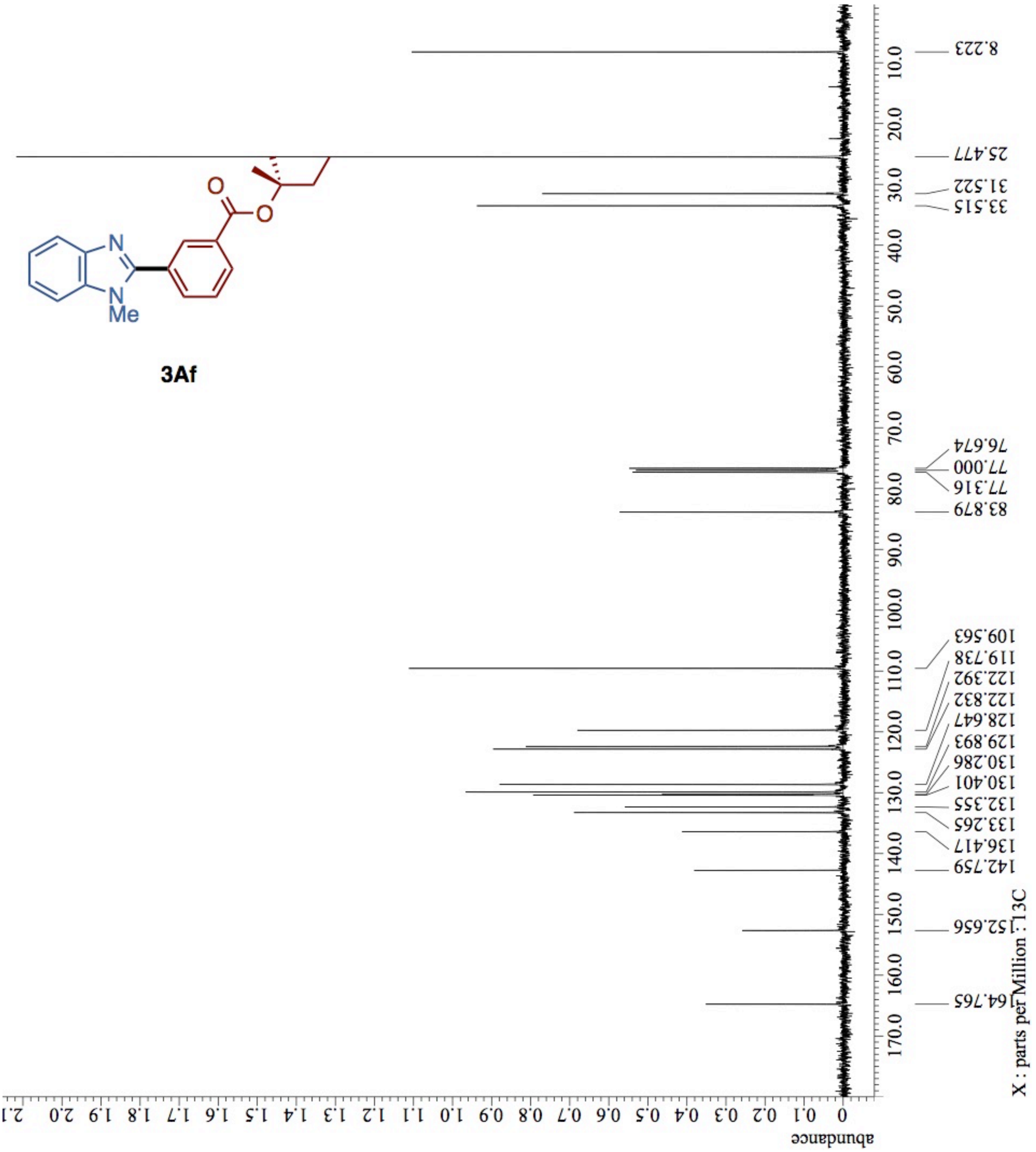

^{1}H NMR of **3Ag** (400 MHz, $CDCl_3$)

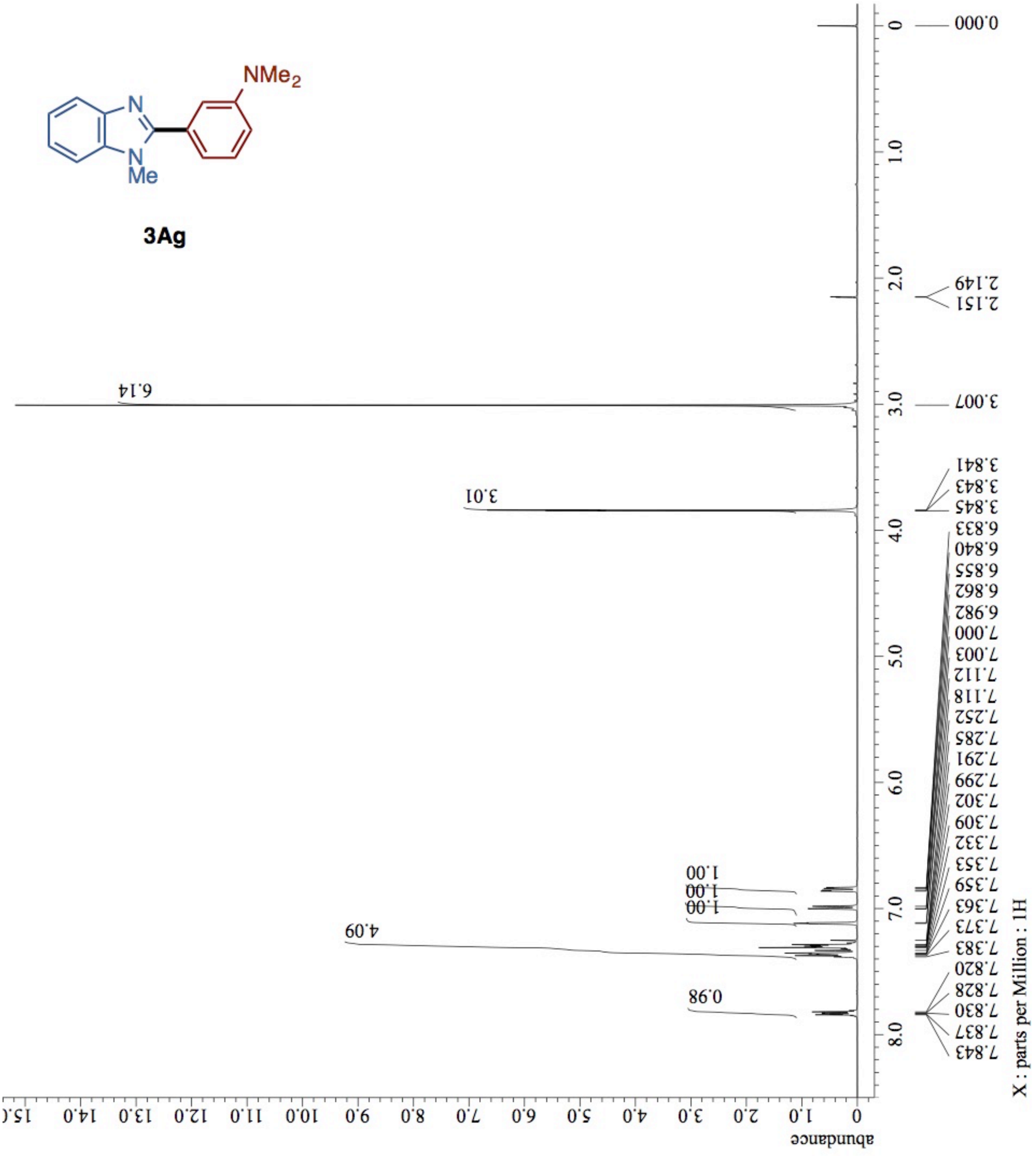

3Ag

^{13}C NMR of **3Ag** (100 MHz, $CDCl_3$)

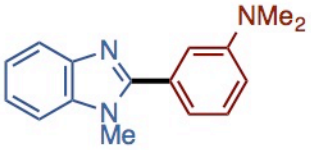

3Ag

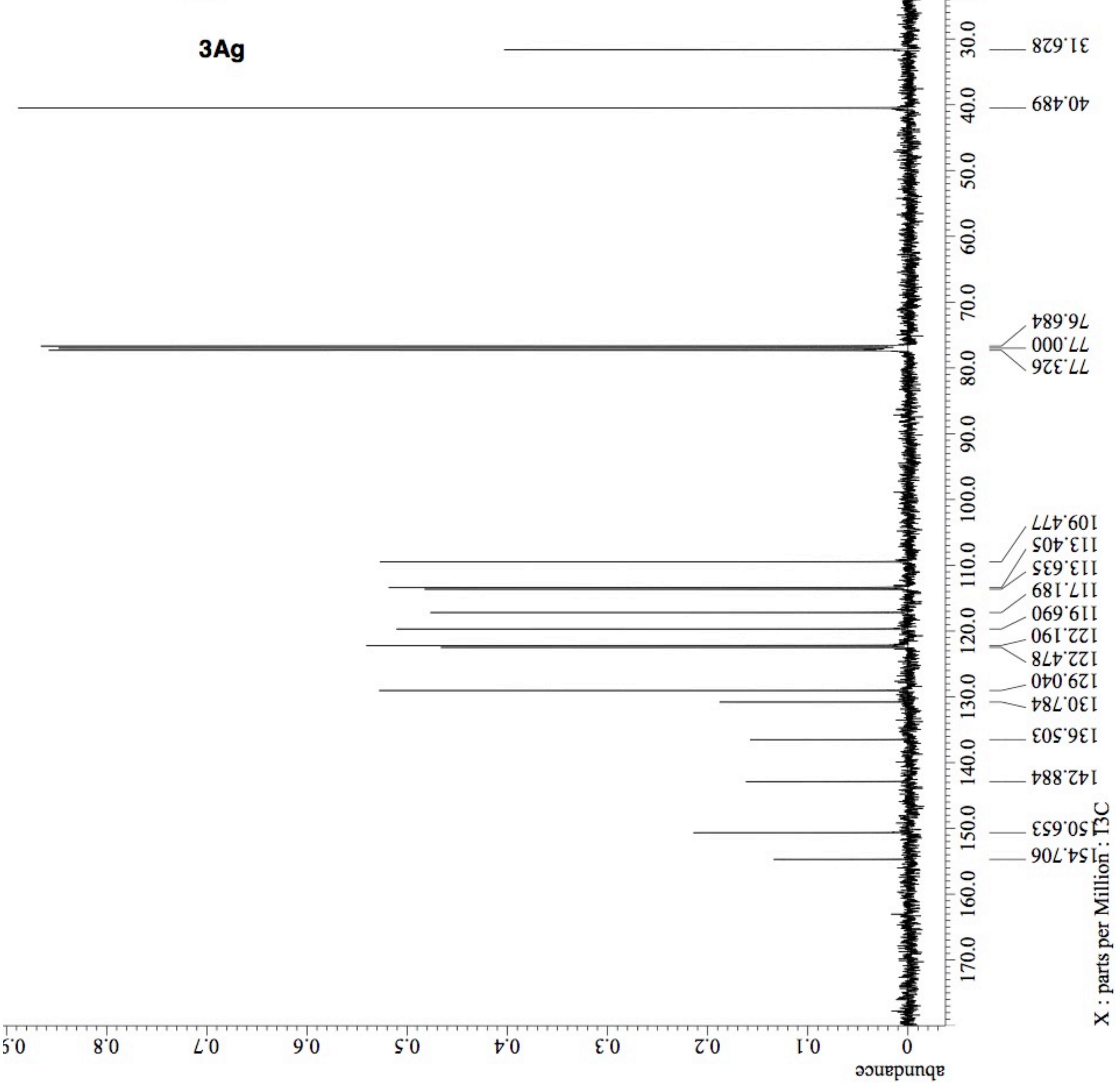

^{1}H NMR of **3Ah** (400 MHz, $CDCl_3$)

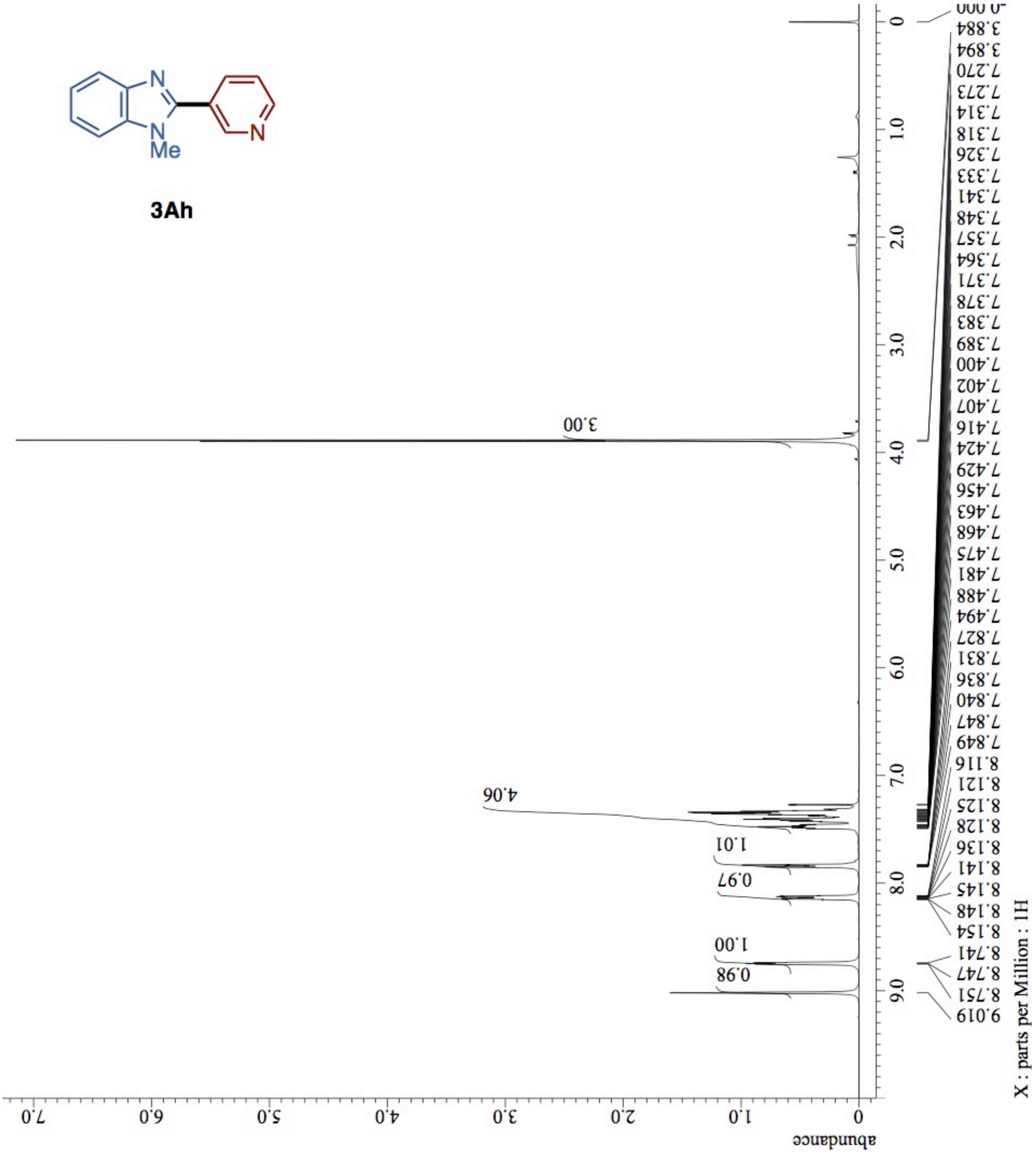

^{13}C NMR of **3Ah** (100 MHz, $CDCl_3$)

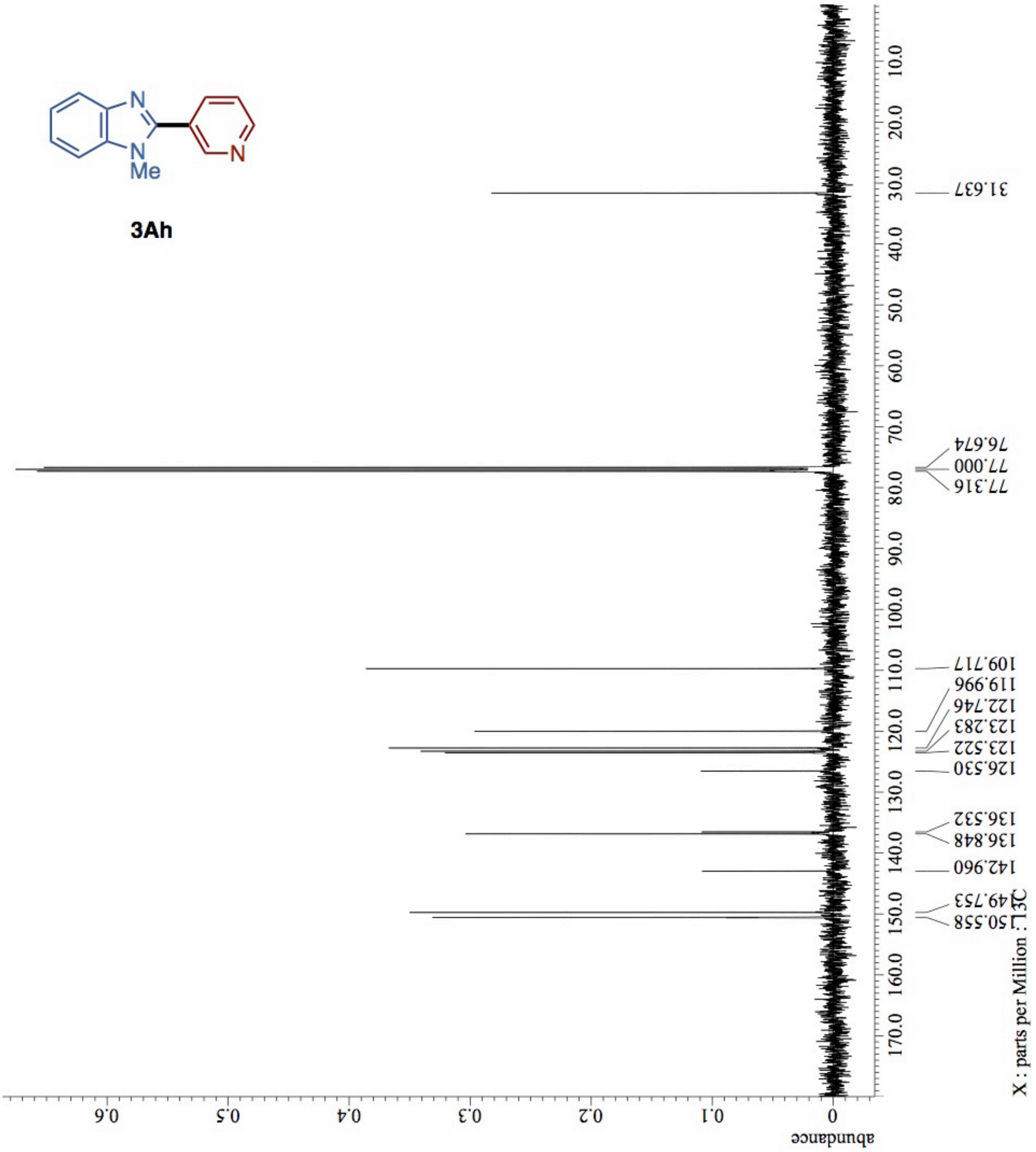

3Ah

^{1}H NMR of **3Ai** (400 MHz, $CDCl_3$)

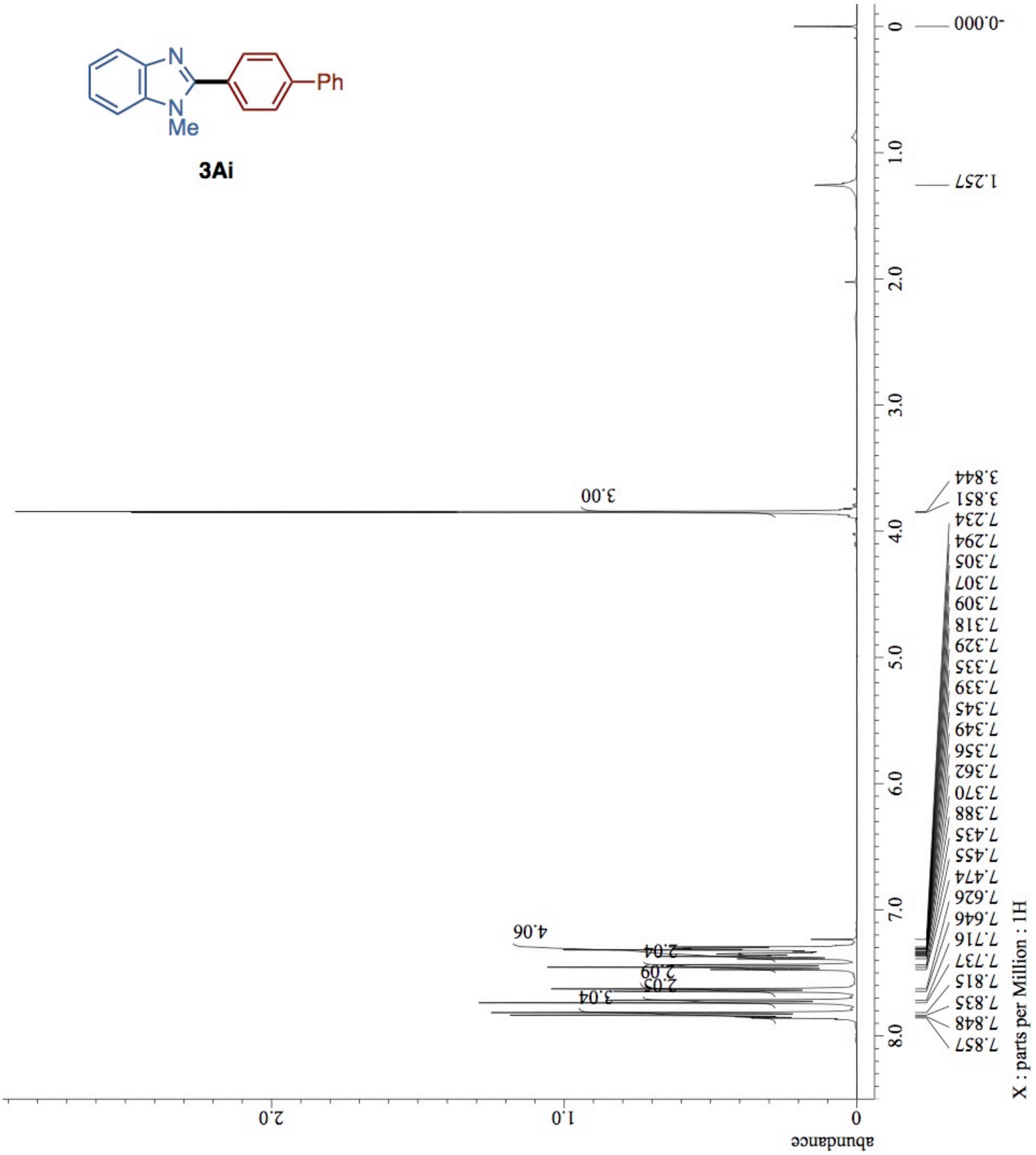

^{13}C NMR of **3Ai** (100 MHz, $CDCl_3$)

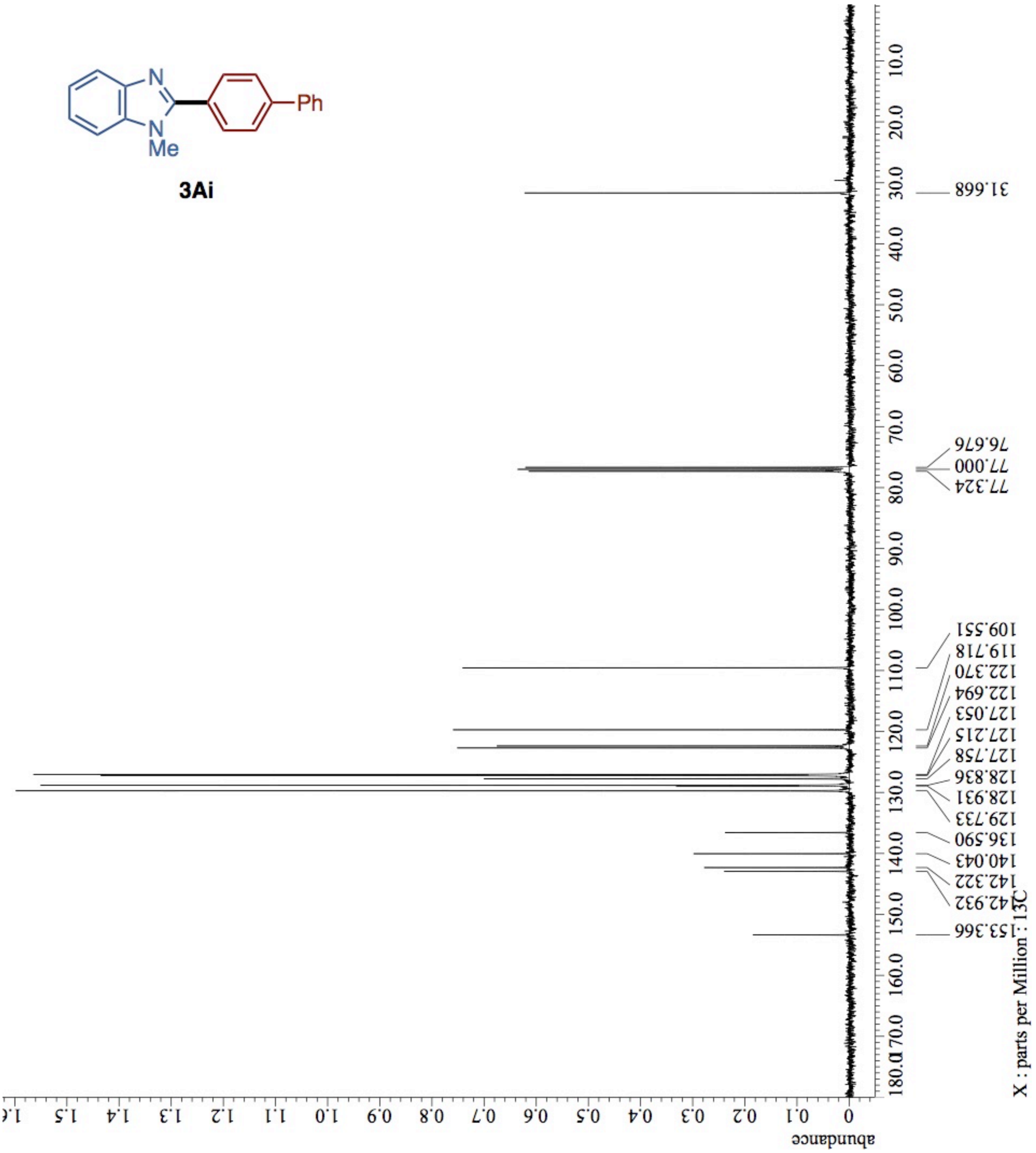

^{1}H NMR of **3Ab** (400 MHz, $CDCl_3$)

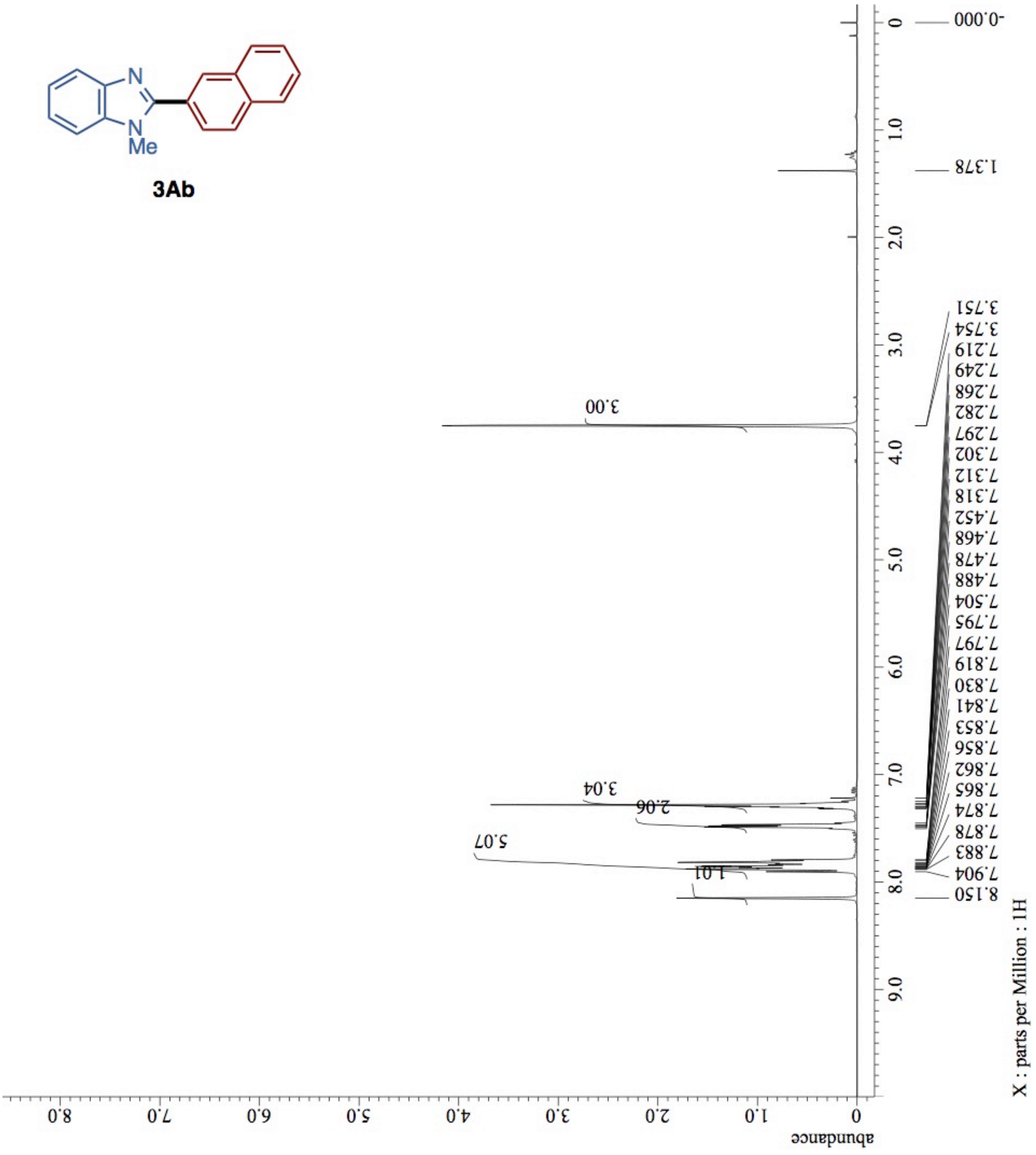

3Ab

^{13}C NMR of **3Ab** (100 MHz, $CDCl_3$)

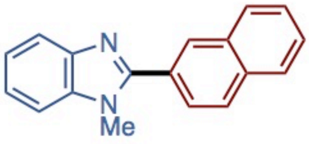

3Ab

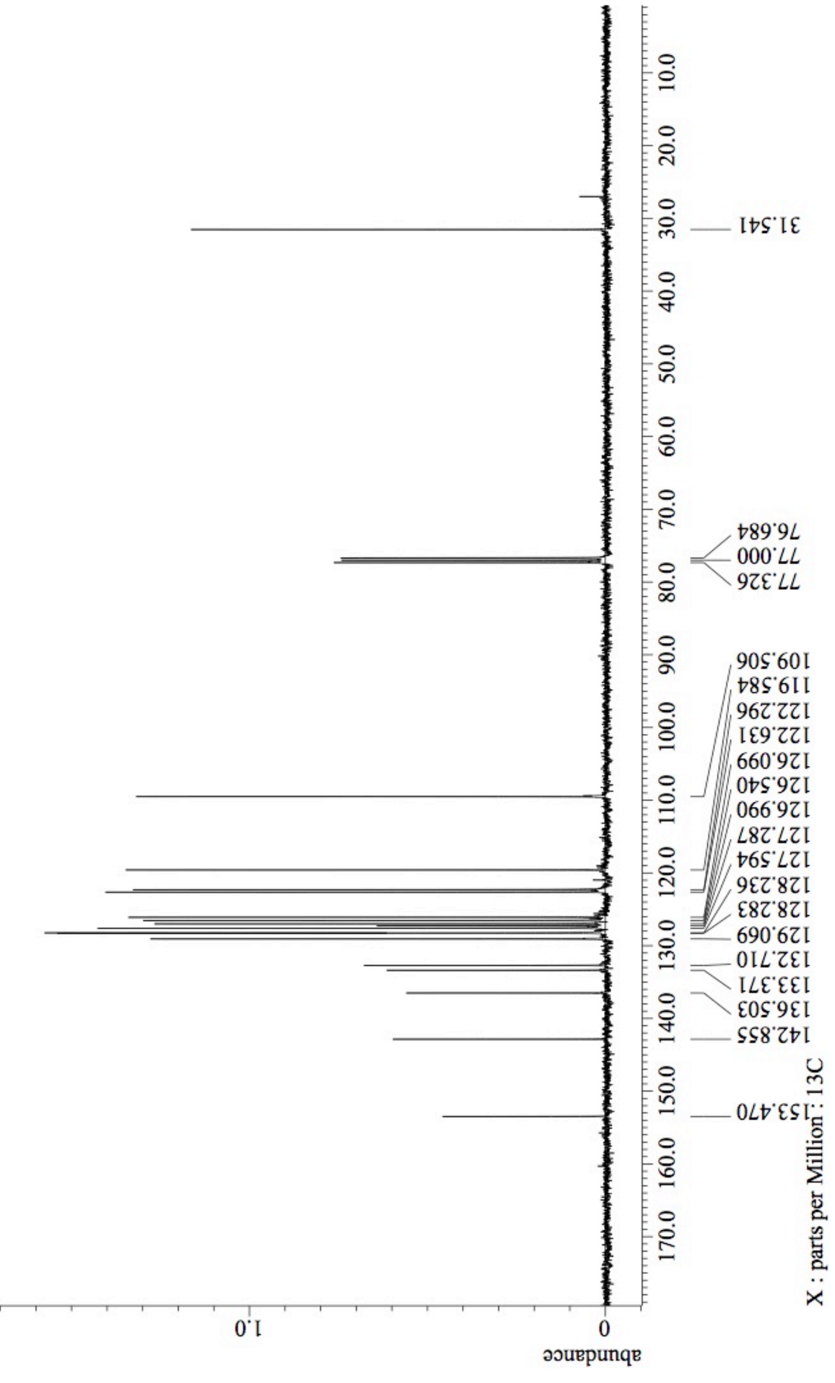

^{1}H NMR of **3Aj** (400 MHz, $CDCl_3$)

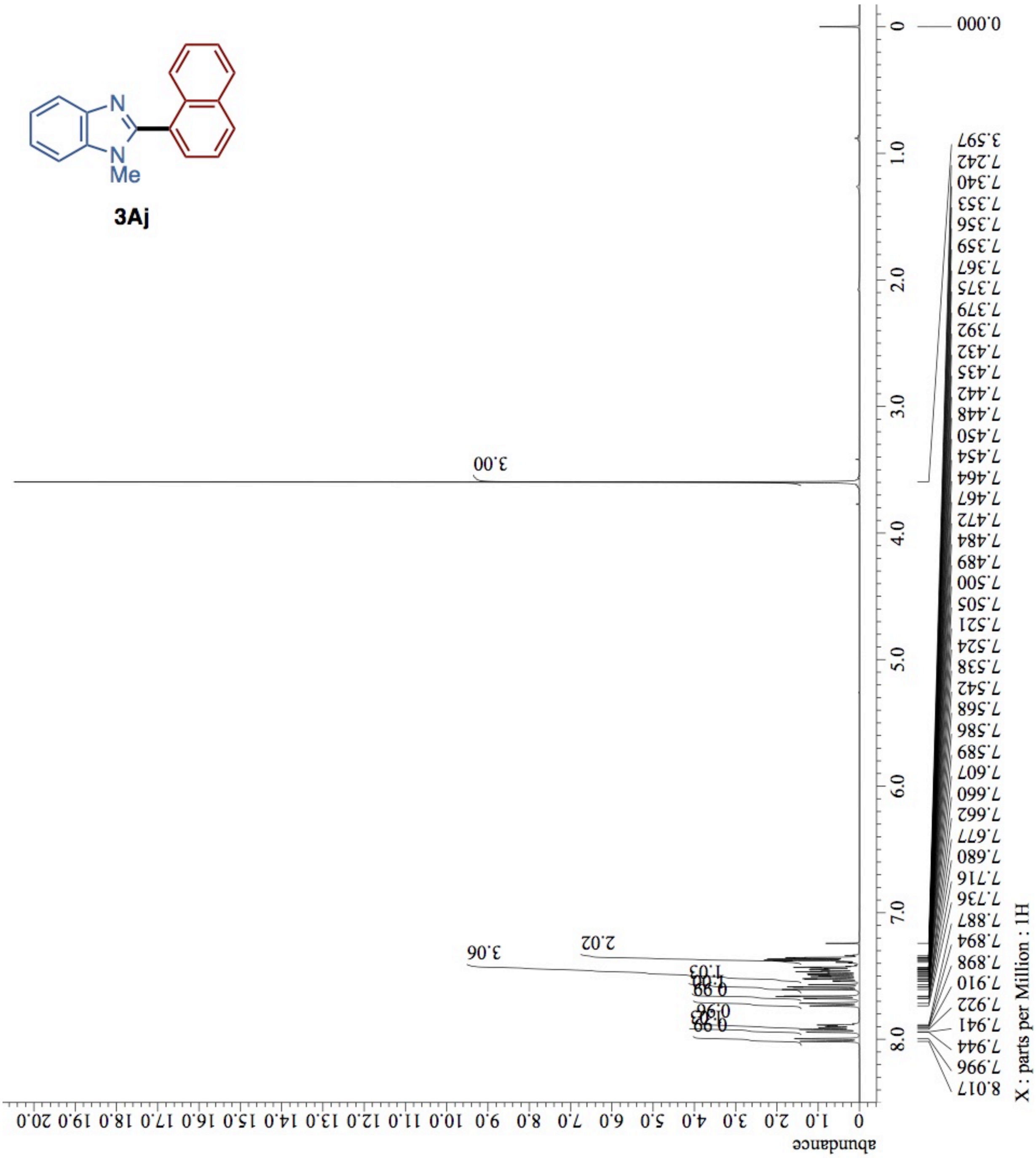

^{13}C NMR of **3Aj** (100 MHz, $CDCl_3$)

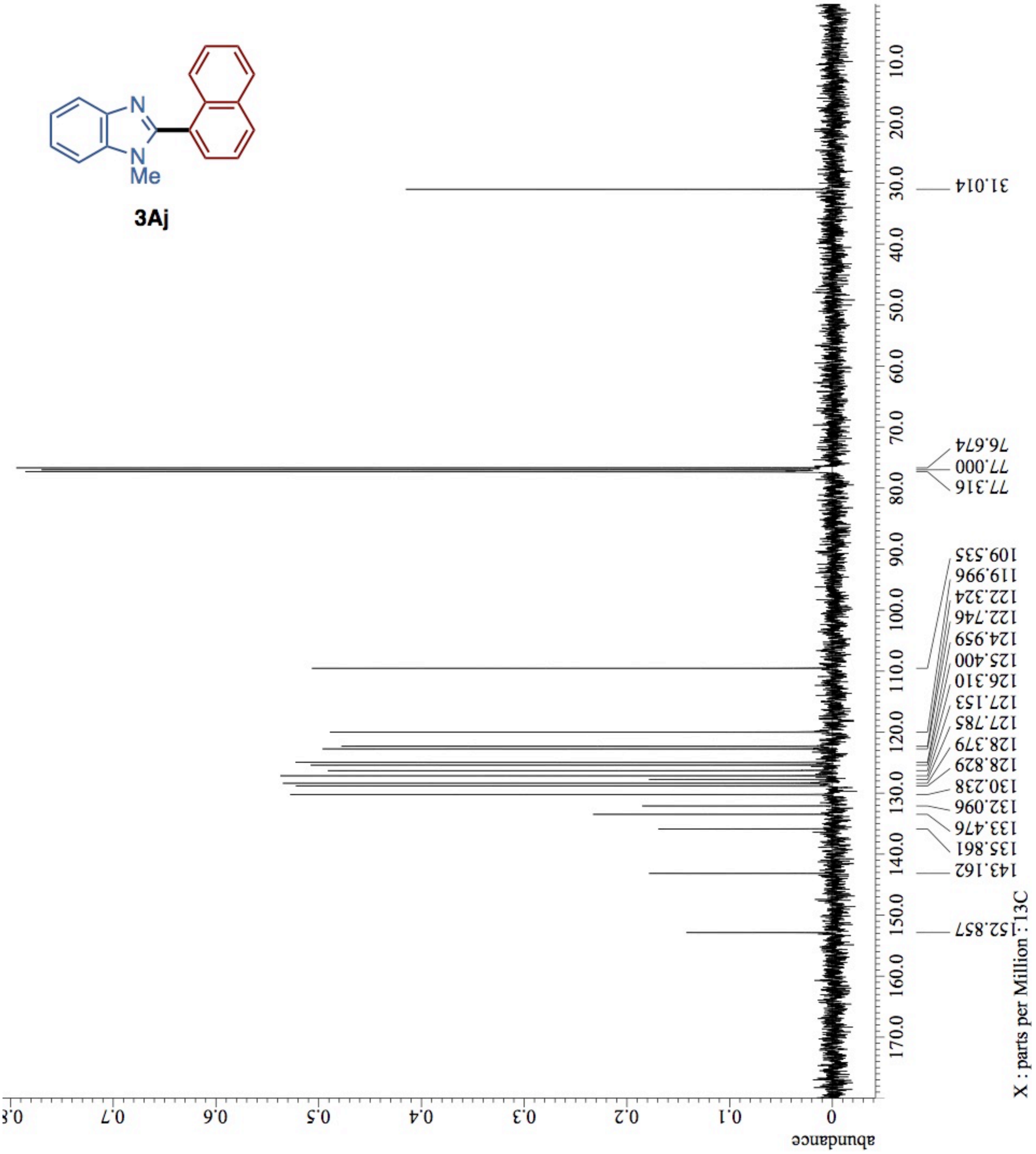

3Aj

^{1}H NMR of **3Qb** (400 MHz, $CDCl_3$)

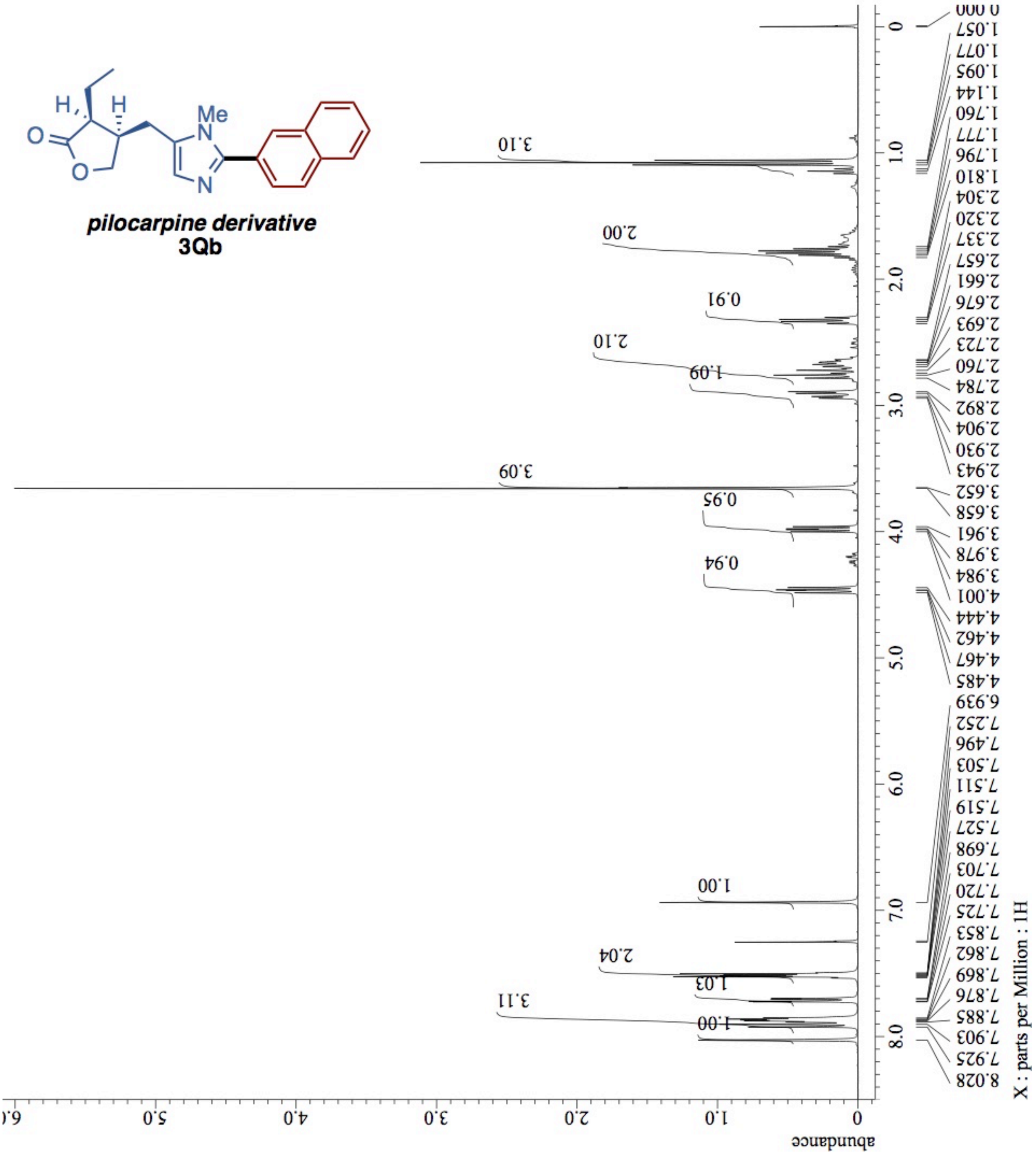

^{13}C NMR of **3Qb** (100 MHz, $CDCl_3$)

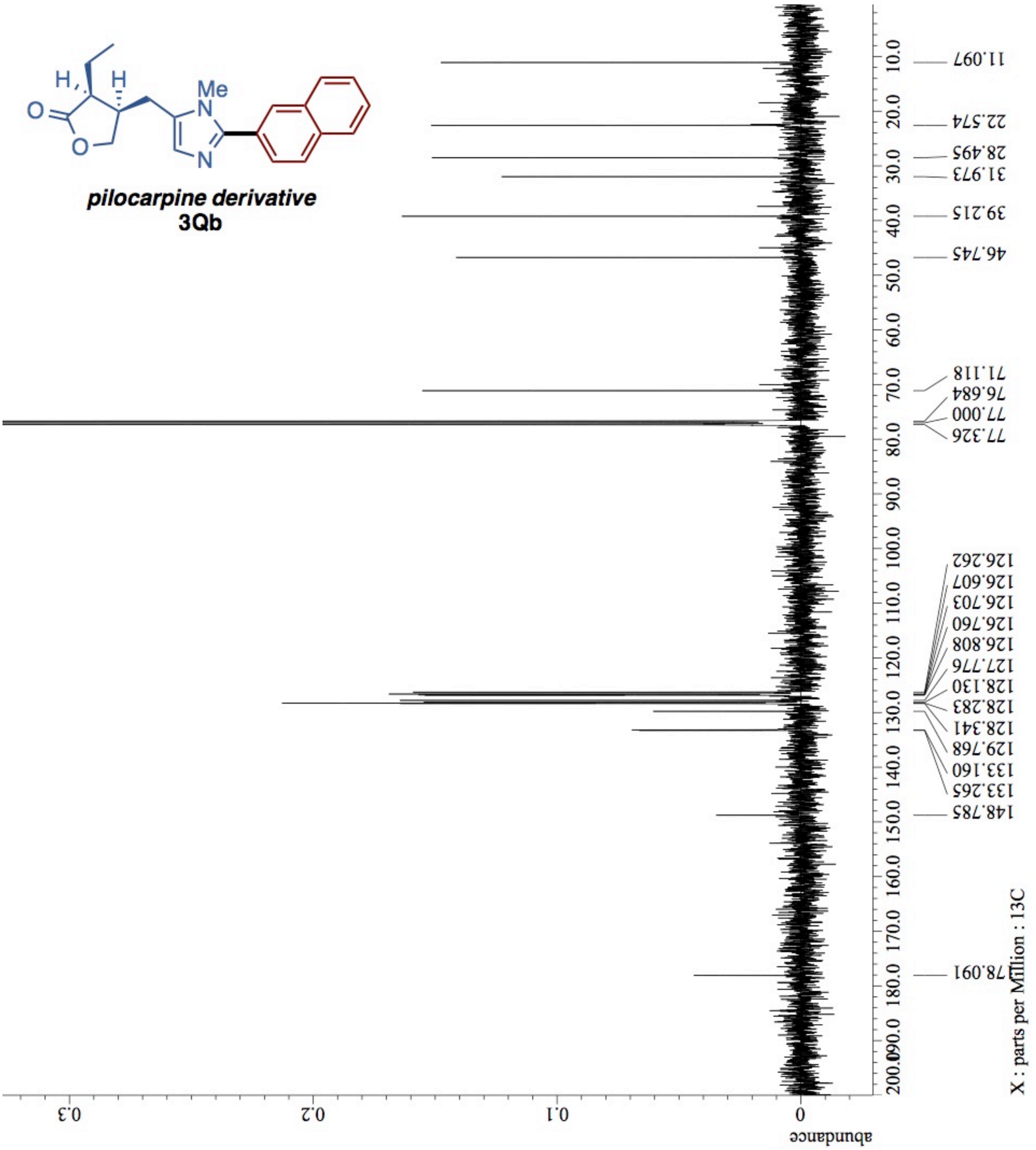

^{1}H NMR of **3As** (400 MHz, $CDCl_3$)

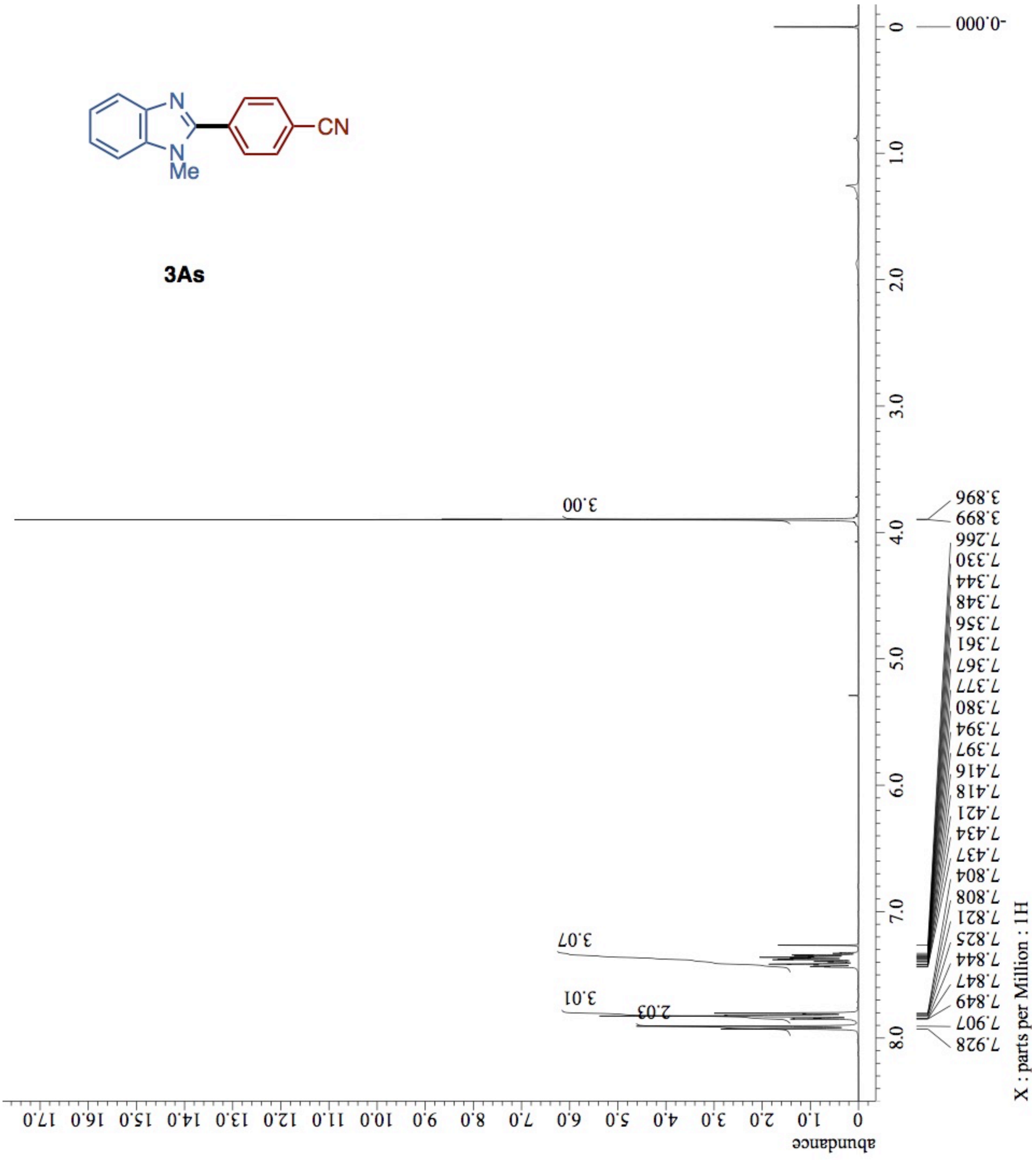

3As

^{13}C NMR of **3As** (100 MHz, $CDCl_3$)

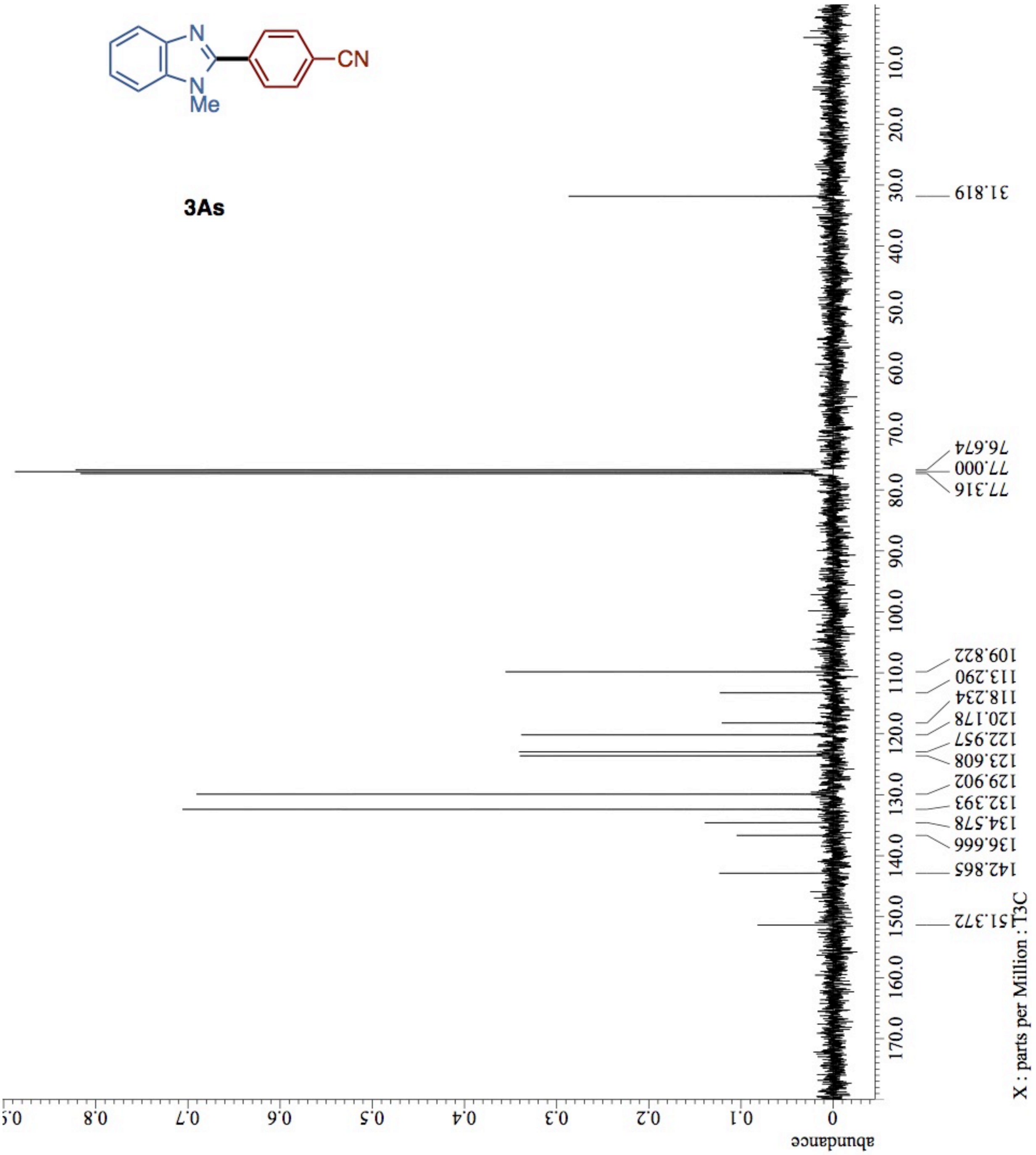

^{1}H NMR of **3At** (400 MHz, CDCl$_3$)

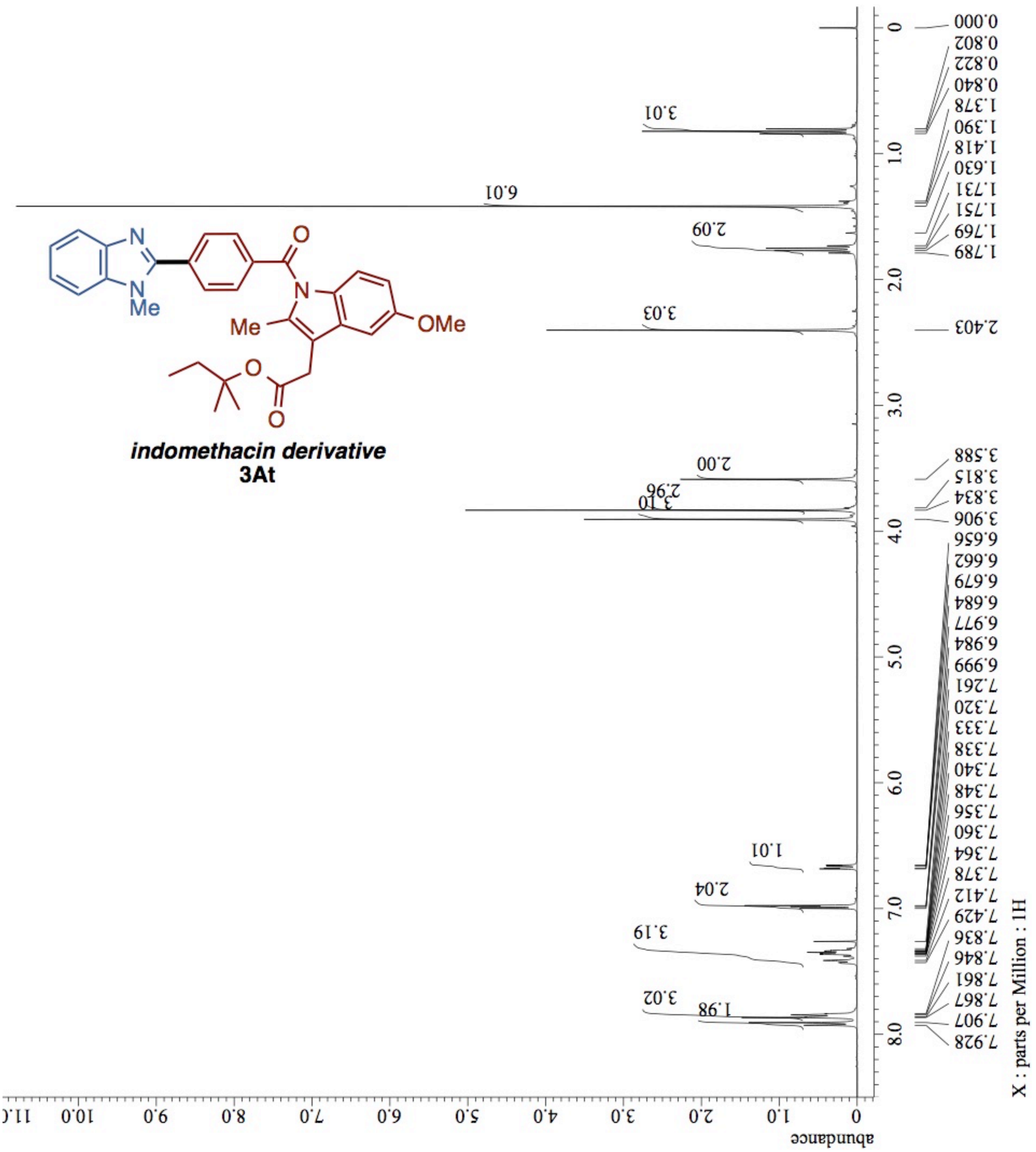

^{13}C NMR of **3At** (100 MHz, $CDCl_3$)

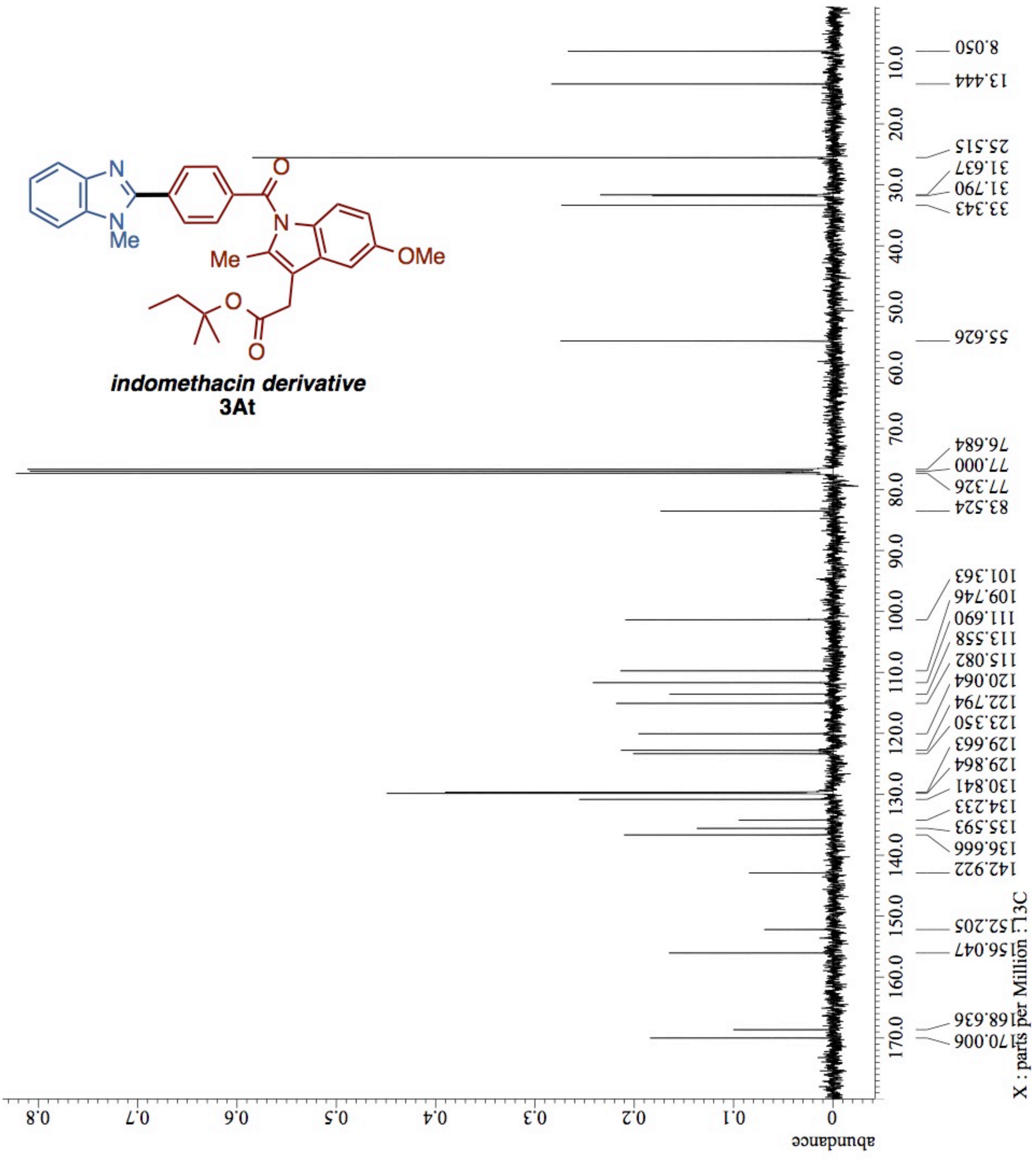

^{1}H NMR of **3Ak** (400 MHz, $CDCl_3$)

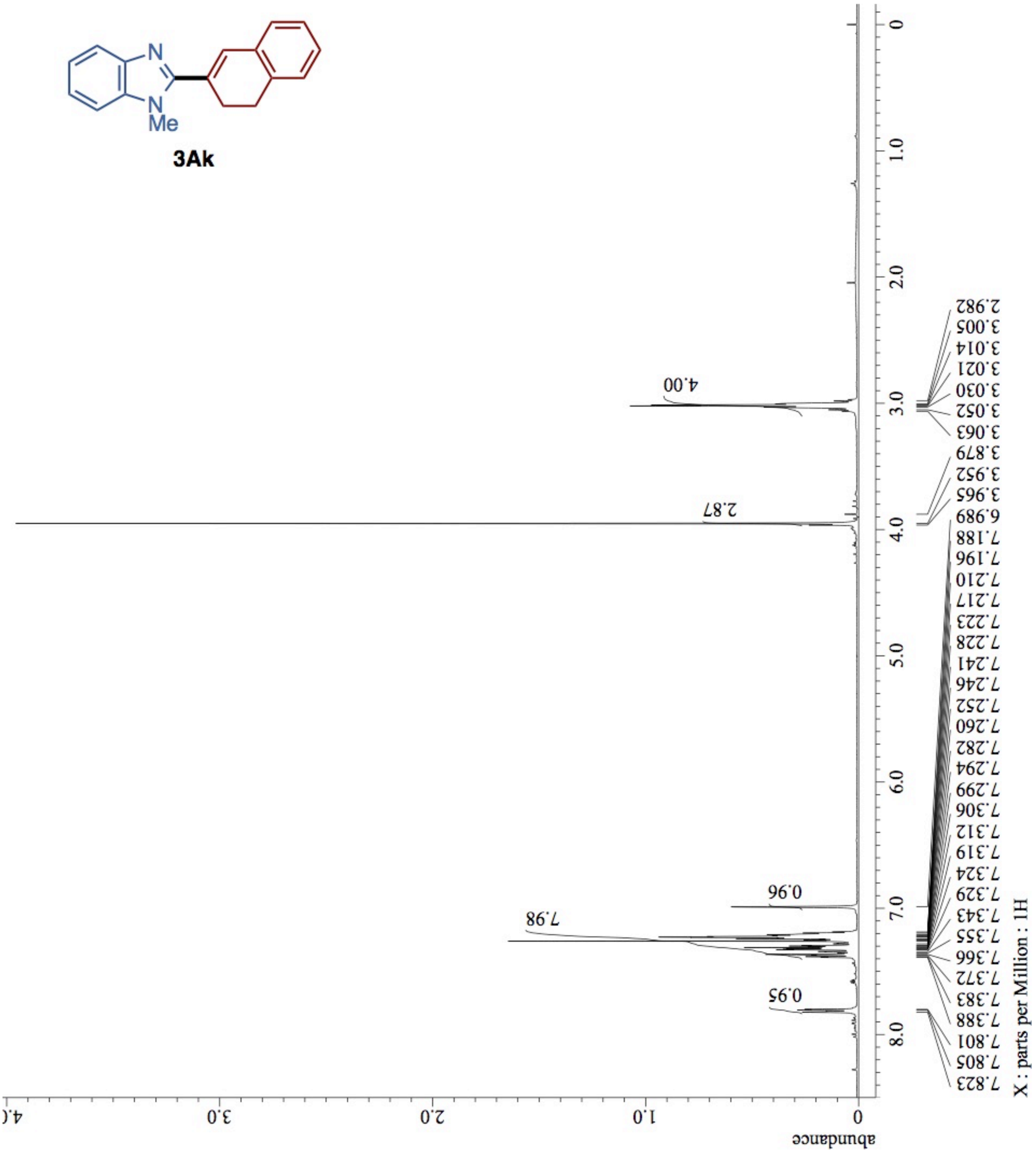

^{13}C NMR of **3Ak** (100 MHz, $CDCl_3$)

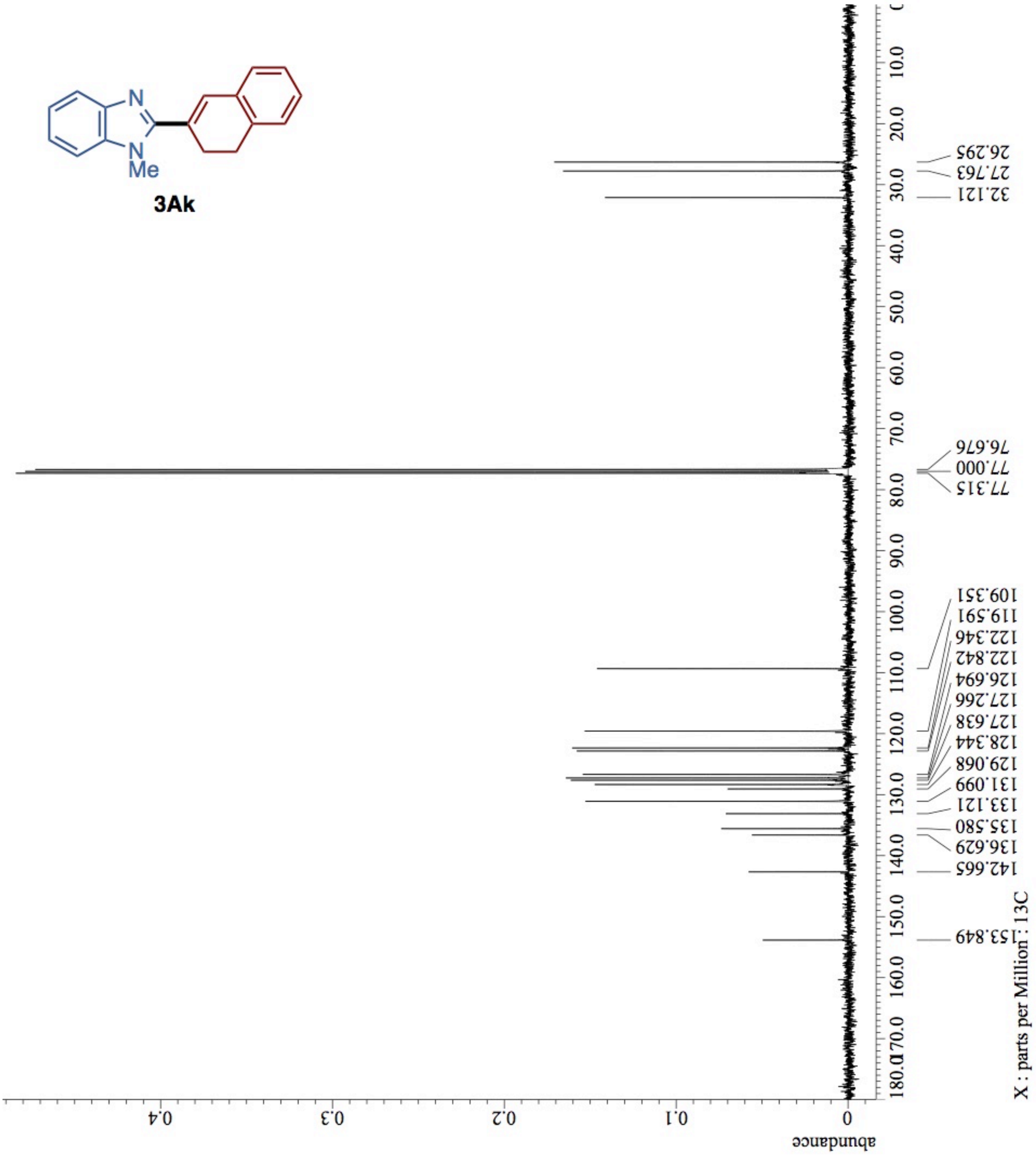

^{1}H NMR of **3AI** (400 MHz, $CDCl_3$)

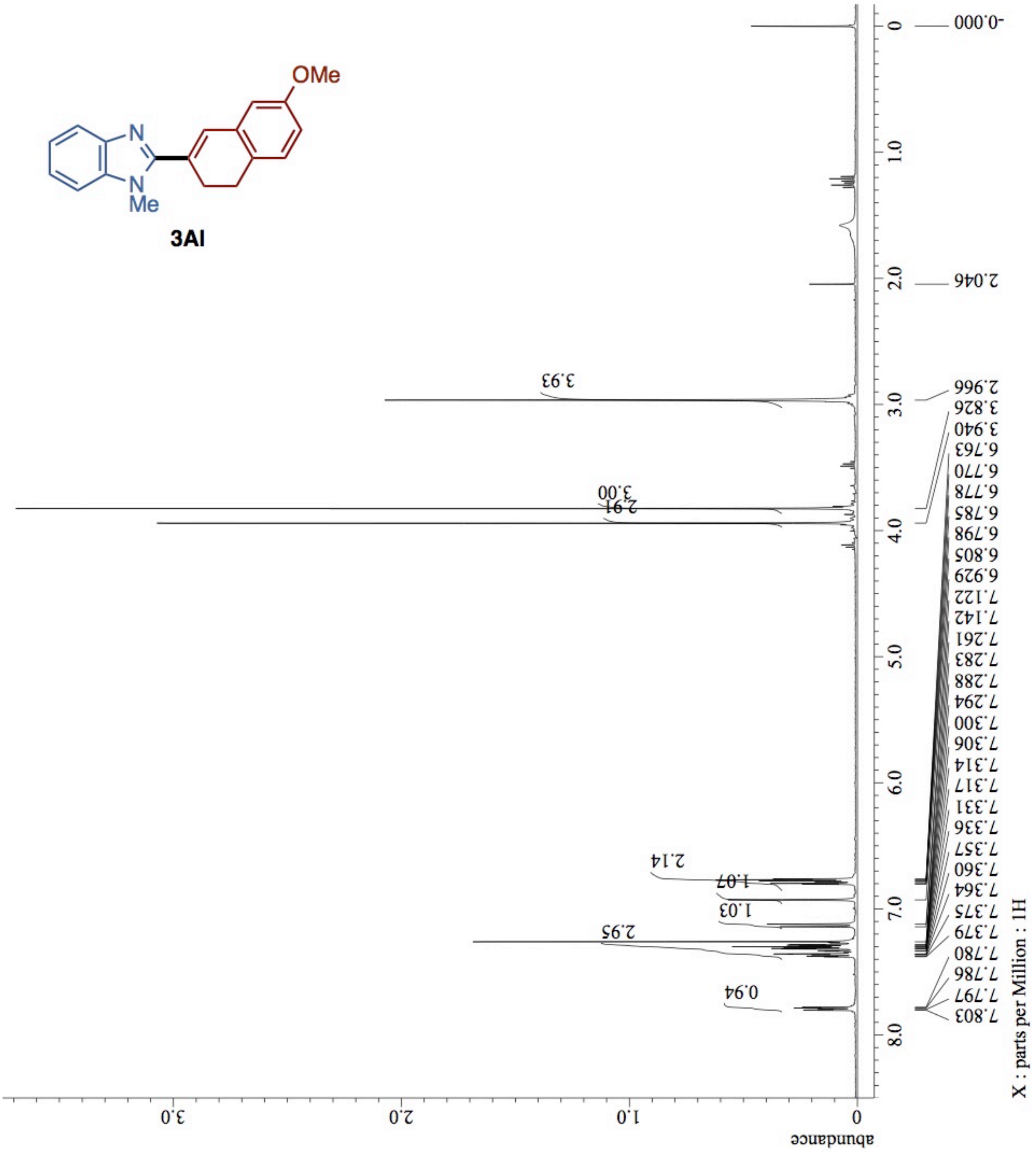

3AI

^{13}C NMR of **3AI** (100 MHz, $CDCl_3$)

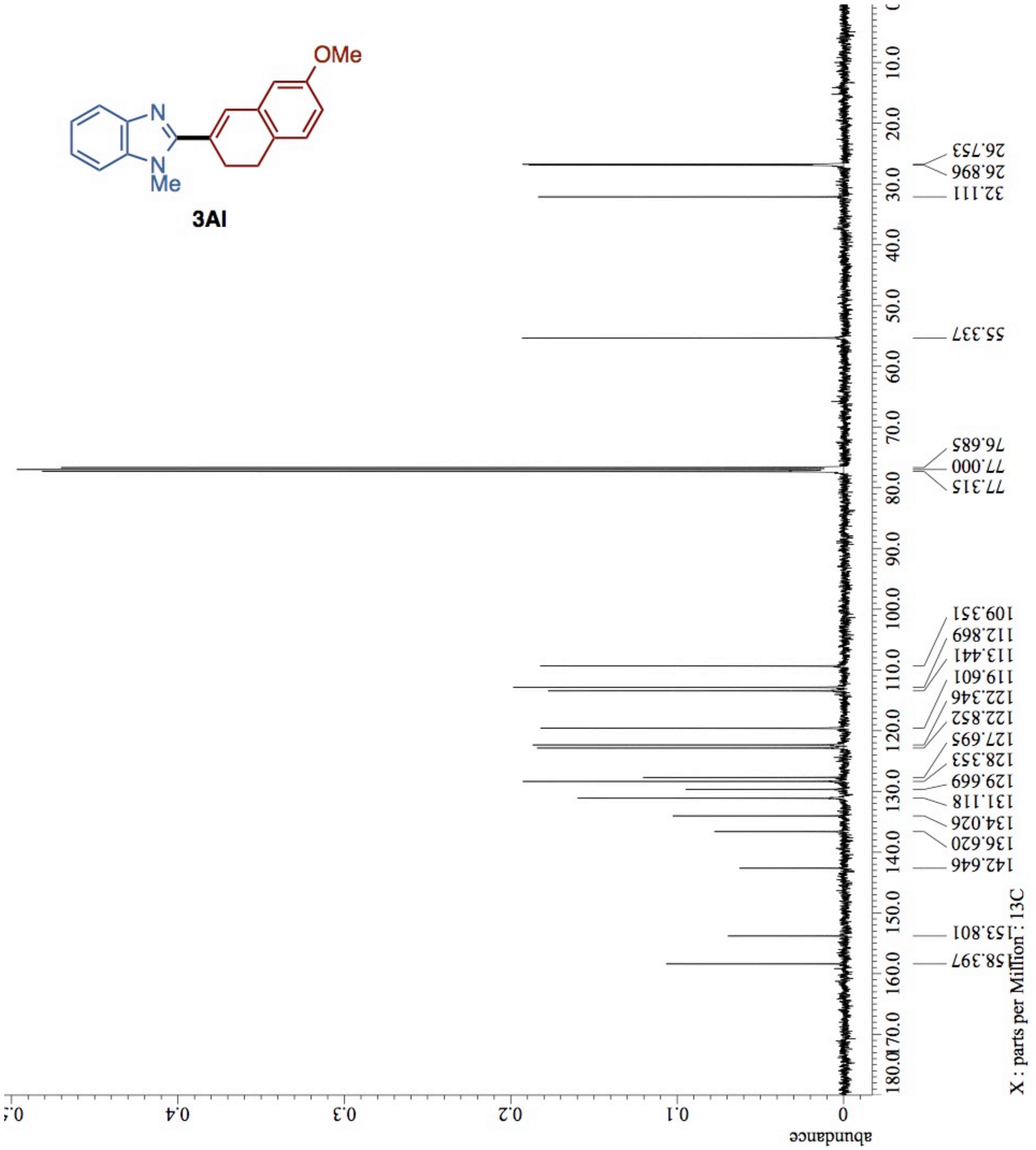

^{1}H NMR of **3Am** (400 MHz, $CDCl_3$)

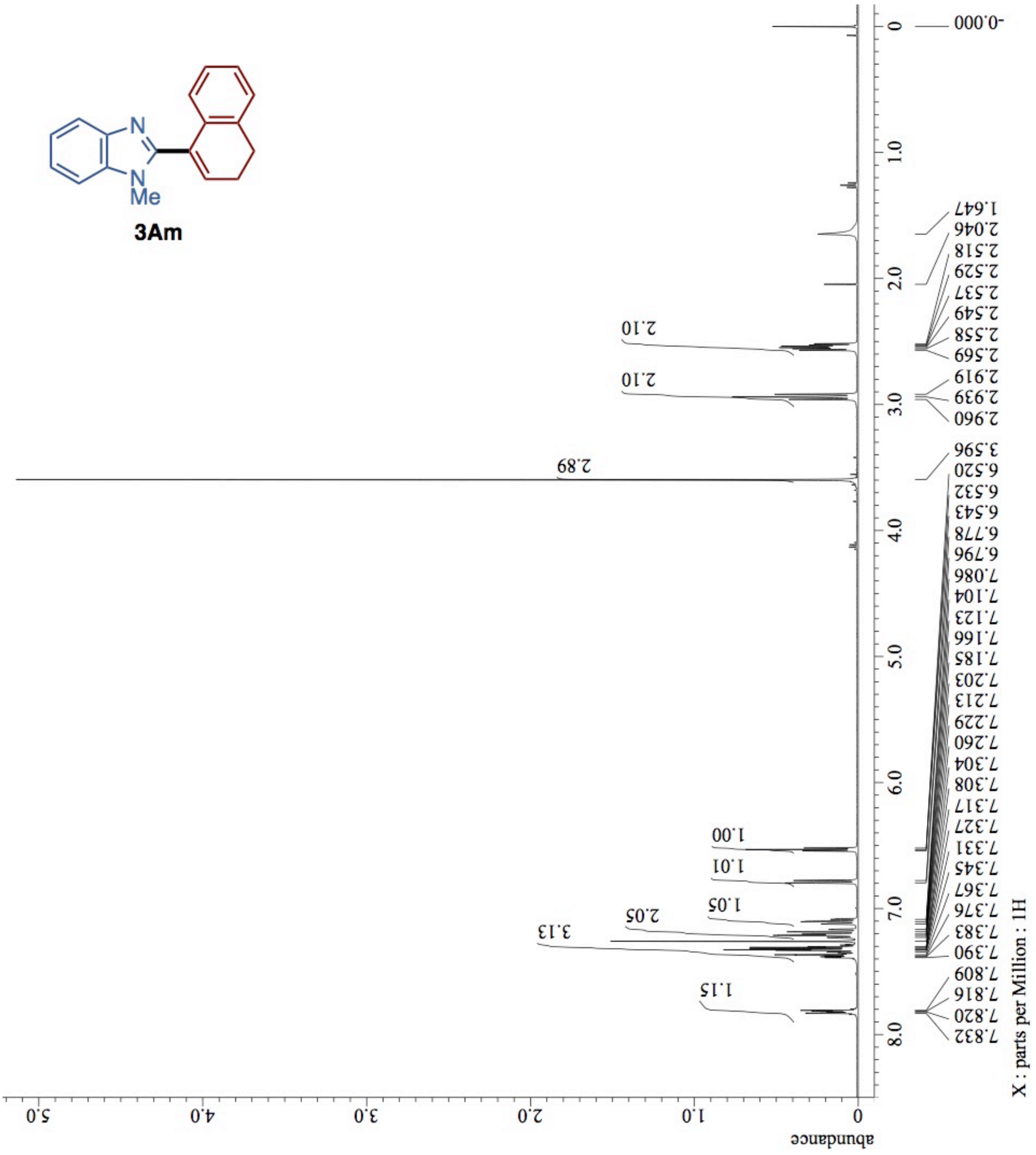

^{13}C NMR of **3Am** (100 MHz, $CDCl_3$)

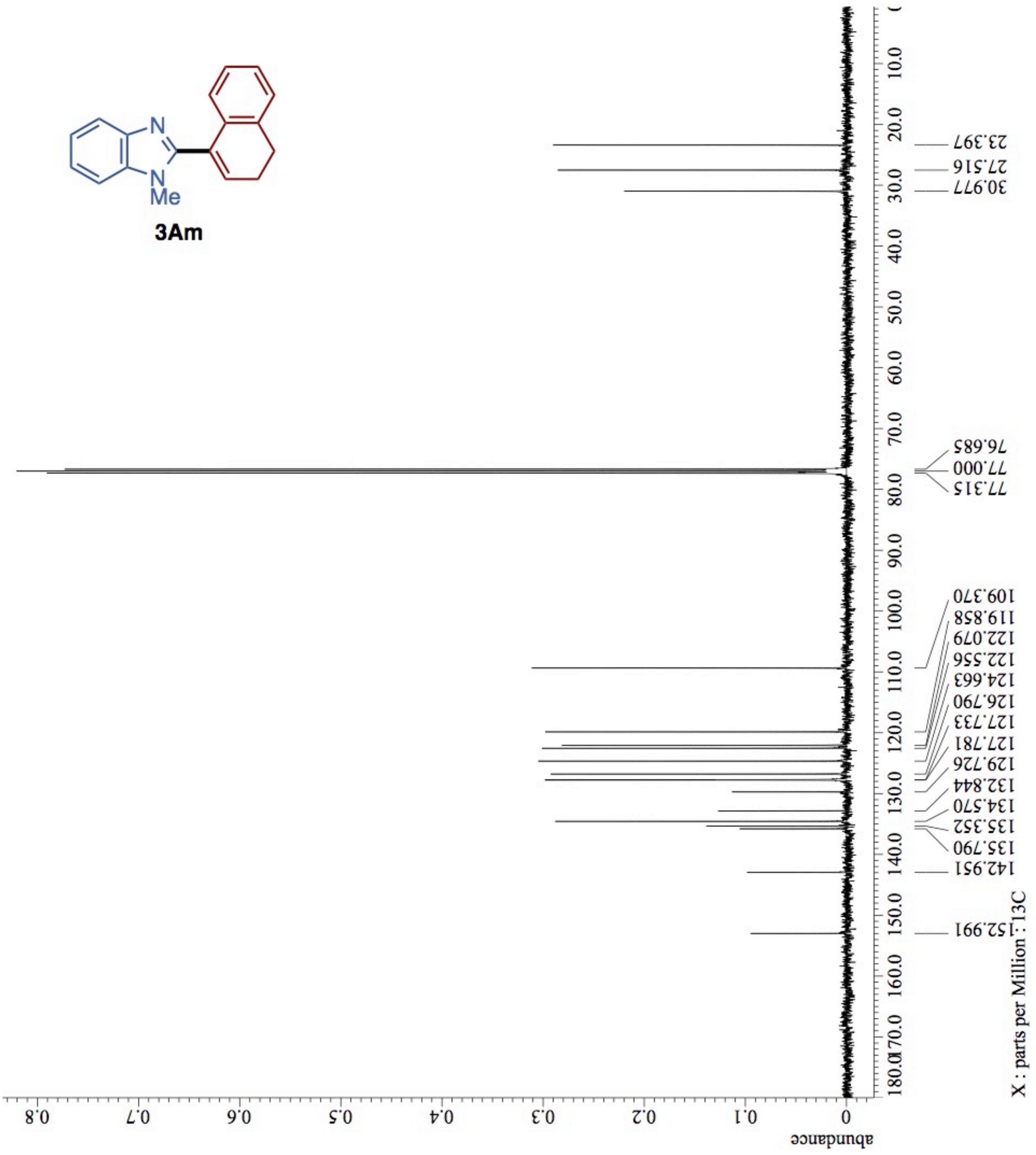

^{1}H NMR of **3An** (400 MHz, $CDCl_3$)

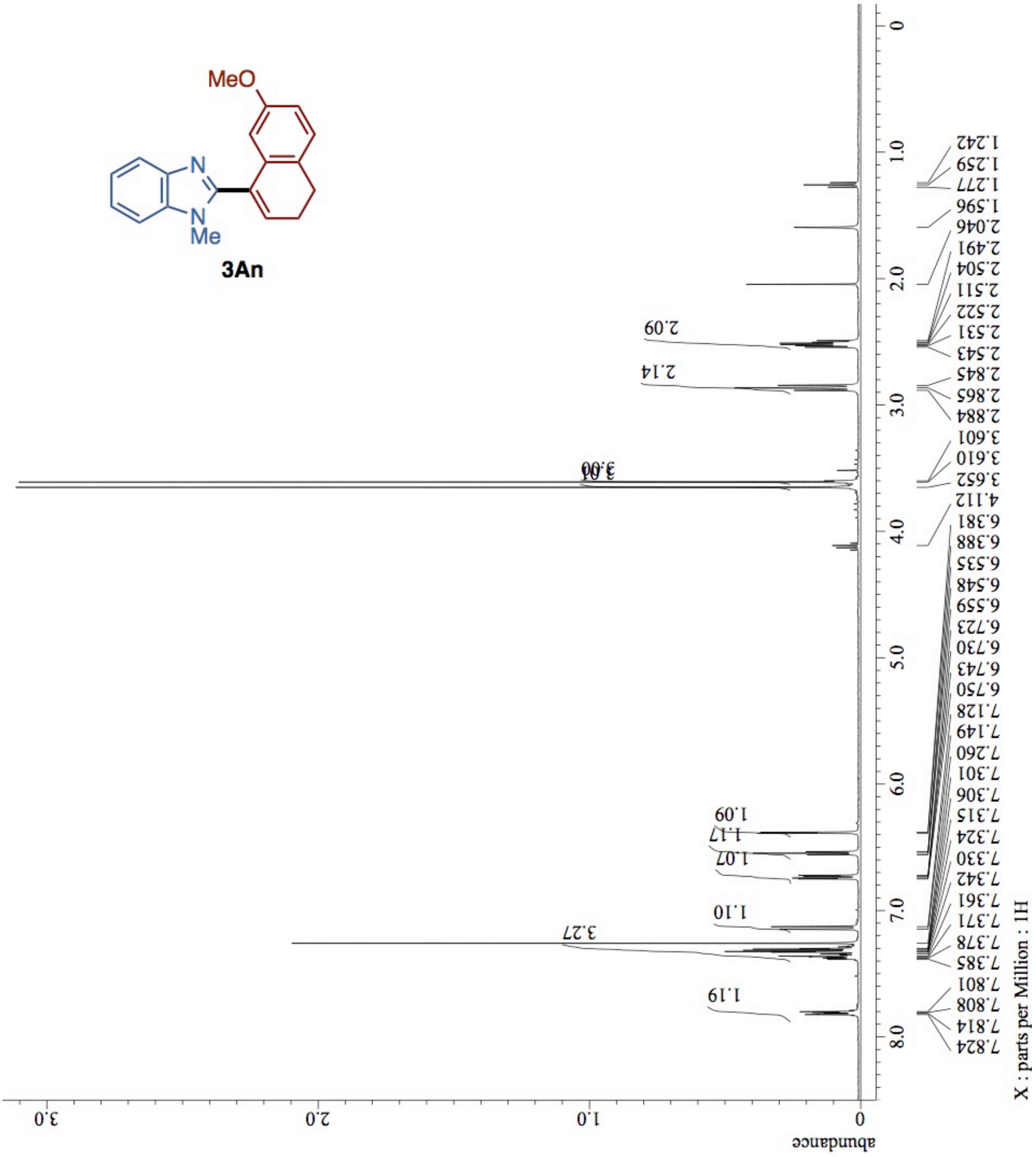

^{13}C NMR of **3An** (100 MHz, $CDCl_3$)

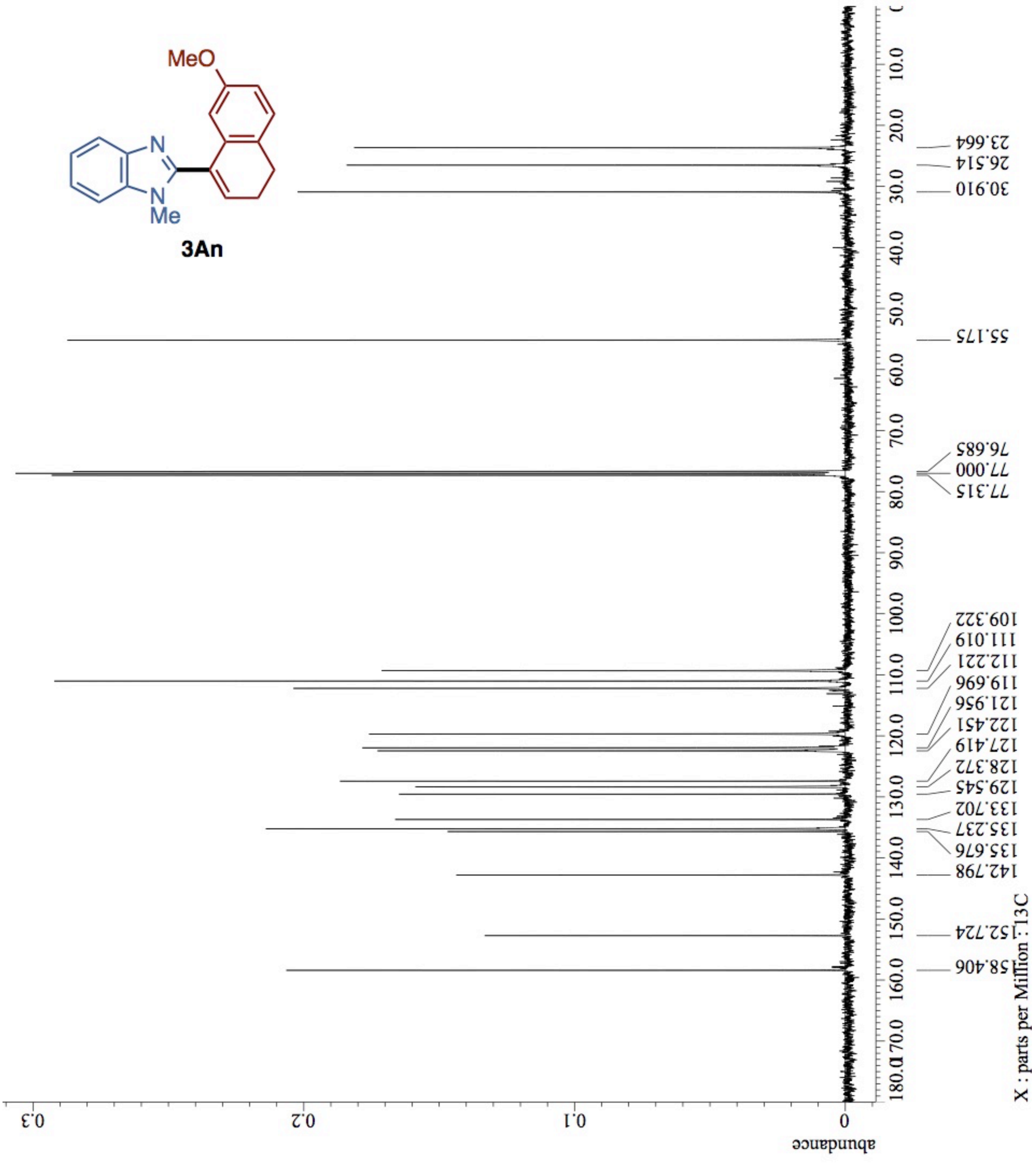

^{1}H NMR of **3Ao** (400 MHz, $CDCl_3$)

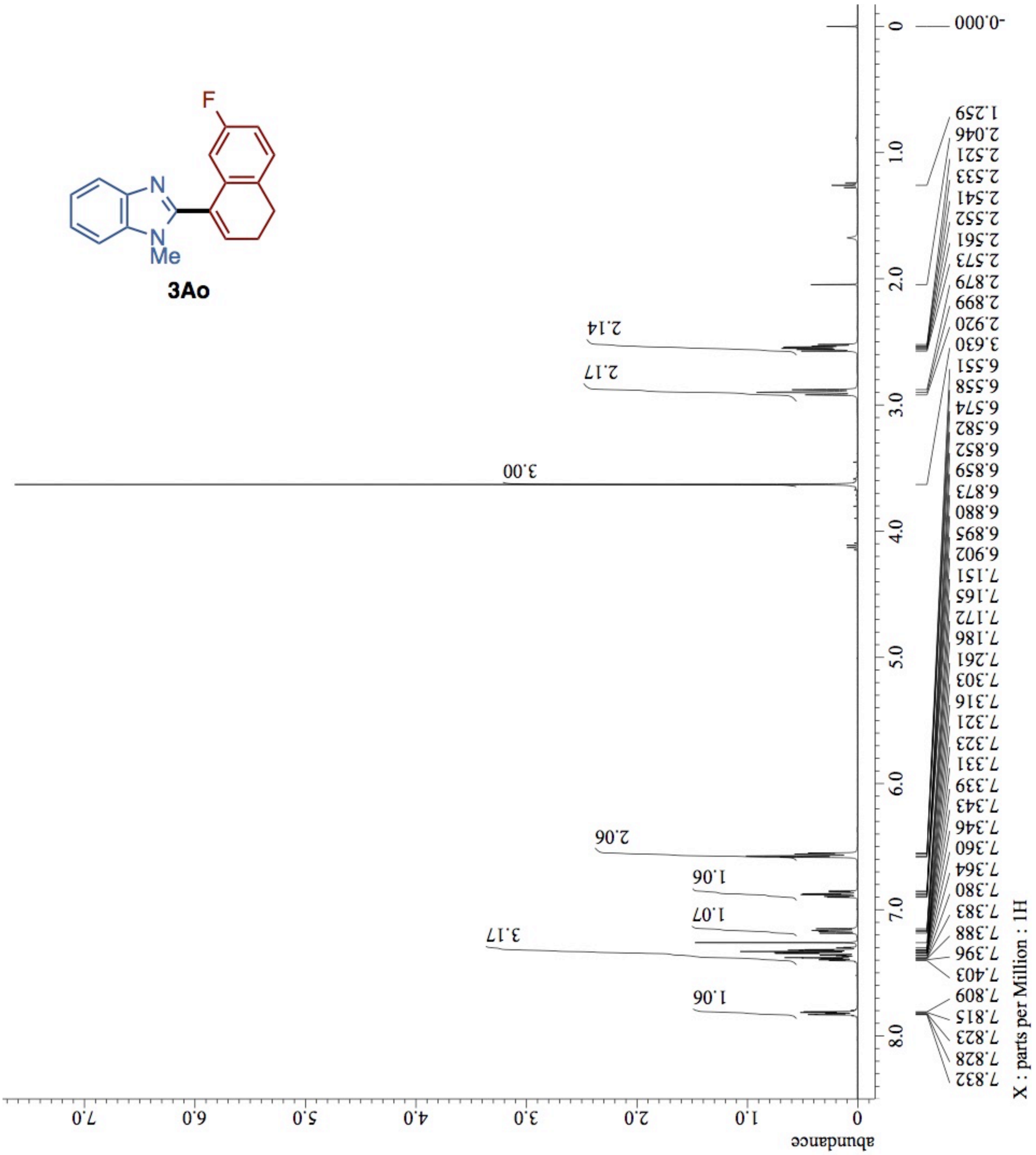

^{13}C NMR of **3Ao** (100 MHz, $CDCl_3$)

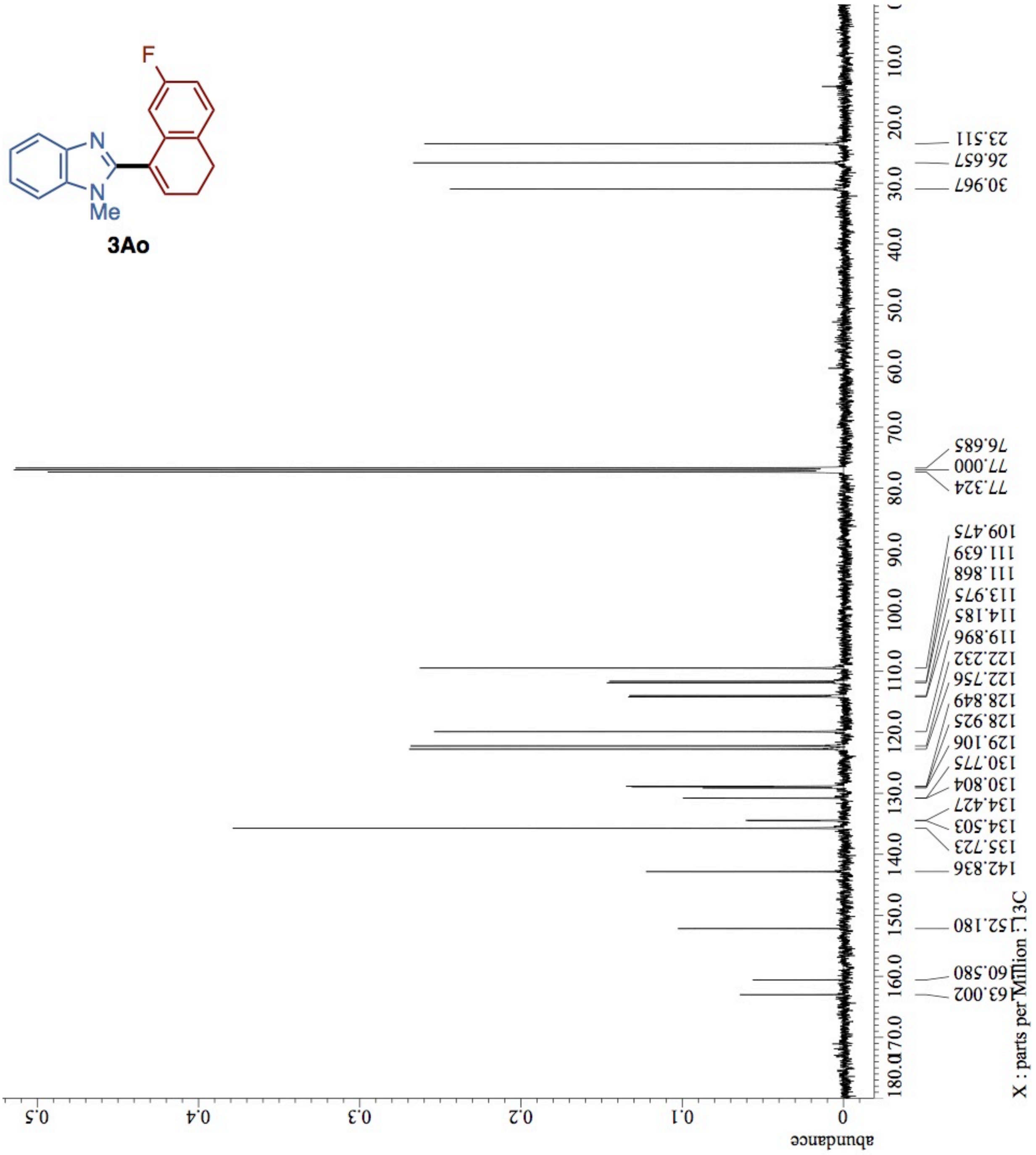

^{1}H NMR of **3Ap** (400 MHz, $CDCl_3$)

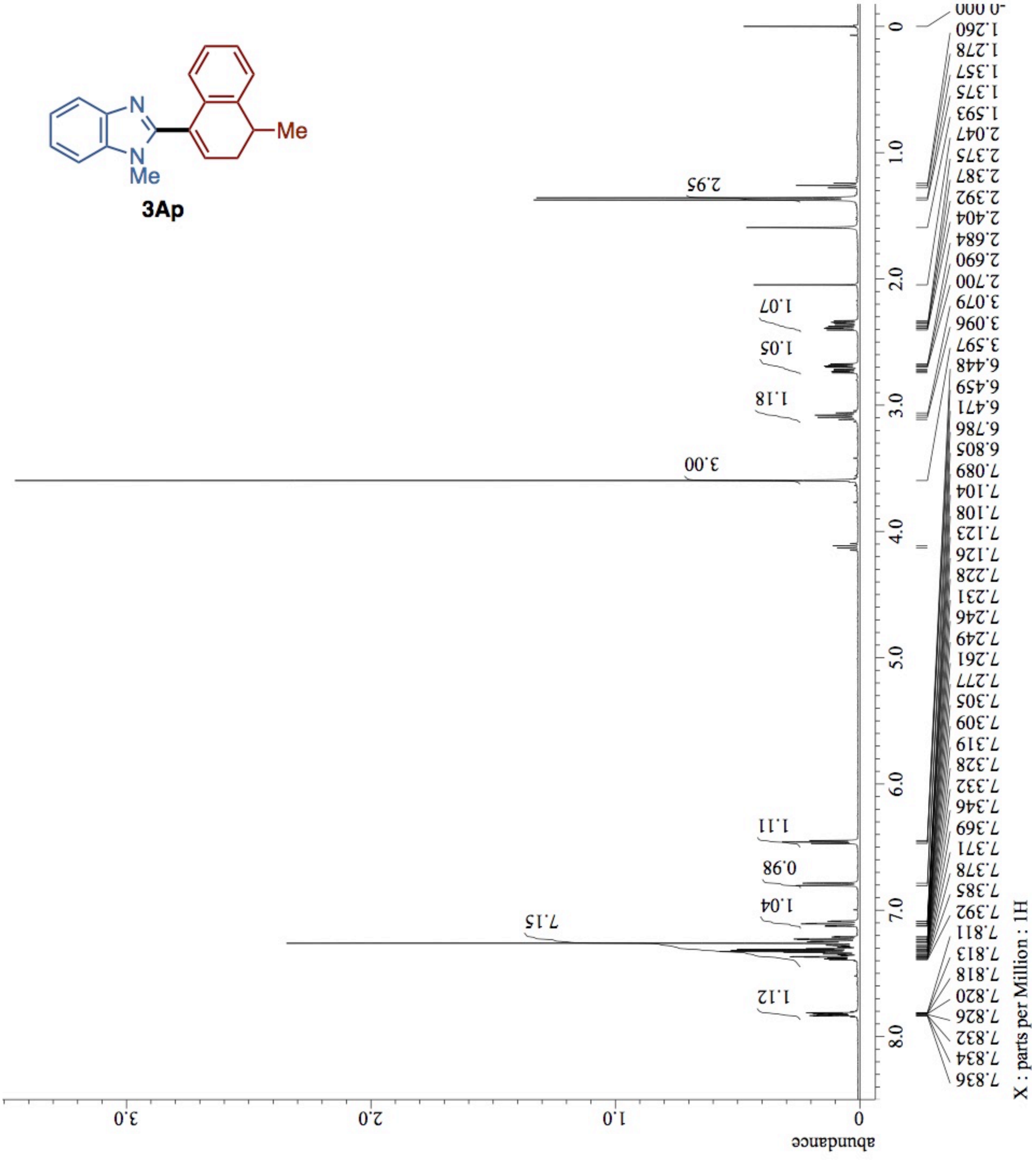

^{13}C NMR of **3Ap** (100 MHz, $CDCl_3$)

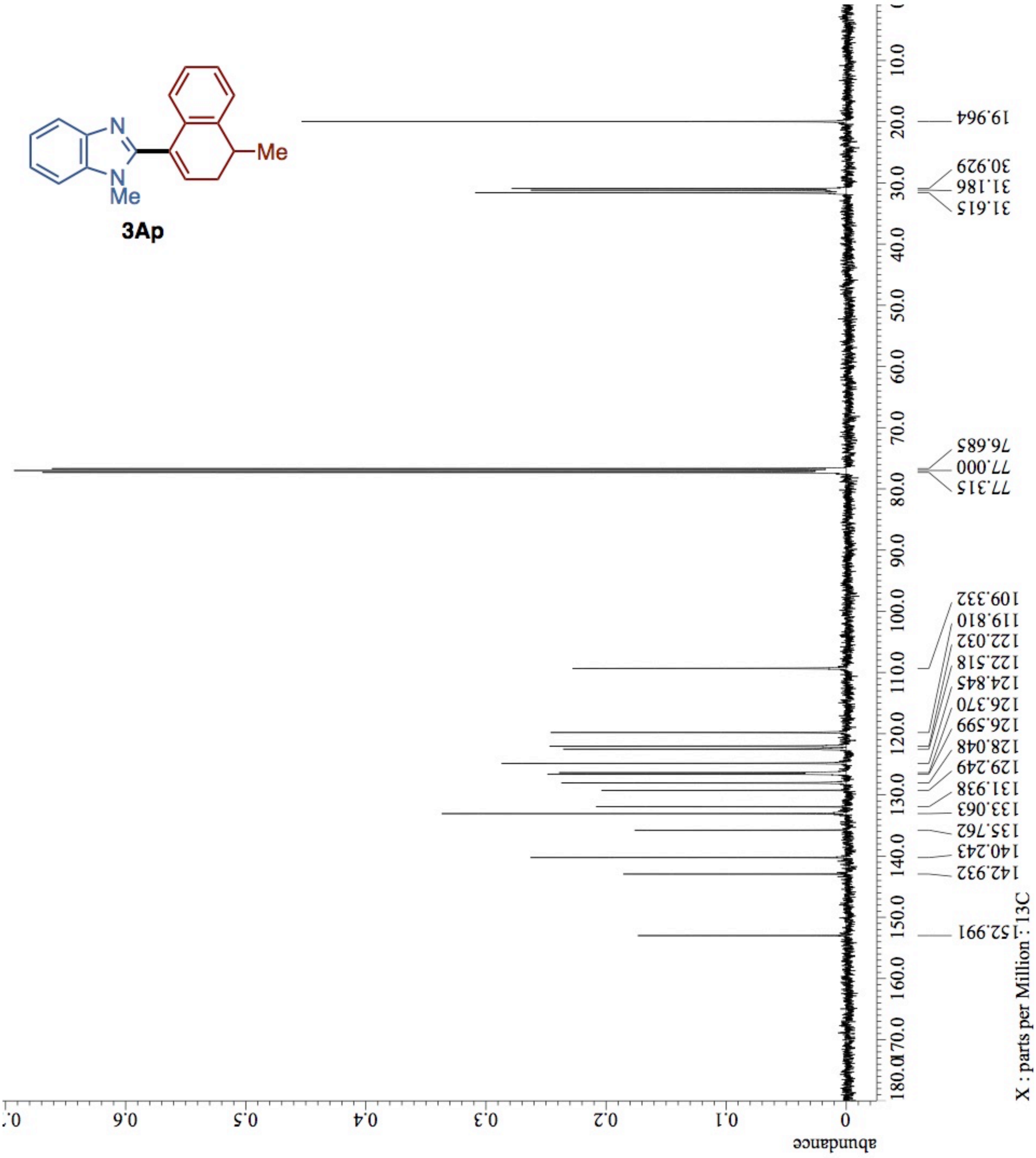

^{1}H NMR of **3Bk** (400 MHz, $CDCl_3$)

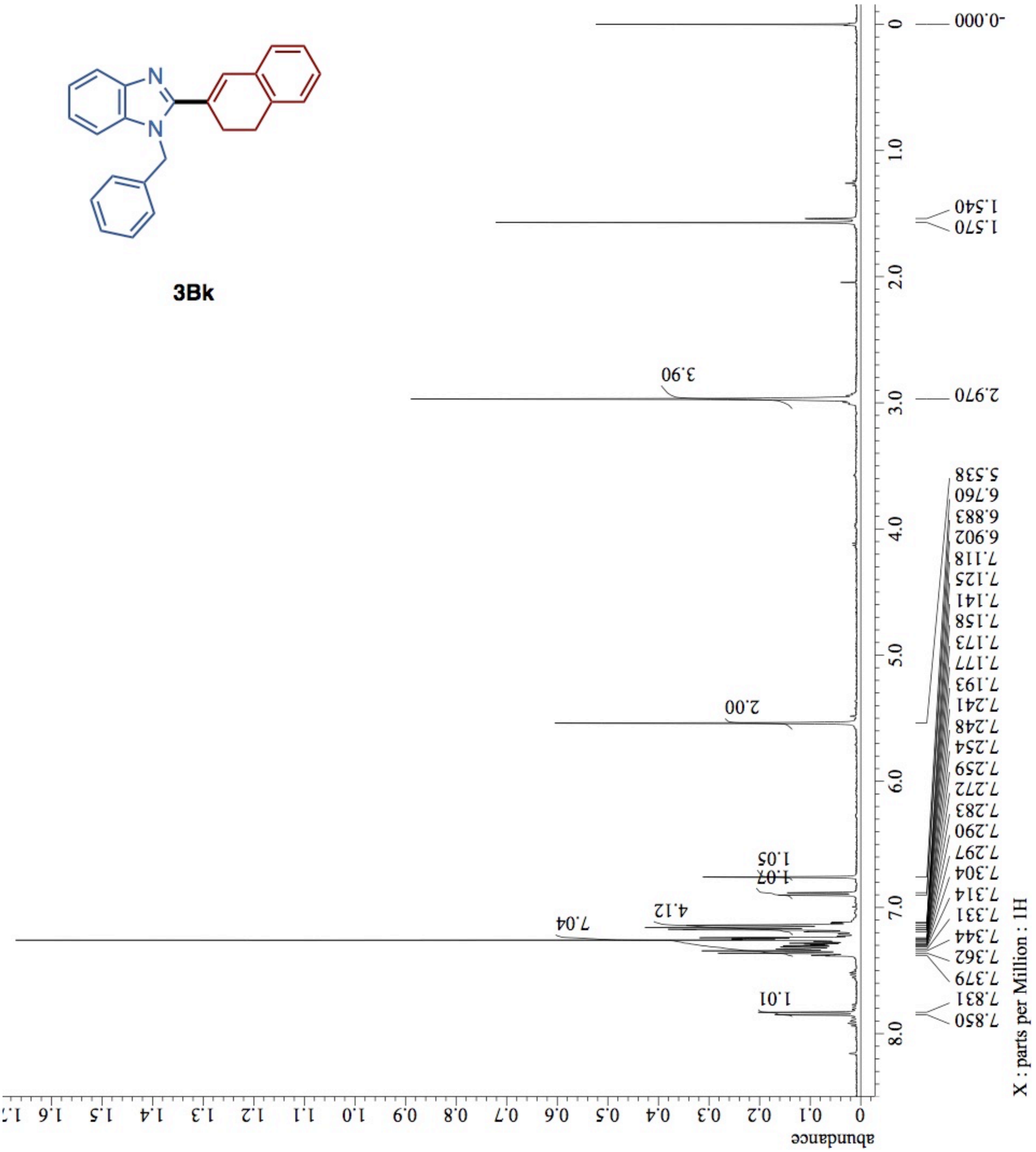

3Bk

^{13}C NMR of **3Bk** (100 MHz, $CDCl_3$)

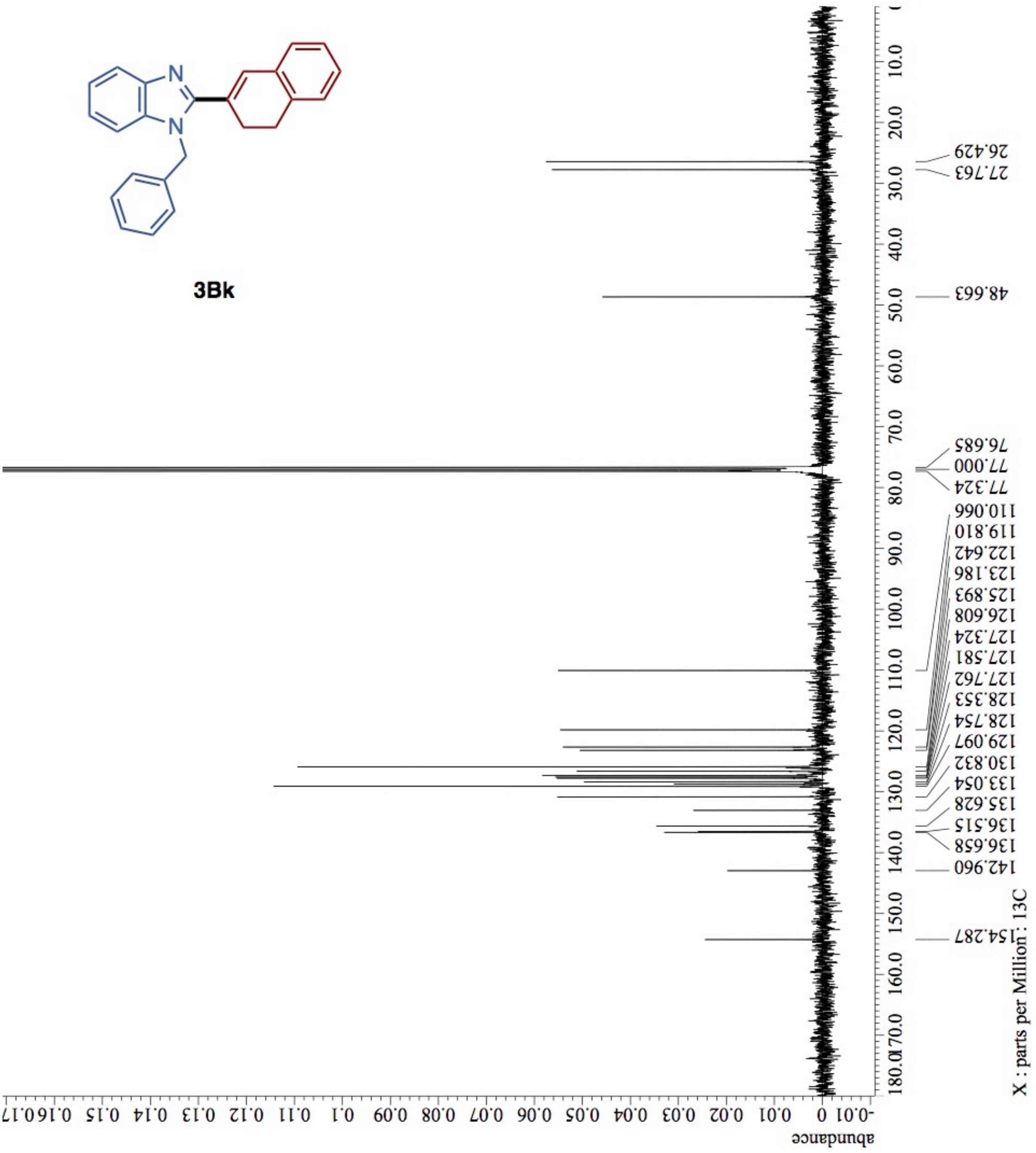

^{1}H NMR of **3Dk** (400 MHz, $CDCl_3$)

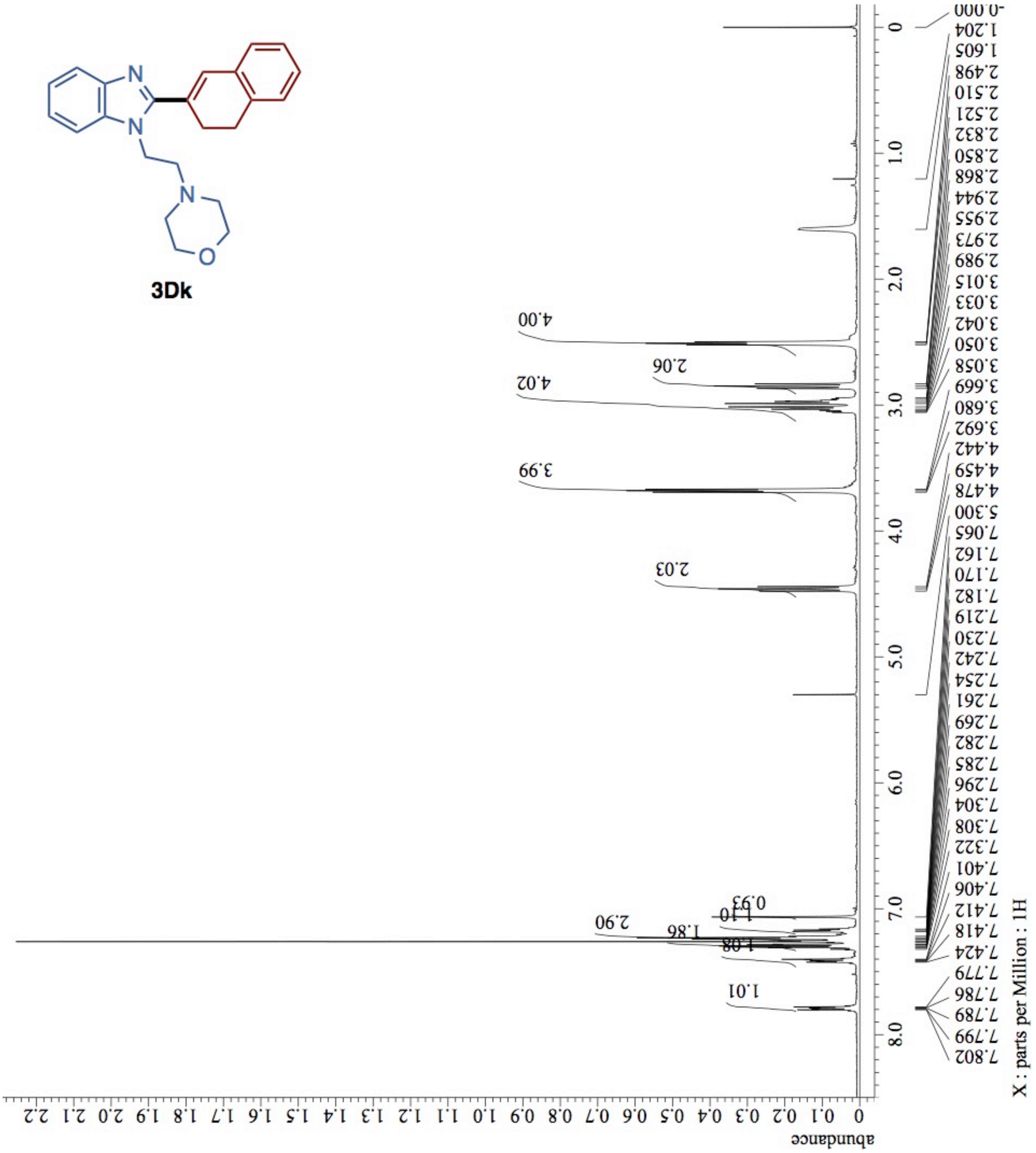

^{13}C NMR of **3Dk** (100 MHz, $CDCl_3$)

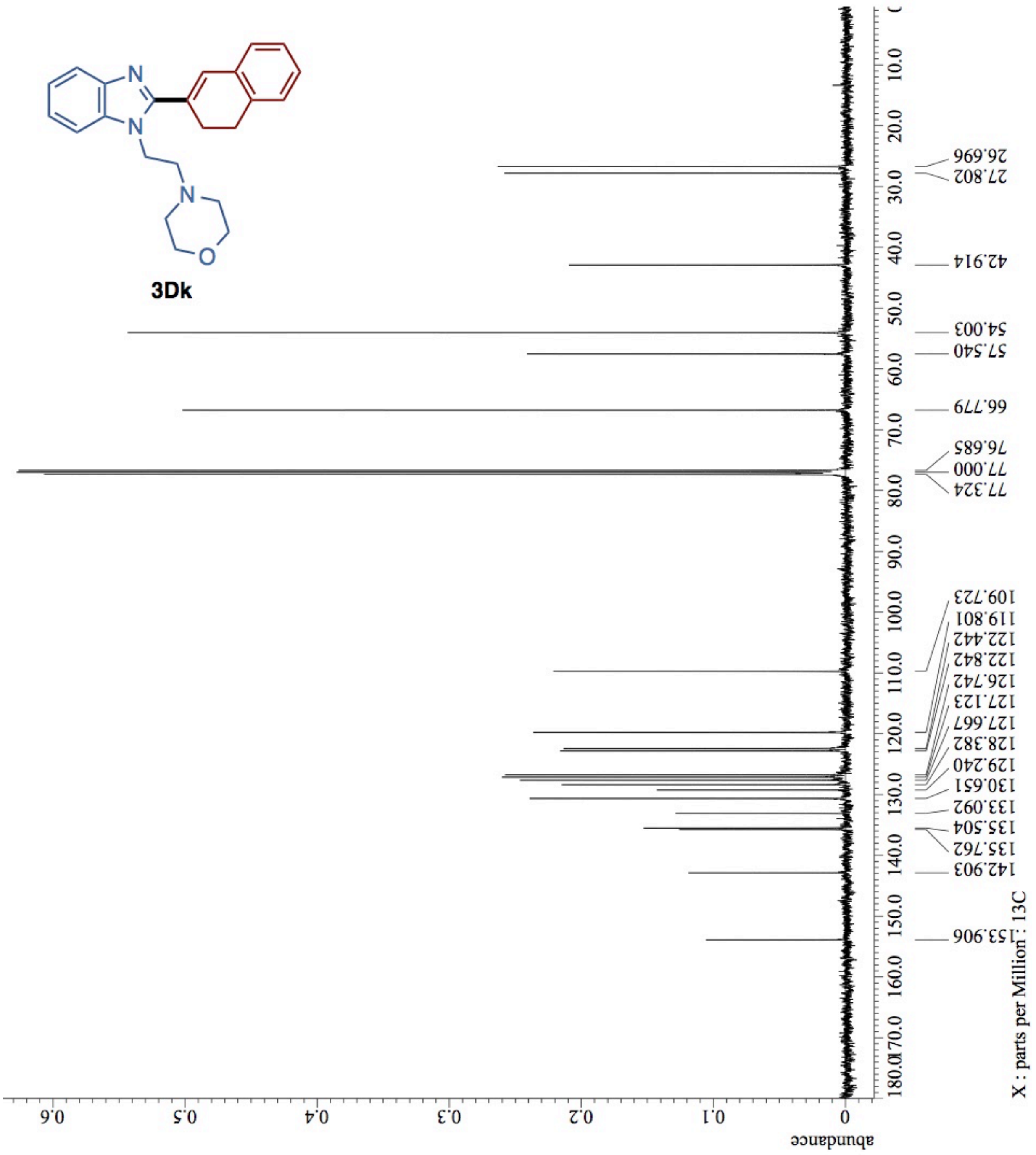

^{1}H NMR of **3Rk** (400 MHz, $CDCl_3$)

3Rk

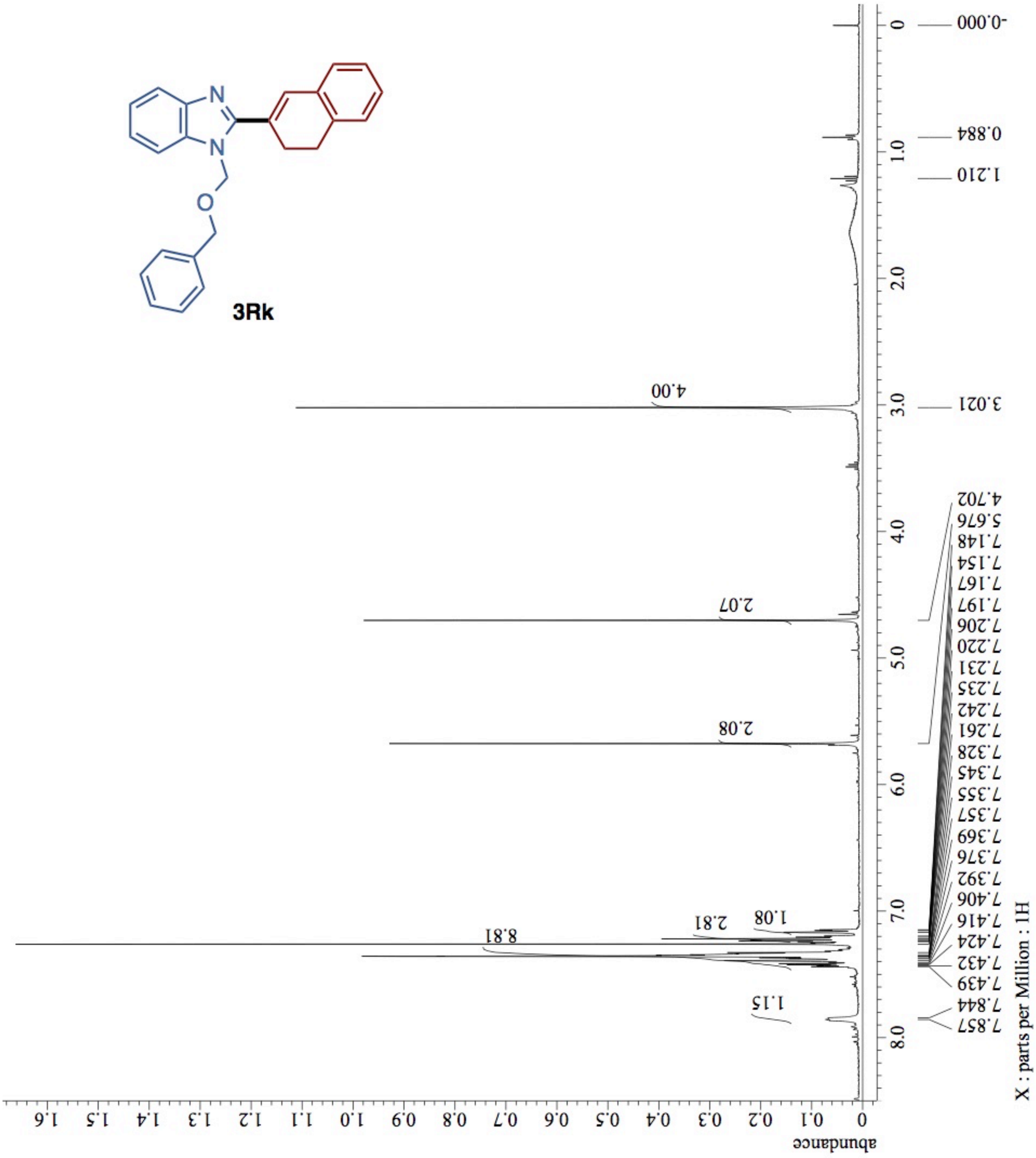

^{13}C NMR of **3Rk** (100 MHz, $CDCl_3$)

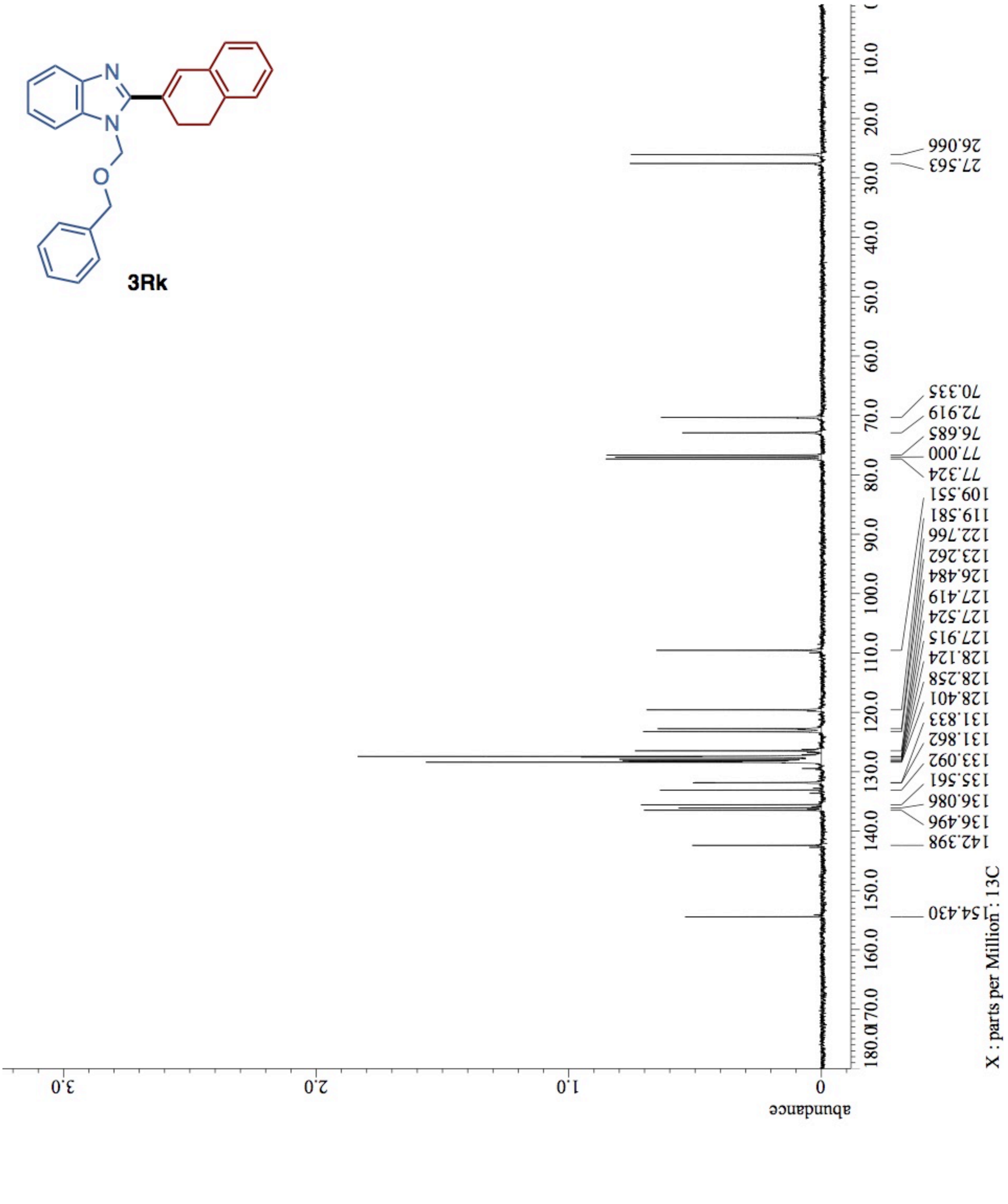

^{1}H NMR of **3Aq** (400 MHz, $CDCl_3$)

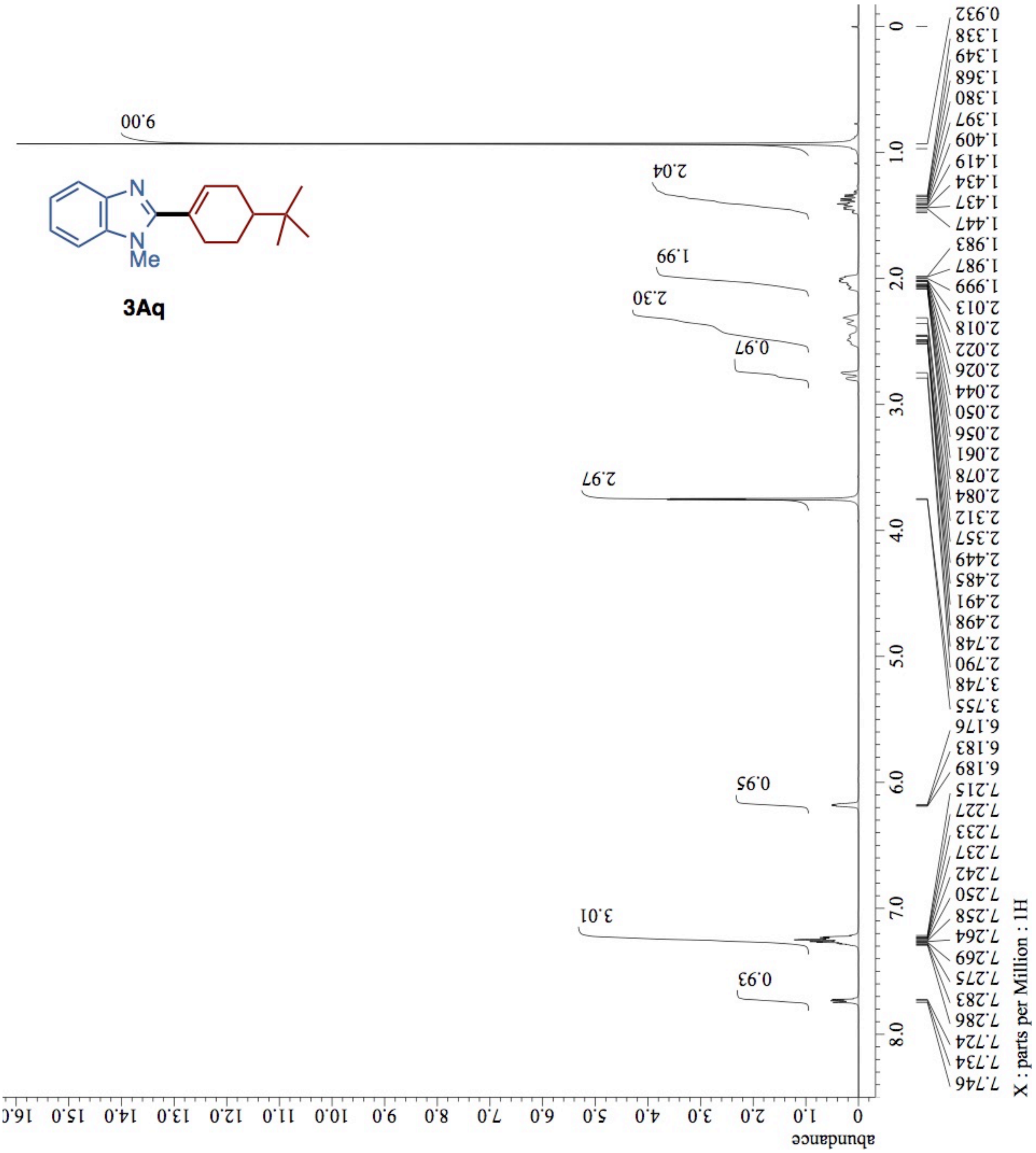

^{13}C NMR of **3Aq** (100 MHz, CDCl$_3$)

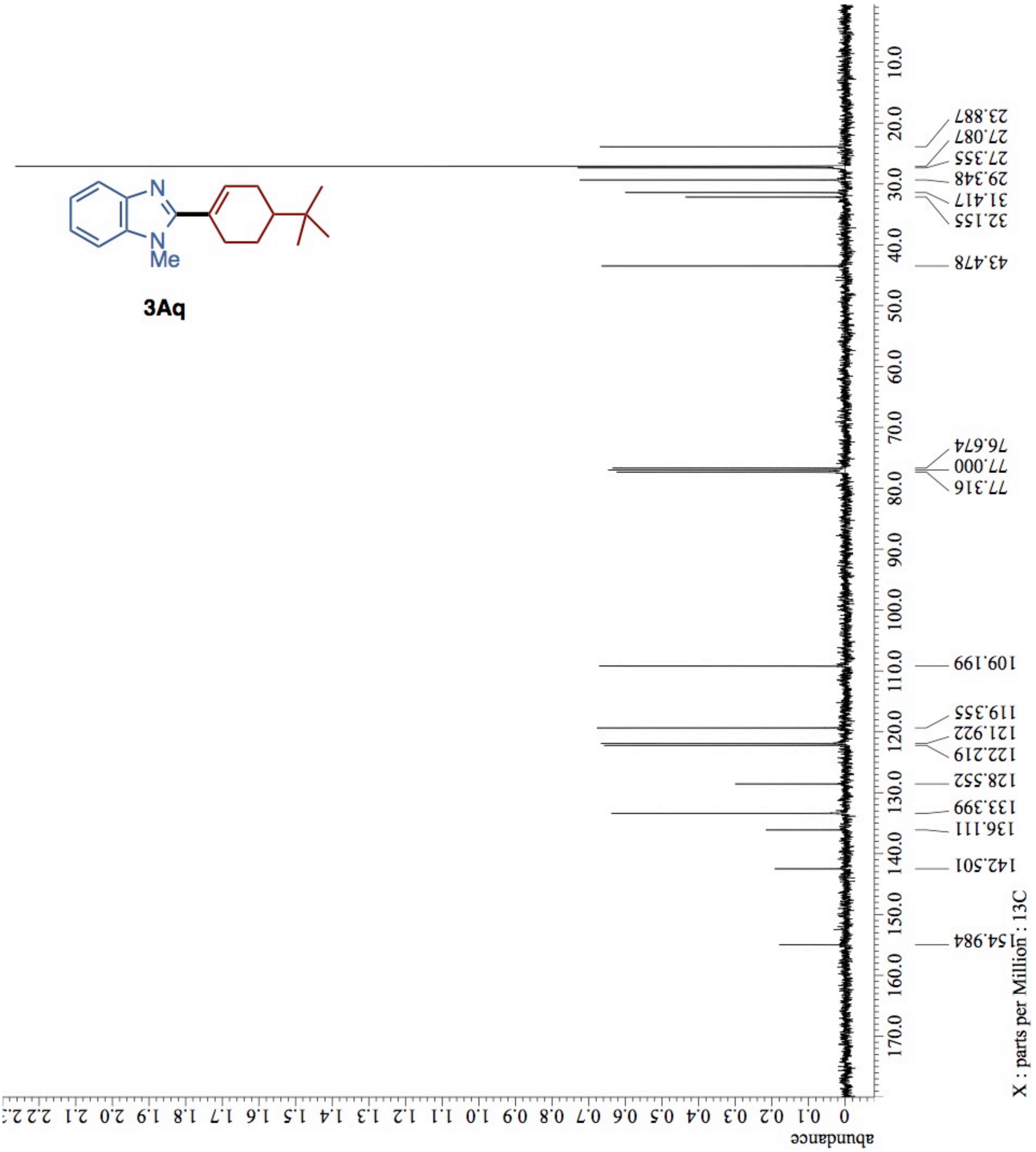

^{1}H NMR of **3Ar** (400 MHz, $CDCl_3$)

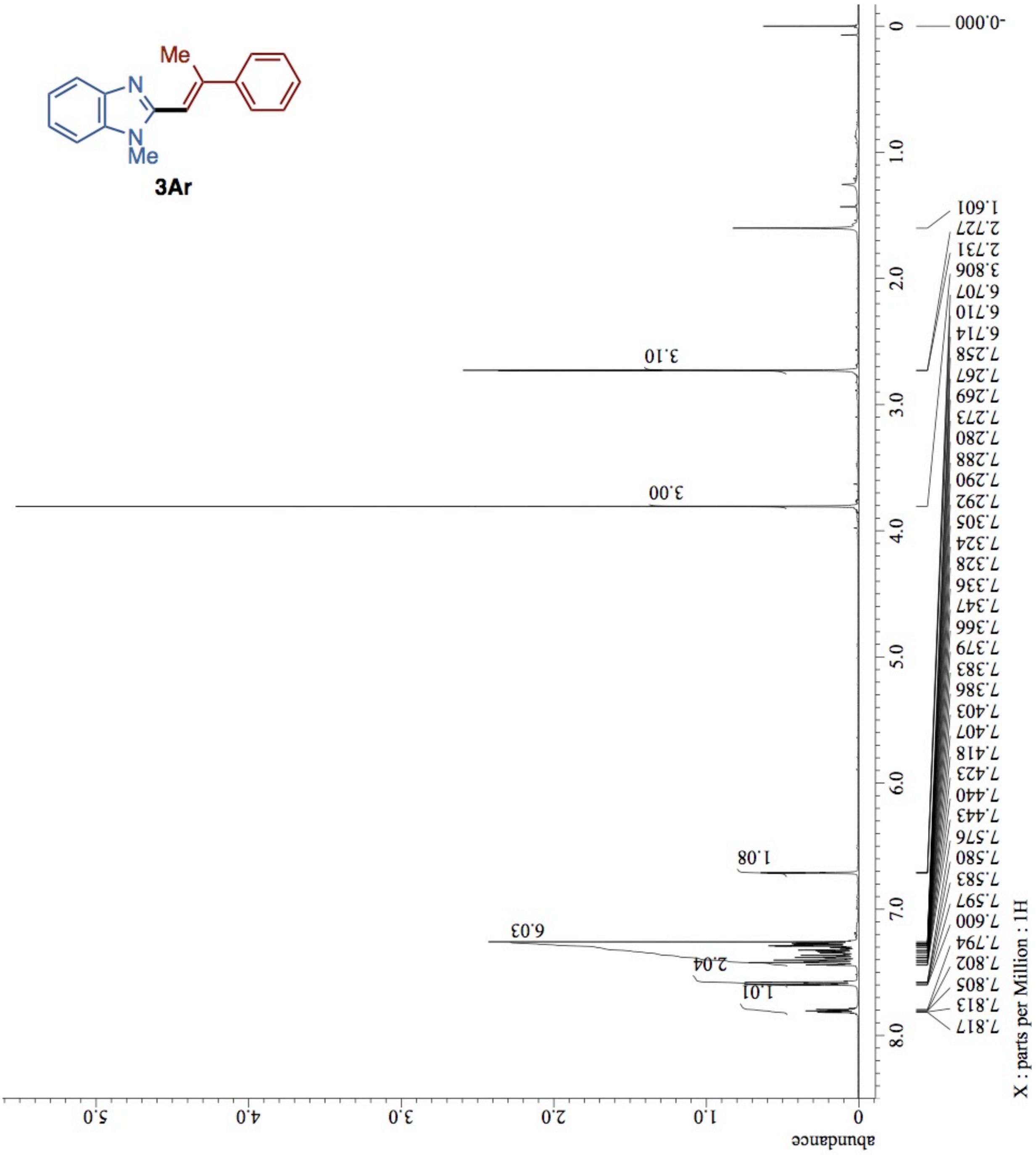

^{13}C NMR of **3Ar** (100 MHz, $CDCl_3$)

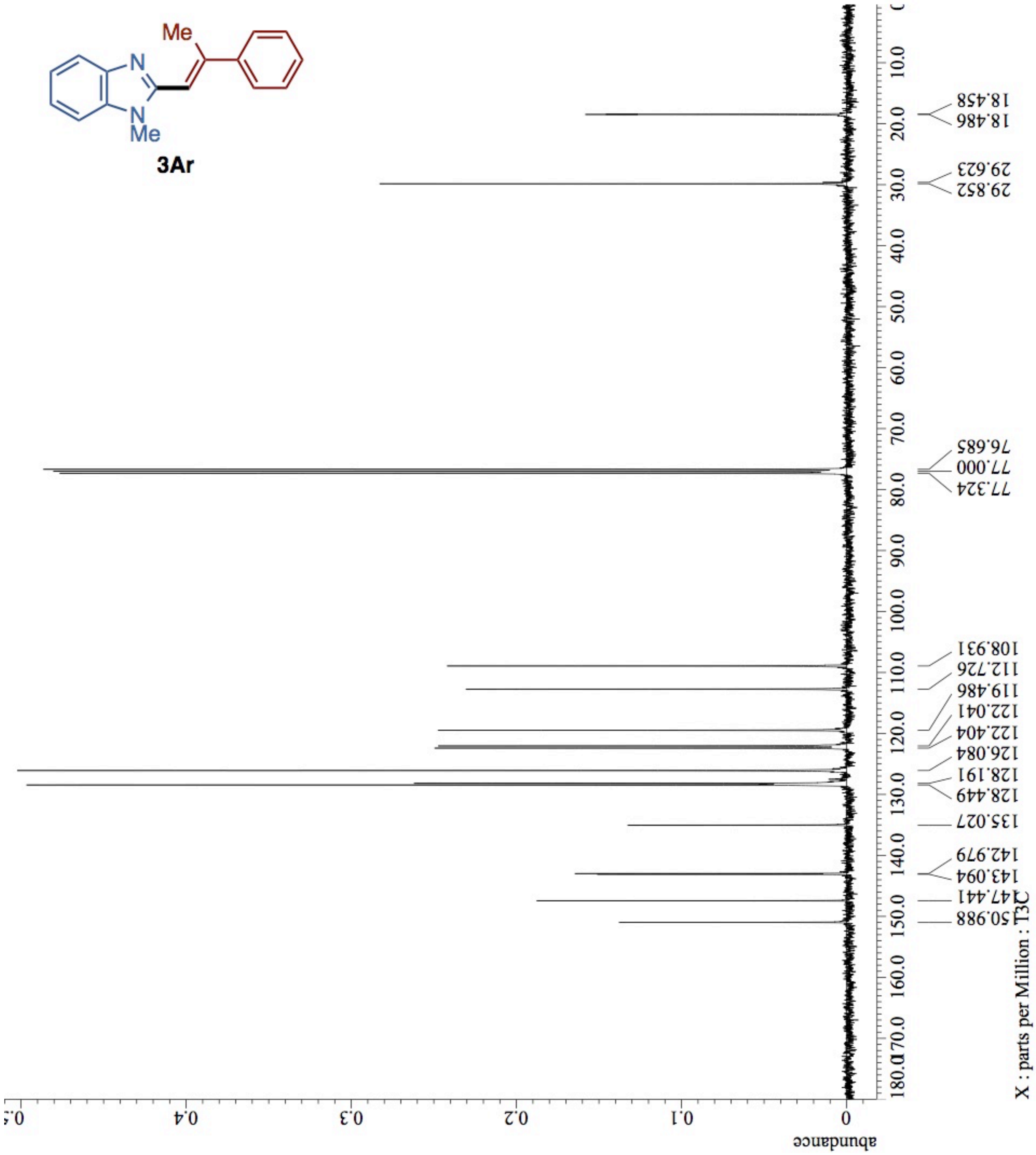

^{1}H NMR of **6Ab** (400 MHz, $CDCl_3$)

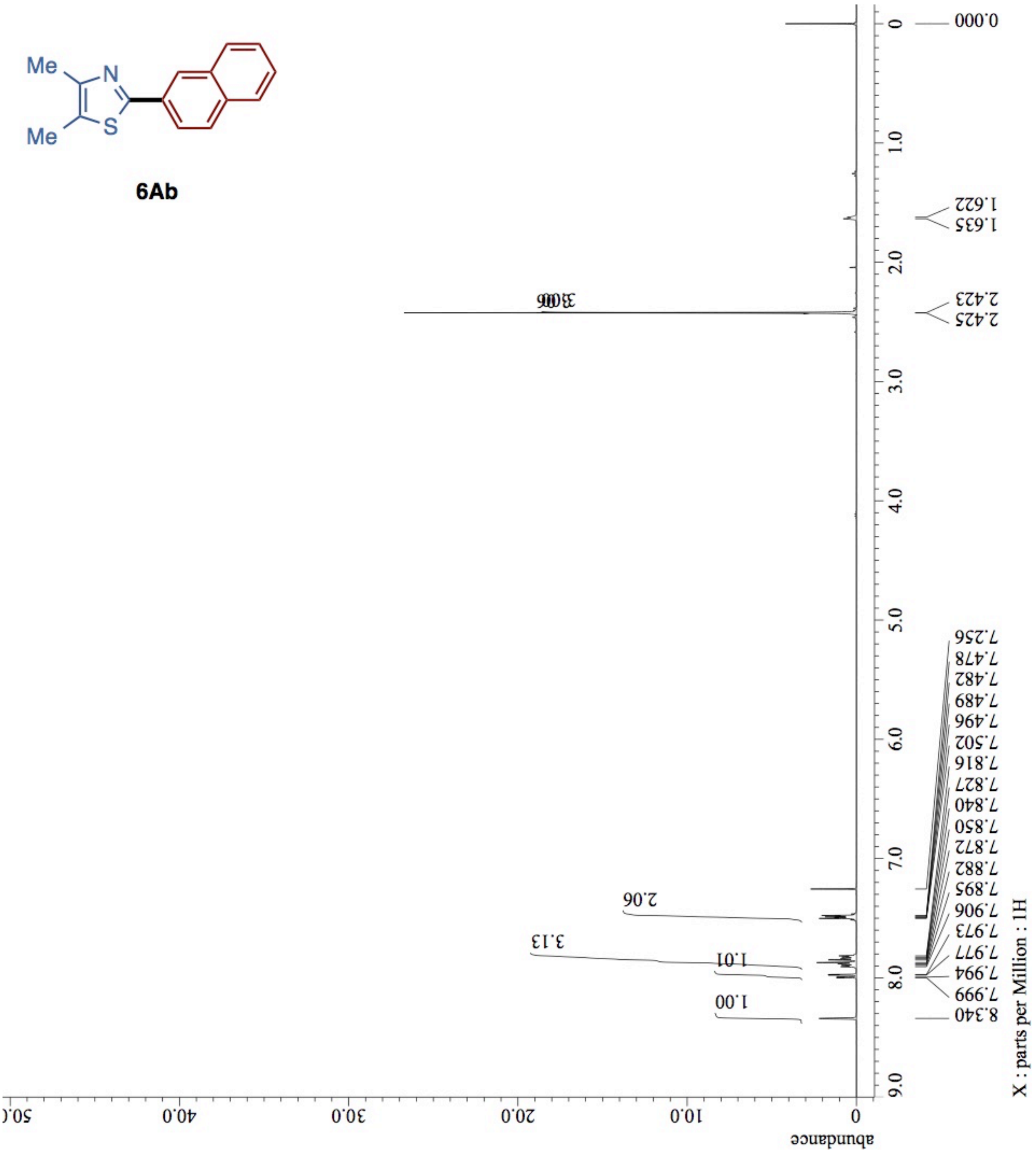

^{13}C NMR of **6Ab** (100 MHz, $CDCl_3$)

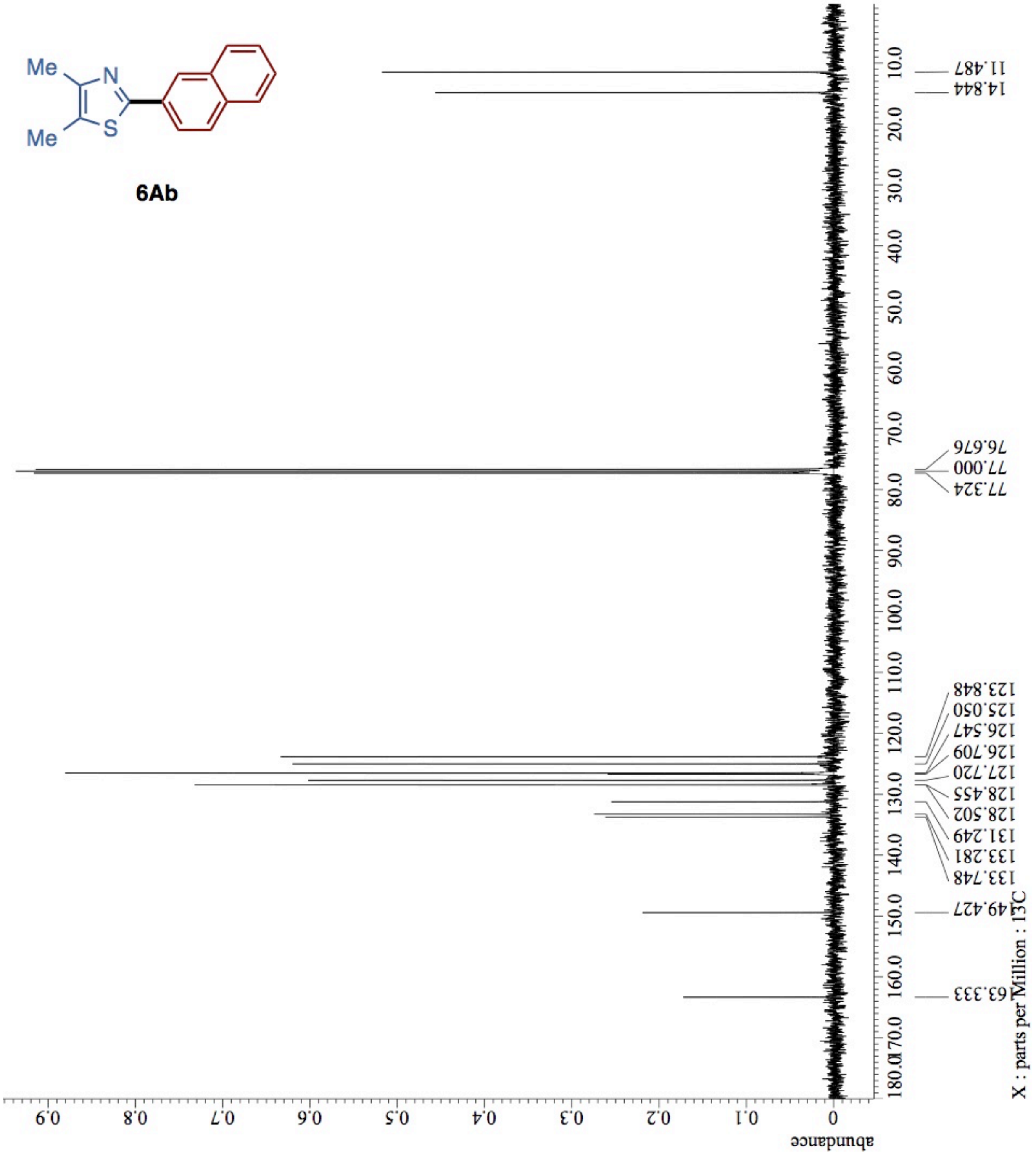

^{1}H NMR of **6Aa** (400 MHz, $CDCl_3$)

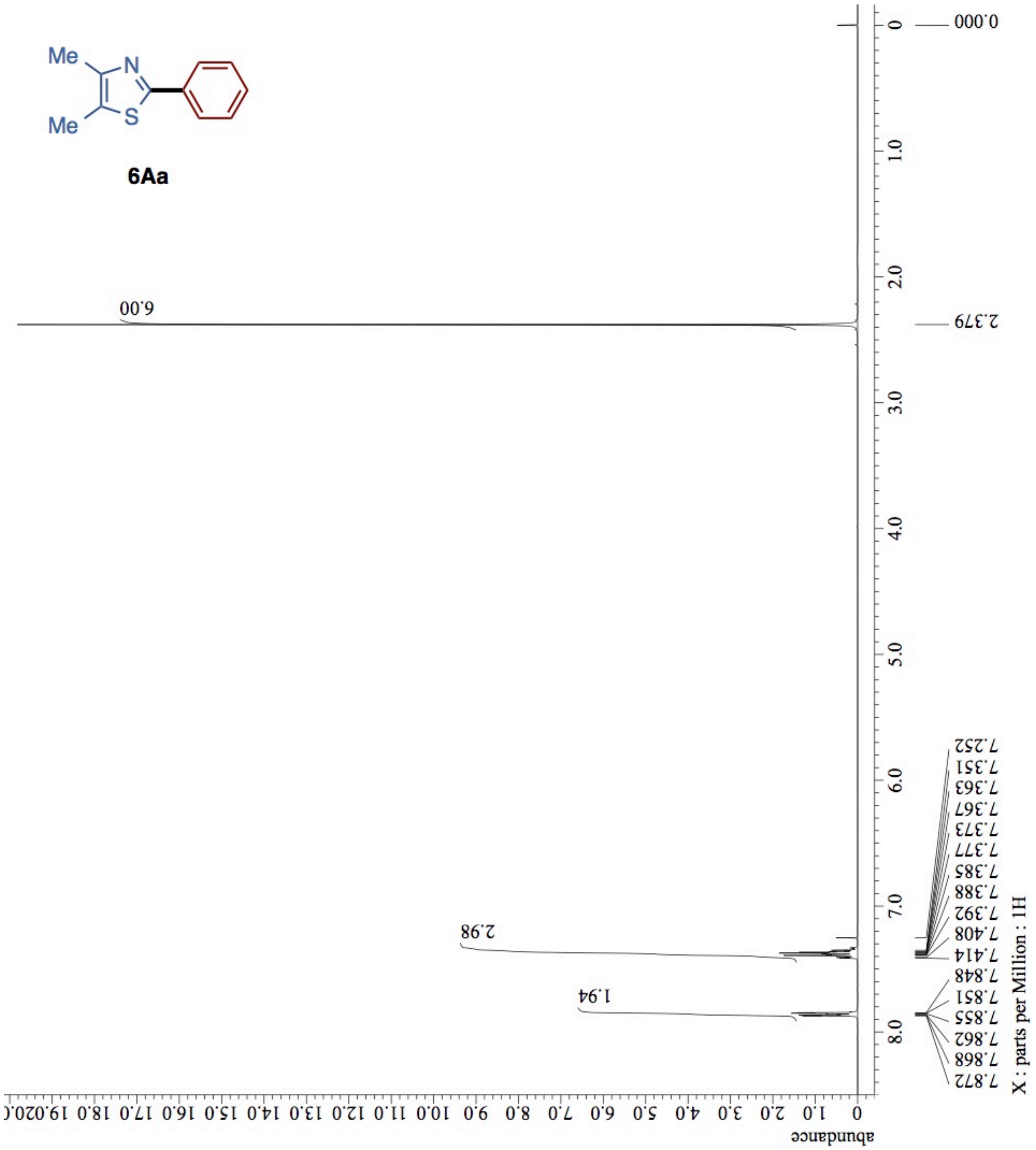

^{13}C NMR of **6Aa** (100 MHz, $CDCl_3$)

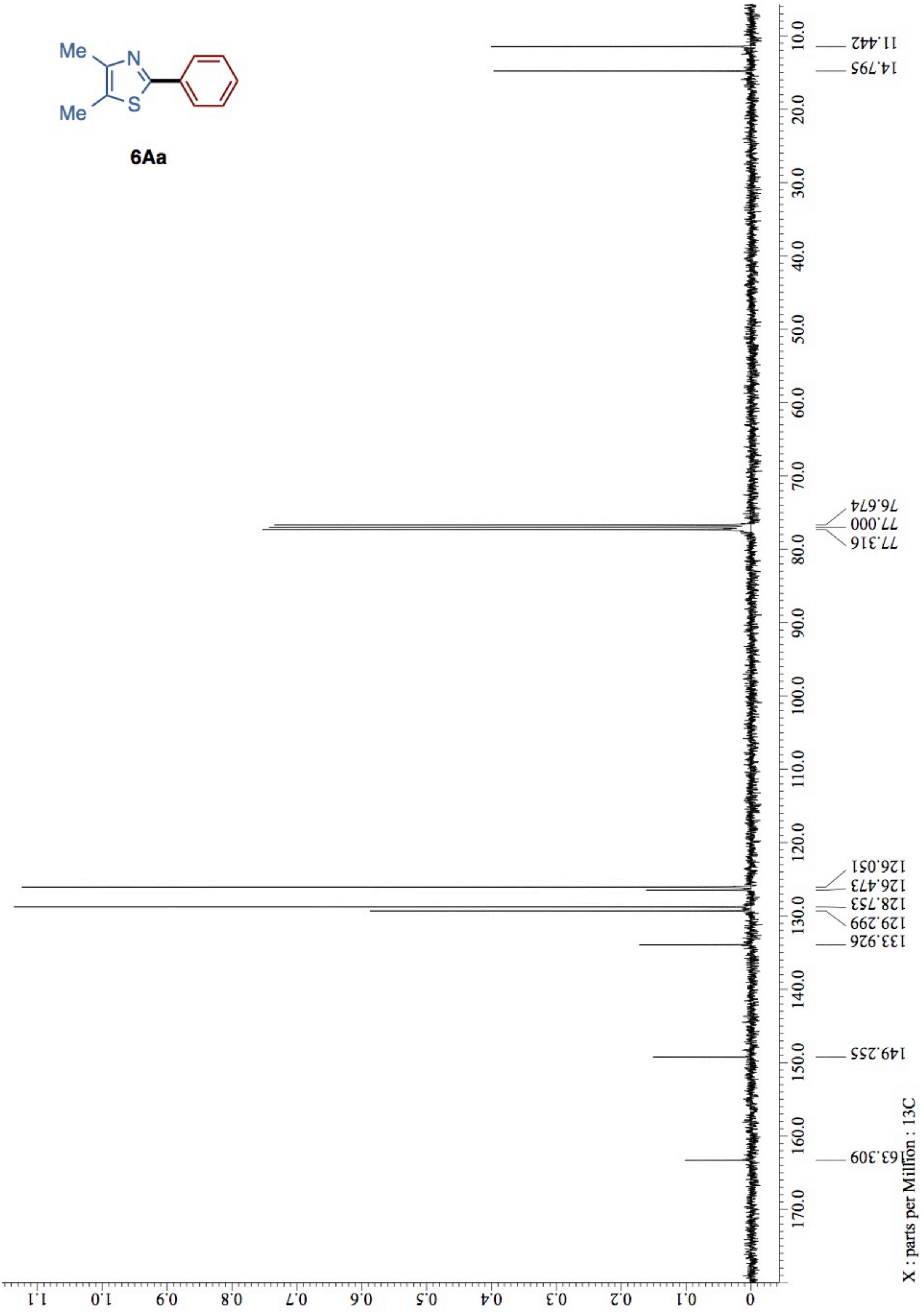

^{1}H NMR of **6Ba** (400 MHz, $CDCl_3$)

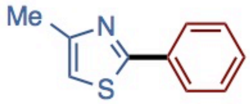

6Ba

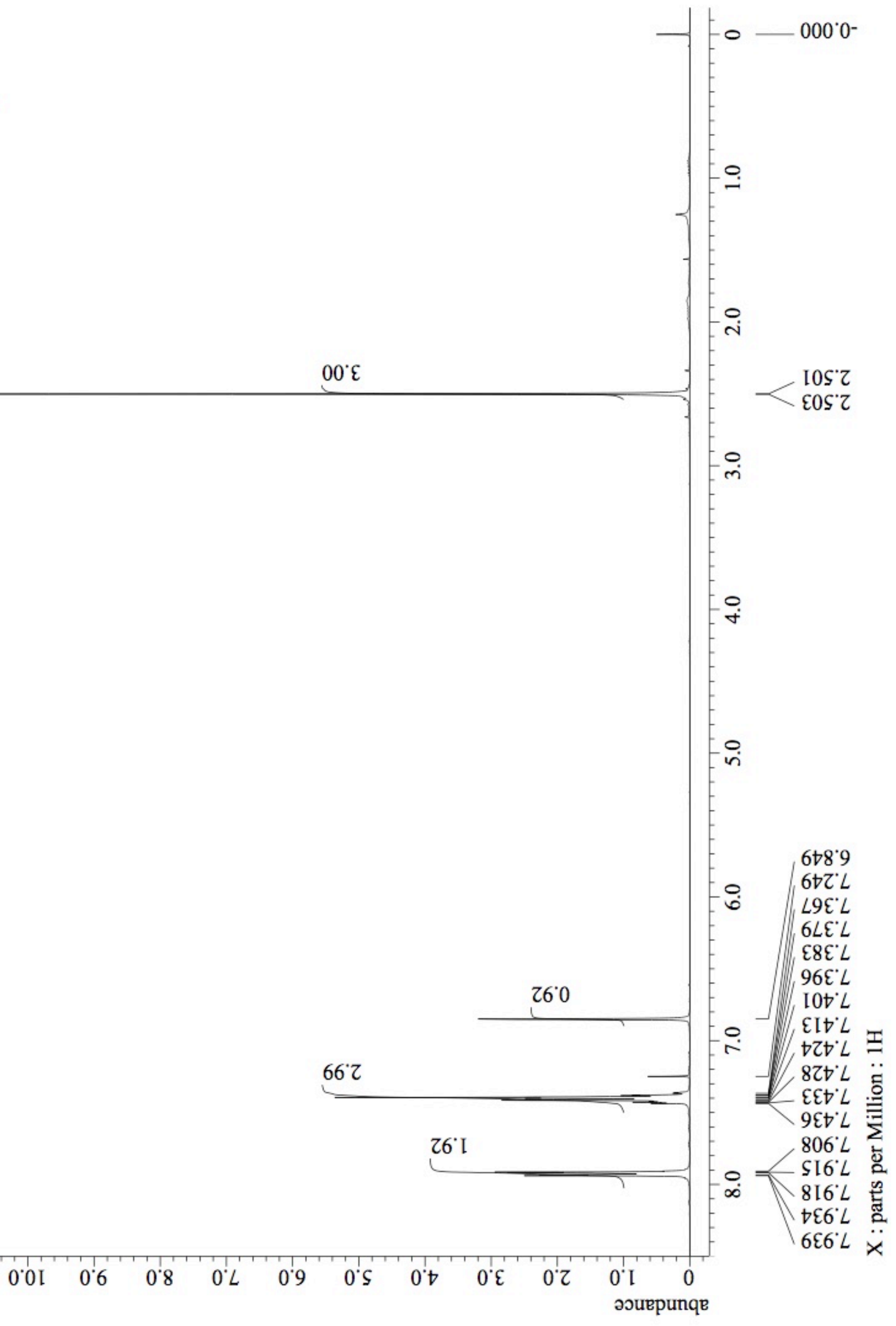

^{13}C NMR of **6Ba** (100 MHz, $CDCl_3$)

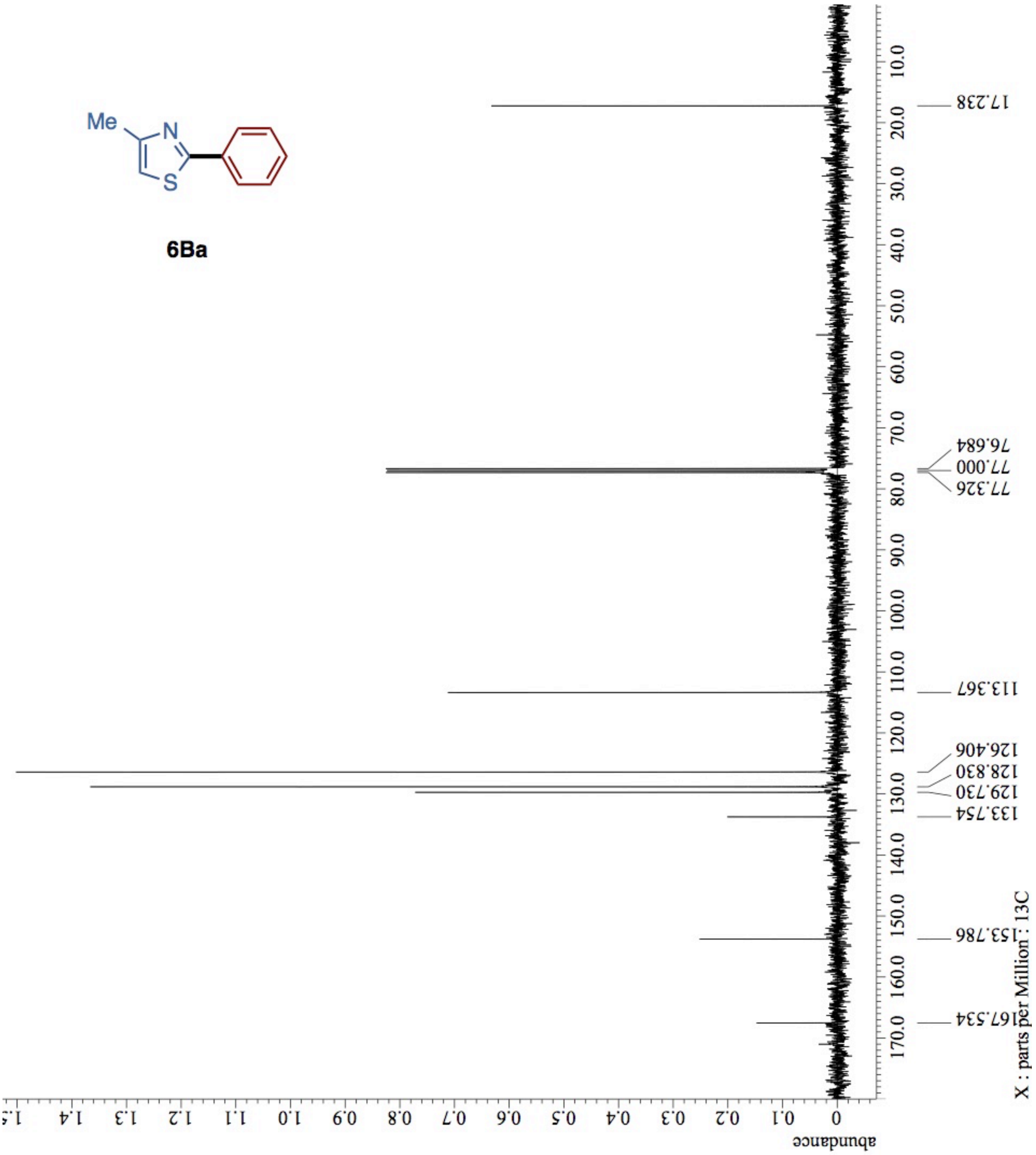

^{1}H NMR of **6Ak** (400 MHz, $CDCl_3$)

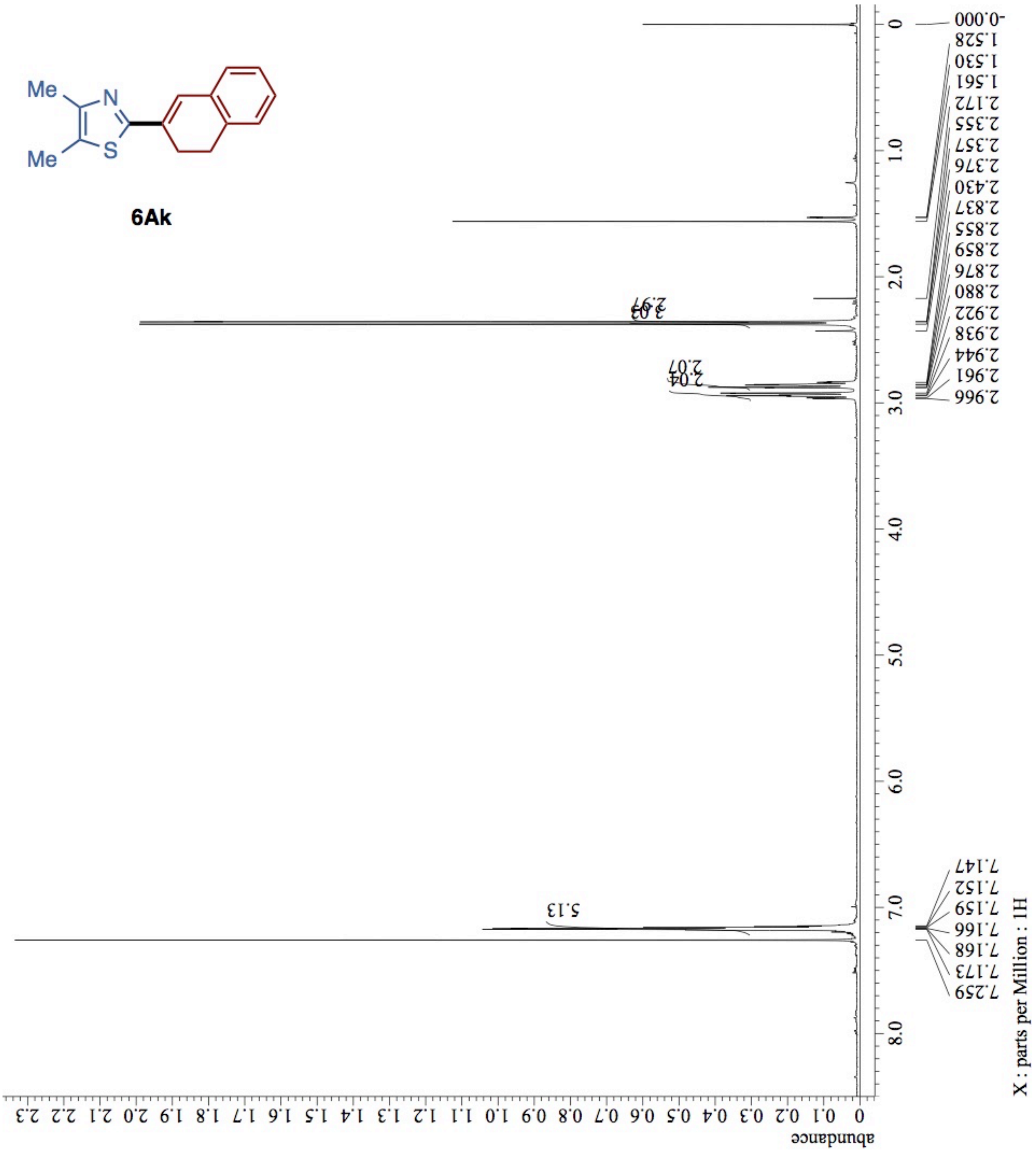

^{13}C NMR of **6Ak** (100 MHz, $CDCl_3$)

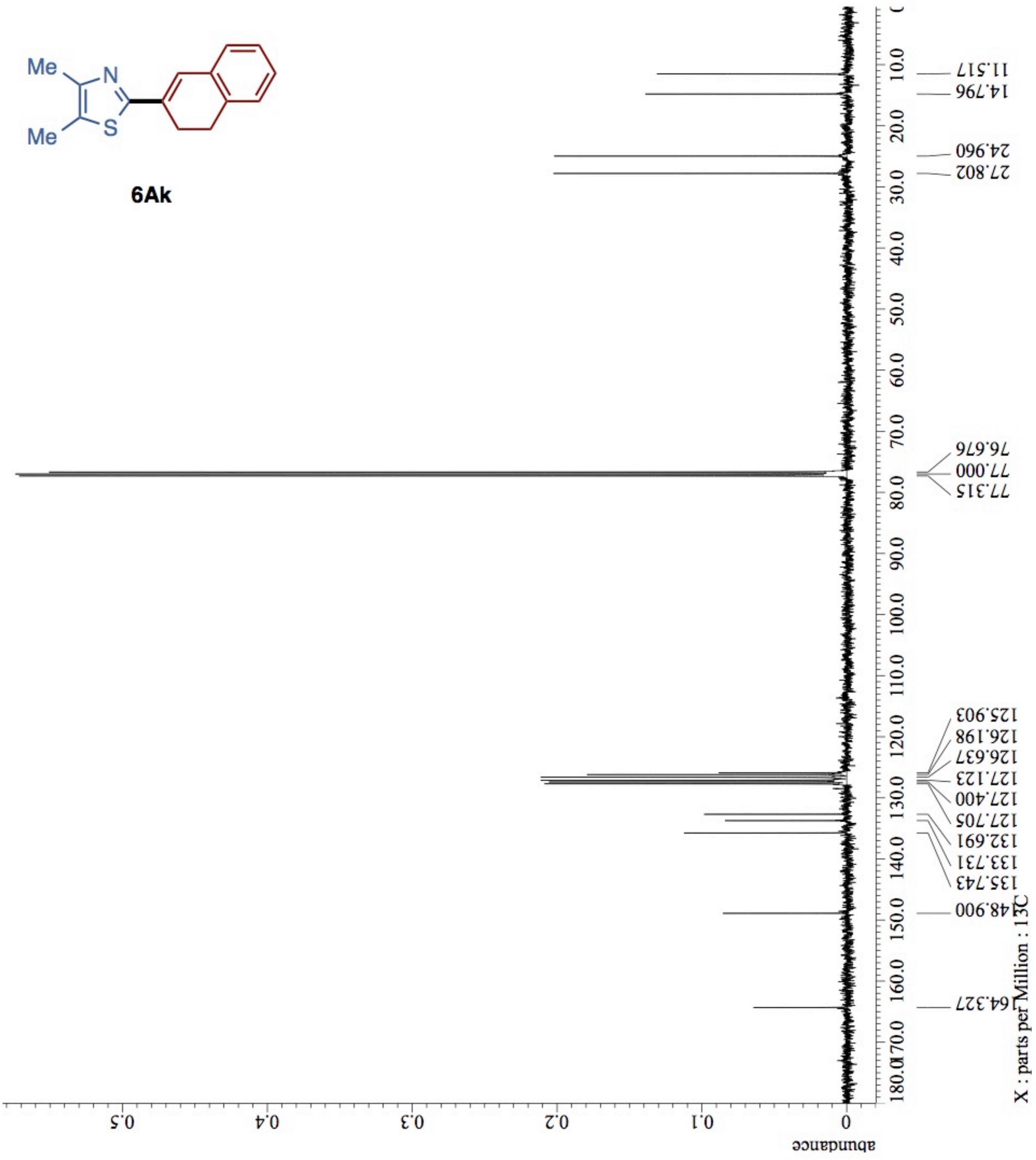

^{1}H NMR of **6Ca** (400 MHz, $CDCl_3$)

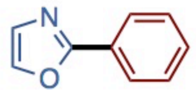

6Ca

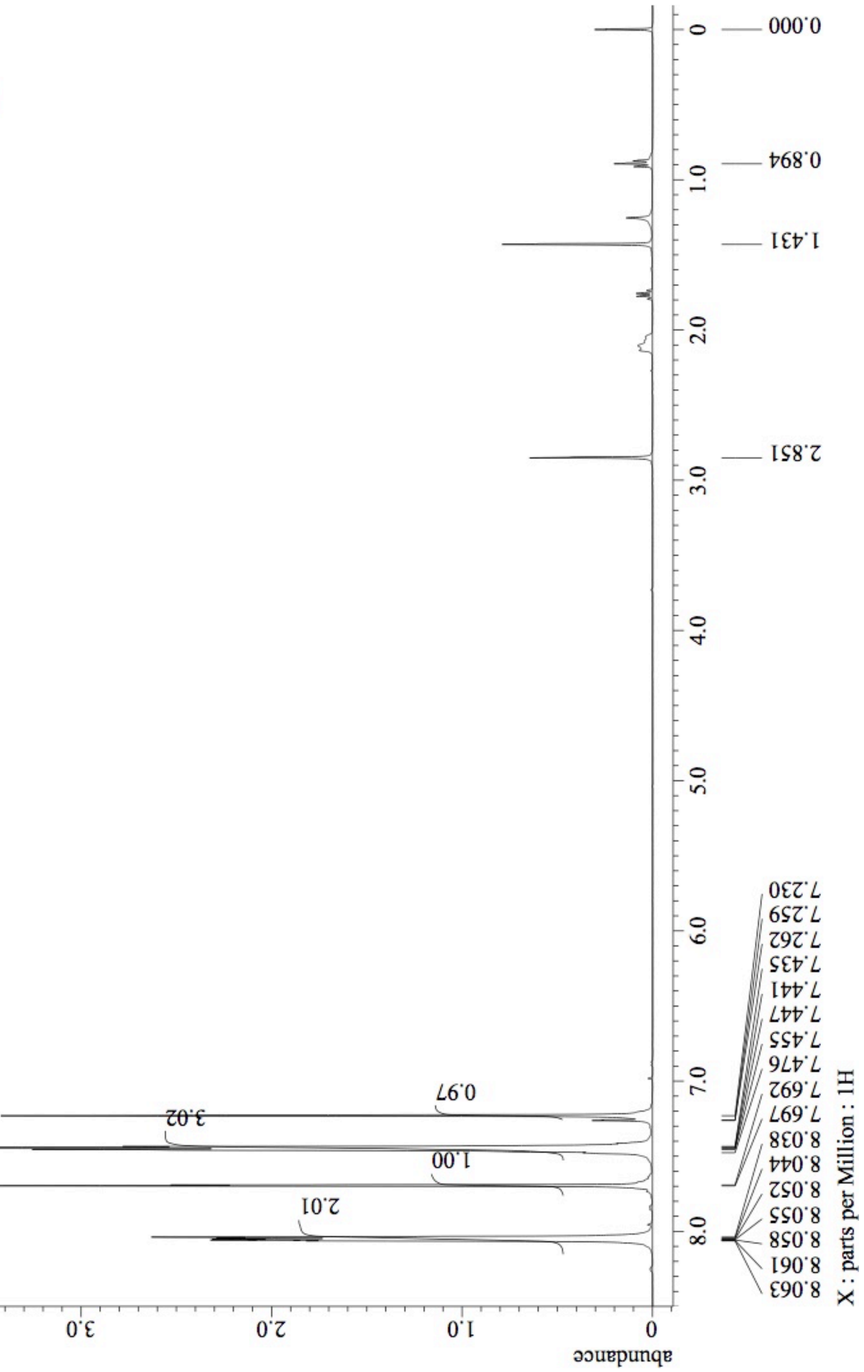

^{13}C NMR of **6Ca** (100 MHz, $CDCl_3$)

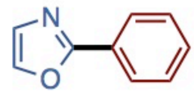

6Ca

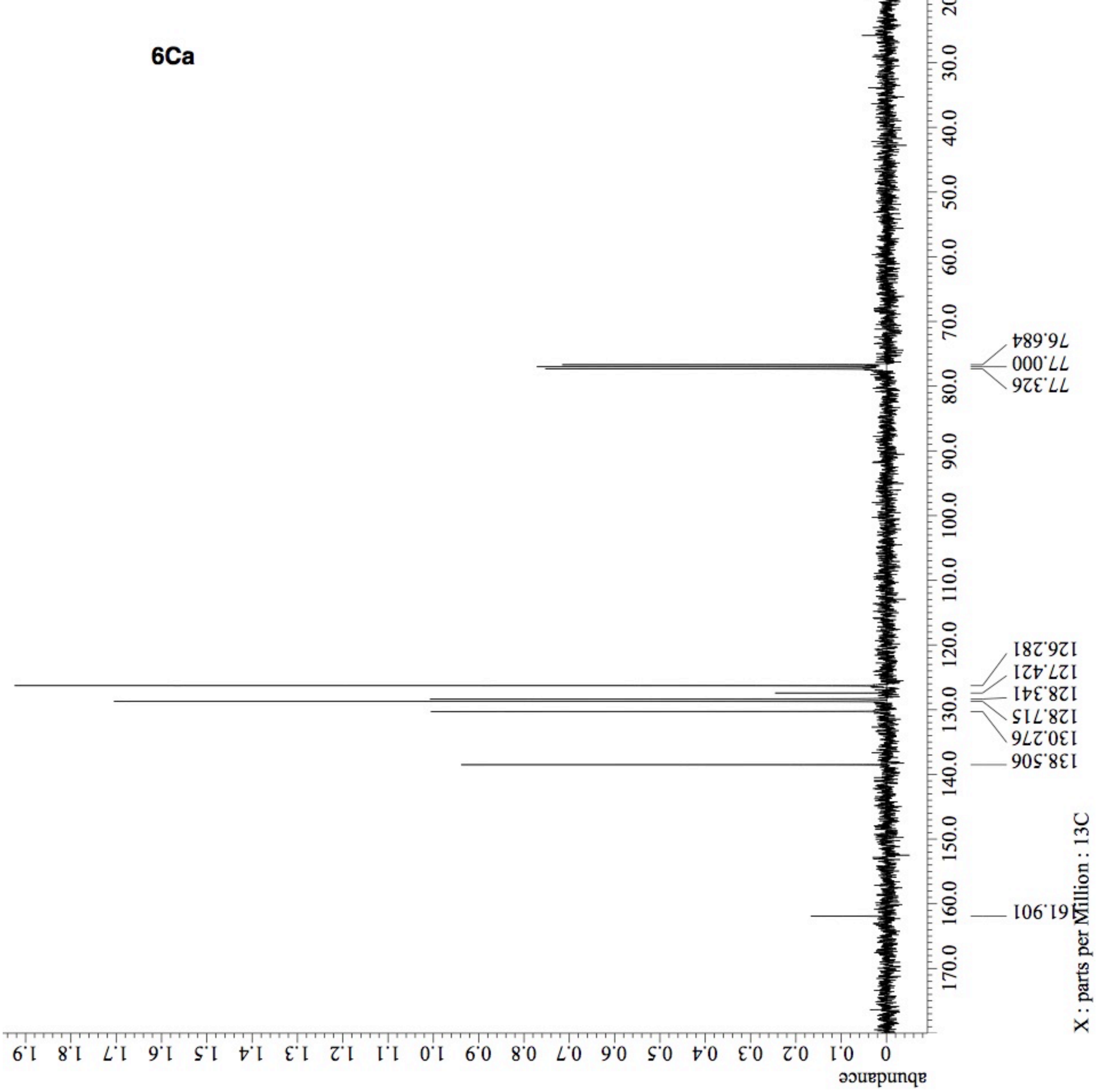

^{1}H NMR of **6Ci** (400 MHz, $CDCl_3$)

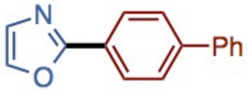

6Ci

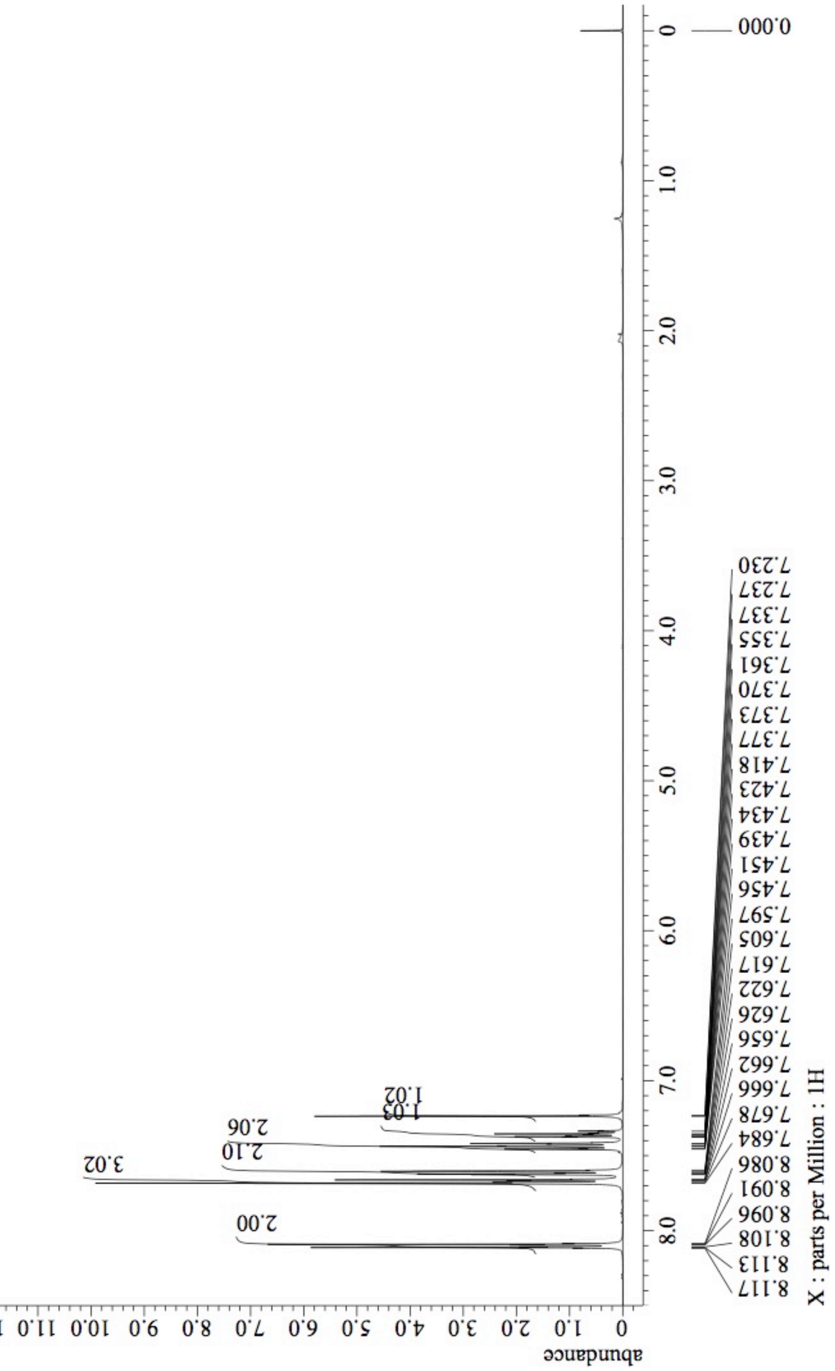

^{13}C NMR of **6Ci** (100 MHz, $CDCl_3$)

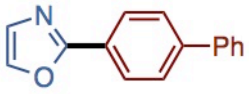

6Ci

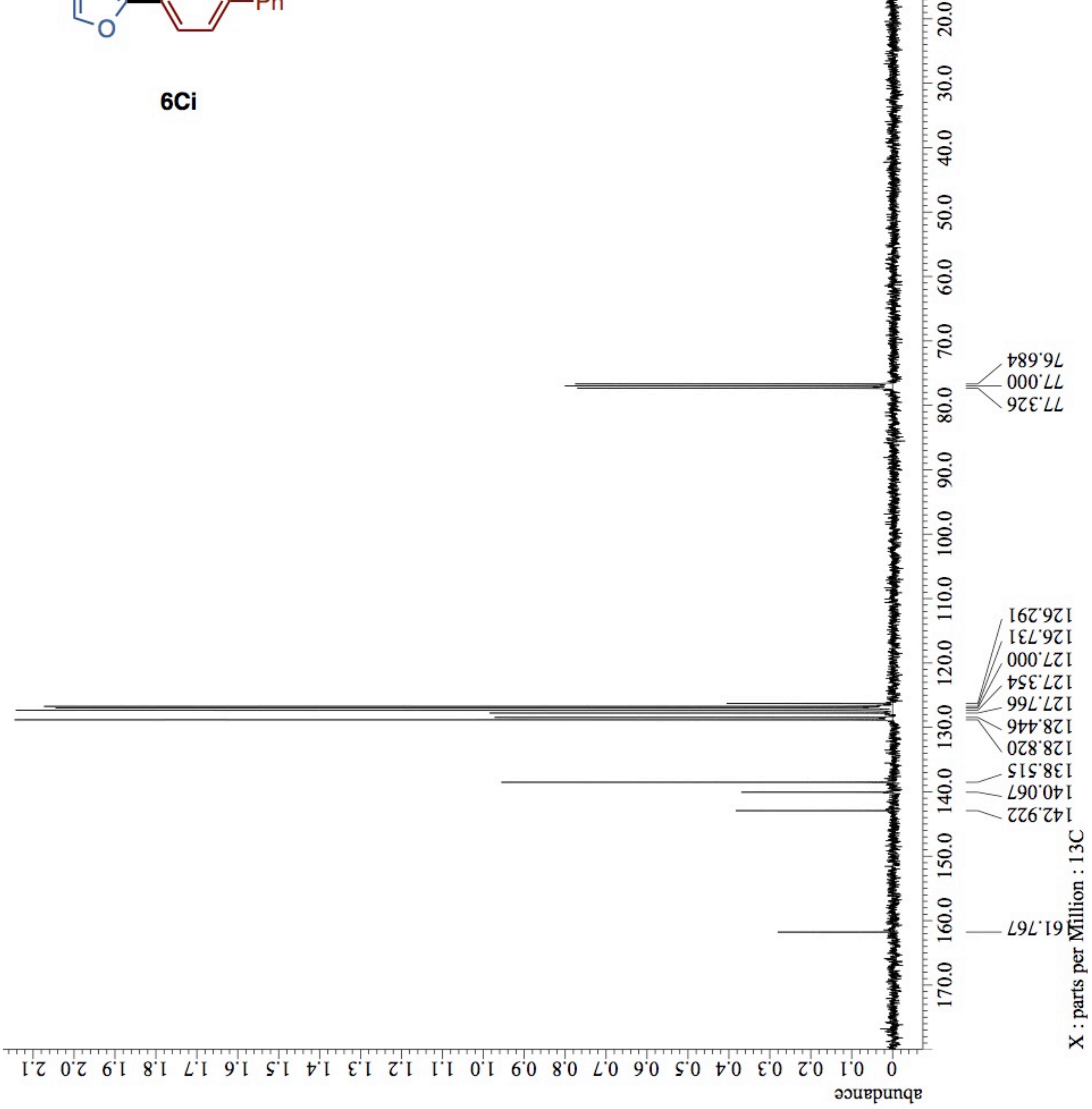

^{1}H NMR of **6Da** (400 MHz, $CDCl_3$)

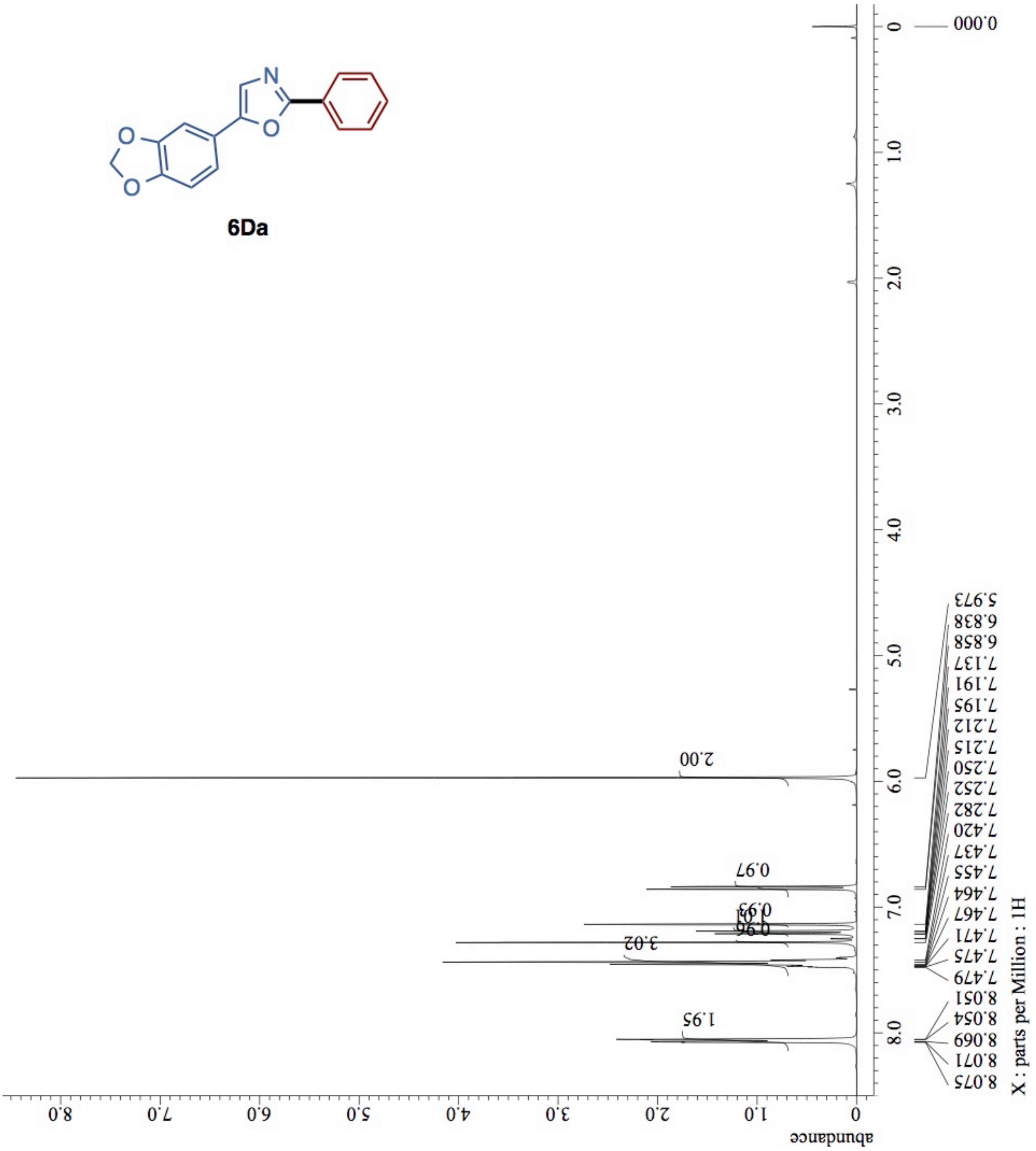

^{13}C NMR of **6Da** (100 MHz, $CDCl_3$)

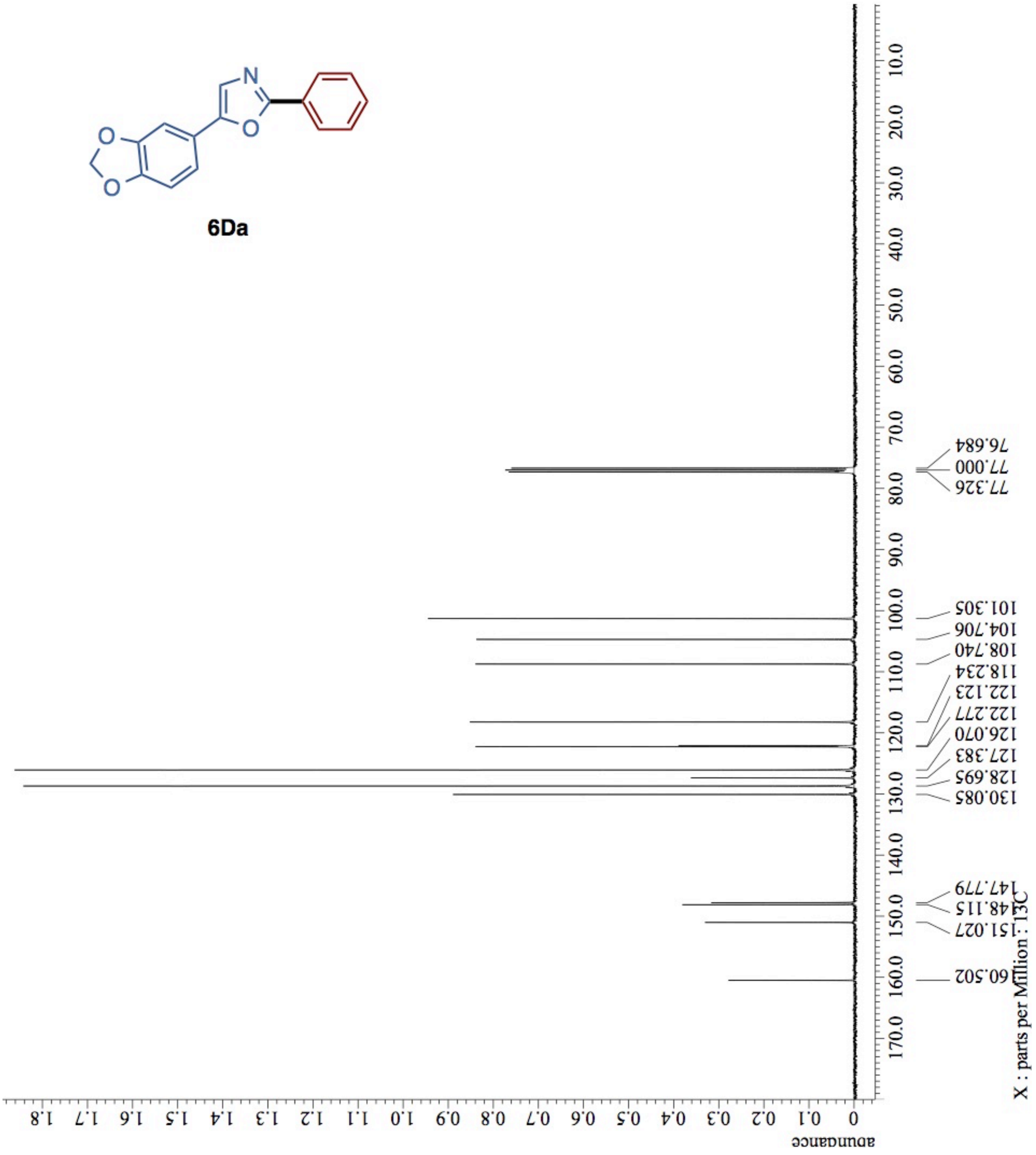

6Da
